# Differential effects of HDAC8 targeting on Foxp3^+^ Tregs and effector T cells promote antitumor immunity

**DOI:** 10.1172/jci.insight.186461

**Published:** 2025-12-11

**Authors:** Fanhua Kong, Yan Xiong, Liqing Wang, Rongxiang Han, Hossein Fazelinia, Jennifer Roof, Lynn Spruce, Aaron B. Beeler, Wayne W. Hancock

**Affiliations:** 1 Zhongnan Hospital of Wuhan University, Institute of Hepatobiliary Diseases of Wuhan University, Transplant Center of Wuhan University, Wuhan University, Wuhan, China.; 2 Department of Kidney Transplantation, Center of Organ Transplantation, The Second Xiangya Hospital of Central South University, Changsha, Hunan, China.; 3 Department of Pathology and Laboratory Medicine, Children’s Hospital of Philadelphia, Philadelphia, Pennsylvania, USA.; 4 Proteomics Core, Abramson Research Center, Philadelphia, Pennsylvania, USA.; 5 Department of Chemistry, Boston University, Boston, Massachusetts, USA.; 6 Department of Pathology and Laboratory Medicine, University of Pennsylvania, Philadelphia, Pennsylvania, USA.

**Keywords:** Immunology, Oncology, Cancer immunotherapy, T cells

## Abstract

HDAC8, an evolutionarily distinct, X-linked, zinc-dependent class I histone/protein deacetylase, is implicated in developmental disorders, parasitic infections, myopathy, and cancers. Our study demonstrates the important role of HDAC8 in immune cells by conditional targeting of HDAC8 in murine T cells and application of selective HDAC8 inhibitors. Using flow cytometry, RNA-seq, and ChIP-seq analyses, we demonstrate that knocking down or inhibiting HDAC8 impaired murine regulatory T cell (Treg) suppressive function in vitro and in vivo, but promoted conventional host T cell responses, thereby limiting syngeneic tumor growth. Mechanistically, HDAC8 knockout downregulated Foxp3 expression, enhanced H3K27 acetylation levels, and promoted IL-2, IL-6, Fas, and FasL expression in both Treg and conventional effector T cells. Thus, our combined genetic and pharmacologic studies establish the central importance of HDAC8 in T cell responses and suggest that selective HDAC8 inhibitors represent a potential therapeutic approach in immuno-oncology.

## Abstract 2

We show HDAC8 is required for normal Foxp3+ Treg function but limits conventional T cell activation such that HDAC8 targeting leads to increased anti-tumor immunity.

## Graphical abstract

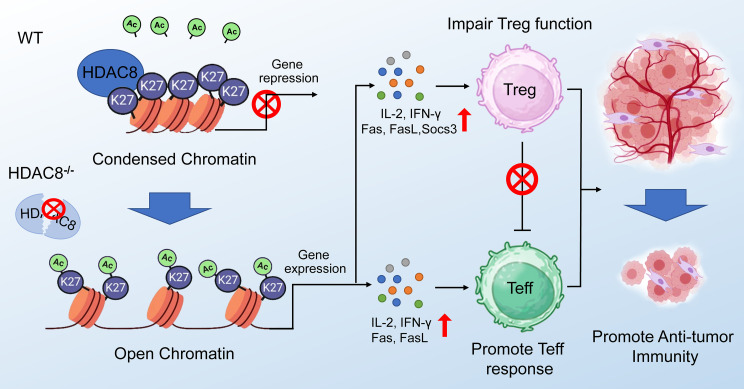

## Introduction

Tumor immunotherapy, particularly through molecular blockade of immune checkpoints like CTLA-4 and PD-1, has shown promising clinical efficacy in various cancer types, including advanced cancers (1). However, long-lasting responses are observed in only approximately 20%–30% of treated patients, underscoring the need for biomarkers to predict clinical response and the development of cancer immunotherapies (2). Regulatory T cells (Tregs), characterized by their expression of CD4, CD25, and the X-linked transcription factor Forkhead box P3 (Foxp3), are essential for maintaining immune homeostasis and inducing immune tolerance (3, 4). By limiting T cell activity, Tregs inhibit the development of autoimmunity but, perhaps critically, can also dampen anticancer immunity (5–7). Hence, knowledge of the assembly and functions of multiprotein complexes containing Foxp3 may provide important therapeutic targets in patients with cancer (8, 9).

Human tumors frequently exhibit dysregulation of epigenetic enzymes, arising from mutations, altered expression, or inappropriate recruitment to specific genomic regions. The identification of these enzymes and their molecular chaperones has been pivotal in driving the swift development of small-molecule inhibitors that modulate the cancer epigenome (10). Histone posttranslational modifications (PTMs), including acetylation, phosphorylation, methylation, and ubiquitination, are critical for regulating gene expression by altering chromatin structure. Among these, histone acetylation changes chromosome structure and regulates gene expression (11). Histone deacetylases (HDACs) remove acetyl groups from lysine residues on histones, leading to the formation of condensed heterochromatin and typically repressing gene expression, but can also target nonhistone proteins, regulating key biochemical functions such as mRNA and protein stability, and enzyme activity (12).

Upregulation of HDAC expression is common in advanced cancers and correlates with poor patient prognosis, indicating HDAC involvement in multiple cancer stages. X-linked HDAC8, a member of the class I family of HDACs, was discovered in 2000 and has been widely studied in both structure and function (13), and is thought to act in vivo primarily by catalyzing the deacetylation of nonhistone proteins such as p53 and structural maintenance of chromosomes 3 (SMC3) (14–16). Its pharmacological inhibition has gained interest in cancer settings due to its aberrant expression and functional importance in various malignancies, including colorectal and lung cancers. Numerous studies have confirmed a role for HDAC8 as an oncogene in various tumors, including neuroblastoma, T cell lymphomas, and gastric, liver, colorectal, lung, and breast cancers (15–20). Additionally, HDAC8 is implicated in immune evasion strategies. For instance, inhibition of HDAC8 activity promotes macrophage switching to a proinflammatory/antitumor phenotype and enhances the expression of natural killer (NK) group 2D ligands, triggering NK cell cytotoxic activity (21). In the tumor microenvironment, HDAC8 inhibition restores H3K27 acetylation, promotes the production of T cell–directed chemokines, and enhances CD8^+^ T cell infiltration within tumors, thereby promoting antitumor immunity (20). However, the functions of HDAC8 in Foxp3^+^ Tregs remain unknown.

The current study highlights an important role of HDAC8 in immune cells by presenting data on the conditional targeting of HDAC8 in T cells and the effects of selective HDAC8 inhibitors (HDAC8is) on Tregs and T cell–dependent immune responses. These findings may contribute to developing more effective strategies for cancer immunotherapy.

## Results

Prior studies delineated oncogenic signaling cascades induced by aberrant HDAC8 expression in cancer cells (22), but no role for HDAC8 in antitumor immune responses was identified. Using The Cancer Genome Atlas (TCGA), tumor immune estimation resource, version 2 (TIMER2), and other databases, we found that HDCA8 was upregulated in most human solid tumors (Supplemental Figure 1; supplemental material available online with this article; https://doi.org/10.1172/jci.insight.186461DS1) and promoted Treg infiltration in tumors (Supplemental Figure 2).This led us to undertake conditional targeting of HDAC8 in T cells, by mating CD4-Cre and HDAC8^fl/fl^ mice to produce what are hereafter termed HDAC8^–/–^ mice, as well as to test the effects of selective HDAC8is on T cell–dependent immune responses in WT mice. Mice bred at normal Mendelian rates, had normal lymphoid development, and under standard lab conditions displayed a normal phenotype and lifespan, without evidence of autoimmunity. To assess the effect of HDAC8 knockdown on T cell proliferation, we analyzed the proliferation of T cells in the thymus, spleen, and lymph nodes of CD4-Cre and HDAC8^–/–^ mice by flow cytometry. As shown in Supplemental Figure 3, genetic deletion of HDAC8 in T cells did not affect the proliferation of CD4^+^ or CD8^+^ T cells (Supplemental Figure 3A), activated CD4^+^CD25^+^ T cells (Supplemental Figure 3B), or CD4^+^Foxp3^+^ Tregs (Supplemental Figure 3C). Moreover, deletion of HDAC8 did not affect the expression of Ki-67 by CD4^+^ T cells (Supplemental Figure 3D). Hence, mice with conditional T cell deletion of HDAC8 are unremarkable.

### Conditional HDAC8 deletion or pharmacologic inhibition of HDAC8 promotes antitumor immunity.

**Figure 1:**
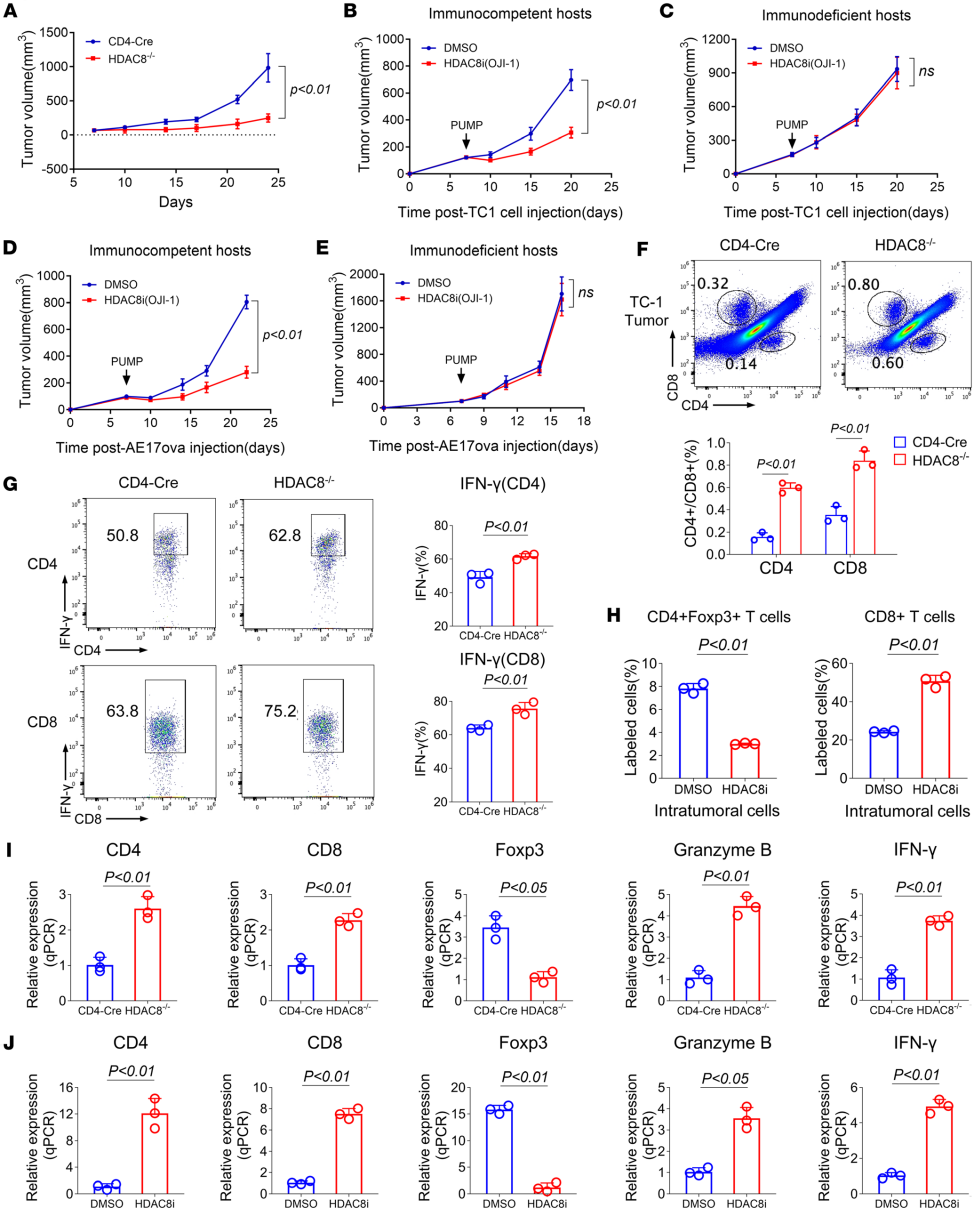
Conditional deletion or inhibition of HDAC8 promotes antitumor immunity in an implantable tumor model involving s.c. injection of TC-1 and AE-17.ova cells. (**A**) Tumor growth in CD4-Cre (*n* = 7) and HDAC8^–/–^ (*n* = 10) mice. (**B**) TC-1 tumor growth in immunocompetent C57BL/6 mice treated with control (diluted DMSO, *n* = 10) or HDAC8i (OJI-1, *n* = 10) via Alzet pump. (**C**) TC-1 tumor growth in immunodeficient C57BL/6 mice treated with control (diluted DMSO, *n* = 10) or HDAC8i (OJI-1, *n* = 10) via Alzet pump. (**D**) AE-17.ova tumor growth in immunocompetent C57BL/6 mice treated with control (diluted DMSO, *n* = 10) or HDAC8i (OJI-1, *n* = 10) via Alzet pump. (**E**) AE-17.ova tumor growth in immunodeficient C57BL/6 mice treated with control (diluted DMSO, *n* = 10) or HDAC8i (OJI-1, *n* = 10) via Alzet pump. (**F**) Flow cytometric analysis of TC-1 intratumoral infiltrating CD4^+^ and CD8^+^ T cells in CD4-Cre (*n* = 3) and HDAC8^–/–^ (*n* = 3) groups. (**G**) Flow cytometry was used to analyze IFN-γ expression by T cells in TC-1 tumors of CD4-Cre (*n* = 3) and HDAC8^–/–^ (*n* = 3) mice. (**H**) Proportion of Foxp3^+^ Tregs and CD8^+^ T cells in TC-1 tumors of mice treated with HDAC8i (OJI-1, 5 mg/kg/d) for 14 days via Alzet pumps (*n* = 3/group). (**I**) qRT-PCR analysis of TC-1 tumor biopsies in CD4-Cre and HDAC8^–/–^ mice (*n* = 3/group). (**J**) qRT-PCR analysis of TC-1 tumor biopsies in DMSO- and HDAC8i-treated (OJI-1, 5 mg/kg/d) mice (*n* = 3/group). Assays were run in triplicate and repeated at least 3 times. The results of a representative experiment are shown. Data are expressed as the mean ± SD of 3 independent experiments. NS, not significant. Comparisons between 2 groups utilized a 2-tailed Student’s *t* test for normally distributed data.

We tested tumor growth by s.c. injection of control CD4-Cre and HDAC8^–/–^ mice with TC-1 lung adenocarcinoma cells (23). Deletion of HDAC8 inhibited TC-1 tumor proliferation (*P* < 0.01, Figure 1A), and when TC-1 tumors were established in WT C57BL/6 and Rag1^–/–^ C57BL/6 mice and treated from day 7 after inoculation with an HDAC8i (OJ-1) (24), HDAC8i therapy inhibited tumor proliferation in immunocompetent (*P* < 0.01, Figure 1B) but not immunodeficient Rag1^–/–^ C57BL/6 mice (Figure 1C). Similar results were observed in the AE-17.ova murine mesothelioma model (25) using immunocompetent and immunodeficient mice (Figure 1, D and E). These findings indicate that the antitumor activity of HDAC8 inhibition is dependent on an adaptive immune response.

Flow cytometric analysis revealed that conditional knockout of HDAC8 resulted in increased infiltration of CD4^+^ and CD8^+^ T cells in TC-1 tumors (*P* < 0.01, Figure 1F), and upregulation of IFN-γ expression in intratumoral CD4^+^ and CD8^+^ T cells (*P* < 0.01, Figure 1G). In addition, the proportion of Foxp3^+^ Tregs was decreased (*P* < 0.01, Figure 1H), and the proportion of CD8^+^ T cells was increased (*P* < 0.01, Figure 1H). Quantitative PCR (qPCR) analysis of TC-1 tumor samples showed that inhibition of HDAC8 deletion led to upregulation of CD4 (*P* < 0.01), CD8 (*P* < 0.01), granzyme B (*P* < 0.01), and IFN-γ (*P* < 0.01) mRNAs, while Foxp3 mRNA was downregulated (*P* < 0.05, Figure 1I). Treatment of TC-1–bearing mice with an HDAC8i produced results consistent with those observed after HDAC8 deletion (Figure 1J). Therefore, HDAC8 targeting increased the infiltration in 2 thoracic tumor models by conventional T cells and diminished accumulation of Foxp3^+^ Tregs.

**Figure 2:**
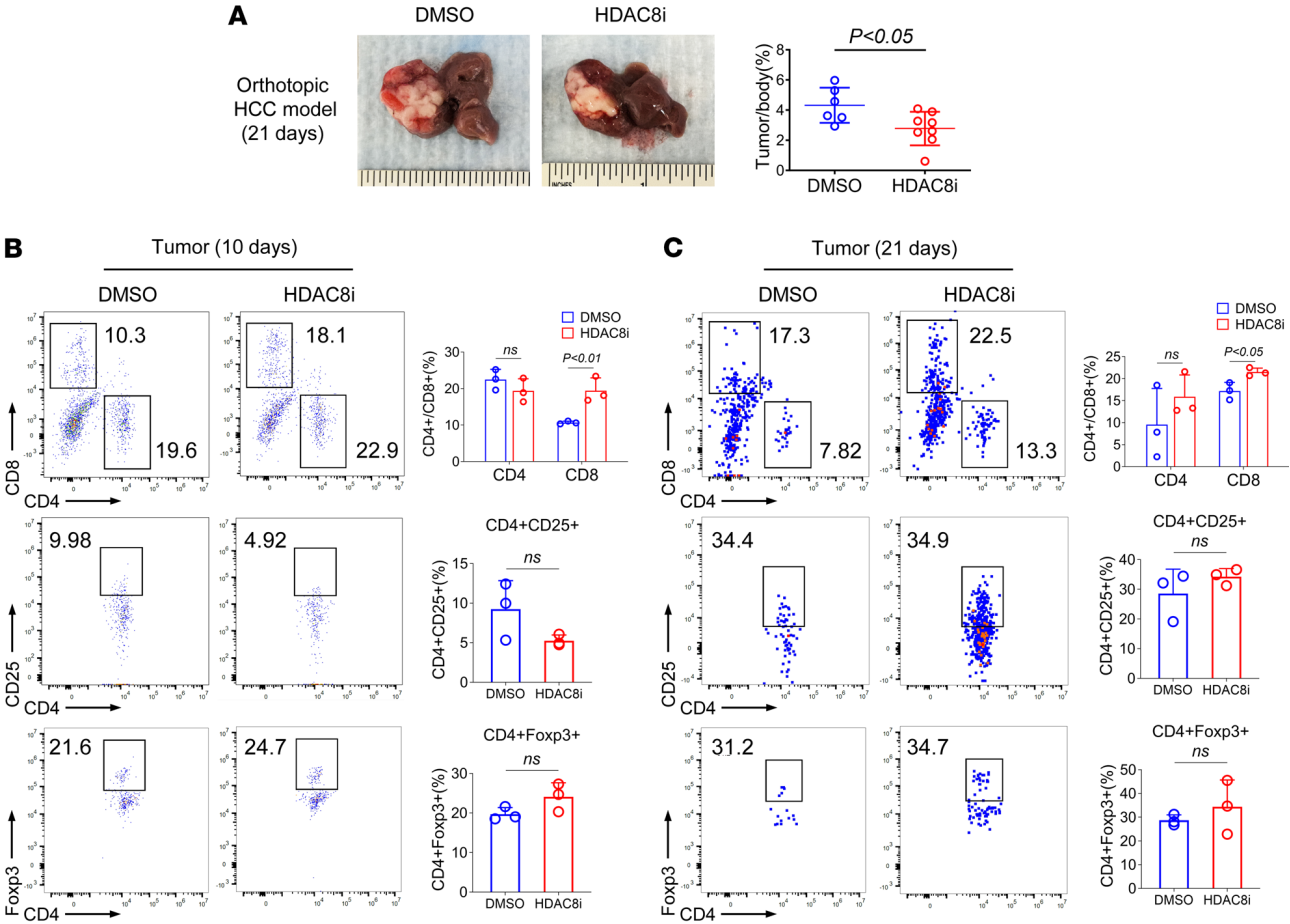
Inhibition of HDAC8 promotes antitumor immunity in an orthotopic HCC model. (**A**) Tumor/body weight ratios of DMSO (*n* = 6) and HDAC8i mice (*n* = 8) at 21 days. (**B** and **C**) Flow cytometry was used to analyze the expression of CD4^+^ T cells (gate: CD4^+^), CD8^+^ T cells (gate: CD8^+^), and Tregs (gate: CD4^+^Foxp3^+^, CD4^+^CD25^+^) in HCC tumors 10 and 21 days after implantation of H22 cells (*n* = 3). Assays were run in triplicate and repeated at least 3 times. The results of a representative experiment are shown. Data were expressed as the mean ± SD of 3 independent experiments. NS, not significant. Comparisons between 2 groups utilized a 2-tailed Student’s *t* test for normally distributed data.

**Figure 3:**
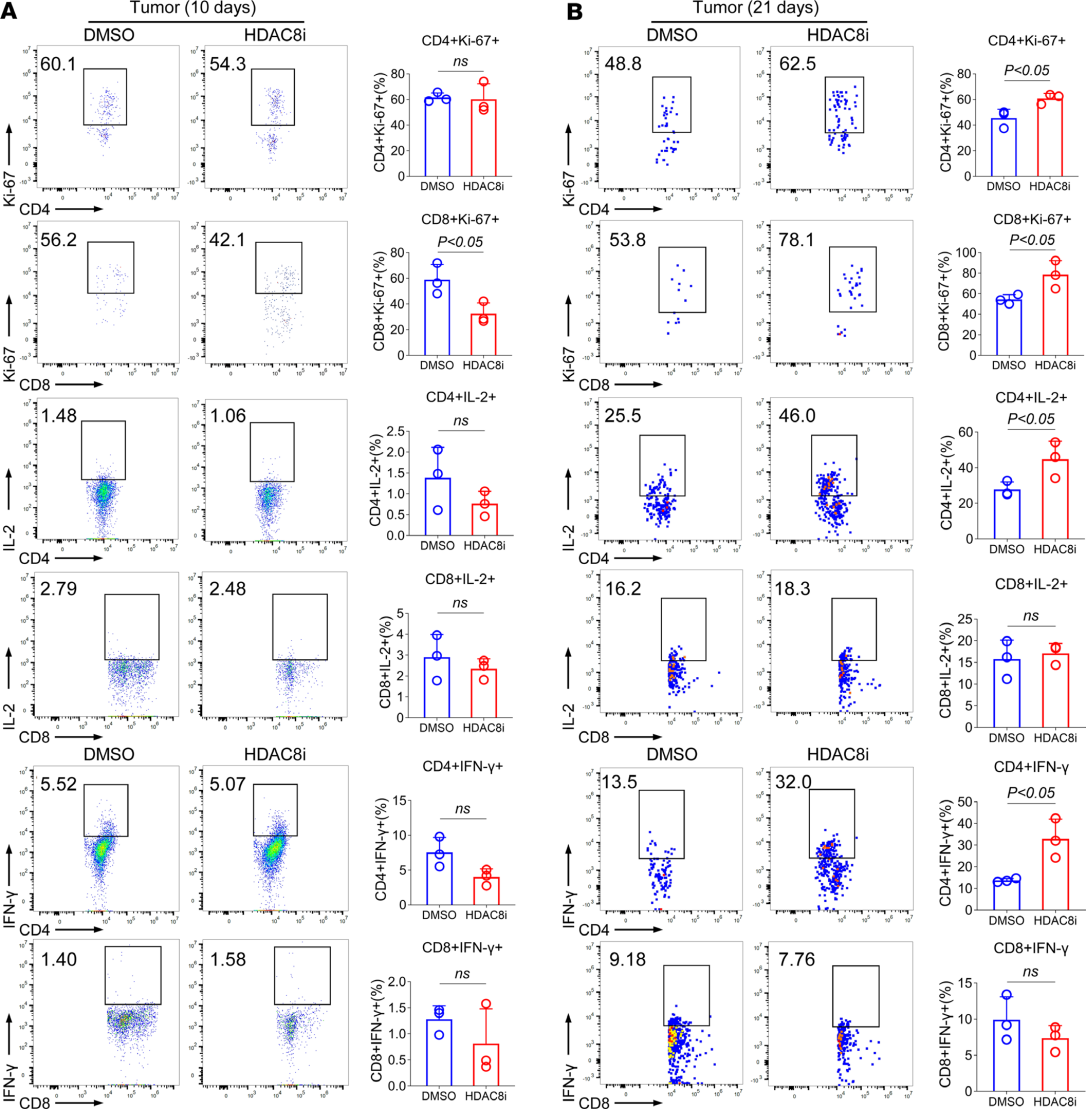
Inhibition of HDAC8 promotes T cell–mediated antitumor immunity. (**A**) Flow cytometry was used to analyze the expression of Ki-67, IL-2, and IFN-γ in HCC tumors 10 days after implantation of H22 cells (*n* = 3). (**B**) Flow cytometry was used to analyze the expression of Ki-67, IL-2, and IFN-γ in HCC tumors 21 days after implantation of H22 cells (*n* = 3). Assays were run in triplicate and repeated at least 3 times. The results of a representative experiment are shown. Data are expressed as the mean ± SD of 3 independent experiments., NS, not significant. Comparisons between 2 groups utilized a 2-tailed Student’s *t* test for normally distributed data.

To assess the effect of conditional deletion or inhibition of HDAC8 in an orthotopic third tumor model, we established an orthotopic hepatocellular carcinoma (HCC) tumor model in WT BALB/c mice, followed by treatment with the HDAC8i (OJI-1, 5 mg/kg/d), or by comparing HCC growth in CD4-Cre versus HDAC8^–/–^ mice. Inhibition of HDAC8 inhibited HCC proliferation (*P* < 0.05, Figure 2A). Flow cytometric analysis was performed 10 and 21 days after implantation of HCC cells, and inhibition of HDAC8 increased the proportion of CD8^+^ T cells in tumor tissue (*P* < 0.05, Figure 2, B and C), but did not affect CD4^+^CD25^+^ T cells or CD4^+^Foxp3^+^ T cells (Figure 2, B and C). Ki-67 is an important marker of cell proliferation and is widely expressed in proliferating cells. Its expression level in T cells is often used to evaluate the proliferative activity of T cells (26). As cytokines such as IL-2 and IFN-γ are crucial for T cell activation, proliferation, and function, and can disrupt the immunosuppressive functions of Tregs (27), their expression levels were assessed within tumors and lymphoid tissues. No statistically significant differences were observed in the expression of Ki-67, IL-2, and IFN-γ by T cells within the tumor 10 days after implantation of HCC cells (Figure 3A). However, inhibition of HDAC8 increased Ki-67 expression in CD4^+^ and CD8^+^ T cells 21 days after implantation of HCC cells (*P* < 0.05, Figure 3B), in conjunction with increased of expression of IL-2 and IFN-γ in CD4^+^ T cells (*P* < 0.05, Figure 3B); additional flow cytometry for infiltrating lymphocytes in the spleen and lymph nodes of HCC-bearing mice receiving HDAC8i are shown in Supplemental Figures 4–7.

In CD4-Cre and HDAC8^–/–^ mice, conditional deletion of HDAC8 inhibited HCC proliferation (*P* < 0.05, Supplemental Figure 8A). Flow cytometric analysis showed deletion of HDAC8 increased the proportion of CD4^+^ and CD8^+^ T cells in tumors and spleens (*P* < 0.05, Supplemental Figure 8B) but decreased corresponding Foxp3^+^ T cell proportions (*P* < 0.05, Supplemental Figure 8C). Tregs promote immune homeostasis and play a crucial role in anticancer immunity by inhibiting the activation and differentiation of CD4^+^ helper T cells and CD8^+^ cytotoxic T cells (28), as well as in some cases by inducing their differentiation into Tregs, thus promoting tumor immune evasion (29, 30). The expression of Ki-67 was upregulated in the infiltrating CD4^+^ and CD8^+^ T cells and Tregs in HCC (*P* < 0.05, Supplemental Figure 9). In addition, IL-2 and IFN-γ were upregulated in the infiltrating CD4^+^ and CD8^+^ T cells and Tregs in HCC (*P* < 0.05, Supplemental Figures 10 and 11). Hence, in this third tumor model, HDAC8 deletion and inhibition increased CD4^+^ and CD8^+^ T cell infiltration and effector cytokine production, and was associated with Treg dysfunction as indicated by increased IL-2 and IFN-γ expression by infiltrating Foxp3^+^ cells.

### Conditional deletion of HDAC8 impairs Treg suppressive functions.

**Figure 4:**
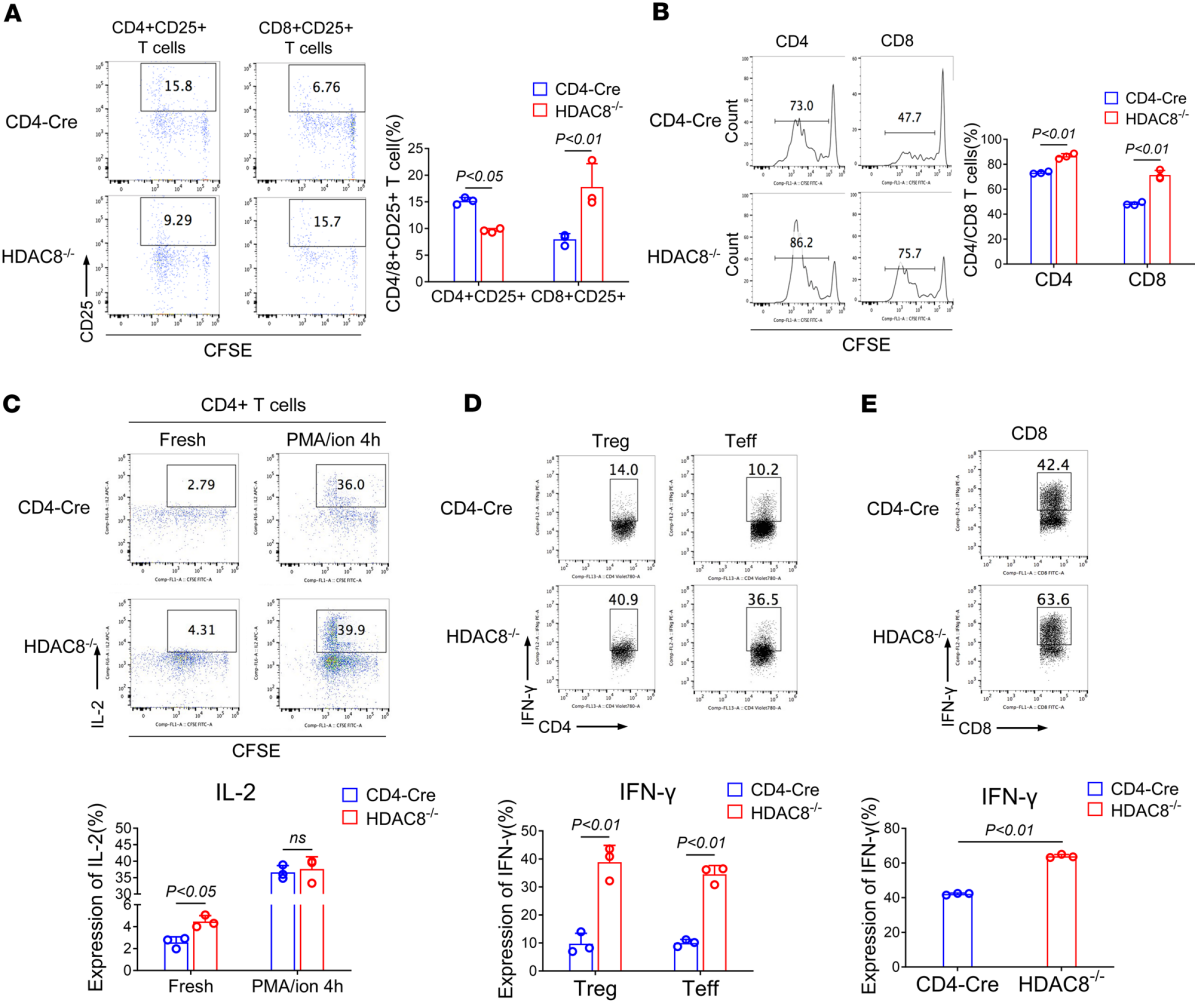
HDAC8 deletion enhances the proliferation and function of CD4^+^ and CD8^+^ T cells in vivo and in vitro. (**A–C**) CFSE-labeled splenocytes and lymph node cells (CD4-Cre and HDAC8^–/–^ mice, *n* = 3/group) were adoptively transferred into B6D2F1/J recipient mice (allogeneic parent→F1 model). CFSE fluorescence histograms (**B**) and flow scatter plot (**A** and **C**) of live CD4-Cre and HDAC8^–/–^ (donor) cells recovered 72 hours after adoptive transfer. The numbers above each figure denote division populations, with the undivided T cells forming the rightmost peak, and the T cells that divided several times residing in the leftmost peak. (**D**) Flow cytometric analysis of IFN-γ production using pooled (*n* = 3/group) Tregs and Teffs from lymph nodes and spleens of CD4-Cre versus HDAC8^–/–^ mice, following anti-CD3/anti-CD28 stimulation (24 hours) and PMA/ionomycin (4 hours). (**E**) Flow cytometric analysis of IFN-γ production using pooled (*n* = 3 /group) CD8^+^ T cells from lymph nodes and spleens of CD4-Cre and HDAC8^–/–^ mice, following anti-CD3/anti-CD28 stimulation (24 hours) and PMA/ionomycin (4 hopurs). Assays were run in triplicate and repeated at least 3 times. The results of a representative experiment are shown. Data are expressed as the mean ± SD of 3 independent experiments. NS, not significant. Comparisons between 2 groups utilized a 2-tailed Student’s *t* test for normally distributed data.

Next, in a parent→F1 model (31), lymphocytes from the spleen and lymph nodes of CD4-Cre and HDAC8^–/–^ C57BL/6 mice were labeled with CFSE and injected into B6D2F1/J mice to analyze the effect of HDAC8 deletion on the activation, proliferation, and cytokine production of alloreactive T cells in vivo. Compared with CD4-Cre controls, HDAC8 deletion impaired the proliferation of Tregs (*P* < 0.05) but enhanced the proliferation of CD8^+^CD25^+^ conventional T cells (*n* = 3, *P* < 0.01) (Figure 4A). The ability of Tregs to inhibit CD4^+^ (*P* < 0.01) and CD8^+^ T cell proliferation (*P* < 0.01) in vivo was also impaired by HDAC8 deletion (Figure 4B) and HDAC8^–/–^ CD4^+^ T cells produced more IL-2 (*P* < 0.05) (Figure 4C). HDAC8 deletion also increased IFN-γ expression by Tregs (*P* < 0.01), effector T cells (Teffs) (*P* < 0.01), and CD8^+^ T cells (*P* < 0.01) (Figure 4, D and E). These findings, using an orthogonal approach to the tumor studies, again show that HDAC8 deletion promotes T cell proliferation and cytokine production while disrupting Treg homeostasis, as reflected by their ability to now produce IL-2 and IFN-γ cytokines.

**Figure 5:**
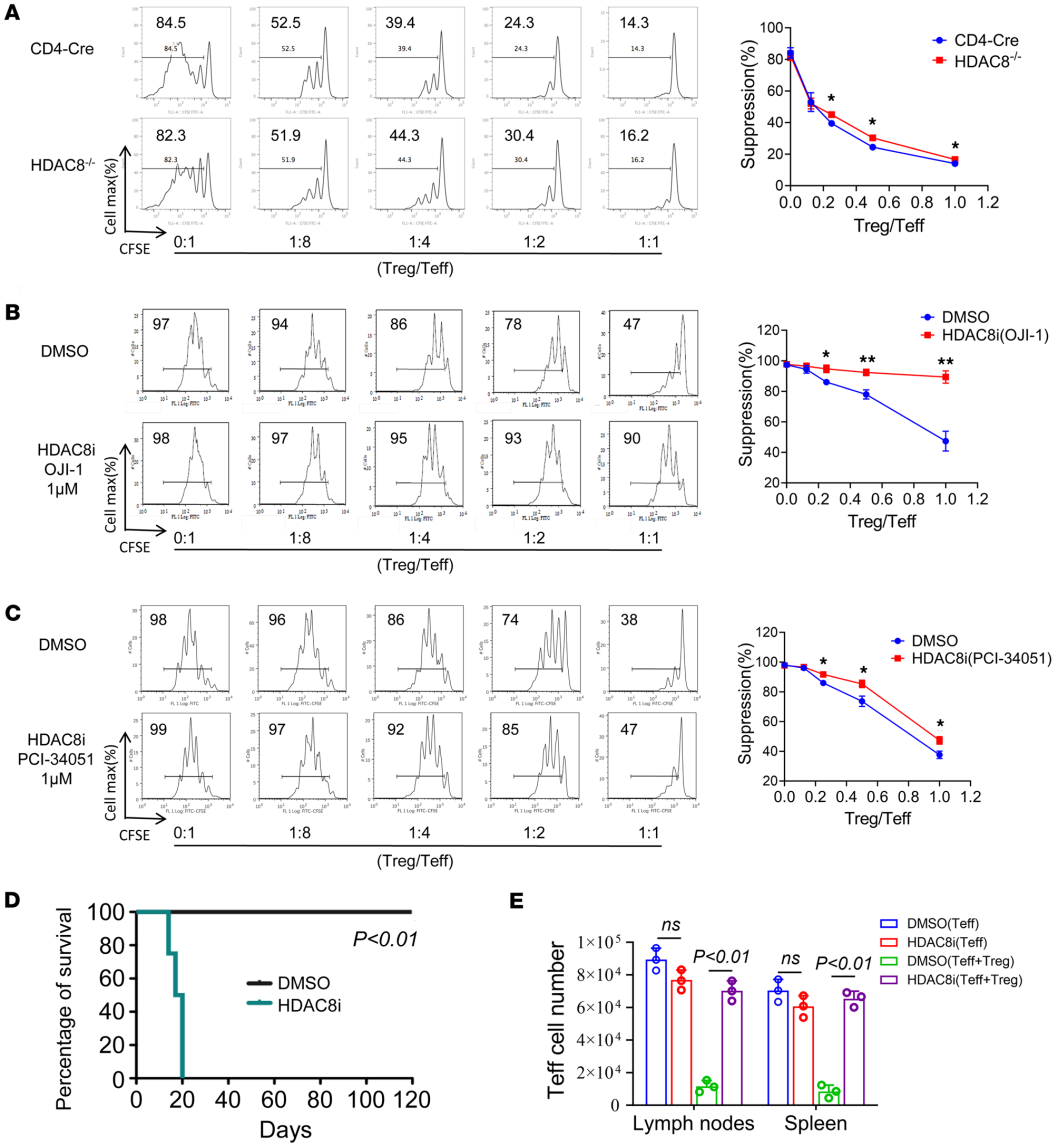
Conditional deletion or inhibition of HDAC8 impaired Treg suppressive function in vivo and in vitro. (**A**) Treg suppression assays using pooled Tregs and Teffs (*n* = 3/group) from lymph nodes and spleens of mice (CD4-Cre and HDAC8^–/–^), as indicated. (**B** and **C**) Treg suppression assays using pooled Tregs and Teffs (3 mice/ group) from lymph nodes and spleens of treated mice (DMSO and HDAC8i, *n* = 3/group), as indicated. (**D**) Immunodeficient Rag1^–/–^ C57BL/6 murine recipients of BALB/c cardiac allografts were injected with conventional HDAC8^–/–^ T effector cells (Teffs) plus Tregs (2:1 ratio), and treated with HDAC8i (OJI-1) 5 mg/kg/d for 14 days via Alzet pumps (*n* = 4/group). (**E**) Immunodeficient Rag1^–/–^ mice were injected with conventional Teffs with and without Tregs and treated with HDAC8i (OJI-1, 5 mg/kg/d) for 7 days (*n* = 3/group). Assays were run in triplicate and repeated at least 3 times. The results of a representative experiment are shown. Data expressed as the mean ± SD of 3 independent experiments. *P < 0.05, **P < 0.01. NS, not significant. Comparisons between 2 groups utilized a 2-tailed Student’s *t* test for normally distributed data.

Focusing directly on the effects of HDAC8 targeting on Tregs, in vitro assays showed that the suppressive function of Tregs was only very modestly impaired in HDAC8^–/–^ mice (Figure 5A) and by HDAC8i therapy (Figure 5, B and C), whereas this effect was more obvious from in vivo assays. Thus, in cardiac allograft recipients (BALB/c→Rag1^–/–^ C57BL/6) adoptively transferred with HDAC8^–/–^ conventional T cells and WT Tregs (2:1 ratio), treatment with HDAC8i (OJI-1, 5 mg/kg/d) but not DMSO control for 14 days after transplantation blocked Treg-induced long-term allograft survival (*P* < 0.01, Figure 5D). Similarly, in homeostatic proliferation assays, when immunodeficient Rag1^–/–^ mice were injected i.p. with conventional Teffs with and without Tregs and treated with HDAC8i (OJI-1, 5 mg/kg/day) for 7 days, HDAC8i use impaired the immunosuppressive function of Tregs and restored Teff cell numbers (*P* < 0.01, Figure 5E). In the in vitro Treg suppression assay, deletion of HDAC8 in Tregs impaired the immunosuppressive function of Tregs, while deletion of HDAC8 in Teffs showed no statistically significant difference (Supplemental Figure 12). Thus, conditional deletion or inhibition of HDAC8 impaired the immunosuppressive functions of Tregs.

### Conditional deletion and inhibition of HDAC8 decreased Foxp3 expression.

**Figure 6:**
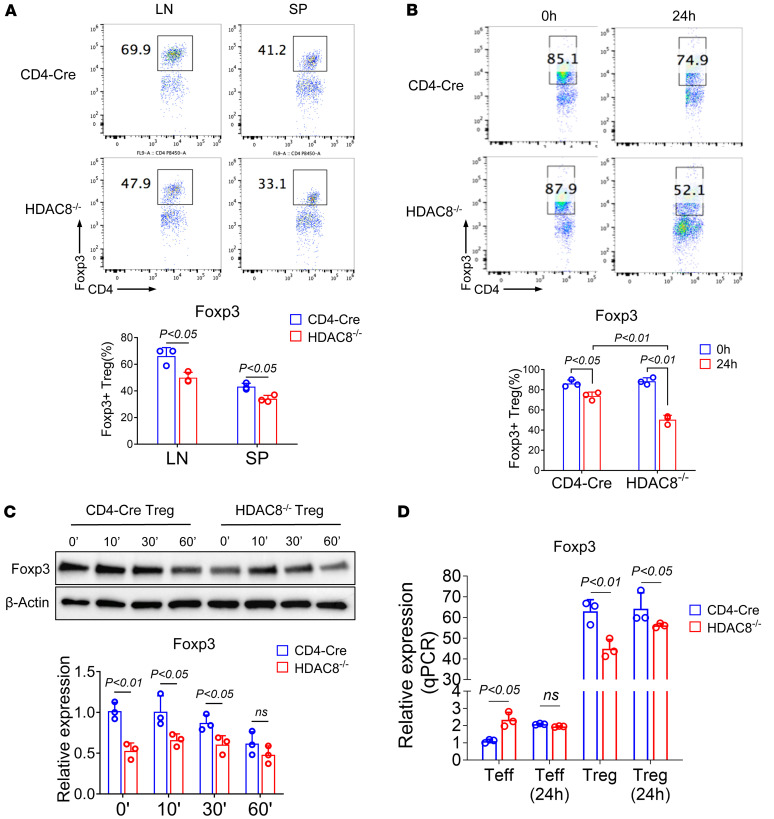
HDAC8 deletion decreased Foxp3 expression in vivo and in vitro. (**A**) The stability of Foxp3 in lymph nodes and spleen was analyzed by flow cytometry 10 days after CD4-Cre and HDAC8^–/–^ Tregs were injected into immunodeficient Rag1^–/–^ mice (*n* = 3/group). (**B**) Flow cytometry was used to analyze Foxp3 within pooled (*n* = 3/group) Tregs from lymph nodes and spleens of CD4-Cre and HDAC8^–/–^ mice, as indicated (anti-CD3/anti-CD28 stimulation for 24 hours). (**C**) Western blot analysis of Foxp3 in CD4-Cre and HDAC8^–/–^ Tregs stimulated with anti-CD3/anti-CD28 beads (1:1) for indicated times. (**D**) qRT-PCR analysis of Foxp3 mRNA in CD4-Cre and HDAC8^–/–^ Teffs and Tregs were stimulated with anti-CD3/anti-CD28 beads (1:1) for indicated times. Assays were run in triplicate and repeated at least 3 times. The results of 1 representative experiment are shown. Data are expressed as the mean ± SD of 3 independent experiments. NS, not significant. Comparisons between 2 groups utilized a 2-tailed Student’s *t* test for normally distributed data. For multiple comparisons, we used 1-way ANOVA followed by Tukey’s post hoc test.

Multiple transcription factors regulate the stability, differentiation, and suppressive function of Foxp3^+^ Tregs (9). Since our data suggested that HDAC8 deletion downregulated Foxp3 expression and impaired the suppressive function of Tregs, we tested whether HDAC8 regulates Foxp3 stability. CD4-Cre and HDAC8^–/–^ Tregs were injected into immunodeficient Rag1^–/–^ mice, and Foxp3 expression was assessed 10 days later, and the proportion of Foxp3^+^ Tregs was downregulated in HDAC8^–/–^ compared with CD4-Cre controls (*P* < 0.05, Figure 6A). In parallel, Tregs from CD4-Cre and HDAC8^–/–^ mice were analyzed after 24 hours of stimulation with anti-CD3/anti-CD28. Foxp3 expression in both groups decreased over time, but HDAC8^–/–^ Tregs showed lower expression levels at 24 hours (*P* < 0.01, Figure 6B), whereas HDAC8 deletion did not alter induced-Treg stability or function (Supplemental Figure 13A). Western blot results showed that deletion of HDAC8 downregulated Foxp3 expression in the presence or absence of anti-CD3/anti-CD28 stimulation compared with the control group (Figure 6C), consistent with the results of qPCR experiments (Figure 6D). Thus, HDAC8 deletion leads to a loss of Foxp3 expression. In addition, Foxp3 is a Treg-specific transcription factor, and its methylation level plays an important role in regulating the development and function of Tregs (32). To assess the effect of HDAC8 depletion on the methylation of Foxp3 DNA, we analyzed the Foxp3 CNS2 region by EpiTYPER DNA methylation. In contrast to CD4-Cre, deletion of HDAC8 did not alter the methylation level of Foxp3 CNS2 in Tregs (Supplemental Figure 13B and Supplemental Table 1). Thus, HDAC8 targeting suppresses Foxp3 expression in Tregs independently of changes in Foxp3 methylation.

### HDAC8 deletion enhances substrate acetylation and expression.

**Figure 7:**
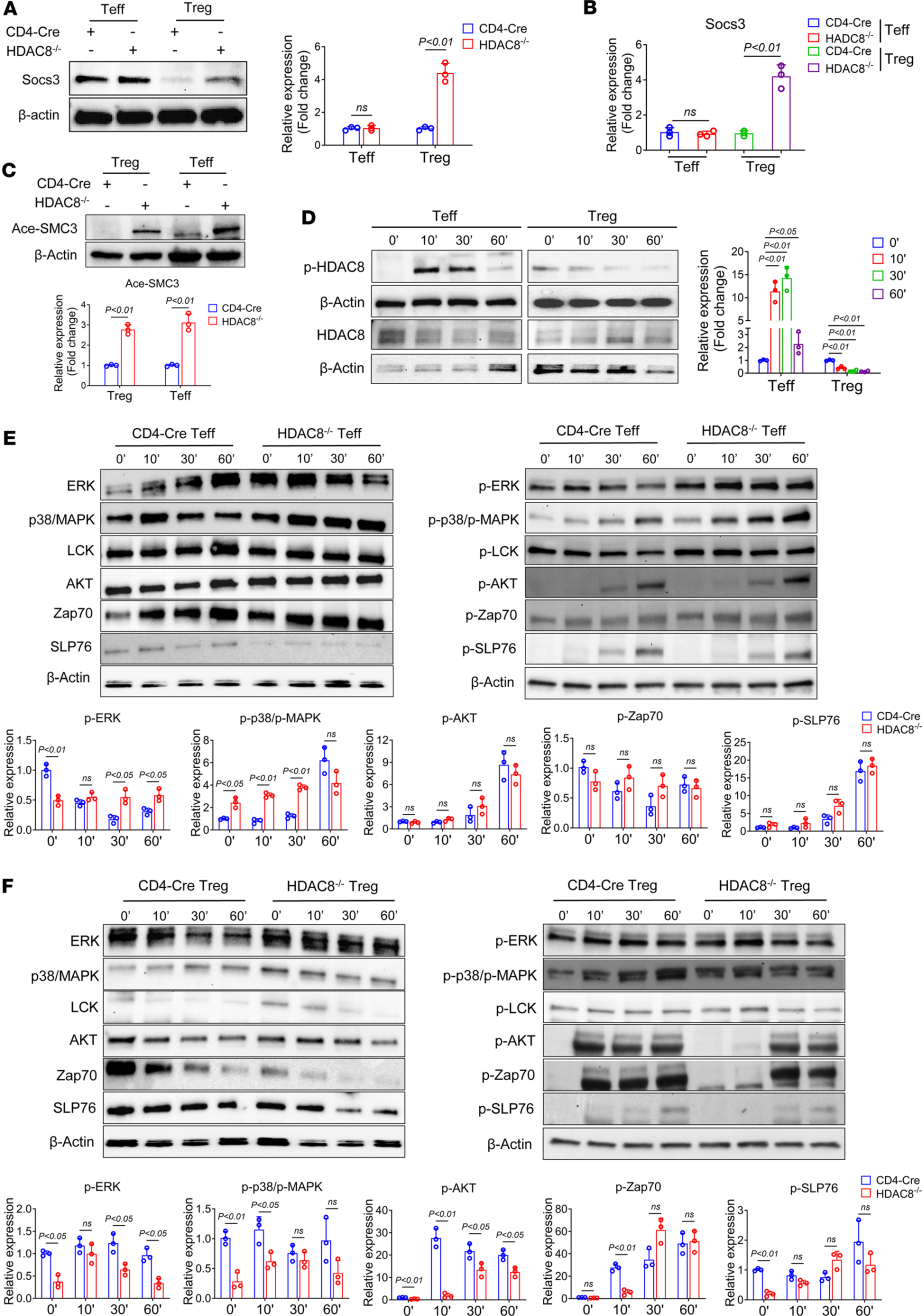
HDAC8 deletion enhances substrate acetylation and promotes its expression. (**A**) Western blot analysis was used to detect the expression of Socs3 in Teffs and Tregs. (**B**) qRT-PCR analysis was used to detect the expression of Socs3 in Teffs and Tregs. (**C**) Western blot analysis was used to detect the expression of acetyl-SMC3 (Ace-SMC3) in Teffs and Tregs. (**D**) Western blot analysis was used to detect the expression of p-HDAC8 in Teffs and Tregs stimulated with anti-CD3/anti-CD28 beads (1:1) for indicated times. (**E** and **F**) Western blot analysis of CD4-Cre Teffs and Tregs and HDAC8^–/–^ Teffs and Tregs were stimulated with anti-CD3/anti-CD28 beads (1:1) for indicated times. Assays were run in triplicate and repeated at least 3 times. The results of a representative experiment are shown. Data are expressed as the mean ± SD of 3 independent experiments. NS, not significant. Comparisons between 2 groups utilized a 2-tailed Student’s *t* test for normally distributed data. For multiple comparisons, we used 1-way ANOVA followed by Tukey’s post hoc test.

In genomic studies, CD4-Cre and HDAC8^–/–^ Tregs/Teffs were compared by RNA-seq; Supplemental Table 2 shows differentially expressed genes in Tregs and Supplemental Table 3 shows differentially expressed genes in Teffs after deletion of HDAC8. The volcano plot of differentially expressed genes (Supplemental Figure 14) showed upregulation of Socs3 in HDAC8^–/–^ Tregs and Teffs. Immunoblotting and qPCR confirmed this finding, with markedly increased Socs3 expression in HDAC8^–/–^ Tregs compared with CD4-Cre Tregs (Figure 7, A and B), and no statistically significant difference observed in Teffs (Figure 7, A and B). Cotransfection experiments also showed that overexpression of Socs3 inhibited HDAC8-mediated regulation of Foxp3 expression (Supplemental Figure 15). Socs3 expression is negatively correlated with Treg function, including reduced Treg proliferation and lower expression of Foxp3 and CTLA-4 (33). Thus, upregulating Socs3 may contribute to decreased Treg suppressive function.

HDAC8 deletion also enhanced substrate acetylation levels, promoting substrate expression. For example, cohesin, a protein complex involved in chromosome organization, is frequently mutated or abnormally expressed in cancer. The diverse functions of cohesin in cell division and gene expression make it a promising therapeutic target (16). The deacetylation of SMC3 is crucial for cohesin recycling during the cell cycle (34). HDAC8 mutation or deletion leads to the accumulation of acetyl-SMC3, reduced chromatin-bound cohesin, and widespread cohesin-mediated transcriptional dysregulation (35). Immunoblotting demonstrated upregulation of acetyl-SMC3 in HDAC8^–/–^ Tregs and Teffs (Figure 7C). Therefore, HDAC8 deletion in Tregs promotes acetyl-SMC3 expression, and likely delays cell cycle progression, inhibits proliferation, and induces apoptosis. Furthermore, phosphorylated HDAC8 (p-HDAC8) expression was upregulated in Teffs after anti-CD3/anti-CD28 stimulation, with the most pronounced difference observed after 10 minutes (Figure 7D), but was downregulated in Tregs (Figure 7D). HDAC8 is phosphorylated within the conserved deacetylase domain at serine 39, reducing its catalytic activity and deacetylation capacity (36). Thus, HDAC8 catalytic activity is inhibited in activated Teffs, while the opposite occurs in Tregs. Immunoblotting showed that p-ERK and p-p38/MAPK levels were upregulated in HDAC8^–/–^ Teffs compared with CD4-Cre cells after stimulation (Figure 7E); however, p-ERK and p-p38/MAPK levels were downregulated in HDAC8^–/–^ Tregs (Figure 7F). Conversely, p-AKT and p-ZAP70 levels were downregulated in HDAC8^–/–^ Tregs after 10 minutes of stimulation (Figure 7F), while no changes were observed in HDAC8^–/–^ Teffs (Figure 7E). Subsequently, p38 and ERK inhibitors were administered following anti-CD3/anti-CD28 stimulation to suppress the activation of p38 and ERK. Immunoblotting results showed that inhibition of p38 and ERK reduced p-HDAC8 expression in Teffs, while no changes were observed in Tregs (Supplemental Figure 16). qRT-PCR results showed that inhibition of p38 and ERK reduced the expression of IL-2, IFN-γ, and granzyme B in Teffs and Tregs, and interestingly, the difference in IL-2 expression was particularly striking in Teffs, implying an important role of HDAC8-mediated ERK/p38 signaling in Teffs (Supplemental Figure 17). Thus, it is possible HDAC8 deletion is modulating Teff/Treg function via differential effects on p38 and ERK signaling pathways.

**Figure 8:**
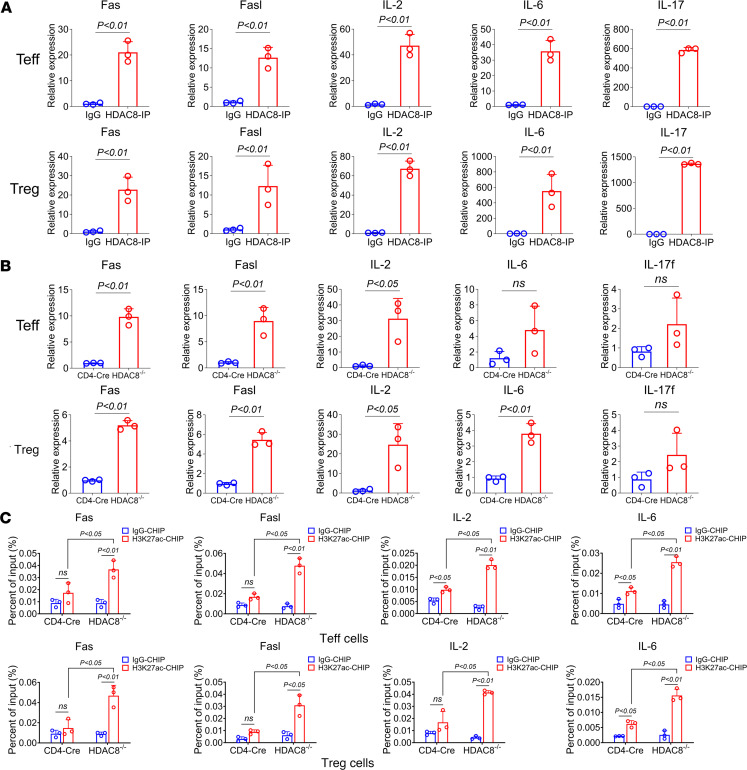
ChIP-PCR and qRT-PCR analyses using pooled Tregs and Teffs from lymph nodes and spleens of CD4-Cre and HDAC8^–/–^ mice. (**A**) ChIP-PCR analysis of HDAC8 IP in Tregs and Teffs. (**B**) qRT-PCR analysis of Fas, Fasl, IL-2, IL-6, and IL-17f in Tregs and Teffs from lymph nodes and spleens of CD4-Cre and HDAC8^–/–^ mice. (**C**) ChIP-PCR analysis of H3K27ac IP in Tregs and Teffs from lymph nodes and spleens of CD4-Cre and HDAC8^–/–^ mice. Assays were run in triplicate and repeated at least 3 times. The results of a representative experiment are shown. Data are expressed as the mean ± SD of 3 independent experiments. NS, not significant. Comparisons between 2 groups utilized a 2-tailed Student’s *t* test for normally distributed data. For multiple comparisons, we used 1-way ANOVA followed by Tukey’s post hoc test.

To further analyze the regulatory mechanism by which HDAC8 deletion affects substrate expression levels, HDAC8 ChIP-seq analysis was performed on mouse Teffs and Tregs. Gene Ontology (GO) and Kyoto Encyclopedia of Genes and Genomes (KEGG) analyses revealed that differentially expressed genes in Teffs were enriched in the MAPK signaling pathway, apoptosis, and cell cycle pathways (Supplemental Figure 18), while in Tregs, these genes were enriched in the MAPK, cAMP, and chemokine signaling pathways (Supplemental Figure 18). Previous analyses identified that HDAC8 deletion upregulates the expression of IL-2, IFN-γ, and other cytokines. The HDAC8 ChIP-seq analysis showed that Fas, Fasl, IL-2, IL-6, and IL-17 could be pulled down by HDAC8 (Supplemental Tables 4–9). Further ChIP-PCR assays confirmed HDAC8 binding to the promoter regions of Fas, Fasl, IL-2, IL-6, and IL-17 (Figure 8A). Additionally, qPCR results indicated that the expression of Fas, Fasl, and IL-2 was upregulated after HDAC8 deletion in Teffs (Figure 8B), and the expression of Fas, Fasl, IL-2, and IL-6 was upregulated in Tregs (Figure 8B). HDAC8 selectively targets H3K27ac, typically present at active enhancers (37, 38). H3K27ac ChIP-PCR results demonstrated that HDAC8 deletion enhanced the acetylation level of H3K27 and promoted the expression of Fas, Fasl, IL-2, and IL-6 (Figure 8C). Therefore, HDAC8 deletion enhances the acetylation level of its substrate, boosts T cell–mediated antitumor immunity by increasing the expression of Fas, Fasl, IL-2, and IL-6, and further impairs the immunosuppressive function of Tregs.

In addition, mass spectrometry was used to analyze the protein binding levels of HDAC8 within Teffs and Tregs. Volcanic dot plots and heatmaps showed that Hipk2, Lkzf5, Nfat5, Crtc3, and Znf639 proteins coprecipitated with HDAC8 in Teffs with the pull-down of HDAC8 (Supplemental Figure 19 and Supplemental Table 10). In Tregs, Hipk2, Nfat5, Pkn1, Fastkd3, Rfc2, Gstt3, Rif1, and Znf639 coprecipitated with HDAC8 (Supplemental Figure 19 and Supplemental Table 11). Interestingly, we found through protein interaction strength analysis that in Teffs, proteins coprecipitated with HDAC8 were enriched in DNA metabolic process, histone deacetylation and modification, and positive regulation of histone methylation (Supplemental Figure 20A). In Tregs, proteins coprecipitated with HDAC8 were enriched in peptidyl–amino acid modification, and phosphorylation (Supplemental Figure 20B). Meanwhile, KEGG enrichment analysis showed that the proteins coprecipitated with HDAC8 were enriched in cell cycle, ATP-dependent chromatin remodeling, and endocytosis (Supplemental Figure 20B). HDAC8, as a classical histone deacetylase, affects epigenetic gene silencing and cancer progression. In this mass spectrometry analysis, we provide the potential target proteins that interact with HDAC8, although whether HDAC8 may affect these proteins through epigenetic or non-epigenetic modifications remains to determined.

## Discussion

Epigenetics involves changes in gene expression without changes in DNA sequence, and understanding the epigenome of infiltrating immune cells in the tumor microenvironment may reshape transcriptional networks that define tumor cell characteristics. Epigenetic therapy, besides altering the intrinsic phenotype of tumor cells, can counteract immune escape mechanisms associated with immune checkpoint blockade and combat primary or acquired drug resistance (39). As a member of the HDAC family, HDAC8 affects epigenetic gene silencing and cancer progression (40), and inhibition of HDAC8 is a benchmark therapy for malignant tumors (14, 16). In this study, conditional HDAC8 targeting uncovered important roles for HDAC8 in immune cells, including differential effects on conventional T cells and Foxp3^+^ Tregs.

HDAC8 has been implicated in immune evasion. Inhibition of HDAC8 can shift glioma-associated microglia/macrophages to a proinflammatory/antitumor phenotype and increase the infiltration and cytotoxic activity of NK cells (21). Additionally, an HDAC8-regulated enhancer program may affect T cell responses to tumors and diminish the response to PD-L1 blockade in HCC (20, 41). Our results suggest that conditional deletion and inhibition of HDAC8 can promote antitumor immunity through effects on adaptive immune responses. Deletion or inhibition of HDAC8 suppressed tumor proliferation in murine syngeneic tumor models, whereas no suppressive effect was observed in immunodeficient mice, indicating that HDAC8 did not directly regulate tumor cell proliferation but instead modulated adaptive immunity within the tumor microenvironment.

Prior work on HDAC8 and malignant tumor progression have not addressed its potential involvement in the actions of Tregs and Teffs (42, 43). Tregs, an immunosuppressive subset of CD4^+^ T cells characterized by Foxp3 expression, play a central role in forming an immune microenvironment conducive to tumorigenesis (44, 45). Tregs also exert inhibitory effects on Teffs, NK cells, monocytes/macrophages, antigen-presenting cells, and other immune cells, affecting cell apoptosis and inhibiting effector cell activation/proliferation through various mechanisms (41, 46). Flow cytometry results demonstrated that HDAC8 deletion enhanced tumor infiltration by CD4^+^ and CD8^+^ T cells while decreasing Treg infiltration. Additionally, HDAC8 deletion upregulated expression of Ki-67, IL-2, and IFN-γ in CD4^+^ and CD8^+^ T cells. Thus, our study identifies HDAC8 as a key regulator of immune responses in both conventional T cells and Tregs, in that targeting of HDAC8 increases T cell activation in conventional T cells and decreases the suppressive actions of Foxp3^+^ Tregs. However, the relative effects on these populations are unclear, given CD4-Cre can delete the floxed HDAC8 gene in conventional CD4^+^ and CD8^+^ T cells as well as in Tregs. Also remaining to be explored is the extent to which enhanced T cell responses and antitumor immunity arise from decreased Treg suppression, increased overall T cell activation, enhancement of tumor-specific responses, or a combination of these mechanisms.

In the current study, the suppressive functions of Foxp3^+^ HDAC8^–/–^ Tregs were impaired in vitro and in vivo, leading to increased antitumor immunity and allograft rejection, in conjunction with aberrant production of cytokines such as IL-2, IFN-γ, and IL-6. These are features of grossly abnormal Tregs that are undergoing loss of normal function and/or becoming so-called exTregs or apoptotic cells (47). Comparable data were seen using HDAC8is (OJ-1 and PCI34051) when tested in WT mice. RNA-seq and immunoblot analysis showed that HDAC8 deletion upregulated Socs3 expression. Socs3 can negatively regulate Foxp3 expression through the phosphorylation of STAT3 (48), and its overexpression can reduce Treg proliferation, Foxp3 and CTLA-4 expression, and inhibitory function. Thus, upregulation of Socs may serve as an effective therapeutic approach to inhibit Tregs (33). Moreover, HDAC8 deletion promoted MAPK/ERK signaling in Teffs, but not in Tregs, indicating distinct biological functions of HDAC8 within these cell types. MAPK/ERK signaling enhances Teff function. Interestingly, HDAC8 deletion in Tregs inhibited AKT and ZAP70 phosphorylation, while no abnormalities were observed in Teffs. Reduced phosphorylation of AKT and ZAP70 downstream of the CD3/CD28 signaling pathway leads to reduced Tregs and an enhanced adaptive immune response (49). Therefore, HDAC8 deletion likely enhances the antitumor immune response by promoting Teff function through MAPK/ERK signaling and inhibiting Treg function by reducing AKT and ZAP70 phosphorylation.

As a structural component of the cohesin complex, the acetylation of SMC3 controls circular cohesin to regulate DNA cyclization, thereby increasing the potential for enhancer-promoter interactions in 3D-chromatin structures and enhancing gene transcription (50). Our results showed that the deletion of HDAC8 promotes SMC3 acetylation. Therefore, it is hypothesized that inhibition or knockdown of HDAC8 in Tregs may alter the 3D-chromatin structure, promoting cytokine gene transcription and impairing Treg function. Additionally, ChIP-PCR results confirmed that HDAC8 deletion enhanced H3K27 acetylation levels and promoted the expression of Fas, Fasl, IL-2, and IL-6, enhancing T cell–mediated antitumor immunity and impairing the immunosuppressive function of Tregs. Our study elucidates the mechanism by which HDAC8 regulates the biological function of Tregs and promotes tumor immune escape. Data on the effects of conditional targeting of HDAC8 in T cells and the impact of selective HDAC8i on T cell–dependent immune responses suggest potential strategies for more effective cancer immunotherapy.

## Methods

### Sex as a biological variable.

Our study examined male and female animals, and similar findings were found using both sexes.

### Mice.

WT BALB/c, WT C57BL/6, Rag1^–/–^ C57BL/6, CD90.1/B6, and B6D2F1/J mice were obtained from The Jackson Laboratory and used at 6–8 weeks of age unless specified otherwise. The targeted CD4-Cre allele was generated by replacing the lck proximal promoter with the mCD4 promoter/enhancer/silencer (51). CD4-Cre mice served as WT controls. For the production of HDAC8^fl/fl^ mice for conditional deletion, frozen sperm from an HDAC8^fl/fl^ mouse was obtained from the European Conditional Mouse Mutagenesis Program, followed by in vitro fertilization, screening and breeding of chimeric mice, crossing of mice showing germline transmission with “Flpase mice” to remove the targeting construct, and interbreeding to obtain homozygous HDAC8^fl/fl^ mice. An allele map is shown in Supplemental Figure 21A. HDAC8^fl/fl^ mice were mated with CD4-Cre mice to delete HDAC8 in all CD4^+^ T cells (C57BL/6NTac-HDAC8^tm1a(EUCOMM)Wtsi^/WtsiBiat) and the efficacy of HDAC8 knockout was monitored by Western blotting (Supplemental Figure 21B).

### Datasets and preprocessing.

To analyze the expression level of HDAC8 in human solid tumors, we download the lung adenocarcinoma (LUAD) RNA-seq transcriptome datasets (including 59 normal tissues and 541 samples of LUAD) in TCGA database (https://portal.gdc.cancer.gov/). TCGA cohort was analyzed using the Perl programming language (version Strawberry-Perl-5.30.0; https://www.perl.org) (52). Based on the HDAC family of genes we obtained, we analyzed differences in the expression of HDAC family genes in LUAD and normal tissues using the “limma” and “reshape2” packets. We entered HDAC8 into the “Gene_DE” module of TIMER2 (https://db3.cistrome.org/browser/) and observed the expression difference in HDAC8 between tumor and adjacent normal tissues (human) for the different tumors or specific tumor subtypes of TCGA project. We used the “Immune-Gene” module of the TIMER2 web server to investigate the association between HDAC8 expression and immune infiltrates in all TCGA tumors. Tregs were selected as immune cells. Immune infiltration was estimated using the CIBERSORT, CIBERSORT-ABS, and QUANTISEQ algorithms. The purity-adjusted Spearman’s rank correlation test was used to calculate the *P* values and partial correlation (cor) values obtained via the purity-adjusted Spearman’s rank correlation test. A heatmap and a scatter plot were used to represent the data.

### Cell lines and cell culture.

Murine HCC (H22) cells were obtained from Wuhan Procell Life Science & Technology, murine lung adenocarcinoma (TC-1) cells (23) were provided by Yvonne Paterson (University of Pennsylvania), and the murine AE-17.ova mesothelioma cell line (25) was provided by Delia Nelson (University of Western Australia, Perth, Australia). Cell lines were cultured in RPMI-1640 medium, supplemented with 10% FBS and 1% penicillin/streptomycin (Invitrogen), at 37°C in a water-saturated atmosphere with 5% CO_2_ and 95% air.

### Western blots.

Proteins separated by gel electrophoresis were transferred to PVDF membranes and incubated with primary Abs for 12 hours at 4°C, followed by secondary Abs for 2 hours at room temperature. Immunoreactive bands were developed using an enhanced chemiluminescence detection kit (Thermo Fisher Scientific). All experiments were performed in triplicate. Abs used for Western blotting are listed in Supplemental Table 12.

### RNA-seq and real-time qPCR.

RNA was isolated using RNeasy kits (QIAGEN) and analyzed using a NanoDrop ND-1000 and a Nanochip 2100 Bioanalyzer (Agilent Technologies). Library preparation, RNA-seq, genome mapping, and analysis were performed by Novogene on the Illumina platform (PE150). Differential expression was analyzed using the DESeq2 R package, with statistical significance set at an FDR-adjusted *P* value (*P*_adj_) of less than 0.05, followed by GO, KEGG, and Reactome database enrichment analyses. *Z* scores were calculated for 3 replicates/condition using the formula *z* = (*x* – *μ*)/*σ*, where *x* represents fragments per kilobase million value, *μ* the mean per row (gene), and *σ* the standard deviation (SD) per row (gene). Heatmaps were generated using the Morpheus app on the Broad Institute website (https://software.broadinstitute.org/morpheus/). Expression of individual genes was verified by qPCR. RNA was reverse transcribed to cDNA (Applied Biosystems), and qPCR performed using TaqMan primer and probe sets. Data were normalized to endogenous 18S rRNA and relative expression determined using 2^–ΔCt^. Primers used for real-time qPCR are listed in Supplemental Table 13.

### Parent→F1 proliferation assays.

Spleen and lymph nodes from CD4-Cre and HDAC8^–/–^ mice were harvested, and single-cell suspensions were prepared and labeled with CFSE (Molecular Probes), as previously described (53). Fluorochrome-labeled mAbs against CD4, CD8, CD25, and IL-2 were purchased from PharMingen. A total of 3 × 10^7^ to 5 × 10^7^ CFSE-labeled splenocytes and lymph node cells from CD4-Cre and HDAC8^–/–^ mice were injected intravenously into B6D2F1/J mice in a total volume of 0.25 mL sterile PBS. Recipients were sacrificed at 72 hours, and spleen and lymph nodes (superficial inguinal, axillary, lateral axillary, cervical, and paraaortic) were harvested.

### Treg suppression assays.

For in vitro studies, 5 × 10^4^ CD4^+^CD25^–^YFP^–^ T cells and CD4^+^CD25^+^YFP^+^ Tregs from CD4-Cre and HDAC8^–/–^ mice (treated with DMSO or HDAC8i) were isolated using a CD4^+^CD25^+^ Treg isolation kit (Miltenyi Biotec, 130-091-041) or by cell sorting, and then added to 96-well plates. Equal numbers of CFSE-labeled CD4^+^CD25^–^ T cells and γ-irradiated antigen-presenting cells, isolated using a CD90.1 kit (Miltenyi Biotec, 130-049-101), along with anti-CD3 mAb (1 μg/mL), were cultured for 72 hours. The proliferation of Teffs was determined after 72 hours by flow cytometry, analyzing CellTrace Violet (Thermo Fisher Scientific) dilution. In vivo Treg suppression assays involved injecting 1 × 10^6^ CD4^+^CD25^–^Thy1.1^+^ cells and 0.5 × 10^6^ Tregs intravenously into Rag1^–/–^ mice. After 1 week, lymph node and spleen cells were stained with Thy1.1-PE and CD4–Pacific Blue, and the number of Thy1.1^+^ T cells was determined using Cytoflex.

### Implantable TC-1 and AE-17.ova tumor cell models.

TC-1 cells were cultured in RPMI-1640 medium supplemented with 10% FBS, 2 mM glutamine, and 5 μg/mL penicillin/streptomycin. AE-17.ova cells were cultured in DMEM supplemented with 10% FBS, 2 mM glutamine, and 5 μg/mL penicillin/streptomycin. For tumor studies, after mice were anesthetized, 200 μL of TC-1 and AE-17.ova cell suspensions resuspended in PBS were collected with a sterile syringe. Each mouse was shaved on their right flank and injected s.c. with 1.2 × 10^6^ TC-1 or 2 × 10^6^ AE-17.ova tumor cells. After implantation of TC-1 and AE-17.ova tumor cells, the long (*L*) and wide (*W*) diameters of the tumors were monitored daily, and tumor volumes were determined using the formula *V* = (*W*^2^ × *L*)/2.

### Orthotopic HCC model.

An orthotopic murine HCC model was established by subcapsular injection of 1 × 10^6^ HCC tumor cells in 30 μL Matrigel Basement Membrane Matrix (BD Biosciences) into the paramedian area of the lower surface of the left liver lobe (54). After anesthesia, the mice were fixed on the operation board, and the abdomen of the mice was fully sterilized with 75% alcohol or iodine complex. The abdominal skin, peritoneum, and other tissues of mice were opened by tissue cutting, and the abdominal cavity and organs were exposed using a mouse abdominal cavity opener, and the mouse liver was exposed. After subcapsular injection of the HCC tumor cells, sterile swab pressure was applied to the puncture site to prevent hemorrhage. After the injection, the injection site was quickly closed with medical glue to prevent the cells from being squeezed and spilled and liver bleeding. After the injection point was completely closed and no bleeding point was found, the device was withdrawn, and the abdominal cavity of mice was closed layer by layer with sterile 4-0 silk thread to ensure that the abdominal cavity was closed, and the abdominal cavity was fully disinfected with iodine again. After complete resuscitation, the mice were transferred to an animal room, the activity of the mice was observed every day after surgery, and samples were taken 10 and 21 days after surgery.

### Single-cell generation.

After obtaining tumor tissue, spleen, and lymph nodes, tumor tissues were mechanically dissociated. Digestion was performed in DMEM containing 80 U/mL DNase I, 300 U/mL collagenase I, and 60 U/mL hyaluronidase (all from Sigma-Aldrich) for 30 minutes at 37°C. The reaction was inactivated with FBS. For spleens and lymph nodes, single-cell suspensions were obtained by mechanical grinding and passage through a 40-μm cell strainer. Erythrocytes were lysed, and the cells were washed and resuspended in PBS with 2% FBS. Cell number and viability were assessed using a Nexcelom Cellometer Auto2000 with AOPI staining solution in PBS.

### Intracellular cytokine staining and flow cytometry.

For analysis of intracellular cytokines, single cells were seeded into 96-well plates and incubated in RPMI-1640 medium supplemented with 10% FBS. T cells were activated by adding PMA (5 ng/mL) and ionomycin (2 μg/mL) and incubated for 4 hours at 37°C. After fixation and permeabilization using the eBioscience Foxp3/Transcription Factor Staining Buffer (Thermo Fisher Scientific, 00-5523-00), cells were incubated for 30 minutes at 4°C in the dark. Following washing, cells were stained in a permeabilizing solution with flow Abs for 30 minutes, washed, and analyzed by flow cytometry. Data were acquired on a NovoCyte Flow Cytometer using NovoExpress software (Agilent Biosciences) and analyzed with NovoExpress. The gating strategy and Abs used for flow cytometry are listed in Supplemental Table 12, and the numbers of cells for each cell subset per flow cytometric analysis are shown in Supplemental Table 13.

### ChIP and RNA-seq.

EZ-Magna Chip A Chromatin Immunoprecipitation kits (Millipore) were used. Teffs or Tregs were fixed with 1% formaldehyde and subjected to sonication to shear the chromatin DNA into sizes ranging from 200 to 500 bp. Chromatin was immunoprecipitated with Abs against acetyl–histone H3 (Lys27) and HDAC8 (Cell Signaling Technology, 66042S and 8173). After reverse crosslinking, ChIP DNA was purified using DNA purification columns (Zymo Research) and subjected to downstream real-time qPCR analysis (ChIP-qPCR) or library preparation for deep sequencing (ChIP-seq). ChIP-seq library was prepared using an NEBNext Library Prep Kit (New England Biolabs) following the instructions and subjected to paired-end sequencing on the Illumina platform. The ChIP-PCR primers are listed in Supplemental Table 14.

### Cardiac transplantation.

Heterotopic cardiac allografts were performed using BALB/c donors and WT or Rag1^–/–^ recipients (C57BL/6 background), as described previously (55). In adoptive transfer studies of Treg-dependent allograft survival, Tregs (0.5 × 10^6^) and Teffs (1 × 10^6^) from WT mice were isolated using magnetic beads and injected intravenously into Rag1^–/–^ mice bearing BALB/c cardiac allografts. In related pharmacologic studies, Rag1^–/–^ allograft recipients were treated with an HDAC8i (OJ-1, 5 mg/kg/d) or DMSO for 14 days following adoptive transplantation. Graft survival was monitored to assess the ability of Tregs to suppress Teff-dependent alloreactivity and cardiac allograft rejection.

### Cell transfection and co-IP assay.

293T cells were transfected for 48 hours with indicated plasmids using Lipofectamine 2000 transfection reagent (Thermo Fisher Scientific). For co-IP to detect endogenous protein interactions, tumor cells were lysed in 500 μL of lysis buffer and immunoprecipitated with the indicated Ab and protein G magnetic beads. After 3 washes with lysis buffer, IPs were resolved by SDS-PAGE, followed by Western blot analysis.

### Mass spectrometry.

Teffs and Tregs were lysed in IP lysis buffer (Thermo Fisher Scientific) containing a protease and phosphatase inhibitor cocktail (Thermo Fisher Scientific). The lysate was subsequently adjusted to 1 μg/μL and placed on ice until use. Thirty microliters of protein A/G magnetic beads (Thermo Fisher Scientific) were added to a 1.5-mL Eppendorf tube and placed in a DynaMag magnet (Thermo Fisher Scientific), and then the supernatant was discarded. After repeated washing of magnetic beads, primary Abs (HDAC8 Ab, 2 μg) and IgG (2 μg) diluted with 200 μL IP lysate were added and incubated for 2 hours at room temperature with rotation. The supernatant of the magnetic beads–Ab complex was discarded, and 200 μg of lysate was incubated with the magnetic beads–Ab complex overnight at 4°C with gentle rotation. The treated bead-Ab-Ag complexes were analyzed by mass spectrometry. In brief, HDAC8 and control co-IPs were digested on-bead using the PreOmics iST kit per the manufacturer’s protocol (56). The resulting peptides were dried via vacuum centrifugation, and reconstituted in 0.1% TFA containing Biognosys iRT peptides. Peptides were analyzed on an Exploris 480 mass spectrometer coupled with an Ultimate 3000 nano UPLC system and an EasySpray source utilizing data-independent acquisition. Raw data were searched using Spectronaut, and the bioinformatics analysis was conducted in R (57).

### Foxp3 DNA methylation analysis.

The extent of CpG DNA methylation at the Foxp3 CNS2 region was determined by sodium bisulfite conversion, followed by PCR amplification and EpiTYPER DNA methylation analysis. DNA was extracted from the purified Tregs and Teffs (2 μg), and sodium bisulfite conversion of cytosine to uracil was performed as previously described (32). The sample was amplified using a specially designed pair of primers (CNS2-F: TTGTTTAGTGGTATTAGGGATTTGG. CNS2-R: TACTCACCAAACATCCAACCTTAAA) to obtain an amplification product with the T7 RNA polymerase promoter sequence. In an in vitro transcription system, the amplified product was transcribed into an RNA fragment using T7 RNA polymerase. RNase A was used to specifically recognize and cleave the uracil from the 3′ end of the RNA, and the RNA fragment was cleaved into small fragments carrying CpG sites. The samples were detected using an Agena MassArray time-of-flight mass spectrometry system. After obtaining the raw data, the methylation and unmethylation of Foxp3 CNS2 were calculated by comparing the areas of CpG peaks and CpA peaks.

### Immunohistology.

Tumor samples and their adjacent normal counterparts were obtained from Zhongnan Hospital of Wuhan University. All tissue samples were validated by pathologists and stored at –80°C until examination. For immunoperoxidase, tissue specimens were fixed in 4% paraformaldehyde, embedded in paraffin, and sectioned into 4-μm-thick slices for slide preparation. After gradient deparaffinization and rehydration, antigen retrieval was performed using a microwave method with citrate buffer (100°C, 4 cycles of 7 minutes each). The slides were washed extensively with PBS and blocked for 30 minutes to minimize nonspecific binding. The primary antibody was incubated overnight at 4°C, followed by incubation with the secondary antibody at room temperature. Color development was achieved using DAB chromogen, and the sections were counterstained with hematoxylin. For immunofluorescence, tissues were fixed with 4% paraformaldehyde and made into paraffin sections. Paraffin sections were deparaffinized and underwent antigen retrieval with 1× One-step Dewaxing/Antigen Retrieval Buffer (pH 6.0; Elabscience). Sections were permeabilized and blocked with 10% goat serum (Bioss) plus 0.3% Triton X-100 (Solarbio) for 1 hour at room temperature and incubated with primary antibodies at 4°C overnight. Slides were rinsed and secondary antibodies were applied for 1 hour at room temperature. Slides were rinsed, restained with DAPI (Sigma-Aldrich; 1:30,000), and imaged using a Zeiss LSM 980 microscope with Airyscan 2.

### Statistics.

Data were analyzed using GraphPad Prism 8.0 and are presented as mean ± SD unless otherwise specified. Comparisons between 2 groups utilized a 2-tailed Student’s *t* test for normally distributed data or a Mann-Whitney *U* test for non-normally distributed data. For multiple comparisons, we used the 2-way ANOVA method for statistical analysis. For data presented as ratios, a 1-sample *t* test (theoretical mean = 1) was used for normally distributed data, while Wilcoxon’s signed-rank test (theoretical median = 1) was applied for non-normally distributed data. Bonferroni’s test was used to correct all *P* values for multiple comparisons when necessary.

### Study approval.

Animal studies were approved by the Institutional Animal Care and Use Committee of the Children’s Hospital of Philadelphia (protocols 17-001047 and 19-000561).

### Data availability.

The datasets presented in this study can be found in online repositories. The names of the repository/repositories and accession number(s) are the Sequence Read Archive (https://www.ncbi.nlm.nih.gov/sra/, accession number: PRJNA1137231). Values for all data points in graphs are reported in the Supporting Data Values file.

## Author contributions

FK prepared the first draft of the manuscript. FK and LW undertook material preparation, data collection and analysis and, with RH and YX, carried out biochemical and animal experiments. HF, JR, and LS undertook the mass spectrometry studies. LW carried out all other experiments. ABB provided HDAC8i and insights into HDAC8 biology. WWH designed the studies, interpreted the results, and edited the final manuscript. All authors read and approved the final manuscript.

## Funding support

This work is the result of NIH funding, in whole or in part, and is subject to the NIH Public Access Policy. Through acceptance of this federal funding, the NIH has been given a right to make the work publicly available in PubMed Central.

- NIH grant 5P01 AI073489-01A1 (to WWH).
- Foerderer Award for Excellence from The Children’s Hospital of Philadelphia (to WWH).
- Fred and Suzanne Biesecker Pediatric Liver Center at The Children’s Hospital of Philadelphia (to WWH).
- National Natural Science Foundation of China, grant no. 82271810.

## Supplementary Material

Supplemental data

Unedited blot and gel images

Supporting data values

## Version 1 12/11/2025

In-Press Preview

## Version 2 02/09/2026

Electronic publication
