# Supplementary material for: Differential effects of HDAC8 targeting on Foxp3+ Tregs and effector T cells promote antitumor immunity
Source: JCI Insight. 2025 Dec 11;11(3):e186461. doi: 10.1172/jci.insight.186461 (PMC12892887; doi:10.1172/jci.insight.186461)
Supplement: Supplemental data [file jciinsight-11-186461-s048.pdf]

# Supplemental material

## Differential Effects of HDAC8 Targeting on Foxp3+ T-regulatory Cells and Effector T-Cells Promote Anti-tumor Immunity

Fanhua Kong,[1,6] Yan Xiong,[1] Liqing Wang,[2] Rongxiang Han,[2] Hossein Fazelinia,[3] Jennifer Roof,[3] Lynn Spruce,[3] Aaron B. Beeler,[4] Wayne W. Hancock[2,5*]

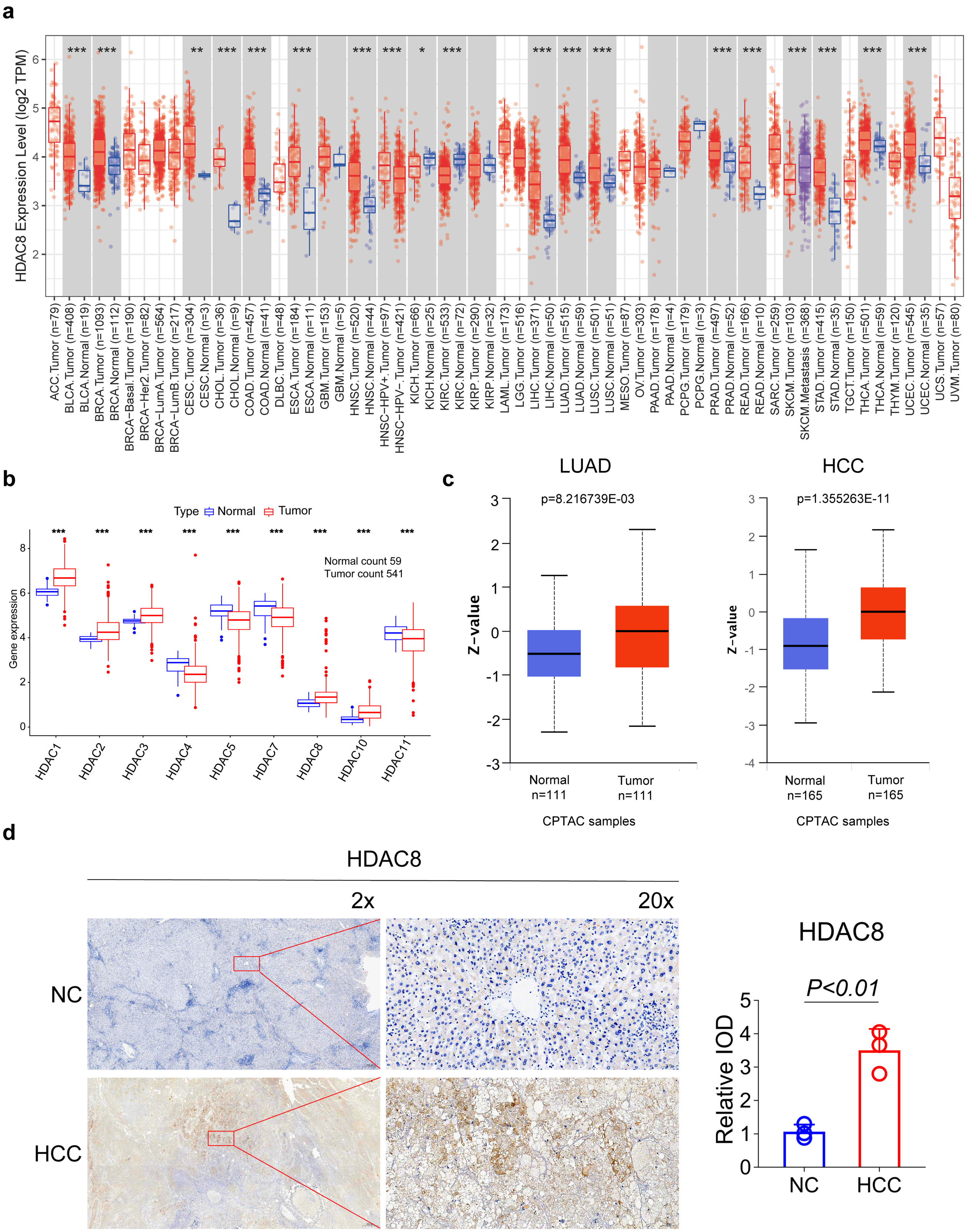


**Fig. S1**: HDAC8 expression is upregulated in human solid tumors. (a) TIMER2 was used to analyze the expression of the HDAC8 gene in different cancers or specific cancer subtypes. (b) TCGA database was used to analyze the expression levels of HDAC family genes in LUAD. (c) CPTAC module of UALCAN database was used to analyze the expression differences of HDAC8 between LUAD, HCC and normal tissues. (d)

Immunohistochemical analysis of HDAC8 in HCC and peri-cancerous tissues. Statistical analysis involved comparisons between two groups using a two-tailed Student's t-test for normally distributed data.

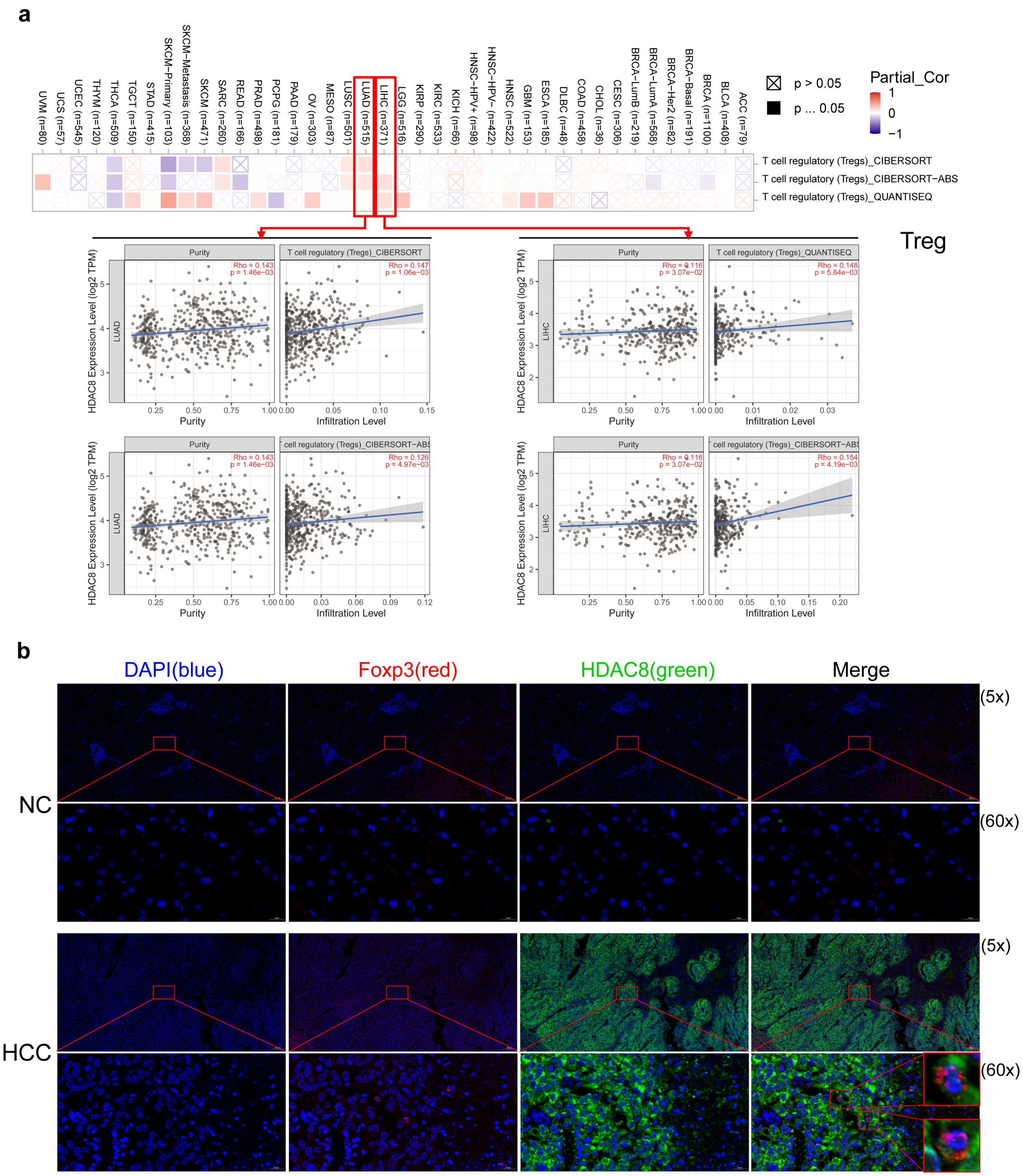


**Fig. S2**: HDAC8 promotes Treg infiltration in human solid tumors. (a) TIMER2 database was used to analyze the correlation between immune infiltration of Treg and HDAC8 expression. We used different algorithms to analyze the potential correlation between HDAC8 gene expression level and Treg invasion level in all types of cancer in the TCGA. (b) Representative combined staining of HDAC8 (green), Foxp3 (red), and 4′,6-diamidino-2-phenylindole (DAPI) (blue) in tumor tissues and para-carcinoma tissues of patients with HCC. Statistical analysis involved comparisons between two groups using a two-tailed Student's t-test for normally distributed data.

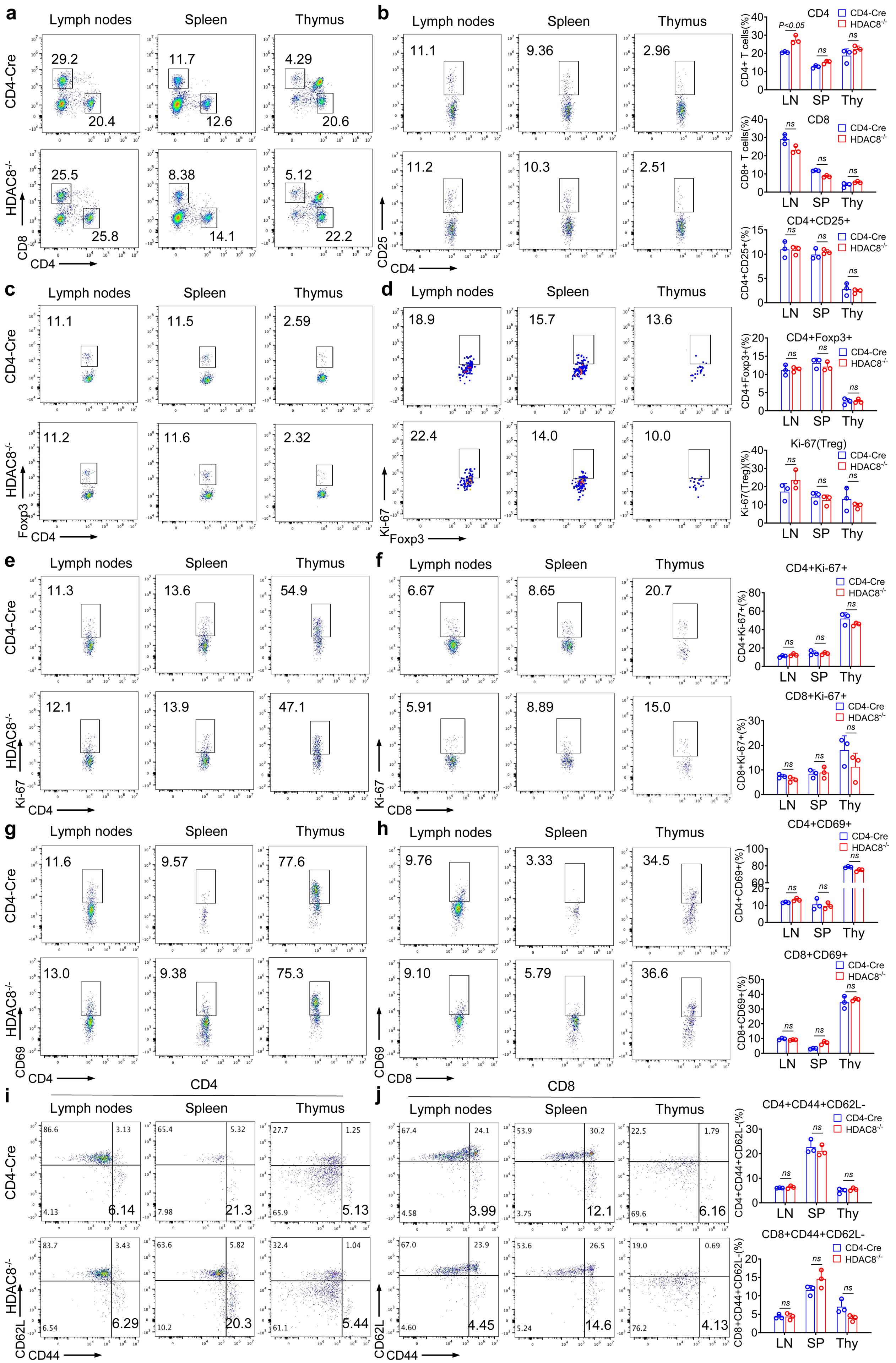

a
b
c
d
e
f
g
h
i
j
Lymph nodes
Spleen
Thymus
CD4-Cre
HDAC8-/-
CD8
CD4
CD25
Foxp3
Ki-67
CD69
CD62L
CD44
CD4+ T cells(%)
CD8+ T cells(%)
CD4+CD25+
CD4+Foxp3+
Ki-67(Treg)
CD4+Ki-67+
CD8+Ki-67+
CD4+CD69+
CD8+CD69+
CD4+CD44+CD62L-
CD8+CD44+CD62L-
P<0.05
ns
LN
SP
Thy

**Fig. S3**: Conditional knockout of HDAC8 has negligible effects on T cell development. (a) Flow cytometry was used to analysis the expression levels of CD4+/CD8+ T cells in lymph nodes, spleen and thymus of CD4-Cre and HDAC8$^{-/-}$ mice. (b-c) Flow cytometry was used to analysis the expression levels of Tregs in lymph nodes, spleen and thymus of CD4-Cre and HDAC8$^{-/-}$ mice. (d-f) Flow cytometry was used to analysis the expression levels of Ki-67 in lymph nodes, spleen and thymus of CD4-Cre and HDAC8$^{-/-}$ mice. (g-h) Flow cytometry was used to analysis the expression levels of CD4+CD69+ and CD8+CD69+ in lymph nodes, spleen and thymus of CD4-Cre and HDAC8$^{-/-}$ mice. (i-j) Flow cytometry was used to analysis the expression levels of CD44+CD62l- in lymph nodes, spleen and thymus of CD4-Cre and HDAC8$^{-/-}$ mice. Statistical analysis involved comparisons between two groups using a two-tailed Student's t-test for normally distributed data.

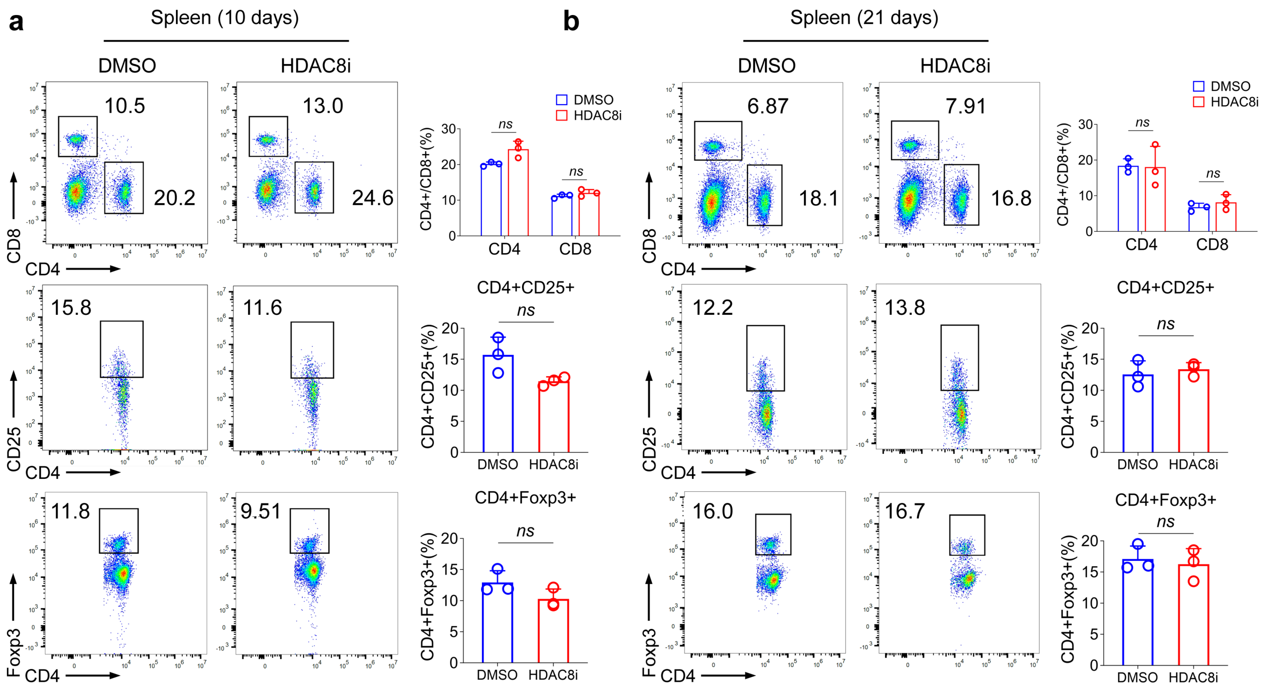


**Fig. S4**: (a) Flow cytometry was used to analyze the expression of CD4+ T cells (Gate: CD4+), CD8+ T cells (Gate: CD8+) and Treg cells (Gate: CD4+Foxp3+, CD4+CD25+) in spleen at 10 days after implantation of H22 cells(n=3/group). (b) Flow cytometry was used to analyze the expression of CD4+ T cells (Gate: CD4+), CD8+ T cells (Gate: CD8+) and Treg cells (Gate: CD4+Foxp3+, CD4+CD25+) in spleen at 21 days after implantation of H22 cells(n=3/group). Assays were run in triplicate and repeated at least 3 times. The results of a representative experiment are shown. Data were expressed as the mean ± SD of three independent experiments (ns=not significant). Statistical analysis: Comparisons between two groups utilized a two-tailed Student's t-test for normally distributed data.

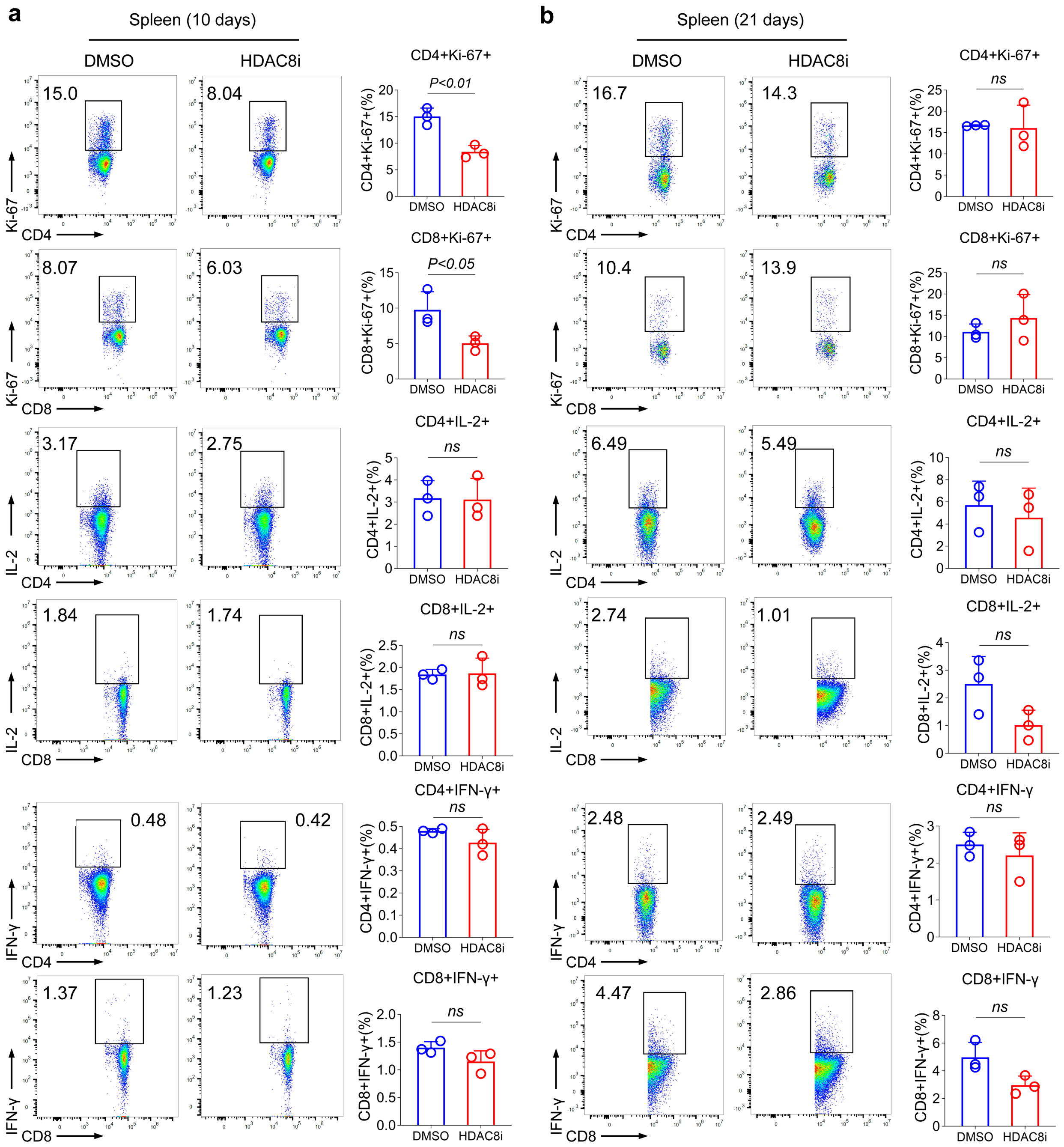


**Fig. S5**: (a) Flow cytometry was used to analyze the expression of Ki-67, IL-2 and IFN-γ in spleen at 10 days after implantation of H22 cells(n=3/group). (b) Flow cytometry was used to analyze the expression of Ki-67, IL-2 and IFN-γ in spleen at 21 days after implantation of H22 cells(n=3/group). Assays were run in triplicate and repeated at least 3 times. The results of a representative experiment are shown. Data were expressed as the mean ± SD of three independent experiments (ns=not significant). Statistical analysis involved comparisons between two groups using a two-tailed Student's t-test for normally distributed data.

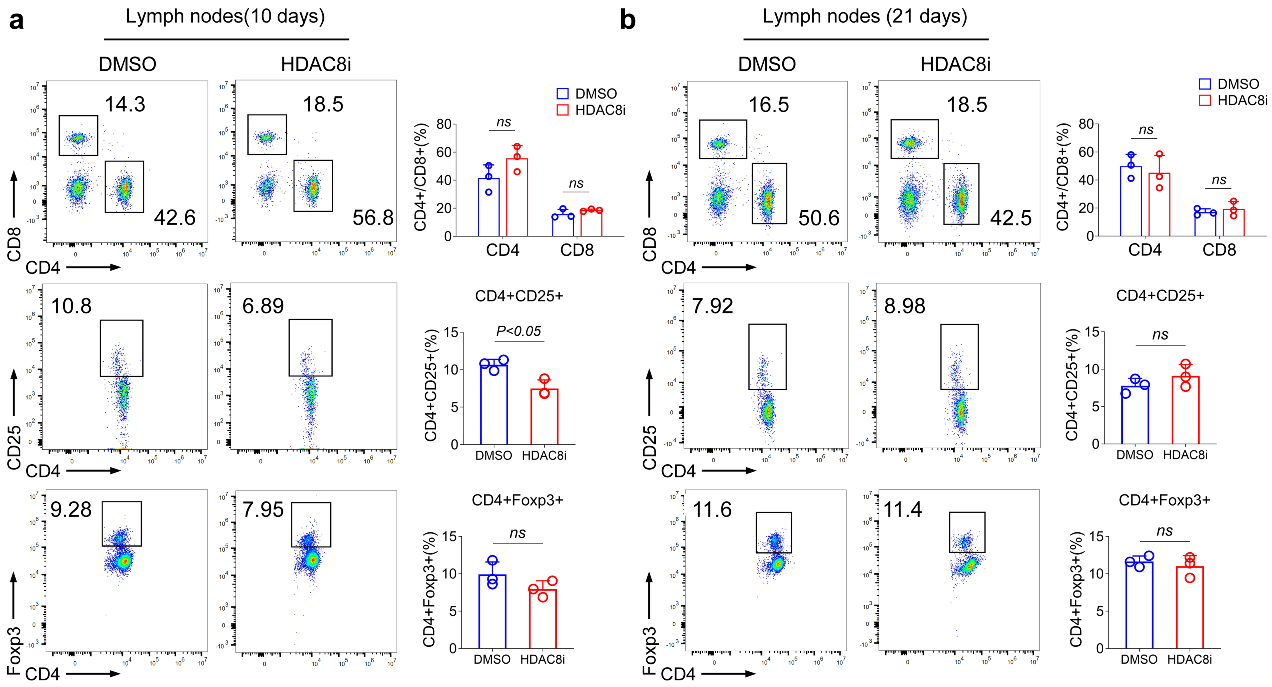


**Fig. S6**: (a) Flow cytometry was used to analyze the expression of CD4+ T cells (Gate: CD4+), CD8+ T cells (Gate: CD8+) and Treg cells (Gate: CD4+Foxp3+, CD4+CD25+) in lymph nodes at 10 days after implantation of H22 cells(n=3/group). (b) Flow cytometry was used to analyze the expression of CD4+ T cells (Gate: CD4+), CD8+ T cells (Gate: CD8+) and Treg cells (Gate: CD4+Foxp3+, CD4+CD25+) in lymph nodes at 21 days after implantation of H22 cells(n=3/group). Assays were run in triplicate and repeated at least 3 times. The results of a representative experiment are shown. Data were expressed as the mean ± SD of three independent experiments (ns=not significant). Statistical analysis: Comparisons between two groups utilized a two-tailed Student's t-test for normally distributed data.

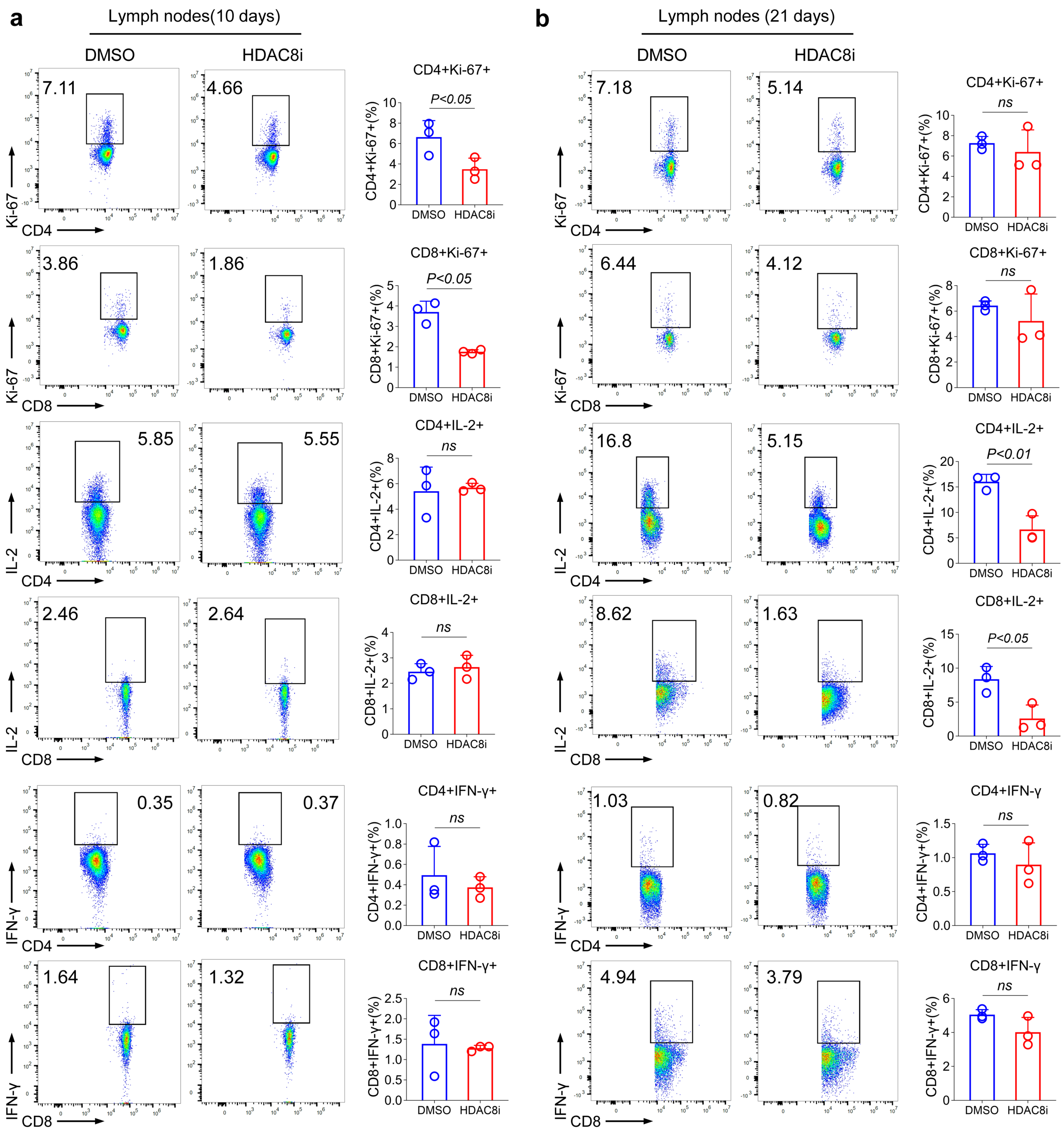


**Fig. S7**: (a) Flow cytometry was used to analyze the expression of Ki-67, IL-2 and IFN-γ in lymph nodes at 10 days after implantation of H22 cells(n=3/group). (b) Flow cytometry was used to analyze the expression of Ki-67, IL-2 and IFN-γ in lymph nodes at 21 days after implantation of H22 cells(n=3/group). Assays were run in triplicate and repeated at least 3 times. The results of a representative experiment are shown. Data were expressed as the mean ± SD of three independent experiments (ns=not significant). Statistical analysis: Comparisons between two groups utilized a two-tailed Student's t-test for normally distributed data.

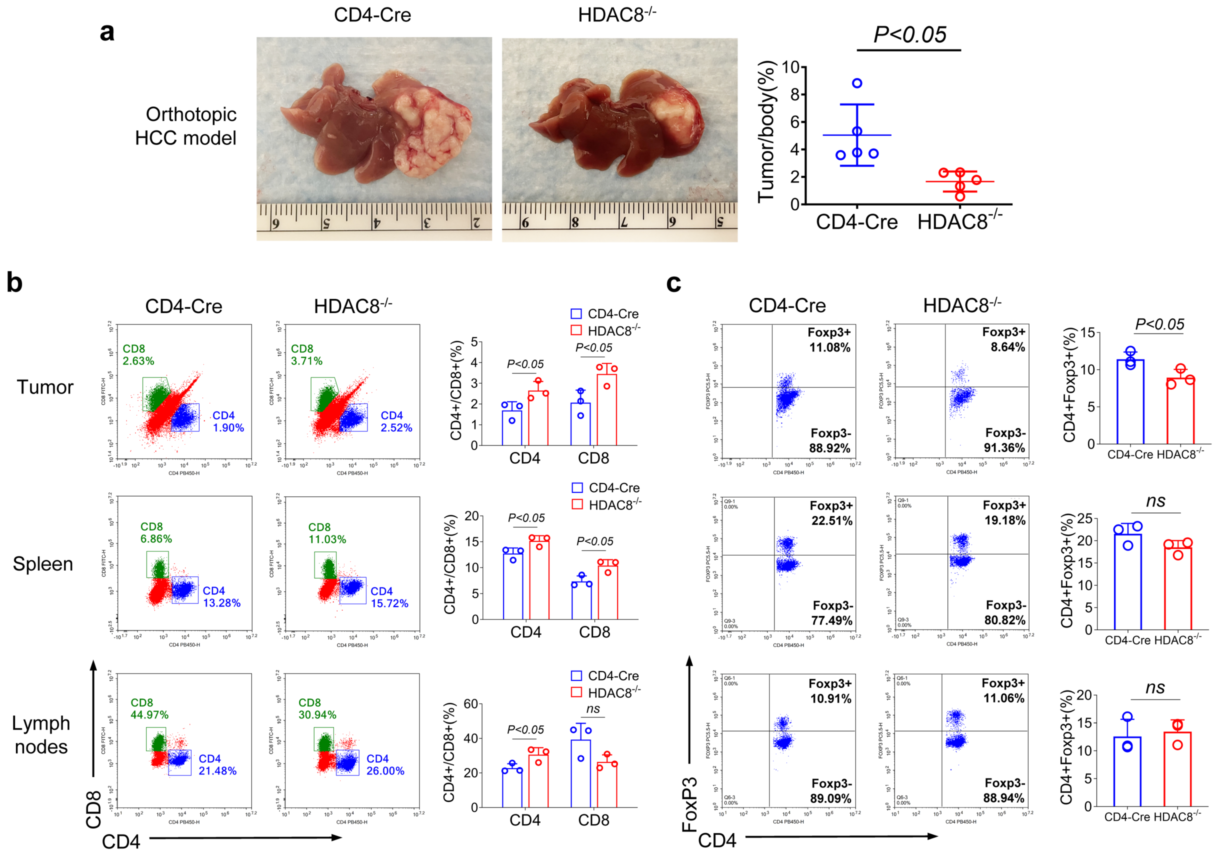


**Fig. S8**: Conditional deletion of HDAC8 promotes anti-tumor immunity in orthotopic HCC model. (a) Tumor/body weight ratios of WT and HDAC8$^{-/-}$ mice (n=5/group) at 21 days. (b, c) Data are expressed as changes in CD4+ T cells (Gate: CD4+), CD8+ T cells (Gate: CD8+), Treg (Gate: CD4+Foxp3+), and Teff cells (Gate: CD4+Foxp3-) in HCC tumors, spleen, and lymph nodes(n=3/group). Assays were run in triplicate and repeated at least 3 times. The results of a representative experiment are shown. Data were expressed as the mean ± SD of three independent experiments (ns=not significant). Statistical analysis: Comparisons between two groups utilized a two-tailed Student’s t-test for normally distributed data.

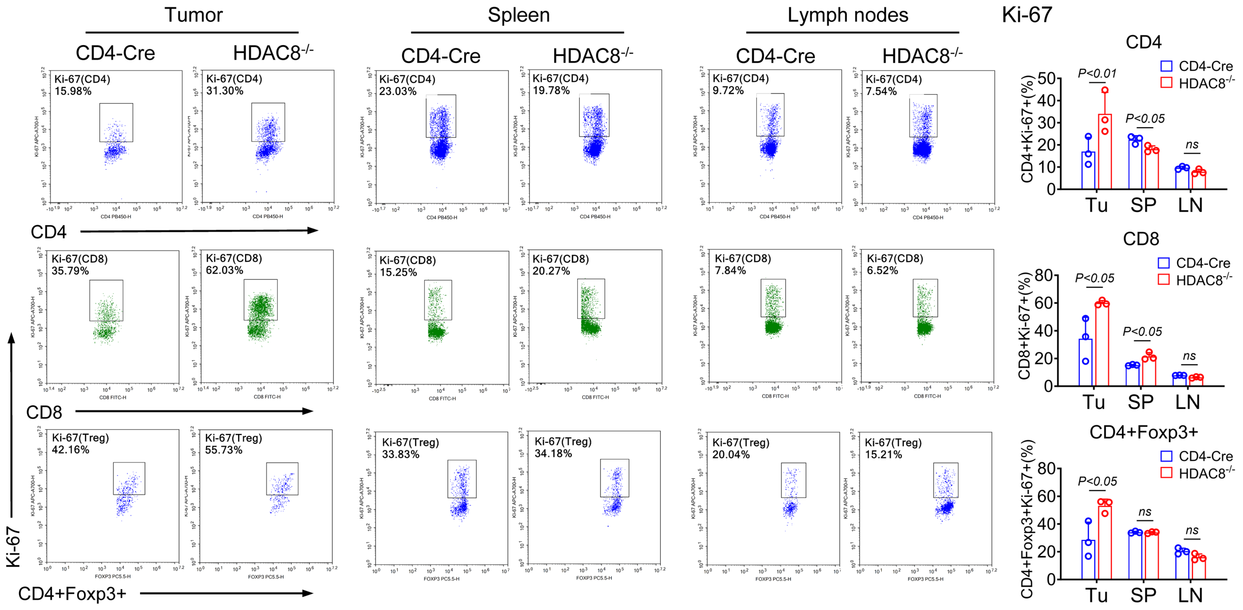


**Fig. S9**: Conditional knockout of HDAC8 promoted T cell proliferation in orthotopic HCC model. Expression of Ki-67 by tumor-infiltrating T cells, and spleen and lymph node T cells(n=3/group). Assays were run in triplicate and repeated at least 3 times. Data were expressed as the mean ± SD of 3 independent experiments (ns=not significant). Statistical analysis: Comparisons between two groups utilized a two-tailed Student's t-test for normally distributed data.

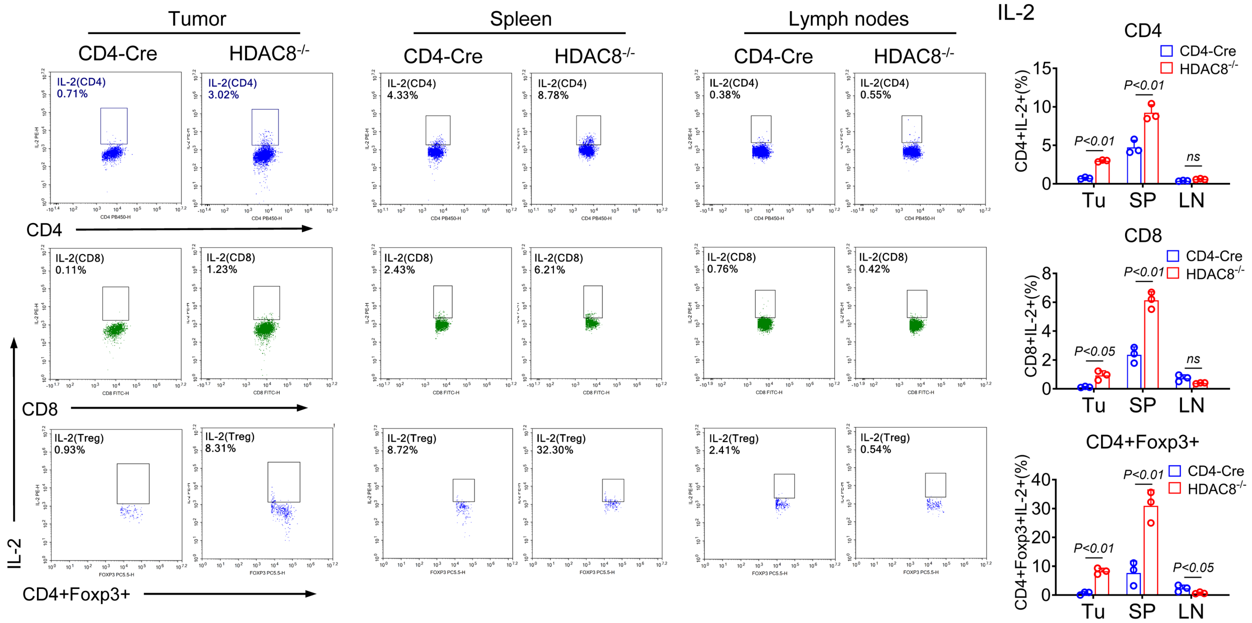


**Fig. S10**: Conditional knockout of HDAC8 promoted IL-2 production by CD4+, CD8+ T cells and Treg cells(n=3/group). Assays were run in triplicate and repeated at least 3 times. Data were expressed as the mean ± SD of 3 independent experiments (ns=not significant). Statistical analysis: Comparisons between two groups utilized a two-tailed Student's t-test for normally distributed data.

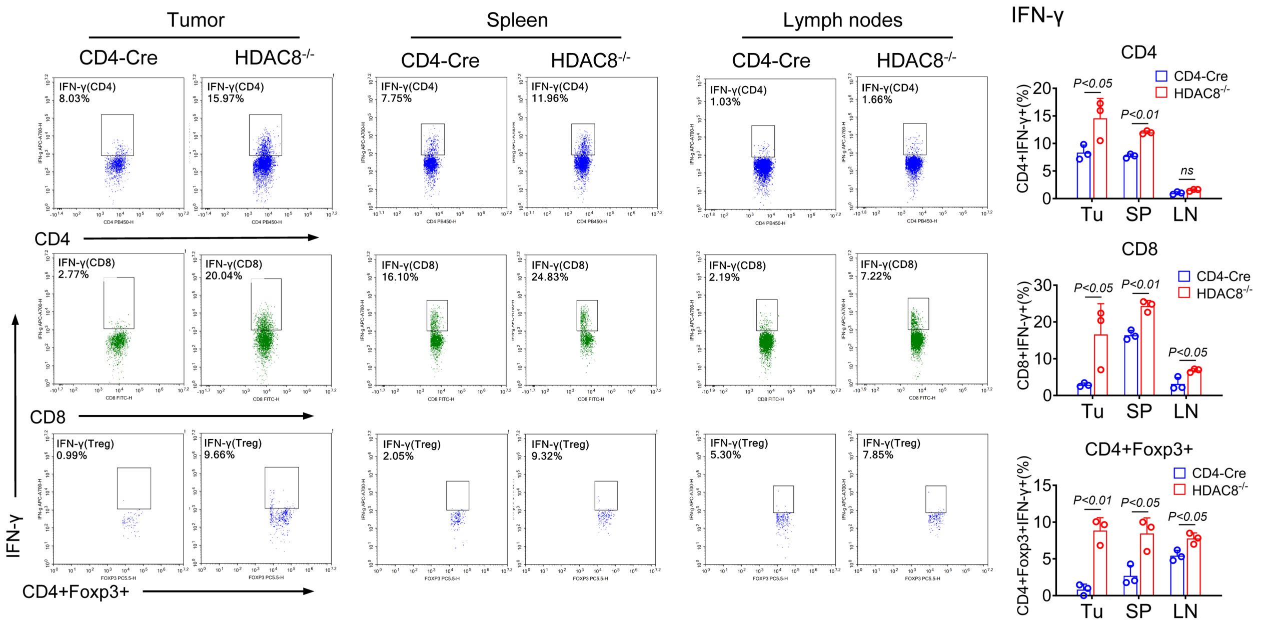


**Fig. S11**: Conditional knockout of HDAC8 promoted IFN-γ production by CD4+, CD8+ T cells and Treg cells(n=3/group). Assays were run in triplicate and repeated at least 3 times. Data were expressed as the mean ± SD of 3 independent experiments (ns=not significant). Statistical analysis: Comparisons between two groups utilized a two-tailed Student's t-test for normally distributed data.

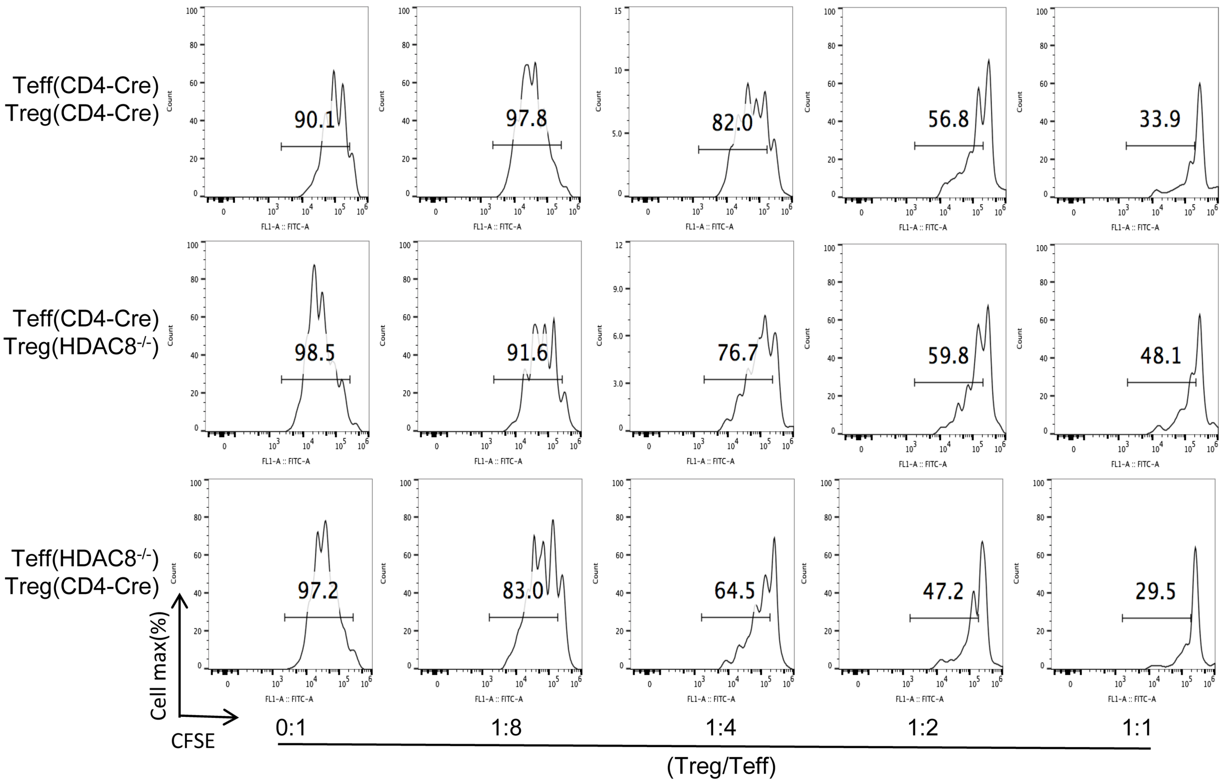


**Fig. S12**: Treg suppression assays using pooled Treg and Teff cells from lymph nodes and spleens of mice (CD4-Cre and HDAC8$^{-/-}$), as indicated.

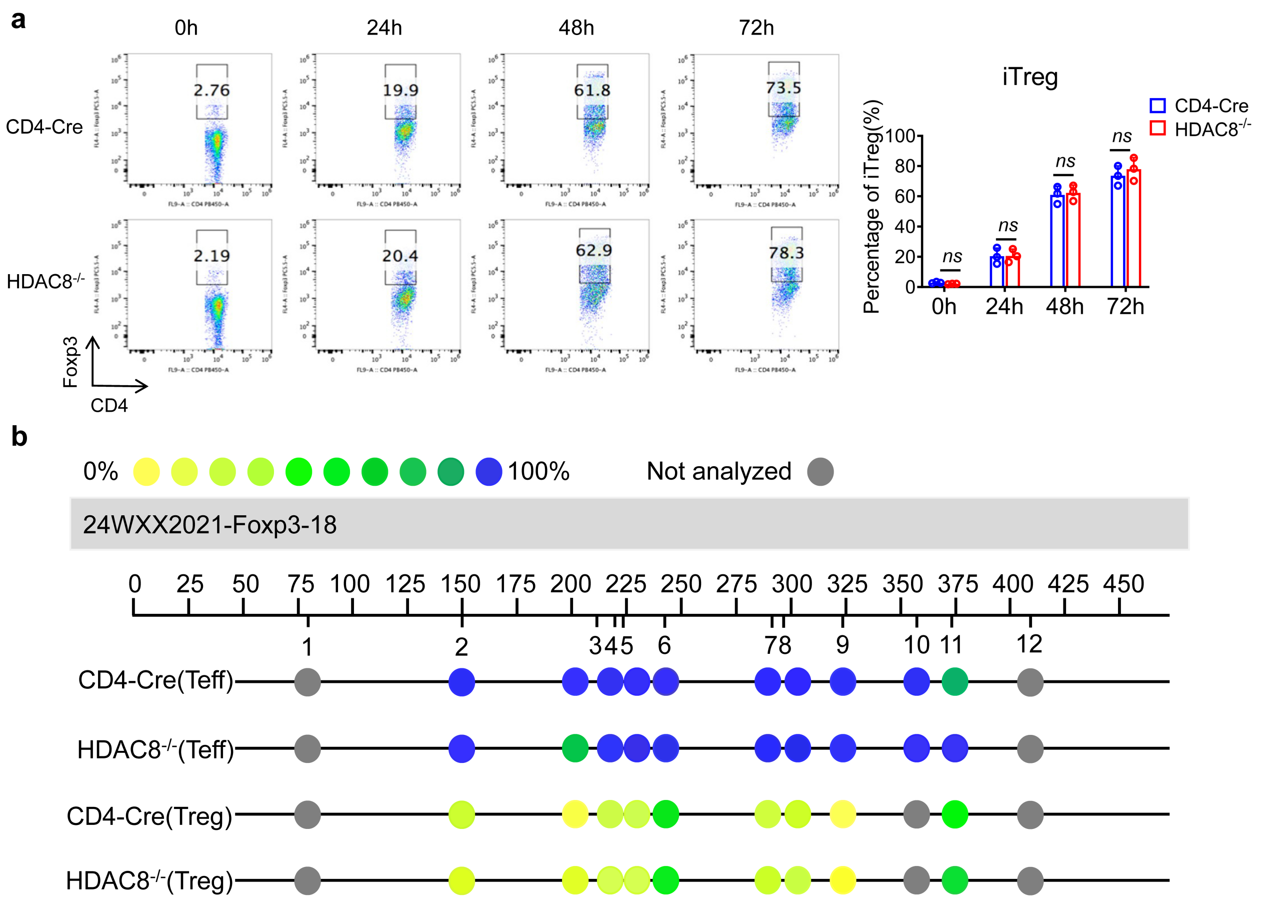


**Fig. S13**: (a) Flow cytometry analysis of CD4-Cre and HDAC8$^{-/-}$ iTreg cells were stimulated with CD3/CD28 mAb-coated beads (1:1) plus TGF-β and IL-2 for the indicated times (n=3/group). For phenotypic analysis, we labeled iTreg cells with CD4, CD25, and Foxp3 antibodies and calculated the percentage of Foxp3+ CD4+ cells to determine the efficiency of iTreg cells after 24 h, 48 h, and 72 h of TGF-β and IL-2 treatment. (b) Dot chart of CpG sites with different degrees of methylation based on raw data for CD4-Cre and HDAC8$^{-/-}$ groups. Methylation dot icon ruler: The lighter the color, the lower the degree of methylation, and the darker the color, the higher the degree of methylation. The sequence ruler of the gene: The upper part of the ruler is the base position, and the lower part is the CpG logo and approximate position. Data were expressed as the mean ±SD of three independent experiments (ns=not significant). Statistical analysis: Comparisons between two groups utilized a two-tailed Student's t-test for normally distributed data.

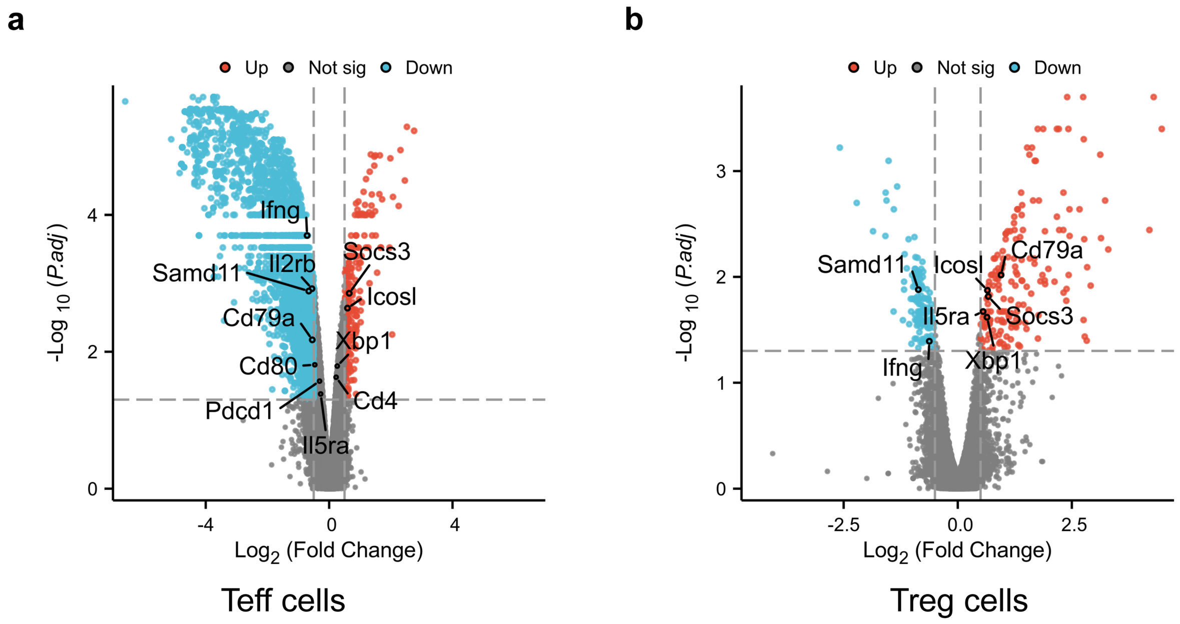


**Fig. S14**: Volcano plot showing statistical significance (Padj) vs. fold change for genes differentially expressed as a result of HDAC8 deletion in Teff cells (a) and Foxp3+ Tregs (b).

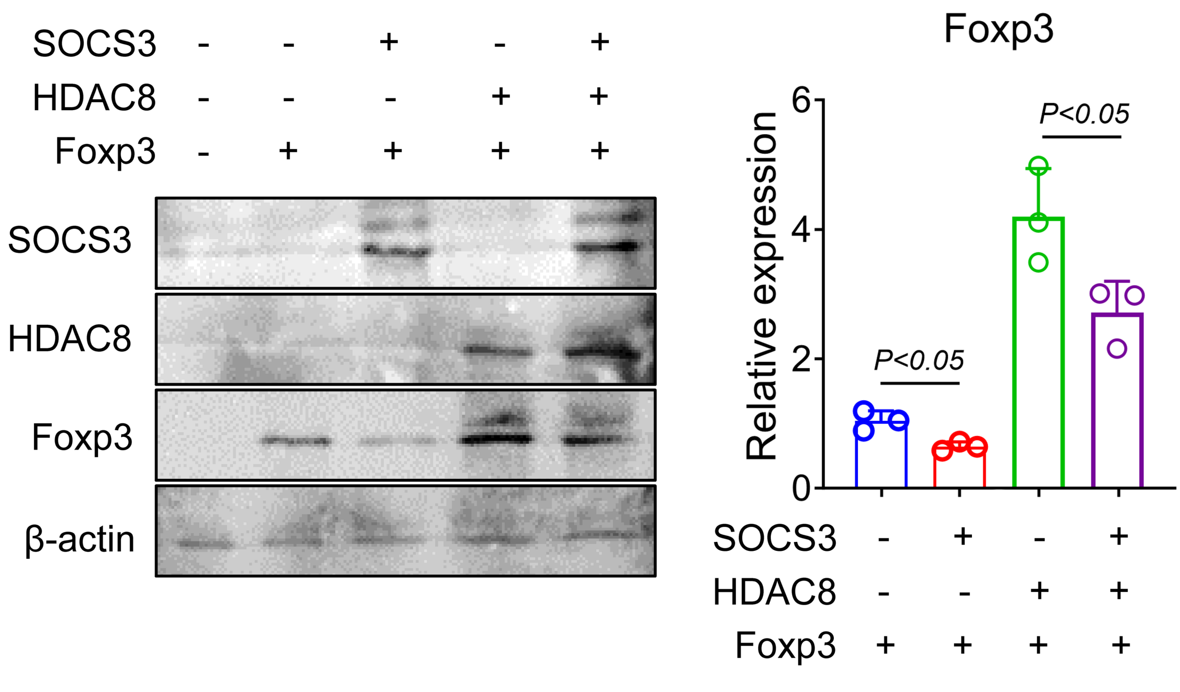


**Fig. S15**: Co-transfection analysis of HDAC8, Socs3 and Foxp3 in 293T cells (n=3/group). Assays were run in triplicate and repeated at least 3 times. Data were expressed as the mean ± SD of 3 independent experiments. Statistical analysis: Comparisons between two groups utilized a two-tailed Student’s t-test for normally distributed data.

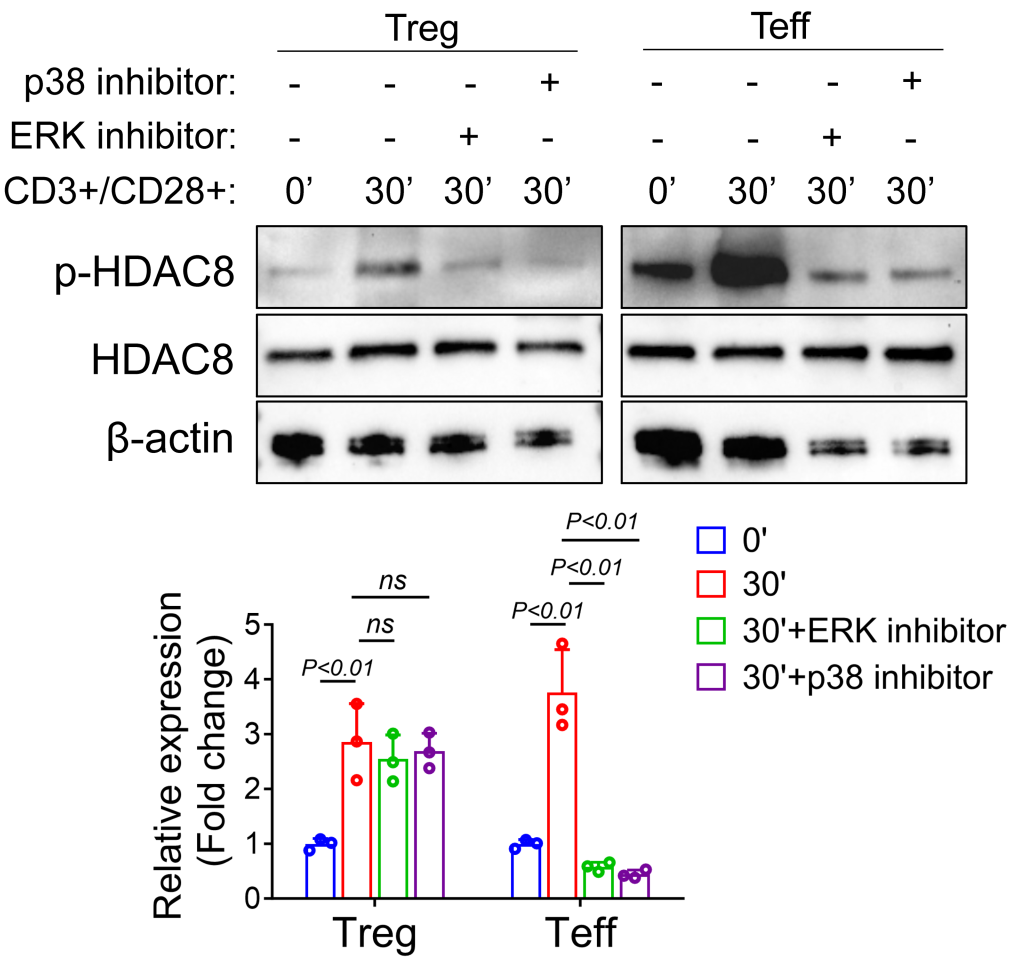


**Fig. S16**: Western blot was used to analysis the expression of p-HDAC8 in Teff and Treg cells stimulated with CD3/CD28 mAbs. Data were expressed as the mean ± SD of 3 independent experiments (ns=not significant). Statistical analysis: Comparisons between two groups utilized a two-tailed Student’s t-test for normally distributed data. For multiple comparisons, we used the two-way ANOVA method for statistical analysis.

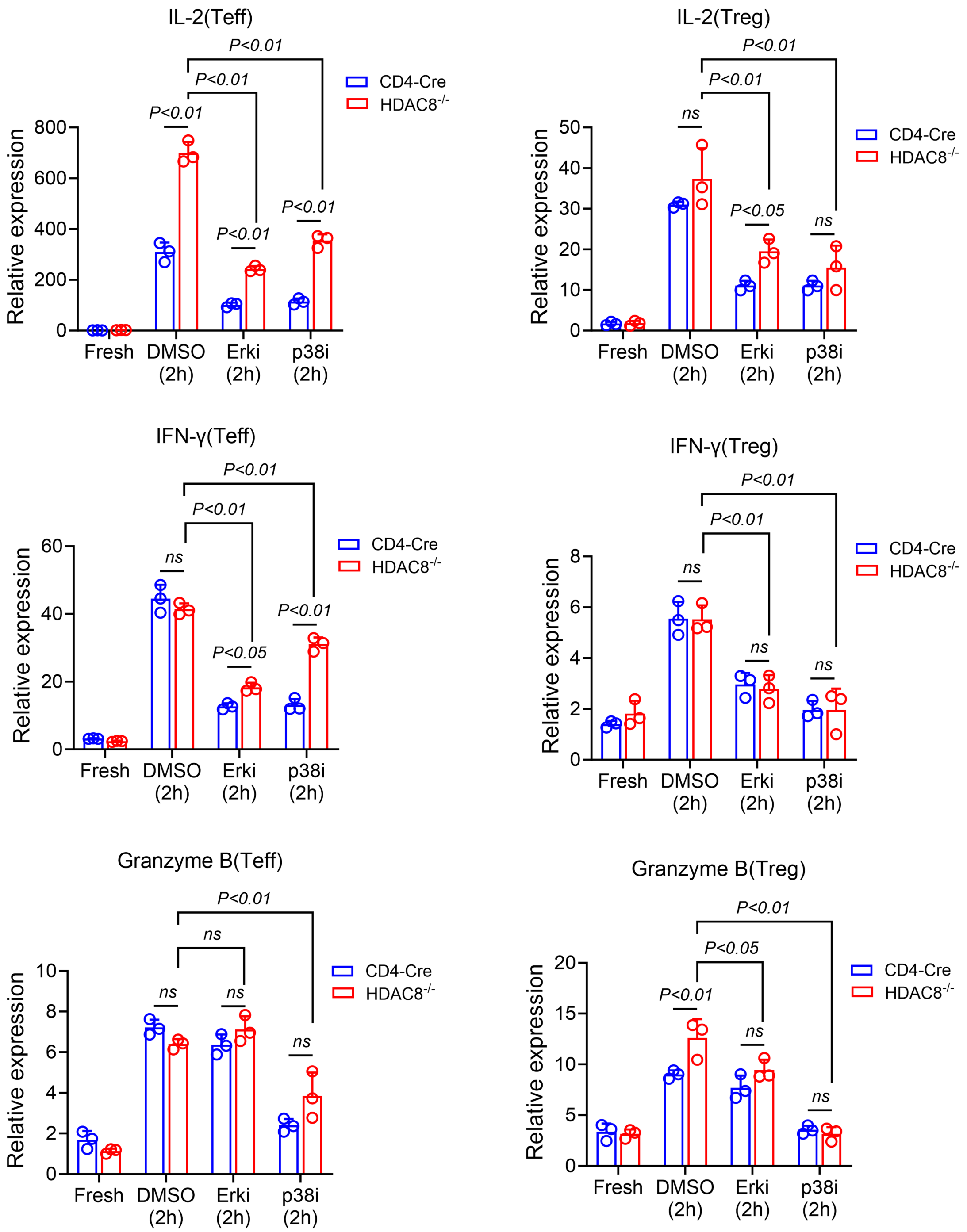


**Fig. S17**: The expression of IL-2, IFN-γ and Granzyme B in Teff/Treg cells stimulated with CD3/CD28 mAbs and treated with ERK/p38 inhibitor was detected by PCR. Data were expressed as the mean ± SD of 3 independent experiments (ns=not significant). Statistical analysis: Comparisons between two groups utilized a two-tailed Student's t-test for normally distributed data. For multiple comparisons, we used the two-way ANOVA method for statistical analysis.

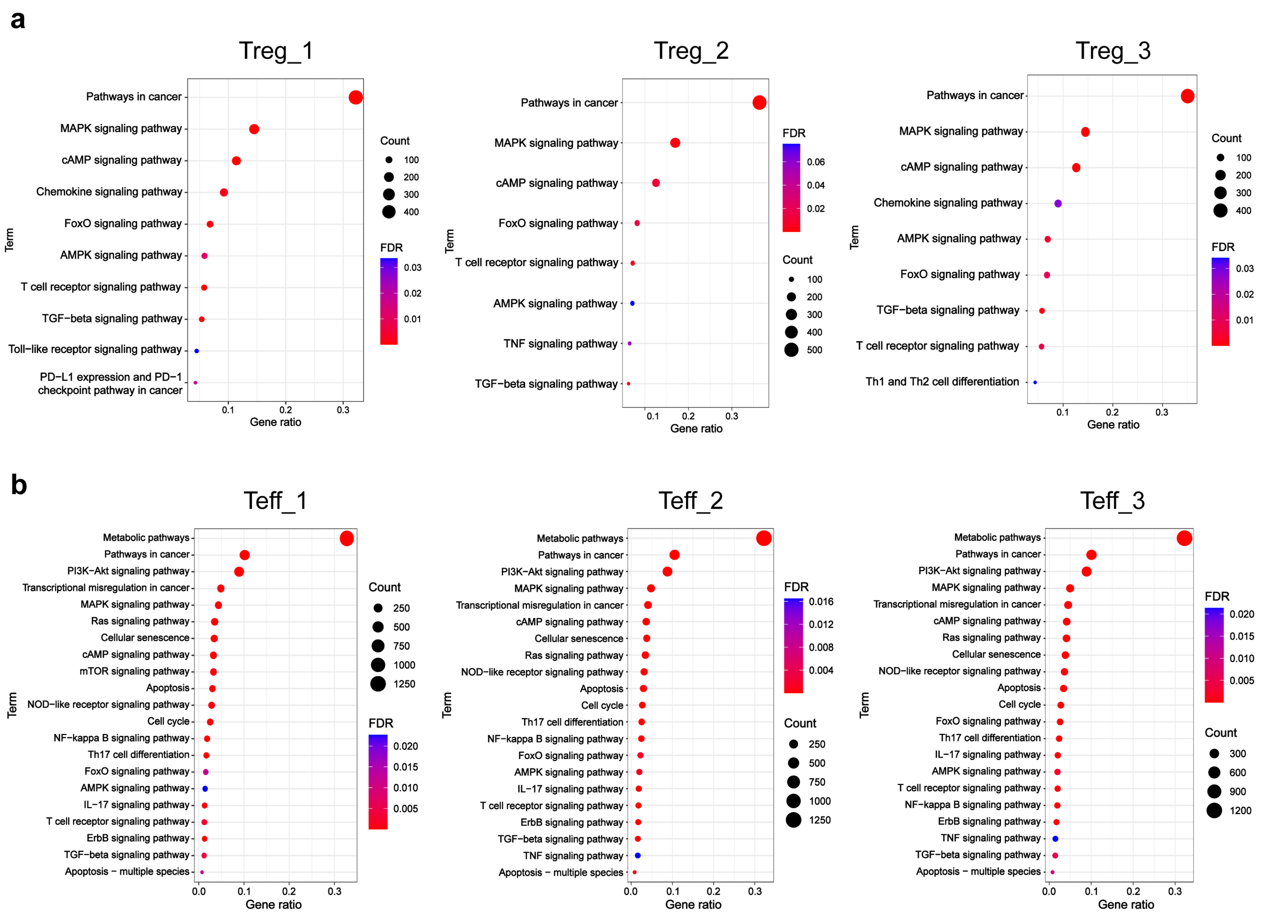


**Fig. S18**: HDAC8 ChIP-seq analysis in Treg (a) and Teff (b) cells (KEGG enrichment analysis).

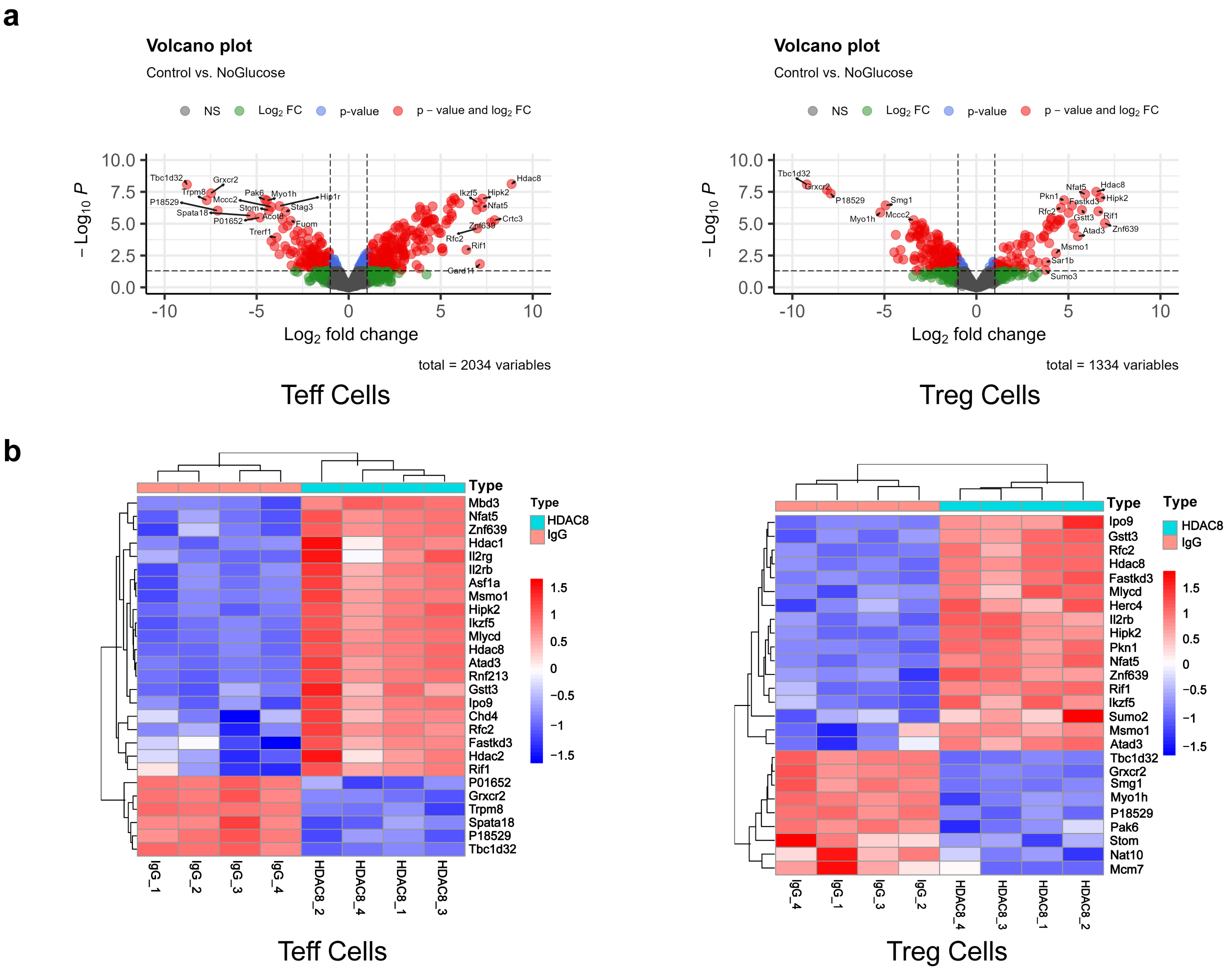


**Fig. S19**: HDAC8 mass spectrometry analysis in Treg and Teff cells. (a) Volcanic dot plot of HDAC8 mass spectrometry analysis in Treg and Teff cells. (b) Heat map of HDAC8 mass spectrometry analysis in Treg and Teff cells.

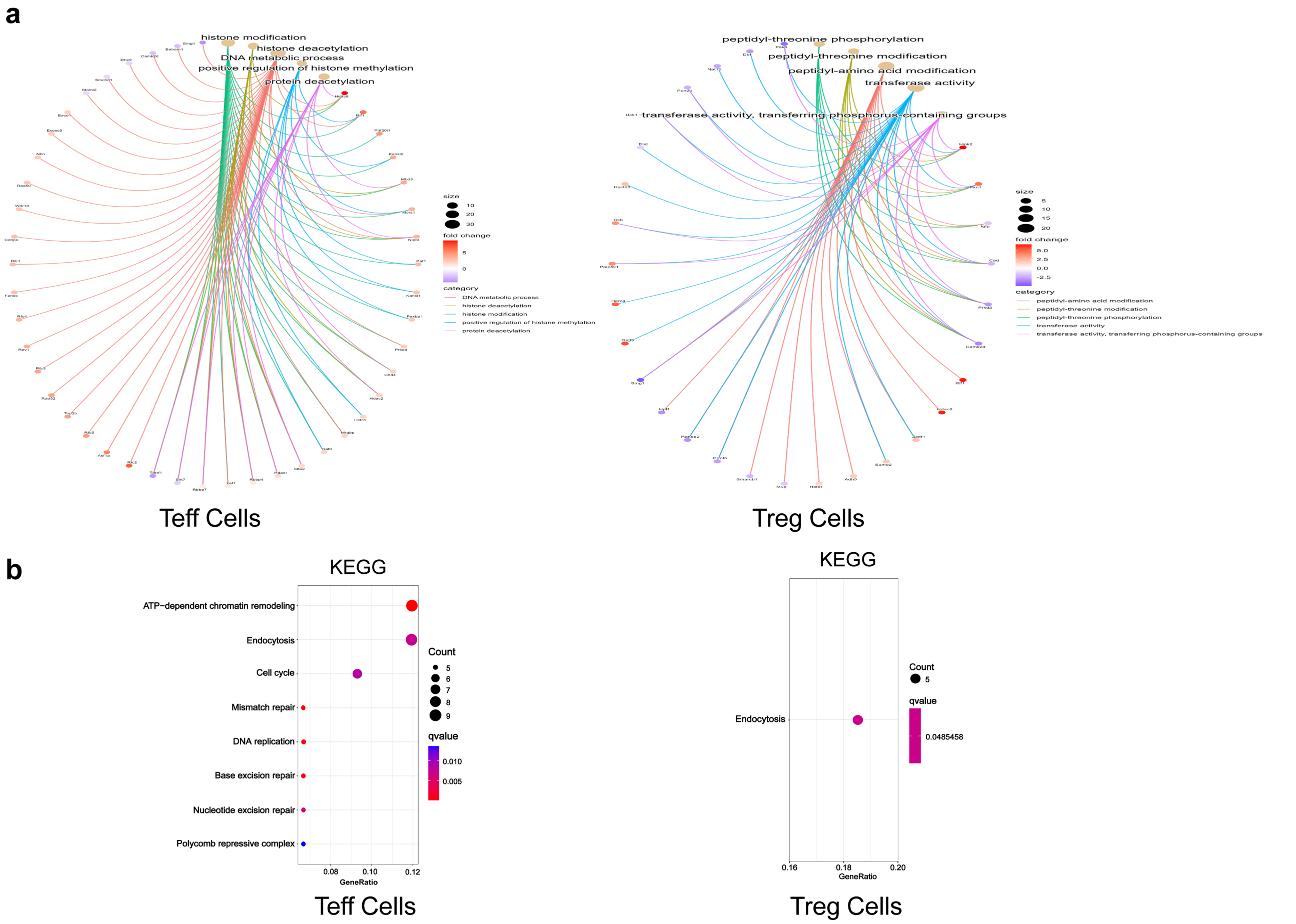


**Fig. S20**: Interaction analysis (a) and KEGG enrichment analysis (b) of HDAC8 co-immunoprecipitated proteins.

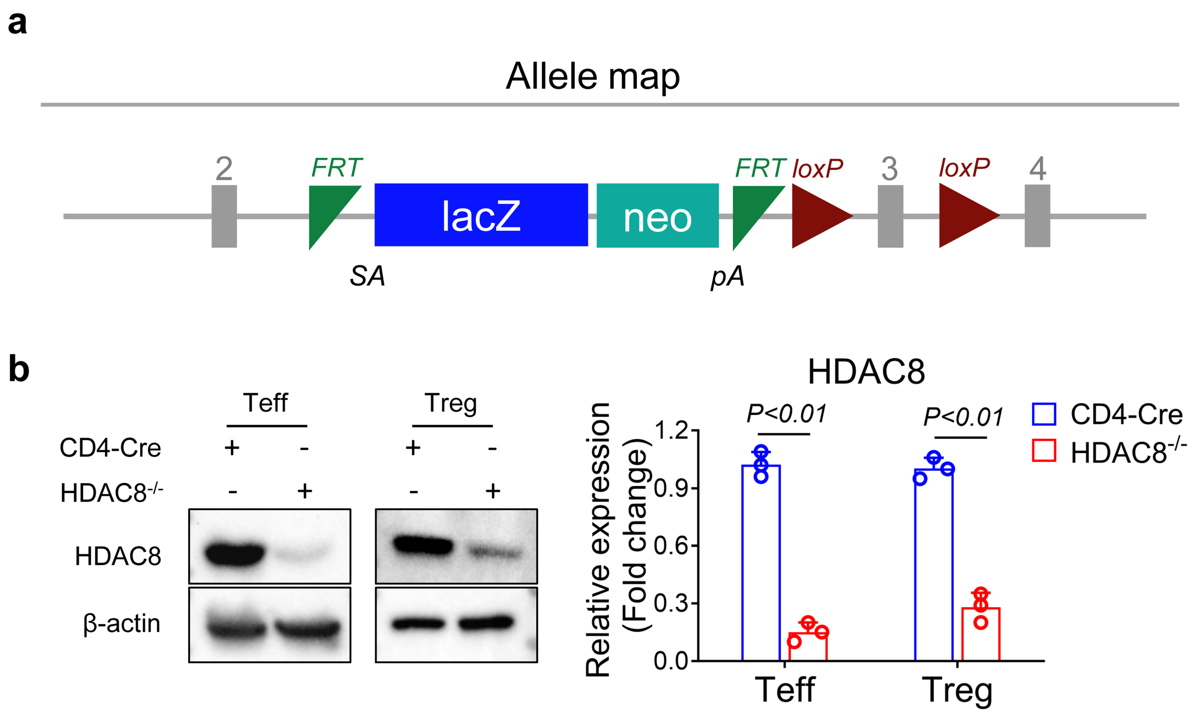


**Fig. S21**: (a) Allele map of HDAC8$^{fl/fl}$ mice. (b) Western blot analysis was used to detect the expression of HDAC8 in Teff and Treg cells. Assays were run in triplicate and repeated at least 3 times. The results of a representative experiment are shown. Data were expressed as the mean ±SD of three independent experiments. Statistical analysis: Comparisons between two groups utilized a two-tailed Student's t-test for normally distributed data.

## List of Supplementary Tables

**Table S1:** Methylation site and relative position of Foxp3 in Foxp3+ Treg and effector T Cells.

**Table S2:** Differentially expressed genes in Foxp3+ Treg cells after deletion of HDAC8.

**Table S3:** Differentially expressed genes in effector T Cells after deletion of HDAC8.

**Table S4:** Narrow peaks ChIPseeker annotation of HDAC8 in Teff cells (1).

**Table S5:** Narrow peaks ChIPseeker annotation of HDAC8 in Teff cells (2).

**Table S6:** Narrow peaks ChIPseeker annotation of HDAC8 in Teff cells (3).

**Table S7:** Narrow peaks ChIPseeker annotation of HDAC8 in Treg cells (1).

**Table S8:** Narrow peaks ChIPseeker annotation of HDAC8 in Treg cells (2).

**Table S9:** Narrow peaks ChIPseeker annotation of HDAC8 in Treg cells (3).

**Table S10:** Mass spectrometry analysis of HDAC8 in effector T cells.

**Table S11:** Mass spectrometry analysis of HDAC8 in Foxp3+ Treg cells.

**Table S12:** Antibodies used in this study.

**Table S13:** Number of cells for each cell subset per flow cytometry.

**Table S14:** Primers sequences used for real-time PCR and ChIP-PCR.

Table S1: Methylation site and relative position of Foxp3 in Foxp3+ Treg and Effector T Cells

| **Methylation site** | **CPG Position** | **Location** | **CD4-Cre (Teff)** | **DHAC8-/- (Teff)** | **CD4-Cre (Treg)** | **DHAC8-/- (Treg)** |
|---|---|---|---|---|---|---|
| 24WXX2021-FoxP3-18_CpG_1 | 79 | H_mass | NA | NA | NA | NA |
| 24WXX2021-FoxP3-18_CpG_2 | 149 | SN1 | 0.93 | 1 | 0.22 | 0.19 |
| 24WXX2021-FoxP3-18_CpG_3 | 211 | SN3 | 1 | 0.71 | 0 | 0.13 |
| 24WXX2021-FoxP3-18_CpG_4.5 | 219:223 | NA | 0.96 | 0.98 | 0.22 | 0.25 |
| 24WXX2021-FoxP3-18_CpG_6 | 242 | SN3 | 1 | 1 | 0.57 | 0.57 |
| 24WXX2021-FoxP3-18_CpG_7.8 | 291:296 | NA | 0.92 | 0.96 | 0.23 | 0.23 |
| 24WXX2021-FoxP3-18_CpG_9 | 323 | SN1 | 1 | 1 | 0.06 | 0 |
| 24WXX2021-FoxP3-18_CpG_10 | 357 | SN1 | 1 | 1 | NA | NA |
| 24WXX2021-FoxP3-18_CpG_11 | 373 | SN1 | 0.86 | 0.9 | 0.5 | 0.64 |
| 24WXX2021-FoxP3-18_CpG_12 | 409 | L_mass SN4 | NA | NA | NA | NA |

| Table S2: The differentially expressed genes in Foxp3+ Treg cells after deletion of HDAC8 | | | | | | |
|---|---|---|---|---|---|---|
| ID | HDAC8-KO Avg (log2) | CD4-Cre Avg (log2) | Fold Change(HDAC8-KO vs CD4-Cre) Tregs | P-val | FDR P-val | Gene Symbol |
| TC0900001592.mm.1 | 11.22 | 11.9 | -1.6 | 0.0001 | 0.0293 | Ccr8 |
| TC0700002007.mm.1 | 8.12 | 6.21 | 3.76 | 2.59E-06 | 0.0037 | Cd163l1 |
| TC1600000662.mm.1 | 9.29 | 9.98 | -1.61 | 6.05E-05 | 0.0212 | Cd200r1 |
| TC0300000693.mm.1 | 5.64 | 6.57 | -1.9 | 6.18E-06 | 0.0065 | Cd5l |
| TC0700000402.mm.1 | 10.24 | 9.29 | 1.93 | 1.46E-05 | 0.0096 | Cd79a |
| TC1600001744.mm.1 | 10.34 | 9.89 | 1.37 | 0.0002 | 0.0431 | Cep97 |
| TC0X00001934.mm.1 | 7.76 | 8.33 | -1.49 | 4.59E-05 | 0.0181 | Cfp |
| TC0300001704.mm.1 | 9.3 | 8.85 | 1.37 | 0.0002 | 0.0384 | Cpa3 |
| TC0100003856.mm.1 | 5.98 | 6.49 | -1.42 | 0.0003 | 0.0489 | Cr2 |
| TC1800000625.mm.1 | 6.11 | 7.19 | -2.11 | 3.73E-06 | 0.0044 | Csf1r |
| TC0500000850.mm.1 | 6.14 | 6.75 | -1.53 | 0.0001 | 0.0255 | Cxcl2 |
| TC0900002171.mm.1 | 11.12 | 10.24 | 1.85 | 1.41E-05 | 0.0096 | Cxcr5 |
| TC0500001894.mm.1 | 8.87 | 9.41 | -1.45 | 0.0002 | 0.0441 | Cyp51 |
| TC0X00002834.mm.1 | 7.27 | 6.55 | 1.65 | 7.14E-05 | 0.0212 | Cysltr1 |
| TC1400000975.mm.1 | 10.04 | 9.04 | 1.99 | 6.72E-06 | 0.0067 | Entpd4 |
| TC1900001095.mm.1 | 7.28 | 6.62 | 1.58 | 6.93E-05 | 0.0212 | Fads2 |
| TC0X00001789.mm.1 | 8.6 | 8.11 | 1.4 | 0.0002 | 0.035 | Gata1 |
| TC0300001447.mm.1 | 13.12 | 12.5 | 1.53 | 8.51E-05 | 0.0239 | Gbp2 |
| TC1400001012.mm.1 | 5.51 | 6.41 | -1.86 | 1.41E-05 | 0.0096 | Gfra2 |
| TC1700000625.mm.1 | 9.34 | 9.95 | -1.53 | 2.98E-05 | 0.0141 | H2-Ob |
| TC1300000262.mm.1 | 8.05 | 6.96 | 2.13 | 0.0003 | 0.0457 | Hist1h1a |
| TC0800000845.mm.1 | 6.9 | 8.08 | -2.26 | 2.67E-05 | 0.0132 | Hmox1 |
| TC1000000778.mm.1 | 10.79 | 10.14 | 1.57 | 2.77E-05 | 0.0134 | Icosl |
| TC1000001452.mm.1 | 9.55 | 10.17 | -1.54 | 0.0002 | 0.0405 | Ifng |
| TC1200002538.mm.1 | 8.17 | 5.86 | 4.97 | 2.24E-06 | 0.0036 | Ighe |
| TC0100000300.mm.1 | 9.16 | 9.92 | -1.69 | 0.0002 | 0.0391 | Il18rap |
| TC0200004602.mm.1 | 5.59 | 6.4 | -1.75 | 3.22E-05 | 0.015 | Il1b |
| TC0100000295.mm.1 | 7.82 | 6.81 | 2.02 | 0.0001 | 0.0268 | Il1r1 |
| TC0600002425.mm.1 | 9.31 | 8.43 | 1.84 | 1.66E-05 | 0.0105 | Il23r |
| TC0600002877.mm.1 | 6.76 | 6.2 | 1.47 | 7.21E-05 | 0.0212 | Il5ra |
| TC0800001599.mm.1 | 13.14 | 13.78 | -1.56 | 3.79E-05 | 0.0161 | Itgb1 |
| TC0500002712.mm.1 | 15.9 | 14.92 | 1.96 | 0.0001 | 0.0266 | Jchain |
| TC0600003062.mm.1 | 10.55 | 11.47 | -1.9 | 1.04E-05 | 0.0084 | Klrg1 |
| TC0600003246.mm.1 | 8.28 | 9.09 | -1.75 | 0.0001 | 0.0255 | Klri1 |
| TC0600003247.mm.1 | 7.85 | 8.5 | -1.58 | 0.0001 | 0.0255 | Klri2 |
| TC0400001697.mm.1 | 4.92 | 5.87 | -1.92 | 2.32E-05 | 0.0121 | LOC100862132 |
| TC1000002929.mm.1 | 6.78 | 7.98 | -2.3 | 0.0001 | 0.0256 | Lyz2 |
| TC0200001935.mm.1 | 5.91 | 6.61 | -1.63 | 7.87E-05 | 0.0226 | Mertk |
| TC0800000952.mm.1 | 8.78 | 8.23 | 1.46 | 0.0003 | 0.048 | Mir27a |
| TC0600002009.mm.1 | 9.16 | 8.49 | 1.6 | 0.0002 | 0.035 | Mir29a |
| TC0600003219.mm.1 | 8.37 | 7.42 | 1.93 | 1.28E-05 | 0.0093 | Mir7649 |
| TC0200000245.mm.1 | 6.04 | 6.77 | -1.67 | 6.53E-05 | 0.0212 | Mrc1 |
| TC0800001095.mm.1 | 9.89 | 9 | 1.86 | 2.18E-05 | 0.0119 | Mt1 |
| TC1800001176.mm.1 | 11.19 | 9.87 | 2.49 | 1.15E-06 | 0.0023 | Mzb1 |
| TC0100002743.mm.1 | 8.41 | 7.42 | 1.98 | 8.53E-06 | 0.0076 | Nmur1 |
| TC0400002139.mm.1 | 9.02 | 10.08 | -2.09 | 0.0001 | 0.0279 | Penk |
| TC0600003329.mm.1 | 8 | 8.68 | -1.6 | 0.0002 | 0.0366 | Plbd1 |
| TC1900000472.mm.1 | 9.6 | 10.04 | -1.36 | 0.0003 | 0.0464 | Pten |
| TC0100002413.mm.1 | 7.68 | 8.36 | -1.6 | 2.64E-05 | 0.0131 | Raph1 |
| TC0300000864.mm.1 | 8.54 | 7.57 | 1.95 | 7.01E-05 | 0.0212 | Rorc |
| TC0400004175.mm.1 | 6.06 | 6.93 | -1.82 | 2.71E-05 | 0.0132 | Samd11 |
| TC0300002465.mm.1 | 9.86 | 9.15 | 1.64 | 0.0001 | 0.0255 | Scnm1 |
| TC0200001959.mm.1 | 7.1 | 7.81 | -1.65 | 3.53E-05 | 0.0159 | Sirpa |
| TC1600001582.mm.1 | 9.04 | 8.35 | 1.61 | 1.90E-05 | 0.0111 | Slc15a2 |
| TC0100002285.mm.1 | 6.17 | 6.99 | -1.77 | 7.69E-05 | 0.0223 | Slc40a1 |
| TC0200005112.mm.1 | 8.05 | 7.13 | 1.89 | 1.18E-05 | 0.009 | Slpi; Mir7678 |
| TC1500002177.mm.1 | 9.04 | 9.63 | -1.51 | 0.0001 | 0.0279 | Snora34; Mir1291 |
| TC1100001783.mm.1 | 14.36 | 15.13 | -1.71 | 2.47E-05 | 0.0125 | Snord104 |
| TSUnmapped00000178. | 13.63 | 14.18 | -1.46 | 0.0002 | 0.0385 | Snord14e |
| TC1100004150.mm.1 | 8.65 | 7.98 | 1.59 | 3.33E-05 | 0.0153 | Socs3 |
| TC1000002647.mm.1 | 5.88 | 7.39 | -2.86 | 2.01E-07 | 0.0008 | Spic |
| TC0200005164.mm.1 | 7.74 | 8.32 | -1.5 | 0.0002 | 0.0391 | Sulf2 |
| TC1300000144.mm.1 | 14.81 | 13.76 | 2.07 | 6.90E-05 | 0.0212 | Tcrg-C3 |
| TC1300000145.mm.1 | 11.97 | 12.73 | -1.69 | 1.14E-05 | 0.0089 | Tcrg-C4; Trgj4 |

| | | | | | | |
|---|---|---|---|---|---|---|
| TC1300000138.mm.1 | 4.69 | 5.67 | -1.98 | 3.85E-05 | 0.0161 | Tcrg-V7 |
| TC0300000889.mm.1 | 6.13 | 7.01 | -1.84 | 6.37E-06 | 0.0066 | Tmod4 |
| TC1600000137.mm.1 | 9.13 | 8.13 | 2.01 | 3.78E-05 | 0.0161 | Tnfrsf17 |
| TC1100003694.mm.1 | 6.33 | 5.8 | 1.45 | 0.0002 | 0.0417 | Tns4 |
| TC0100002038.mm.1 | 12 | 11.12 | 1.85 | 9.82E-06 | 0.0083 | Tram2 |
| TC1400000632.mm.1 | 11.6 | 12.41 | -1.75 | 0.0001 | 0.0255 | Trav11d |
| TC1400002800.mm.1 | 11.43 | 11.9 | -1.38 | 0.0003 | 0.048 | Trav9d-2 |
| TC1400000644.mm.1 | 10.5 | 10.98 | -1.4 | 0.0002 | 0.0359 | Trav9d-3 |
| TC1400000715.mm.1 | 8.7 | 7.41 | 2.46 | 7.19E-05 | 0.0212 | Trdv4 |
| TC1300002762.mm.1 | 14.57 | 13.27 | 2.47 | 1.10E-06 | 0.0023 | Trgj1; Tcrg-C1 |
| TC1300001835.mm.1 | 13.09 | 12.22 | 1.83 | 5.73E-06 | 0.0062 | Txndc5 |
| TC0300002802.mm.1 | 7.33 | 8.27 | -1.92 | 1.05E-05 | 0.0084 | Vcam1 |
| TC1100000072.mm.1 | 13.36 | 12.72 | 1.56 | 8.60E-05 | 0.024 | Xbp1 |
| TC0X00000676.mm.1 | 6.77 | 7.62 | -1.8 | 1.16E-05 | 0.0089 | Xlr3b |
| TC0X00000723.mm.1 | 6.35 | 7 | -1.57 | 0.0001 | 0.0304 | Xlr3d-ps |
| TC1600001838.mm.1 | 10.59 | 11.21 | -1.53 | 0.0001 | 0.0304 | Zfp654 |

| Table S3: The differentially expressed genes in effector T Cells after deletion of HDAC8 | | | | | | |
|---|---|---|---|---|---|---|
| ID | HDAC8-KO Avg (log2) | CD4-Cre Avg (log2) | Fold Change(HDAC8-KO vs CD4-Cre) Teff | P-val | FDR P-val | Gene Symbol |
| TC1100000251.mm.1 | 6.66 | 7.06 | -1.32 | 0.0001 | 0.0032 | Bcl11a |
| TC0100002894.mm.1 | 12.41 | 12.65 | -1.19 | 0.0023 | 0.029 | Bcl2 |
| TC0200001927.mm.1 | 10.87 | 11.16 | -1.22 | 0.0026 | 0.0314 | Bcl2l11 |
| TC0900003324.mm.1 | 10.14 | 9.77 | 1.29 | 0.0018 | 0.0241 | Bcl9l |
| TC0300003235.mm.1 | 5.8 | 6.08 | -1.22 | 0.0041 | 0.0443 | Bglap3 |
| TC0600001203.mm.1 | 10.85 | 11.11 | -1.19 | 0.0024 | 0.0297 | Bhlhe40 |
| TC0600003428.mm.1 | 7.06 | 7.41 | -1.27 | 0.003 | 0.0346 | Bhlhe41 |
| TC1300002437.mm.1 | 5.36 | 5.65 | -1.23 | 0.0018 | 0.0241 | Bhmt2 |
| TC1400002219.mm.1 | 8.01 | 8.51 | -1.41 | 6.60E-05 | 0.0017 | Blk |
| TC1900001498.mm.1 | 7.79 | 8.2 | -1.33 | 0.0005 | 0.0081 | Blnk |
| TC1700000420.mm.1 | 7.86 | 7.67 | 1.14 | 0.0046 | 0.0478 | Bnip1 |
| TC0X00002994.mm.1 | 7.02 | 7.39 | -1.3 | 0.001 | 0.0147 | Btk |
| TC0400002505.mm.1 | 5.12 | 5.87 | -1.68 | 3.42E-05 | 0.001 | Ccl19 |
| TC4_JH584293_random | 5.52 | 6.12 | -1.51 | 0.0001 | 0.0025 | Ccl19 |
| TC4_JH584294_random | 5.96 | 6.45 | -1.41 | 0.0003 | 0.0063 | Ccl19; Gm2023 |
| TC0400000395.mm.1 | 5.99 | 6.64 | -1.57 | 1.26E-05 | 0.0005 | Ccl19; Gm2457 |
| TC0400000412.mm.1 | 5.93 | 6.8 | -1.82 | 1.05E-05 | 0.0004 | Ccl19; Gm2564 |
| TC0400000389.mm.1 | 5.8 | 6.34 | -1.45 | 0.0002 | 0.0035 | Ccl19-ps1 |
| TC1100003396.mm.1 | 14.67 | 15.32 | -1.57 | 3.79E-05 | 0.0011 | Ccl5 |
| TC0700004610.mm.1 | 6.62 | 6.86 | -1.18 | 0.0022 | 0.0281 | Ccnd1; Mir3962 |
| TC0900003169.mm.1 | 8.81 | 8.29 | 1.44 | 0.0032 | 0.0365 | Ccr4 |
| TC1100003696.mm.1 | 15.77 | 15.32 | 1.36 | 0.0007 | 0.0109 | Ccr7 |
| TC0900001670.mm.1 | 7.8 | 8.27 | -1.38 | 7.80E-05 | 0.002 | Ccr9 |
| TC0300003241.mm.1 | 8.78 | 9.61 | -1.78 | 2.96E-05 | 0.0009 | Cd160 |
| TC1300001155.mm.1 | 9.18 | 9.95 | -1.71 | 3.69E-06 | 0.0002 | Cd180 |
| TC0700004277.mm.1 | 8.31 | 9.26 | -1.93 | 1.27E-05 | 0.0005 | Cd19 |
| TC1600000662.mm.1 | 8.43 | 9.14 | -1.64 | 3.87E-05 | 0.0011 | Cd200r1 |
| TC0800001625.mm.1 | 6.29 | 7.08 | -1.73 | 0.0003 | 0.0064 | Cd209c |
| TC0700002779.mm.1 | 7.7 | 7.93 | -1.18 | 0.0045 | 0.0475 | Cd22 |
| TC1800000909.mm.1 | 12.2 | 12.46 | -1.2 | 0.0016 | 0.0217 | Cd226 |
| TC1000000402.mm.1 | 10.26 | 10.78 | -1.43 | 0.0036 | 0.0402 | Cd24a |
| TC0600003140.mm.1 | 14.41 | 14.05 | 1.28 | 0.0005 | 0.0084 | Cd27; Mir8113 |
| TC1100004052.mm.1 | 6.48 | 6.78 | -1.23 | 0.0033 | 0.0372 | Cd300c |
| TC1100004060.mm.1 | 7.01 | 7.39 | -1.31 | 0.0008 | 0.0122 | Cd300lf |
| TC0700002950.mm.1 | 6.63 | 6.87 | -1.18 | 0.0039 | 0.0422 | Cd33 |
| TC0500000468.mm.1 | 8.58 | 9.5 | -1.89 | 4.41E-06 | 0.0002 | Cd38 |
| TC0600003124.mm.1 | 16.92 | 16.69 | 1.17 | 0.0018 | 0.0236 | Cd4 |
| TC0200002544.mm.1 | 6.24 | 6.49 | -1.18 | 0.0047 | 0.0487 | Cd40 |
| TC0X00000533.mm.1 | 12.91 | 12.49 | 1.34 | 0.0001 | 0.0032 | Cd40lg |
| TC1900001113.mm.1 | 15.89 | 15.53 | 1.29 | 0.0003 | 0.0057 | Cd5 |
| TC0100003037.mm.1 | 13.43 | 13.16 | 1.21 | 0.0014 | 0.0196 | Cd55 |
| TC0200001551.mm.1 | 6.08 | 6.5 | -1.34 | 0.0002 | 0.0036 | Cd59b |
| TC1100004240.mm.1 | 10.46 | 11.37 | -1.88 | 3.96E-05 | 0.0012 | Cd7 |
| TC0400002523.mm.1 | 9.15 | 9.69 | -1.45 | 2.28E-05 | 0.0007 | Cd72 |
| TC1800000617.mm.1 | 15.2 | 15.95 | -1.68 | 7.28E-06 | 0.0003 | Cd74; Mir5107 |
| TC0700000402.mm.1 | 8.82 | 9.37 | -1.46 | 0.0004 | 0.0067 | Cd79a |
| TC1100003920.mm.1 | 9.64 | 10.23 | -1.51 | 5.11E-05 | 0.0014 | Cd79b |
| TC1600000553.mm.1 | 7.28 | 7.75 | -1.38 | 0.001 | 0.0155 | Cd80 |
| TSUnmapped00000047. | 7.28 | 7.93 | -1.58 | 0.0003 | 0.0053 | Cd83 |
| TC0600003148.mm.1 | 9.89 | 9.6 | 1.22 | 0.0012 | 0.0173 | Cd9 |
| TC1100003037.mm.1 | 14.04 | 13.71 | 1.26 | 0.0004 | 0.008 | Chd3 |
| TC0300002684.mm.1 | 5.18 | 5.65 | -1.39 | 0.0005 | 0.0087 | Chil3 |
| TC0300002685.mm.1 | 4.84 | 5.16 | -1.25 | 0.0035 | 0.0391 | Chil4 |
| TC0200001441.mm.1 | 5.67 | 6.2 | -1.44 | 0.003 | 0.0346 | Chst1 |
| TC0700000991.mm.1 | 11.82 | 12.49 | -1.59 | 1.56E-05 | 0.0006 | Chsy1 |
| TC1600000116.mm.1 | 6.84 | 7.32 | -1.4 | 0.0006 | 0.0102 | Ciita |
| TC0900001365.mm.1 | 7.6 | 8.01 | -1.33 | 0.0008 | 0.0134 | Cish |
| TC0300003080.mm.1 | 4.99 | 5.25 | -1.19 | 0.0042 | 0.0447 | Clca3b |
| TC1400001288.mm.1 | 7.39 | 6.84 | 1.47 | 0.0001 | 0.003 | Cldn10 |
| TC0600001521.mm.1 | 12.06 | 12.31 | -1.19 | 0.0031 | 0.0355 | Clec2i |
| TC0600001401.mm.1 | 5.59 | 5.88 | -1.22 | 0.0045 | 0.0473 | Clec4a2 |
| TC0600001403.mm.1 | 5.75 | 6.22 | -1.39 | 0.0003 | 0.0064 | Clec4n |
| TC0600003537.mm.1 | 6.66 | 7.27 | -1.53 | 0.0002 | 0.0038 | Clec9a |
| TC0500003238.mm.1 | 11.62 | 11.39 | 1.17 | 0.0039 | 0.0429 | Clip1 |
| TC1400001331.mm.1 | 8.84 | 9.19 | -1.27 | 0.0013 | 0.0189 | Clybl |
| TC0400002603.mm.1 | 8.62 | 9.04 | -1.34 | 0.0002 | 0.0034 | Coro2a |
| TC0100003029.mm.1 | 13.51 | 13.9 | -1.31 | 0.0017 | 0.0228 | Cxcr4 |
| TC0900001672.mm.1 | 8.67 | 9.3 | -1.55 | 0.0002 | 0.0048 | Cxcr6 |
| TC0900001416.mm.1 | 10.81 | 10.47 | 1.27 | 0.0022 | 0.0281 | Dalrd3 |
| TC0200000894.mm.1 | 12.5 | 11.38 | 2.17 | 5.51E-07 | 4.61E-05 | Dapl1 |
| TC0800002569.mm.1 | 10.3 | 10.03 | 1.2 | 0.0018 | 0.024 | Dcaf15 |
| TC1400000304.mm.1 | 10.49 | 10.28 | 1.16 | 0.0037 | 0.041 | Dcp1a |

| | | | | | | |
|---|---|---|---|---|---|---|
| TC1000002224.mm.1 | 11.85 | 12.22 | -1.3 | 0.0037 | 0.0406 | Ddit4 |
| TC0800000211.mm.1 | 5.41 | 6.28 | -1.83 | 4.90E-06 | 0.0002 | Defa21 |
| TC0800000220.mm.1 | 5.5 | 6.31 | -1.76 | 4.74E-05 | 0.0013 | Defa22 |
| TC1000003161.mm.1 | 17.57 | 17.32 | 1.19 | 0.0015 | 0.0201 | Dgka |
| TC0800002904.mm.1 | 10.01 | 9.74 | 1.21 | 0.0015 | 0.0206 | Dhx38 |
| TC0X00000735.mm.1 | 11.01 | 11.25 | -1.19 | 0.0028 | 0.0332 | Dkc1 |
| TC1400001432.mm.1 | 7.27 | 8.15 | -1.83 | 0.0001 | 0.0029 | Dnase1l3 |
| TC1900001547.mm.1 | 9.29 | 9.02 | 1.21 | 0.0047 | 0.0486 | Dnmbp |
| TC0300001245.mm.1 | 10.43 | 10.14 | 1.22 | 0.0008 | 0.0132 | Dnttip2 |
| TC1300002062.mm.1 | 7.71 | 8.15 | -1.36 | 0.0003 | 0.0057 | Dok3 |
| TC1600002182.mm.1 | 11.01 | 10.78 | 1.17 | 0.0028 | 0.0336 | Donson |
| TC0900002720.mm.1 | 6.51 | 6.87 | -1.28 | 0.0014 | 0.019 | Dppa5a |
| TC1700001067.mm.1 | 5.08 | 4.54 | 1.45 | 0.0018 | 0.0236 | Dreh |
| TC0X00001888.mm.1 | 17.43 | 17.83 | -1.32 | 0.0005 | 0.0095 | Drr1 |
| TC0100002921.mm.1 | 7.83 | 7.5 | 1.26 | 0.0039 | 0.0429 | Dse1 |
| TC0600002629.mm.1 | 13.35 | 13.07 | 1.21 | 0.0009 | 0.0139 | Dusp11 |
| TC0200001913.mm.1 | 12.61 | 12.91 | -1.23 | 0.0024 | 0.0296 | Dusp2 |
| TC1000002199.mm.1 | 5.06 | 5.79 | -1.66 | 5.88E-05 | 0.0016 | Dux |
| TC1400001872.mm.1 | 5.41 | 5.81 | -1.31 | 0.0005 | 0.0083 | Ear10 |
| TC1400000459.mm.1 | 5.19 | 5.61 | -1.33 | 0.0003 | 0.0057 | Ear12; Ear2; Ear3 |
| TC0700000787.mm.1 | 5.53 | 5.92 | -1.31 | 0.0005 | 0.0088 | Egfbp2; Klk1b26 |
| TC1000000934.mm.1 | 6.85 | 6.4 | 1.36 | 0.0016 | 0.0213 | Eid3 |
| TC1200000829.mm.1 | 9.45 | 9.18 | 1.2 | 0.0013 | 0.0179 | Eif2b2 |
| TC0600001606.mm.1 | 8.95 | 8 | 1.92 | 7.91E-06 | 0.0003 | Emp1 |
| TC0200005472.mm.1 | 6.82 | 7.39 | -1.48 | 9.10E-05 | 0.0022 | Eng |
| TC1400000975.mm.1 | 10.15 | 9.19 | 1.95 | 2.04E-06 | 0.0001 | Entpd4 |
| TC1400000980.mm.1 | 10.58 | 9.66 | 1.89 | 1.24E-06 | 8.45E-05 | Entpd4; Gm21685 |
| TC1700001299.mm.1 | 8.14 | 7.47 | 1.59 | 1.05E-05 | 0.0004 | Epas1 |
| TC0600000444.mm.1 | 7.95 | 8.23 | -1.21 | 0.0029 | 0.0339 | Ephb6 |
| TC0700000033.mm.1 | 8.33 | 7.91 | 1.34 | 0.0007 | 0.0109 | Eps8l1 |
| TC1400001086.mm.1 | 12.75 | 12.97 | -1.16 | 0.0047 | 0.0489 | Epsti1 |
| TC0600003020.mm.1 | 8.72 | 8.43 | 1.23 | 0.0013 | 0.018 | Erc1 |
| TC0200003573.mm.1 | 4.18 | 4.42 | -1.18 | 0.0036 | 0.0399 | Ermn |
| TC1700000815.mm.1 | 5.18 | 5.66 | -1.39 | 0.0047 | 0.0487 | Esp3 |
| TC1700002092.mm.1 | 4.85 | 5.3 | -1.36 | 0.0004 | 0.0071 | Esp36 |
| TC1300002462.mm.1 | 9.13 | 9.57 | -1.36 | 0.0003 | 0.0065 | F2r |
| TC1300002460.mm.1 | 8.76 | 8.17 | 1.5 | 5.80E-05 | 0.0016 | F2rl1 |
| TC0600003456.mm.1 | 8.63 | 10.17 | -2.92 | 8.46E-07 | 6.31E-05 | Far2os1; RP23-285C21.5 |
| TC1100002077.mm.1 | 6.58 | 7.19 | -1.52 | 0.0004 | 0.0077 | Fau-ps2 |
| TC0100001166.mm.1 | 9.26 | 10.41 | -2.21 | 9.52E-07 | 6.88E-05 | Fcmr |
| TC0300000694.mm.1 | 6.73 | 7.06 | -1.25 | 0.0006 | 0.0098 | Fcrl1 |
| TC0300000695.mm.1 | 7.71 | 8.34 | -1.54 | 8.74E-05 | 0.0022 | Fcrl5 |
| TC0100003520.mm.1 | 7.53 | 7.95 | -1.34 | 0.0004 | 0.0067 | Fcrla |
| TC0300000843.mm.1 | 5.62 | 6.38 | -1.69 | 1.77E-05 | 0.0006 | Flg2 |
| TC0500003626.mm.1 | 7.31 | 7.52 | -1.16 | 0.0047 | 0.0487 | Flt3 |
| TC1200002523.mm.1 | 8.69 | 8.29 | 1.32 | 0.0025 | 0.0303 | Fntb |
| TC0600001393.mm.1 | 10.2 | 9.93 | 1.21 | 0.0028 | 0.0336 | Foxj2; Mir7231 |
| TC1700000214.mm.1 | 5.13 | 5.47 | -1.26 | 0.0009 | 0.0141 | Fpr2; Fpr3 |
| TC1200000688.mm.1 | 9.92 | 10.15 | -1.17 | 0.0049 | 0.0499 | Fut8 |
| TC1300002654.mm.1 | 5.76 | 6.37 | -1.52 | 0.0026 | 0.031 | Gapt |
| TC1700002637.mm.1 | 7.4 | 7.81 | -1.33 | 0.002 | 0.0261 | Haao |
| TC1100000336.mm.1 | 6.27 | 7.43 | -2.24 | 8.49E-06 | 0.0003 | Hba-a2; Hba-a1 |
| TC0700003909.mm.1 | 6.39 | 6.93 | -1.46 | 0.0008 | 0.0133 | Hbb-bs; Hbb-b1 |
| TC0700003908.mm.1 | 6.4 | 7.17 | -1.7 | 1.13E-05 | 0.0004 | Hbb-bt; Hbb-b2; Hbb-b1 |
| TC0500003243.mm.1 | 6.11 | 6.76 | -1.57 | 0.0038 | 0.042 | Hcar2 |
| TC0200002308.mm.1 | 7.05 | 7.46 | -1.33 | 0.0005 | 0.0083 | Hck |
| TC0X00002741.mm.1 | 8.94 | 9.26 | -1.25 | 0.0015 | 0.0207 | Hdac8 |
| TC0700003622.mm.1 | 7.89 | 7.52 | 1.29 | 0.0017 | 0.0231 | Hdgfrp3 |
| TC1100003395.mm.1 | 8.49 | 8.87 | -1.31 | 0.0007 | 0.0118 | Heatr9 |
| TC1300001353.mm.1 | 10.57 | 10.87 | -1.23 | 0.0044 | 0.0468 | Hmgcs1 |
| TC1000000836.mm.1 | 14.86 | 14.62 | 1.18 | 0.002 | 0.0257 | Hmha1 |
| TC0600000625.mm.1 | 4.95 | 5.27 | -1.25 | 0.003 | 0.0352 | Hottip |
| TC0500002843.mm.1 | 6.5 | 6.76 | -1.2 | 0.0013 | 0.0179 | Hpse |
| TC0300002553.mm.1 | 5.39 | 5.74 | -1.28 | 0.0006 | 0.0108 | Hsd3b4; Gm10681 |
| TC1700001936.mm.1 | 7.12 | 8 | -1.84 | 5.90E-06 | 0.0003 | Hspa1a |
| TC1700001935.mm.1 | 7.9 | 8.57 | -1.59 | 0.001 | 0.0152 | Hspa1b; Hspa1a |
| TC0300002680.mm.1 | 7.83 | 8.27 | -1.36 | 9.97E-05 | 0.0024 | I830077J02Rik |
| TC0900000184.mm.1 | 8.49 | 8.79 | -1.23 | 0.0007 | 0.012 | Icam1 |
| TC0100000490.mm.1 | 12.94 | 12.71 | 1.18 | 0.0044 | 0.0464 | Icos |
| TC1000000778.mm.1 | 10.05 | 9.45 | 1.51 | 9.49E-05 | 0.0023 | Icosl |
| TC1200001544.mm.1 | 14.52 | 14.91 | -1.31 | 0.0019 | 0.0244 | Id2 |
| TC0700003581.mm.1 | 10.13 | 9.7 | 1.35 | 0.0001 | 0.0025 | Idh2 |
| TC1200002269.mm.1 | 10.57 | 10.16 | 1.33 | 0.0015 | 0.0201 | Ifi27l2a |
| TC0800002403.mm.1 | 11.7 | 12.02 | -1.25 | 0.0005 | 0.0093 | Ifi30 |
| TC1900001422.mm.1 | 11.77 | 11.1 | 1.6 | 0.0006 | 0.0106 | Ifit1bl1 |

| | | | | | | |
|---|---|---|---|---|---|---|
| TC1900001423.mm.1 | 6.72 | 6.3 | 1.34 | 0.0004 | 0.008 | Ifit1bl2 |
| TC1900000501.mm.1 | 9.38 | 9 | 1.3 | 0.0036 | 0.04 | Ifit3 |
| TC1900000502.mm.1 | 7.77 | 6.95 | 1.76 | 0.0005 | 0.0087 | Ifit3b |
| TC1000001452.mm.1 | 10.36 | 11.07 | -1.64 | 4.81E-06 | 0.0002 | Ifng |
| TC0400000895.mm.1 | 5.86 | 6.28 | -1.33 | 0.0008 | 0.0122 | Ifnz |
| TC0300002122.mm.1 | 11.19 | 10.9 | 1.22 | 0.0007 | 0.0113 | Ift80 |
| TC0700001008.mm.1 | 8.6 | 8.96 | -1.28 | 0.001 | 0.0149 | Igf1r |
| TC1200002462.mm.1 | 4.58 | 5.21 | -1.55 | 0.0035 | 0.0392 | Ighd5-7 |
| TC1200002467.mm.1 | 4.58 | 5.21 | -1.55 | 0.0035 | 0.0392 | Ighd6-1 |
| TC1200002540.mm.1 | 9.18 | 8.31 | 1.83 | 9.39E-07 | 6.82E-05 | Ighg1 |
| TC1200002539.mm.1 | 15.78 | 15.08 | 1.63 | 1.11E-05 | 0.0004 | Ighg2b |
| TC1200002541.mm.1 | 7.44 | 7.88 | -1.36 | 8.01E-05 | 0.002 | Ighg3 |
| TC1200002542.mm.1 | 16.56 | 17.34 | -1.71 | 3.14E-06 | 0.0002 | Ighm |
| TC0700004207.mm.1 | 8.32 | 8.68 | -1.28 | 0.0021 | 0.0264 | Igsf6 |
| TC1100004265.mm.1 | 13.31 | 12.96 | 1.27 | 0.0026 | 0.0317 | Igtp |
| TC1800000610.mm.1 | 8.81 | 8.08 | 1.66 | 1.15E-05 | 0.0004 | Iigp1 |
| TC0800001874.mm.1 | 12.43 | 12.17 | 1.19 | 0.0029 | 0.0338 | Ikbkb |
| TC0100003057.mm.1 | 12.81 | 12.5 | 1.25 | 0.0008 | 0.0127 | Ikbke |
| TC0100002534.mm.1 | 9.96 | 10.67 | -1.64 | 6.35E-06 | 0.0003 | Ikzf2 |
| TC0100001167.mm.1 | 6.07 | 6.53 | -1.37 | 0.0025 | 0.0307 | Il10 |
| TC0100000299.mm.1 | 11.85 | 12.11 | -1.2 | 0.0014 | 0.0192 | Il18r1 |
| TC0100000300.mm.1 | 10.24 | 10.75 | -1.43 | 6.93E-05 | 0.0018 | Il18rap |
| TC0200004602.mm.1 | 5.52 | 5.94 | -1.34 | 0.0046 | 0.0478 | Il1b |
| TC0100000297.mm.1 | 10.18 | 9.89 | 1.23 | 0.0032 | 0.0363 | Il1rl2 |
| TC0700001794.mm.1 | 12.47 | 12.12 | 1.28 | 0.001 | 0.0146 | Il21r |
| TC0600002425.mm.1 | 6.41 | 5.98 | 1.35 | 0.0009 | 0.0135 | Il23r |
| TC1500001833.mm.1 | 14.7 | 15.26 | -1.47 | 4.17E-05 | 0.0012 | Il2rb |
| TC0700001793.mm.1 | 14.32 | 14.09 | 1.18 | 0.002 | 0.0254 | Il4ra |
| TC0600002877.mm.1 | 6.67 | 6.95 | -1.21 | 0.0038 | 0.0413 | Il5ra |
| TC1300001265.mm.1 | 14.7 | 14.25 | 1.37 | 8.91E-05 | 0.0022 | Il6st |
| TC0100000222.mm.1 | 11.5 | 11.21 | 1.22 | 0.0047 | 0.0489 | Imp4 |
| TC0400000982.mm.1 | 10.03 | 9.63 | 1.32 | 0.0002 | 0.0043 | Inadl |
| TC0100000946.mm.1 | 9.68 | 9.44 | 1.18 | 0.003 | 0.0346 | Ing5 |
| TC0700001888.mm.1 | 9.85 | 9.51 | 1.26 | 0.0017 | 0.0224 | Inpp5f |
| TC1000001691.mm.1 | 13.38 | 13.04 | 1.26 | 0.0008 | 0.0128 | Ipcef1 |
| TC1300002463.mm.1 | 12.53 | 12.88 | -1.27 | 0.0005 | 0.0086 | Iqgap2 |
| TC0700000825.mm.1 | 10.02 | 9.81 | 1.16 | 0.0043 | 0.0455 | Irf3 |
| TC1300000338.mm.1 | 10.39 | 10.12 | 1.2 | 0.0017 | 0.0226 | Irf4 |
| TC0700004530.mm.1 | 10.74 | 10.5 | 1.18 | 0.0023 | 0.0283 | Irf7 |
| TC0800000756.mm.1 | 10.91 | 10.64 | 1.2 | 0.0044 | 0.0465 | Isyna1 |
| TC1500002039.mm.1 | 11.32 | 10.8 | 1.43 | 0.0002 | 0.0035 | Mapk11 |
| TC1300000972.mm.1 | 8.06 | 8.45 | -1.31 | 0.0003 | 0.0054 | Mef2c |
| TC0700001739.mm.1 | 10.82 | 10.49 | 1.25 | 0.0016 | 0.0219 | Mettl9 |
| TC0700001192.mm.1 | 8.92 | 8.54 | 1.29 | 0.0046 | 0.048 | Mex3b |
| TC1100000874.mm.1 | 4.15 | 3.91 | 1.19 | 0.0021 | 0.0268 | Mfsd6l |
| TC1500000666.mm.1 | 9.25 | 9.02 | 1.17 | 0.0033 | 0.0377 | Micall1 |
| TC1300001941.mm.1 | 10.49 | 10.23 | 1.19 | 0.0049 | 0.0499 | Mirlet7d |
| TC0700004451.mm.1 | 11.78 | 12.1 | -1.25 | 0.0008 | 0.0127 | Mki67 |
| TC0900000046.mm.1 | 5.72 | 6.05 | -1.25 | 0.0034 | 0.0384 | Mmp12 |
| TC1700001596.mm.1 | 5.99 | 5.63 | 1.28 | 0.0023 | 0.0283 | Mmp25 |
| TC1900000246.mm.1 | 8.95 | 9.61 | -1.59 | 1.32E-05 | 0.0005 | Mpeg1 |
| TC1100001353.mm.1 | 9.53 | 9.8 | -1.2 | 0.0019 | 0.0244 | Mpo |
| TC0X00002470.mm.1 | 10.35 | 10.01 | 1.27 | 0.0018 | 0.024 | Mpp1 |
| TC0700003060.mm.1 | 6.02 | 6.53 | -1.42 | 0.0018 | 0.0234 | Mrgpra4 |
| TC0700002401.mm.1 | 10.99 | 10.71 | 1.21 | 0.0013 | 0.0188 | Mrip-ps |
| TC0100000845.mm.1 | 5.79 | 5.99 | -1.15 | 0.0045 | 0.0475 | Mroh2a |
| TC1900001128.mm.1 | 8.6 | 9.59 | -1.97 | 1.13E-06 | 7.83E-05 | Ms4a1 |
| TC1900001137.mm.1 | 7.14 | 7.75 | -1.52 | 0.0003 | 0.0052 | Ms4a6d |
| TC1000000492.mm.1 | 8.21 | 7.76 | 1.36 | 0.0026 | 0.0309 | Msl3l2 |
| TC0500003618.mm.1 | 8.41 | 7.95 | 1.38 | 0.0009 | 0.0142 | Mtif3 |
| TC0600002641.mm.1 | 10.66 | 11.01 | -1.27 | 0.0005 | 0.0093 | Mxd1 |
| TC1000001840.mm.1 | 12.15 | 11.59 | 1.47 | 2.46E-05 | 0.0008 | Myb |
| TC1500000453.mm.1 | 10.43 | 10.83 | -1.32 | 0.0002 | 0.004 | Myc |
| TC0900000927.mm.1 | 8.83 | 9.56 | -1.67 | 5.89E-06 | 0.0003 | Myo1e |
| TC1700000585.mm.1 | 11.03 | 11.28 | -1.19 | 0.0017 | 0.0224 | Myo1f |
| TC1800001176.mm.1 | 9.42 | 9.67 | -1.19 | 0.0028 | 0.0328 | Mzb1 |
| TC1600001255.mm.1 | 6.41 | 6.18 | 1.18 | 0.0025 | 0.0302 | Mzt2 |
| TC0500000623.mm.1 | 9.63 | 9.95 | -1.25 | 0.0011 | 0.0162 | N4bp2 |
| TC0500003685.mm.1 | 10.41 | 10.12 | 1.22 | 0.0013 | 0.0183 | N4bp2l1 |
| TC0700000810.mm.1 | 8.09 | 8.65 | -1.47 | 3.85E-05 | 0.0011 | Napsa |
| TC0800000694.mm.1 | 7.84 | 7.33 | 1.42 | 0.0009 | 0.0143 | Nat2 |
| TC0100003129.mm.1 | 7 | 7.29 | -1.23 | 0.0024 | 0.0292 | Nav1 |
| TC0400000140.mm.1 | 9.75 | 9.43 | 1.24 | 0.0018 | 0.0241 | Nbn |
| TC0500003368.mm.1 | 8.27 | 8.62 | -1.27 | 0.0003 | 0.0061 | Ncf1 |
| TC1200001353.mm.1 | 11.18 | 10.95 | 1.17 | 0.0033 | 0.0372 | Ncoa1 |

| | | | | | | |
|---|---|---|---|---|---|---|
| TC0200005006.mm.1 | 12.12 | 11.83 | 1.22 | 0.0015 | 0.0201 | Ndrg3 |
| TC0900000976.mm.1 | 9.04 | 9.72 | -1.61 | 6.77E-06 | 0.0003 | Nedd4 |
| TC0100002177.mm.1 | 12.85 | 12.01 | 1.79 | 5.92E-06 | 0.0003 | Neurl3 |
| TC0200003791.mm.1 | 10.2 | 9.96 | 1.18 | 0.004 | 0.0432 | Nfe2l2 |
| TC1900000689.mm.1 | 9.94 | 9.66 | 1.21 | 0.0031 | 0.0355 | Nfkb2 |
| TC1700000864.mm.1 | 10.55 | 10.18 | 1.29 | 0.0046 | 0.0485 | Nfkbie |
| TC1600001742.mm.1 | 12.3 | 11.96 | 1.27 | 0.0003 | 0.0064 | Nfkbiz |
| TC1300000087.mm.1 | 6.16 | 6.44 | -1.21 | 0.0042 | 0.0454 | Nid1 |
| TC0700000759.mm.1 | 13.73 | 14.23 | -1.41 | 8.11E-05 | 0.002 | Nkg7 |
| TC0700000350.mm.1 | 5.15 | 5.41 | -1.2 | 0.0025 | 0.0303 | Nlrp4e |
| TC0700004656.mm.1 | 8.74 | 8.47 | 1.2 | 0.0036 | 0.0397 | Nmb |
| TC1300001812.mm.1 | 7.07 | 7.76 | -1.61 | 0.0003 | 0.0055 | Nrn1 |
| TC1100000327.mm.1 | 12.49 | 12.21 | 1.21 | 0.0012 | 0.0169 | Nsg2 |
| TC0700004388.mm.1 | 9.9 | 9.65 | 1.19 | 0.002 | 0.0255 | Nsmce4a |
| TC0400000374.mm.1 | 6.96 | 7.33 | -1.29 | 0.0031 | 0.036 | Nudt2 |
| TC1200002053.mm.1 | 10.36 | 9.84 | 1.43 | 2.21E-05 | 0.0007 | Numb |
| TC0900002233.mm.1 | 5.36 | 5.6 | -1.18 | 0.0028 | 0.033 | Nxpe2 |
| TC1600001743.mm.1 | 11.76 | 12.19 | -1.34 | 0.001 | 0.0149 | Nxpe3 |
| TC0500001349.mm.1 | 9.58 | 9.27 | 1.23 | 0.0022 | 0.028 | Oas1b |
| TC1000000140.mm.1 | 4.65 | 5.04 | -1.31 | 0.0027 | 0.0324 | Olig3 |
| TC0X00000949.mm.1 | 4.67 | 4.89 | -1.17 | 0.0029 | 0.0346 | Otud6a |
| TC0500001399.mm.1 | 10.92 | 11.36 | -1.36 | 0.0003 | 0.0053 | P2rx7 |
| TC0300002053.mm.1 | 5.34 | 6.13 | -1.73 | 0.0012 | 0.0168 | P2ry13 |
| TC1000003152.mm.1 | 13.46 | 13.7 | -1.18 | 0.0033 | 0.0379 | Pa2g4 |
| TC1900000954.mm.1 | 12.38 | 12.13 | 1.19 | 0.0025 | 0.03 | Pacs1 |
| TC0400001770.mm.1 | 8.45 | 8.74 | -1.23 | 0.0011 | 0.0156 | Padi2 |
| TC0400002063.mm.1 | 10.04 | 9.78 | 1.2 | 0.0024 | 0.0294 | Pank4 |
| TC1500002347.mm.1 | 9.48 | 9.21 | 1.21 | 0.0014 | 0.0197 | Parp10 |
| TC0400002562.mm.1 | 6.54 | 6.93 | -1.31 | 0.0002 | 0.004 | Pax5 |
| TC1800000359.mm.1 | 5.18 | 5.52 | -1.27 | 0.0004 | 0.0079 | Pcdhb5 |
| TC0100002840.mm.1 | 9.85 | 10.16 | -1.24 | 0.0021 | 0.0269 | Pdcd1 |
| TC0800002869.mm.1 | 9.83 | 9.6 | 1.17 | 0.0045 | 0.0475 | Pdf; Cog8 |
| TC0200001047.mm.1 | 12 | 11.66 | 1.27 | 0.001 | 0.0154 | Pdk1 |
| TC1100002755.mm.1 | 8.35 | 7.99 | 1.28 | 0.0041 | 0.044 | Pdlim4 |
| TC0900002347.mm.1 | 11.1 | 11.49 | -1.31 | 0.0003 | 0.0065 | Peak1 |
| TC1100003932.mm.1 | 13.07 | 12.86 | 1.16 | 0.0028 | 0.0336 | Pecam1 |
| TC1100000205.mm.1 | 14.77 | 14.47 | 1.23 | 0.0016 | 0.0215 | Peli1 |
| TC0300001601.mm.1 | 7.85 | 7.58 | 1.21 | 0.0016 | 0.0215 | Pex2 |
| TC1700000426.mm.1 | 12.43 | 12.17 | 1.2 | 0.0048 | 0.0496 | Phf1 |
| TC0200001396.mm.1 | 11.31 | 11.02 | 1.22 | 0.0017 | 0.023 | Phf21a |
| TC0100000112.mm.1 | 4.07 | 4.43 | -1.28 | 0.0022 | 0.0276 | Pi15 |
| TC1900001506.mm.1 | 8.46 | 8.87 | -1.33 | 0.0009 | 0.0134 | Pik3ap1 |
| TC1100000009.mm.1 | 10.25 | 9.99 | 1.19 | 0.0028 | 0.0331 | Pik3ip1 |
| TC0200003051.mm.1 | 14.26 | 13.99 | 1.2 | 0.0044 | 0.0464 | Pip4k2a |
| TC0700004632.mm.1 | 7.06 | 7.75 | -1.6 | 0.0002 | 0.0046 | Pira1 |
| TC0700000030.mm.1 | 6.31 | 6.7 | -1.31 | 0.0013 | 0.0189 | Pira6 |
| TC0700004631.mm.1 | 7.87 | 8.35 | -1.39 | 8.48E-05 | 0.0021 | Pirb; Pira1 |
| TC1600000163.mm.1 | 6.45 | 7.18 | -1.66 | 0.0006 | 0.0103 | Pla2g10os |
| TC0500002840.mm.1 | 7.58 | 8.16 | -1.5 | 2.29E-05 | 0.0008 | Plac8 |
| TC0800000286.mm.1 | 7.39 | 6.77 | 1.54 | 0.0002 | 0.005 | Plat |
| TC0300002172.mm.1 | 6.11 | 6.72 | -1.53 | 0.0042 | 0.0447 | Platr10 |
| TC1300000799.mm.1 | 5.26 | 5.57 | -1.25 | 0.0024 | 0.0296 | Platr2 |
| TC0600001477.mm.1 | 5.86 | 6.15 | -1.22 | 0.0019 | 0.0247 | Platr31 |
| TC0400002395.mm.1 | 5.62 | 6.13 | -1.42 | 3.81E-05 | 0.0011 | Platr9 |
| TC0600003329.mm.1 | 8.89 | 9.44 | -1.47 | 5.90E-05 | 0.0016 | Plbd1 |
| TC0800001407.mm.1 | 8.05 | 8.3 | -1.19 | 0.0025 | 0.0308 | Plcg2 |
| TC1200001272.mm.1 | 8.43 | 9.19 | -1.69 | 5.75E-06 | 0.0003 | Pld4 |
| TC0200005136.mm.1 | 6.85 | 7.18 | -1.26 | 0.001 | 0.0147 | Pltp |
| TC1000002714.mm.1 | 9.57 | 9.83 | -1.2 | 0.0015 | 0.0202 | Plxnc1 |
| TC0600002965.mm.1 | 7.62 | 7.15 | 1.39 | 0.0004 | 0.0068 | Plxnd1 |
| TC0200002026.mm.1 | 6.88 | 7.35 | -1.38 | 0.0018 | 0.0237 | Prnd; Prnp; Prn; PRND |
| TC0500000809.mm.1 | 7.19 | 7.54 | -1.27 | 0.0012 | 0.0167 | Proll |
| TC0900001119.mm.1 | 6.75 | 7.32 | -1.48 | 0.0008 | 0.013 | Prss35 |
| TC1800000834.mm.1 | 7.35 | 7.95 | -1.51 | 4.73E-05 | 0.0013 | Pstpip2 |
| TC0400001593.mm.1 | 5.86 | 6.24 | -1.3 | 0.0032 | 0.0365 | Ptafr |
| TC0300002595.mm.1 | 6.57 | 6.97 | -1.32 | 0.0009 | 0.0141 | Ptgfrn |
| TC1500001690.mm.1 | 6.98 | 7.21 | -1.17 | 0.0031 | 0.0359 | Ptk2 |
| TC0200002602.mm.1 | 11.42 | 11.2 | 1.17 | 0.0039 | 0.0426 | Ptpn1 |
| TC0200004090.mm.1 | 9.13 | 9.85 | -1.65 | 5.30E-05 | 0.0015 | Ptprj |
| TC0500002059.mm.1 | 7.52 | 7.81 | -1.23 | 0.0017 | 0.0225 | Pus7 |
| TC0700002507.mm.1 | 11.16 | 10.82 | 1.26 | 0.001 | 0.0151 | Pvr |
| TC1400000076.mm.1 | 12.14 | 11.94 | 1.15 | 0.0048 | 0.0496 | Pxk |
| TC0100003591.mm.1 | 13.55 | 13.25 | 1.23 | 0.001 | 0.0154 | Pydc4 |
| TC1200001225.mm.1 | 11.94 | 11.66 | 1.22 | 0.003 | 0.0351 | Rcor1 |
| TSUnmapped00000010. | 9.81 | 9.32 | 1.4 | 0.0005 | 0.0092 | Rcor3 |

| | | | | | | |
|---|---|---|---|---|---|---|
| TC0100003826.mm.1 | 9.07 | 8.74 | 1.25 | 0.0009 | 0.0138 | Rcor3 |
| TC0300000807.mm.1 | 7.3 | 7.99 | -1.61 | 1.01E-05 | 0.0004 | S100a4 |
| TC0700003041.mm.1 | 6.17 | 6.61 | -1.36 | 0.0046 | 0.048 | Saa1 |
| TC0800000941.mm.1 | 8.12 | 7.75 | 1.29 | 0.0003 | 0.0055 | Samd1 |
| TC0400004175.mm.1 | 5.52 | 6.18 | -1.58 | 4.41E-05 | 0.0013 | Samd11 |
| TC1000000231.mm.1 | 9.13 | 9.52 | -1.3 | 0.002 | 0.0252 | Samd3 |
| TC1600001919.mm.1 | 8.69 | 8.93 | -1.18 | 0.0036 | 0.0401 | Samsn1 |
| TC0200000468.mm.1 | 5.42 | 5.8 | -1.3 | 0.002 | 0.0255 | Sardhos; RP23-171K6.3 |
| TC0100001446.mm.1 | 7.42 | 8.09 | -1.59 | 0.0015 | 0.0203 | Scarna3a; Mir1843b |
| TC0700000610.mm.1 | 8.09 | 8.63 | -1.46 | 0.0033 | 0.0378 | Scgb1b19 |
| TC0700000612.mm.1 | 7.37 | 7.84 | -1.38 | 0.0004 | 0.0079 | Scgb1b20; Scgb1b11 |
| TC0700000625.mm.1 | 4.23 | 4.79 | -1.48 | 0.0014 | 0.0197 | Scgb1b26-ps; Scgb1b28-ps |
| TC0700000597.mm.1 | 5.46 | 6.35 | -1.85 | 3.30E-06 | 0.0002 | Scgb1b29 |
| TC0700000591.mm.1 | 5.78 | 6.51 | -1.65 | 0.0009 | 0.0134 | Scgb1b3 |
| TC0700000592.mm.1 | 7.28 | 8.17 | -1.86 | 3.55E-06 | 0.0002 | Scgb1b7 |
| TC0700002802.mm.1 | 5.36 | 6.06 | -1.62 | 0.0041 | 0.0444 | Scgb2b15; Scgb2b17 |
| TC0700002805.mm.1 | 5.36 | 6.06 | -1.62 | 0.0041 | 0.0444 | Scgb2b15; Scgb2b17 |
| TC0700002815.mm.1 | 6.47 | 6.84 | -1.29 | 0.0005 | 0.0094 | Scgb2b23-ps |
| TC0700002796.mm.1 | 5.61 | 6.03 | -1.34 | 0.0018 | 0.0237 | Scgb2b7 |
| TC1000000388.mm.1 | 11.35 | 11.04 | 1.24 | 0.0019 | 0.025 | Scml4 |
| TC0300002465.mm.1 | 9.97 | 9.34 | 1.55 | 9.70E-06 | 0.0004 | Scnm1 |
| TC0600002553.mm.1 | 8.39 | 8.08 | 1.24 | 0.0005 | 0.0091 | Sema4f |
| TC1600000307.mm.1 | 11.64 | 11.4 | 1.19 | 0.0038 | 0.0415 | Senp2 |
| TC1200002533.mm.1 | 5.1 | 5.36 | -1.19 | 0.0028 | 0.0335 | Serpina1a |
| TC1200002524.mm.1 | 7.62 | 8.36 | -1.68 | 4.30E-05 | 0.0012 | Serpina3f |
| TC1200002525.mm.1 | 9.69 | 10.52 | -1.78 | 9.64E-06 | 0.0004 | Serpina3g |
| TC1800001640.mm.1 | 6.91 | 7.18 | -1.2 | 0.0034 | 0.0383 | Setbp1 |
| TC0300001958.mm.1 | 10.4 | 10.15 | 1.2 | 0.0023 | 0.0287 | Setd7 |
| TC1100004285.mm.1 | 8.94 | 9.34 | -1.32 | 0.0002 | 0.0044 | Sfi1 |
| TC0200000087.mm.1 | 8.15 | 8.45 | -1.23 | 0.0007 | 0.0113 | Sfmbt2 |
| TC1000000171.mm.1 | 10.1 | 9.79 | 1.24 | 0.0018 | 0.024 | Sgk1 |
| TC1500002333.mm.1 | 12.71 | 12.39 | 1.25 | 0.0021 | 0.0262 | Sh3bp1 |
| TC1400001676.mm.1 | 10.5 | 10.85 | -1.27 | 0.0035 | 0.0395 | Sh3bp5 |
| TC0700000757.mm.1 | 6.47 | 6.85 | -1.3 | 0.0003 | 0.0057 | Siglecg |
| TC0700000931.mm.1 | 7.91 | 8.79 | -1.84 | 1.43E-06 | 9.33E-05 | Siglech |
| TC0200001959.mm.1 | 7.39 | 7.98 | -1.5 | 4.27E-05 | 0.0012 | Sirpa |
| TC0300003225.mm.1 | 5.95 | 6.75 | -1.74 | 7.07E-06 | 0.0003 | Sirpb1a |
| TC0300001674.mm.1 | 6.01 | 6.88 | -1.83 | 5.99E-05 | 0.0016 | Sirpb1a; Sirpb1b |
| TC0300000210.mm.1 | 11.83 | 11.6 | 1.17 | 0.0035 | 0.0392 | Skil |
| TC1800000805.mm.1 | 11.04 | 10.69 | 1.27 | 0.0003 | 0.0064 | Smad7 |
| TC0300000574.mm.1 | 15.4 | 15.14 | 1.2 | 0.0023 | 0.0282 | Smc4 |
| TC1700000152.mm.1 | 5.45 | 6.01 | -1.48 | 0.0006 | 0.0095 | Smok2a |
| TC0500003714.mm.1 | 6.61 | 7.04 | -1.34 | 0.0008 | 0.0124 | Smok3a |
| TC0500003715.mm.1 | 6.54 | 7.08 | -1.46 | 0.0004 | 0.0069 | Smok3b; Smok3a |
| TC1100003937.mm.1 | 12.16 | 11.87 | 1.22 | 0.0008 | 0.0134 | Smurf2 |
| TC0800001976.mm.1 | 11.78 | 11.1 | 1.6 | 0.0005 | 0.0086 | Snord13 |
| TSUnmapped00000175. | 11.91 | 11.05 | 1.82 | 0.0005 | 0.0089 | Snord13 |
| TC0700002993.mm.1 | 15.42 | 15.7 | -1.21 | 0.0028 | 0.0331 | Snord35a |
| TC0700002991.mm.1 | 11.69 | 12.2 | -1.42 | 0.0007 | 0.0115 | Snord35b |
| TC0300002445.mm.1 | 11.34 | 11.07 | 1.21 | 0.0015 | 0.0203 | Snx27 |
| TC0900002359.mm.1 | 9.27 | 9.04 | 1.18 | 0.0028 | 0.0331 | Snx33 |
| TC0200004756.mm.1 | 12.49 | 12.81 | -1.25 | 0.0007 | 0.0114 | Snx5 |
| TC1100004150.mm.1 | 8.27 | 7.62 | 1.57 | 5.07E-05 | 0.0014 | Socs3 |
| TC1200000321.mm.1 | 7.18 | 7.57 | -1.32 | 0.0007 | 0.0119 | Sostdc1 |
| TC0800003095.mm.1 | 6.14 | 5.85 | 1.22 | 0.0024 | 0.0295 | Spata21 |
| TC0500000072.mm.1 | 6.43 | 7.11 | -1.6 | 0.0002 | 0.004 | Speer1 |
| TC0500002115.mm.1 | 5.64 | 6.48 | -1.79 | 3.22E-05 | 0.001 | Speer4a; Gm10471 |
| TC0500002130.mm.1 | 5.83 | 6.58 | -1.68 | 0.0011 | 0.0158 | Speer4b |
| TC0500001980.mm.1 | 6.13 | 7.34 | -2.3 | 2.98E-06 | 0.0002 | Speer4c |
| TC0500001976.mm.1 | 6.06 | 7.18 | -2.17 | 2.88E-06 | 0.0002 | Speer4c; 4930572003Rik |
| TC0500000108.mm.1 | 5.71 | 6.76 | -2.07 | 1.52E-06 | 9.79E-05 | Speer4cos |
| TC0500000110.mm.1 | 6.31 | 7.4 | -2.13 | 2.63E-05 | 0.0008 | Speer4d |
| TC0500001973.mm.1 | 6.01 | 7.05 | -2.06 | 0.0003 | 0.0055 | Speer4e |
| TC1000000412.mm.1 | 5.53 | 5.94 | -1.33 | 0.0023 | 0.0287 | Speer5-ps1 |
| TC0500000106.mm.1 | 6.04 | 7.46 | -2.67 | 4.69E-05 | 0.0013 | Speer8-ps1 |
| TC0200001368.mm.1 | 7.14 | 7.41 | -1.2 | 0.0031 | 0.036 | Spi1 |
| TC0700002967.mm.1 | 6.96 | 7.64 | -1.6 | 7.81E-06 | 0.0003 | Spib |
| TC1000002647.mm.1 | 5.36 | 5.92 | -1.47 | 0.0005 | 0.0081 | Spic |
| TC0X00002421.mm.1 | 5.88 | 6.21 | -1.26 | 0.0027 | 0.032 | Spin2d |
| TC0X00002017.mm.1 | 5.11 | 6.84 | -3.31 | 9.97E-07 | 7.13E-05 | Spin2e |
| TC0X00002034.mm.1 | 5.81 | 6.76 | -1.93 | 0.0028 | 0.0328 | Spin2g |
| TC0X00000287.mm.1 | 5.06 | 6.19 | -2.19 | 0.0002 | 0.0046 | Spin2-ps6; 4930408F14Rik |
| TC0700002720.mm.1 | 8.94 | 8.56 | 1.3 | 0.0017 | 0.0225 | Spint2 |
| TC1100003147.mm.1 | 6.37 | 6.6 | -1.17 | 0.0037 | 0.041 | Spns3 |
| TC0600000957.mm.1 | 7.15 | 6.92 | 1.18 | 0.0036 | 0.04 | Spr-ps1 |

| | | | | | | |
|---|---|---|---|---|---|---|
| TC0300000821.mm.1 | 5.62 | 6.09 | -1.39 | 0.0005 | 0.0091 | Sprr2a2; Sprr2a1 |
| TC0300000822.mm.1 | 6.43 | 6.91 | -1.4 | 0.0005 | 0.009 | Sprr2a3; Sprr2a1 |
| TC0300000823.mm.1 | 5.47 | 5.94 | -1.39 | 0.0004 | 0.0067 | Sprr2b |
| TC0200000518.mm.1 | 12.88 | 12.64 | 1.18 | 0.0024 | 0.0292 | Sptan1 |
| TC0500003439.mm.1 | 11.95 | 11.7 | 1.19 | 0.0033 | 0.0375 | Srrt |
| TC0X00002922.mm.1 | 4.75 | 5.91 | -2.22 | 4.93E-05 | 0.0014 | Srsx |
| TC0X00003385.mm.1 | 4.97 | 5.42 | -1.36 | 0.0006 | 0.0105 | Ssxb1 |
| TC0X00001802.mm.1 | 5.63 | 6.07 | -1.36 | 0.0003 | 0.0056 | Ssxb2 |
| TC1600001770.mm.1 | 8.42 | 8.76 | -1.27 | 0.0021 | 0.0269 | St3gal6 |
| TC0600003383.mm.1 | 12.65 | 12.28 | 1.29 | 0.0004 | 0.0069 | St8sia1 |
| TC1800000758.mm.1 | 6.73 | 7.02 | -1.22 | 0.0029 | 0.0343 | Stard6 |
| TC1100003790.mm.1 | 11.99 | 11.73 | 1.2 | 0.0012 | 0.0178 | Stat3 |
| TC1600000507.mm.1 | 6.05 | 6.54 | -1.41 | 0.0023 | 0.0286 | Stfa2l1 |
| TC0200005164.mm.1 | 8.08 | 8.4 | -1.24 | 0.0008 | 0.0127 | Sulf2 |
| TC0700002374.mm.1 | 5.42 | 5.69 | -1.2 | 0.0013 | 0.0181 | Sult2a3 |
| TC0700002375.mm.1 | 5.41 | 5.78 | -1.3 | 0.0015 | 0.0203 | Sult2a-ps3 |
| TC1000003158.mm.1 | 9.14 | 9.41 | -1.2 | 0.0045 | 0.0474 | Suox |
| TC1000002380.mm.1 | 7.9 | 8.17 | -1.21 | 0.0023 | 0.0291 | Susd2 |
| TC0700001626.mm.1 | 8.64 | 8.96 | -1.24 | 0.0044 | 0.0468 | Swap70 |
| TC1300000612.mm.1 | 7.88 | 8.29 | -1.33 | 0.0001 | 0.0032 | Syk |
| TC0400003873.mm.1 | 10.96 | 10.61 | 1.28 | 0.0043 | 0.0456 | Szrd1 |
| TC1700000077.mm.1 | 14.65 | 14.35 | 1.23 | 0.0024 | 0.0297 | Tagap |
| TC0600003139.mm.1 | 11.58 | 11.3 | 1.22 | 0.0013 | 0.0186 | Tapbpl |
| TC1700000118.mm.1 | 6.73 | 7.11 | -1.3 | 0.0043 | 0.0456 | Tdgf1-ps2 |
| TC0300002425.mm.1 | 4.96 | 5.29 | -1.26 | 0.0024 | 0.0294 | Tdpoz1 |
| TC0700002597.mm.1 | 5.23 | 4.84 | 1.31 | 0.003 | 0.0347 | Tescl |
| TC1000001661.mm.1 | 13.07 | 12.82 | 1.19 | 0.0029 | 0.0337 | Tespa1 |
| TC1000002269.mm.1 | 8.9 | 9.2 | -1.23 | 0.0034 | 0.0385 | Tet1 |
| TC1100003928.mm.1 | 9.7 | 10 | -1.23 | 0.0007 | 0.0113 | Tex2 |
| TC1700002481.mm.1 | 10.75 | 10.39 | 1.29 | 0.0005 | 0.0094 | Tgif1 |
| TC1100002649.mm.1 | 13.89 | 13.54 | 1.28 | 0.0021 | 0.0265 | Tgtp2 |
| TC1100004148.mm.1 | 7.38 | 7.07 | 1.24 | 0.0023 | 0.0287 | Thal |
| TC0400003681.mm.1 | 8.66 | 9.14 | -1.4 | 0.0002 | 0.0046 | Themis2 |
| TC0500002480.mm.1 | 10.89 | 10.52 | 1.29 | 0.0002 | 0.0041 | Tlr1 |
| TC0400003577.mm.1 | 7.14 | 7.44 | -1.23 | 0.0024 | 0.0292 | Tlr12 |
| TC0400001738.mm.1 | 10.74 | 10.54 | 1.15 | 0.0046 | 0.0477 | Tmco4 |
| TC1000002992.mm.1 | 7.53 | 7.82 | -1.22 | 0.0047 | 0.0491 | Tmevpg1 |
| TCX_GL456233_random | 9.9 | 9.49 | 1.33 | 0.0006 | 0.0096 | Tmlhe |
| TC0300000889.mm.1 | 6.32 | 7.06 | -1.67 | 1.30E-05 | 0.0005 | Tmod4 |
| TC0500002678.mm.1 | 4.95 | 5.29 | -1.27 | 0.0003 | 0.0059 | Tmprss11c |
| TC1500001939.mm.1 | 7.51 | 7.87 | -1.28 | 0.0005 | 0.0084 | Tnfrsf13c |
| TC0400002013.mm.1 | 9.18 | 8.75 | 1.35 | 0.0004 | 0.007 | Tnfrsf25 |
| TC1100002207.mm.1 | 6.49 | 6.77 | -1.22 | 0.0035 | 0.0396 | Tns3 |
| TC1100003694.mm.1 | 7.11 | 5.91 | 2.29 | 3.04E-07 | 3.00E-05 | Tns4 |
| TC1500002349.mm.1 | 7.61 | 7.38 | 1.17 | 0.0034 | 0.0383 | Tonsl |
| TC0200003355.mm.1 | 12.29 | 12.05 | 1.18 | 0.0048 | 0.0491 | Traf1 |
| TC0100000897.mm.1 | 7.83 | 7.63 | 1.15 | 0.0048 | 0.0494 | Traf3ip1 |
| TC1500002111.mm.1 | 10.91 | 10.66 | 1.18 | 0.0033 | 0.0378 | Twf1 |
| TC0700000364.mm.1 | 4.99 | 5.64 | -1.56 | 0.0012 | 0.0172 | V1rd18; V1rd19 |
| TC0500001880.mm.1 | 5.37 | 6.17 | -1.74 | 9.82E-05 | 0.0024 | V1rg10; Vmn1r79 |
| TC1100000899.mm.1 | 13.14 | 12.78 | 1.28 | 0.0005 | 0.0093 | Vamp2 |
| TC0900001621.mm.1 | 11.31 | 10.97 | 1.26 | 0.0006 | 0.0104 | Vipr1 |
| TC1000000548.mm.1 | 11.74 | 11.48 | 1.19 | 0.0028 | 0.0332 | Vsir |
| TC1400001709.mm.1 | 8.34 | 9 | -1.59 | 7.92E-06 | 0.0003 | Wdfy4 |
| TC0300001589.mm.1 | 8.53 | 8.9 | -1.29 | 0.0003 | 0.006 | Wls |
| TC0400002271.mm.1 | 10.52 | 10.75 | -1.18 | 0.0047 | 0.0488 | Wwp1 |
| TC1100000072.mm.1 | 12.28 | 12.02 | 1.2 | 0.0011 | 0.0162 | Xbp1 |
| TC1700002526.mm.1 | 9.66 | 10.01 | -1.28 | 0.0003 | 0.0061 | Xdh |
| TC0X00002247.mm.1 | 6.91 | 7.21 | -1.24 | 0.0028 | 0.0333 | Xlr |
| TC0X00002418.mm.1 | 5.95 | 6.37 | -1.34 | 0.0012 | 0.0177 | Xlr3a |
| TC0X00000676.mm.1 | 6.7 | 7.32 | -1.53 | 0.0044 | 0.0464 | Xlr3b |
| TC0X00002424.mm.1 | 6.95 | 7.44 | -1.41 | 0.0031 | 0.0355 | Xlr3c |
| TC0X00000723.mm.1 | 6.47 | 7.22 | -1.69 | 0.0003 | 0.0064 | Xlr3d-ps |
| TC0700004265.mm.1 | 13.63 | 13.37 | 1.2 | 0.0016 | 0.0216 | Xpo6 |
| TC1100003570.mm.1 | 9.62 | 9.29 | 1.26 | 0.0009 | 0.0135 | Xylt2 |
| TC0700001816.mm.1 | 11.38 | 11.07 | 1.25 | 0.0012 | 0.0178 | Ypel3 |
| TC1100002679.mm.1 | 8.07 | 7.84 | 1.17 | 0.0032 | 0.037 | Zfp354c |
| TC1100002835.mm.1 | 10.02 | 9.72 | 1.23 | 0.0032 | 0.0366 | Zfp39 |
| TC1000000908.mm.1 | 10.27 | 10.05 | 1.17 | 0.0046 | 0.0478 | Zfp433 |
| TC0400003995.mm.1 | 6.86 | 7.13 | -1.21 | 0.0034 | 0.0384 | Zfp534 |
| TC0900001834.mm.1 | 7.35 | 7.15 | 1.15 | 0.004 | 0.0435 | Zfp560 |
| TC0400001878.mm.1 | 6.61 | 7.27 | -1.57 | 4.91E-05 | 0.0014 | Zfp600 |
| TC0800003231.mm.1 | 8.6 | 8.37 | 1.18 | 0.0049 | 0.0499 | Zfp617 |
| TC1300002228.mm.1 | 7.17 | 8.11 | -1.92 | 7.49E-05 | 0.0019 | Zfp640 |
| TC0800000874.mm.1 | 8.98 | 9.32 | -1.27 | 0.0008 | 0.0122 | Zfp827 |

| | | | | | | |
|---|---|---|---|---|---|---|
| TC0200002709.mm.1 | 12.01 | 11.74 | 1.21 | 0.0017 | 0.023 | Zfp831 |
| TC0800002368.mm.1 | 10.12 | 9.79 | 1.25 | 0.0025 | 0.0304 | Zfp868 |
| TC0800002369.mm.1 | 10.85 | 10.55 | 1.23 | 0.0037 | 0.0405 | Zfp869 |
| TC1100000839.mm.1 | 8.49 | 8.22 | 1.2 | 0.0017 | 0.0225 | Zkscan6 |
| TC0200005155.mm.1 | 10.78 | 10.57 | 1.16 | 0.0048 | 0.0493 | Zmynd8 |
| TC0800003235.mm.1 | 11 | 10.72 | 1.22 | 0.0019 | 0.0251 | Znrf1 |
| TC1100000231.mm.1 | 10.6 | 10.14 | 1.38 | 0.0012 | 0.017 | Zrsr1 |
| TC0700000113.mm.1 | 4.99 | 5.39 | -1.32 | 0.0017 | 0.0229 | Zscan4c |
| TC0700002323.mm.1 | 4.68 | 5.29 | -1.52 | 0.0001 | 0.0025 | Zscan4d |
| TC0700000115.mm.1 | 5.13 | 5.54 | -1.33 | 0.0008 | 0.0126 | Zscan4f |

| Table S4: Narrow peaks ChIPseeker annotation of HDAC8 in Teff cells (1) | | | | | | | | |
|---|---|---|---|---|---|---|---|---|
| name | signalValue | '-log10pvalue | '-log10qvalue | peak_summit | annotation | geneStart | geneEnd | geneName |
| Teff_1_peak_20565 | 4.06257 | 5.34677 | 2.44727 | 106 | Intron (ENSMUST000000256 | 34300075 | 34316677 | Fas |
| Teff_1_peak_2618 | 3.25006 | 4.00752 | 1.3737 | 123 | Distal Intergenic | 161781422 | 161788358 | Fasl |
| Teff_1_peak_5431 | 20.3079 | 41.1946 | 36.2183 | 174 | Intron (ENSMUST000002394 | 118502035 | 118555352 | Ifngas1 |
| Teff_1_peak_5432 | 7.60641 | 11.7718 | 8.1354 | 163 | Distal Intergenic | 118502035 | 118556525 | Ifngas1 |
| Teff_1_peak_5430 | 4.74045 | 6.40962 | 3.4088 | 134 | Intron (ENSMUST000002394 | 118502035 | 118555352 | Ifngas1 |
| Teff_1_peak_5433 | 3.8722 | 5.18708 | 2.43194 | 163 | Distal Intergenic | 118502035 | 118556525 | Ifngas1 |
| Teff_1_peak_3616 | 4.87509 | 6.76423 | 3.64202 | 180 | Distal Intergenic | 19591949 | 19610229 | Ifngr1 |
| Teff_1_peak_8255 | 4.06257 | 5.34677 | 2.44727 | 212 | Promoter (<=1kb) | 40203131 | 40207062 | Ifrd1 |
| Teff_1_peak_33688 | 5.2373 | 7.03074 | 3.87203 | 163 | Distal Intergenic | 138657089 | 138658544 | Igbp1b |
| Teff_1_peak_33687 | 4.8739 | 6.76096 | 3.64202 | 115 | Distal Intergenic | 138657089 | 138658544 | Igbp1b |
| Teff_1_peak_33686 | 4.06257 | 5.34677 | 2.44727 | 112 | Distal Intergenic | 138657089 | 138658544 | Igbp1b |
| Teff_1_peak_39290 | 5.63696 | 7.97698 | 4.75884 | 105 | Intron (ENSMUST000000354 | 65101535 | 65135580 | Igdcc4 |
| Teff_1_peak_4809 | 5.68622 | 8.24419 | 4.94201 | 128 | Distal Intergenic | 87826377 | 87828362 | Igf1os |
| Teff_1_peak_34858 | 21.1254 | 43.2605 | 38.2193 | 252 | Intron (ENSMUST000000056 | 68148590 | 68187969 | Igf1r |
| Teff_1_peak_34857 | 10.3968 | 17.5237 | 13.5064 | 131 | Intron (ENSMUST000000056 | 68148590 | 68187969 | Igf1r |
| Teff_1_peak_34859 | 9.65718 | 16.1589 | 12.2314 | 194 | Promoter (<=1kb) | 68210259 | 68212926 | Igf1r |
| Teff_1_peak_34855 | 5.6876 | 8.24801 | 4.94201 | 127 | Intron (ENSMUST000000056 | 67952827 | 68233668 | Igf1r |
| Teff_1_peak_34856 | 4.06158 | 5.34407 | 2.44727 | 110 | Intron (ENSMUST000000056 | 68148590 | 68187969 | Igf1r |
| Teff_1_peak_15486 | 8.20741 | 12.8886 | 9.18821 | 116 | Intron (ENSMUST000001000 | 22083942 | 22137209 | Igf2bp2 |
| Teff_1_peak_15485 | 6.32601 | 9.91631 | 6.42252 | 156 | Intron (ENSMUST000001000 | 22059074 | 22089118 | Igf2bp2 |
| Teff_1_peak_15484 | 3.89293 | 4.82424 | 2.09523 | 175 | Intron (ENSMUST000001000 | 22076018 | 22079118 | Igf2bp2 |
| Teff_1_peak_32192 | 4.86616 | 6.742 | 3.64202 | 168 | Intron (ENSMUST000000318 | 49093111 | 49214161 | Igf2bp3 |
| Teff_1_peak_32193 | 4.06257 | 5.34677 | 2.44727 | 231 | Intron (ENSMUST000000318 | 49093111 | 49214161 | Igf2bp3 |
| Teff_1_peak_32191 | 4.06257 | 5.34677 | 2.44727 | 98 | Intron (ENSMUST000000318 | 49107400 | 49111100 | Igf2bp3 |
| Teff_1_peak_17045 | 6.49854 | 9.785 | 6.3163 | 94 | Intron (ENSMUST000001609 | 12682406 | 12769664 | Igf2r |
| Teff_1_peak_17044 | 4.82859 | 6.63811 | 3.62767 | 108 | Intron (ENSMUST000000245 | 12698527 | 12701279 | Igf2r |
| Teff_1_peak_1101 | 5.35278 | 7.76387 | 4.56047 | 108 | Distal Intergenic | 72824503 | 72852474 | Igfbp2 |
| Teff_1_peak_1103 | 4.02382 | 5.24228 | 2.44727 | 100 | Distal Intergenic | 72824503 | 72852474 | Igfbp2 |
| Teff_1_peak_1102 | 3.89293 | 4.82424 | 2.09523 | 177 | Distal Intergenic | 72824503 | 72852474 | Igfbp2 |
| Teff_1_peak_1104 | 3.25006 | 4.00752 | 1.3737 | 87 | Distal Intergenic | 72824503 | 72852474 | Igfbp2 |
| Teff_1_peak_7257 | 6.43812 | 9.61993 | 6.21244 | 145 | Distal Intergenic | 99041244 | 99054392 | Igfbp4 |
| Teff_1_peak_7256 | 6.33868 | 9.17091 | 5.7961 | 124 | Distal Intergenic | 99041244 | 99054392 | Igfbp4 |
| Teff_1_peak_9435 | 9.79491 | 16.4512 | 12.4844 | 305 | Distal Intergenic | 113254830 | 113260236 | Igha |
| Teff_1_peak_9436 | 6.81263 | 11.0415 | 7.48584 | 311 | Distal Intergenic | 113254830 | 113260236 | Igha |
| Teff_1_peak_9434 | 4.53674 | 5.76459 | 2.82567 | 191 | Distal Intergenic | 113254830 | 113260236 | Igha |
| Teff_1_peak_9437 | 3.02404 | 4.00811 | 1.37413 | 96 | Downstream (2-3kb) | 113254830 | 113260236 | Igha |
| Teff_1_peak_34468 | 5.48614 | 7.97585 | 4.75786 | 101 | Intron (ENSMUST000001079 | 43474186 | 43478233 | Iglon5 |
| Teff_1_peak_40563 | 3.25006 | 4.00752 | 1.3737 | 96 | Downstream (1-2kb) | 49782536 | 49788243 | Igsf1 |
| Teff_1_peak_15791 | 4.82859 | 6.63811 | 3.62767 | 128 | Intron (ENSMUST000002095 | 38892671 | 38924468 | Igsf11 |
| Teff_1_peak_15792 | 4.06257 | 5.34677 | 2.44727 | 177 | Intron (ENSMUST000002095 | 38892671 | 38924468 | Igsf11 |
| Teff_1_peak_15793 | 3.91464 | 4.96478 | 2.22899 | 172 | Intron (ENSMUST000002095 | 38902508 | 38924468 | Igsf11 |
| Teff_1_peak_28685 | 24.8174 | 65.4539 | 59.8419 | 230 | Distal Intergenic | 140026846 | 140246784 | Igsf21 |
| Teff_1_peak_25632 | 4.78411 | 6.52117 | 3.51573 | 339 | Distal Intergenic | 101377083 | 101379540 | Igsf3 |
| Teff_1_peak_16814 | 11.3004 | 19.5869 | 15.4588 | 259 | Distal Intergenic | 96361668 | 96422121 | Igsf5 |
| Teff_1_peak_16818 | 5.6876 | 8.24801 | 4.94201 | 126 | Promoter (2-3kb) | 96386331 | 96403328 | Igsf5 |
| Teff_1_peak_16815 | 4.63987 | 6.38707 | 3.38873 | 107 | Distal Intergenic | 96361668 | 96422121 | Igsf5 |
| Teff_1_peak_16817 | 3.95037 | 5.05305 | 2.30734 | 91 | Intron (ENSMUST000001137 | 96386331 | 96403328 | Igsf5 |
| Teff_1_peak_2855 | 5.42499 | 7.63169 | 4.43834 | 108 | Intron (ENSMUST000001395 | 172311771 | 172319841 | Igsf8 |
| Teff_1_peak_19121 | 4.87509 | 6.76423 | 3.64202 | 217 | Promoter (<=1kb) | 36752355 | 36753454 | Ik |
| Teff_1_peak_5821 | 4.06158 | 5.34407 | 2.44727 | 112 | Intron (ENSMUST000001457 | 11685003 | 11707800 | Ikzf1 |
| Teff_1_peak_7246 | 12.1848 | 21.755 | 17.5156 | 182 | Intron (ENSMUST000001031 | 98484267 | 98489020 | Ikzf3 |
| Teff_1_peak_7245 | 7.31085 | 11.3767 | 7.75927 | 178 | Promoter (<=1kb) | 98484267 | 98489020 | Ikzf3 |
| Teff_1_peak_7247 | 4.87509 | 6.76423 | 3.64202 | 116 | Intron (ENSMUST000001031 | 98464902 | 98545800 | Ikzf3 |
| Teff_1_peak_5668 | 8.24028 | 13.1256 | 9.37352 | 121 | Intron (ENSMUST000002386 | 128630843 | 128669358 | Ikzf4 |
| Teff_1_peak_2131 | 5.94102 | 8.42072 | 5.08169 | 137 | Distal Intergenic | 131019845 | 131024974 | Il10 |
| Teff_1_peak_25102 | 13.9664 | 25.6341 | 21.2176 | 139 | Promoter (2-3kb) | 68690644 | 68698547 | Il12a |
| Teff_1_peak_25103 | 11.8124 | 21.8562 | 17.6016 | 122 | Distal Intergenic | 68694021 | 68698547 | Il12a |
| Teff_1_peak_25100 | 5.19839 | 7.05527 | 3.88204 | 157 | Distal Intergenic | 68690644 | 68698547 | Il12a |
| Teff_1_peak_25101 | 4.87509 | 6.76423 | 3.64202 | 95 | Distal Intergenic | 68690644 | 68698547 | Il12a |
| Teff_1_peak_25104 | 4.78411 | 6.52117 | 3.51573 | 107 | Distal Intergenic | 68694021 | 68698547 | Il12a |
| Teff_1_peak_32498 | 10.7056 | 20.977 | 16.776 | 142 | Intron (ENSMUST000000184 | 67292018 | 67376188 | Il12rb2 |
| Teff_1_peak_32494 | 10.5601 | 18.1593 | 14.0921 | 142 | Intron (ENSMUST000001174 | 67291318 | 67339694 | Il12rb2 |
| Teff_1_peak_32497 | 6.49854 | 9.785 | 6.3163 | 104 | Intron (ENSMUST000000184 | 67292018 | 67376188 | Il12rb2 |
| Teff_1_peak_32493 | 4.87509 | 6.76423 | 3.64202 | 120 | Intron (ENSMUST000001174 | 67291318 | 67339694 | Il12rb2 |
| Teff_1_peak_32496 | 4.87509 | 6.76423 | 3.64202 | 174 | Promoter (<=1kb) | 67291318 | 67339694 | Il12rb2 |
| Teff_1_peak_32495 | 4.06257 | 5.34677 | 2.44727 | 107 | Intron (ENSMUST000001174 | 67291318 | 67339694 | Il12rb2 |
| Teff_1_peak_40980 | 5.6876 | 8.24801 | 4.94201 | 111 | Distal Intergenic | 147383478 | 147403832 | Il13ra2 |
| Teff_1_peak_37426 | 6.50011 | 9.78938 | 6.3163 | 117 | Distal Intergenic | 82331914 | 82344473 | Il15 |
| Teff_1_peak_21235 | 11.7839 | 21.0232 | 16.8089 | 152 | Promoter (1-2kb) | 11723164 | 11733968 | Il15ra |
| Teff_1_peak_267 | 28.438 | 62.4018 | 56.8625 | 175 | Distal Intergenic | 20730905 | 20734496 | Il17a |
| Teff_1_peak_33337 | 6.49854 | 9.785 | 6.3163 | 110 | Distal Intergenic | 120463247 | 120483729 | Il17ra |
| Teff_1_peak_11969 | 11.3752 | 19.95 | 15.7816 | 239 | Intron (ENSMUST000000353 | 27087765 | 27101491 | Il17rd |
| Teff_1_peak_11968 | 8.07973 | 12.6436 | 8.96196 | 134 | Intron (ENSMUST000000353 | 27067397 | 27100976 | Il17rd |
| Teff_1_peak_11967 | 7.85596 | 12.2248 | 8.55132 | 114 | Intron (ENSMUST000000353 | 27067397 | 27100976 | Il17rd |
| Teff_1_peak_11966 | 3.98676 | 5.14542 | 2.3925 | 115 | Distal Intergenic | 27038941 | 27107286 | Il17rd |
| Teff_1_peak_33218 | 5.35278 | 7.76387 | 4.56047 | 116 | Distal Intergenic | 113458484 | 113470758 | Il17re |
| Teff_1_peak_603 | 5.6876 | 8.24801 | 4.94201 | 137 | Intron (ENSMUST000001677 | 40466006 | 40500854 | Il18r1 |
| Teff_1_peak_2130 | 4.8739 | 6.76096 | 3.64202 | 186 | Distal Intergenic | 130932816 | 130940115 | Il19 |
| Teff_1_peak_23134 | 4.06257 | 5.34677 | 2.44727 | 111 | Promoter (1-2kb) | 129299610 | 129309972 | Il1a |
| Teff_1_peak_21463 | 6.97254 | 10.4526 | 6.9404 | 111 | Distal Intergenic | 24291196 | 24293820 | Il1f10 |
| Teff_1_peak_21462 | 4.02382 | 5.24228 | 2.44727 | 103 | Distal Intergenic | 24153161 | 24160519 | Il1f8 |
| Teff_1_peak_595 | 8.51062 | 13.6178 | 9.83287 | 123 | Intron (ENSMUST000000272 | 40266657 | 40277178 | Il1r1 |
| Teff_1_peak_593 | 7.42628 | 11.2985 | 7.72844 | 331 | Intron (ENSMUST000000272 | 40225080 | 40317257 | Il1r1 |
| Teff_1_peak_592 | 5.6876 | 8.24801 | 4.94201 | 112 | Distal Intergenic | 40225080 | 40317257 | Il1r1 |
| Teff_1_peak_594 | 4.37955 | 5.76076 | 2.82232 | 104 | Intron (ENSMUST000000272 | 40266657 | 40277178 | Il1r1 |
| Teff_1_peak_589 | 5.2373 | 7.03074 | 3.87203 | 112 | Intron (ENSMUST000001916 | 40084698 | 40125231 | Il1r2 |
| Teff_1_peak_15584 | 7.78586 | 13.379 | 9.61786 | 121 | Intron (ENSMUST000000961 | 26624156 | 26715068 | Il1rap |
| Teff_1_peak_15582 | 5.19839 | 7.05527 | 3.88204 | 146 | Distal Intergenic | 26581704 | 26725264 | Il1rap |
| Teff_1_peak_15583 | 4.06257 | 5.34677 | 2.44727 | 76 | Promoter (1-2kb) | 26624156 | 26715068 | Il1rap |
| Teff_1_peak_40729 | 12.6247 | 22.5167 | 18.2394 | 164 | Intron (ENSMUST000001139 | 86747242 | 87890235 | Il1rapl1 |
| Teff_1_peak_40731 | 5.94102 | 8.42072 | 5.08169 | 122 | Distal Intergenic | 86769118 | 88115645 | Il1rapl1 |
| Teff_1_peak_40727 | 4.87509 | 6.76423 | 3.64202 | 117 | Intron (ENSMUST000001139 | 86747242 | 87890235 | Il1rapl1 |
| Teff_1_peak_40728 | 4.8739 | 6.76096 | 3.64202 | 135 | Intron (ENSMUST000001139 | 86747242 | 87890235 | Il1rapl1 |

| | | | | | | | | |
|---|---|---|---|---|---|---|---|---|
| Teff_1_peak_602 | 6.97254 | 10.4526 | 6.9404 | 132 | Distal Intergenic | 40429570 | 40446723 | Il1rl1 |
| Teff_1_peak_601 | 4.58264 | 5.84655 | 2.87288 | 199 | Distal Intergenic | 40429570 | 40446723 | Il1rl1 |
| Teff_1_peak_24612 | 17.871 | 35.1453 | 30.3733 | 150 | Promoter (<=1kb) | 37120523 | 37125959 | Il2 |
| Teff_1_peak_24613 | 7.11067 | 10.8439 | 7.3222 | 160 | Distal Intergenic | 37120523 | 37125959 | Il2 |
| Teff_1_peak_3621 | 10.7056 | 20.977 | 16.776 | 158 | Distal Intergenic | 19712570 | 19760053 | Il20ra |
| Teff_1_peak_39807 | 8.51062 | 13.6178 | 9.83287 | 145 | Intron (ENSMUST000000984 | 100465708 | 100486385 | Il20rb |
| Teff_1_peak_39808 | 8.12514 | 13.0194 | 9.27358 | 317 | Promoter (2-3kb) | 100465708 | 100486385 | Il20rb |
| Teff_1_peak_35815 | 8.77087 | 14.2462 | 10.4486 | 157 | Intron (ENSMUST000002062 | 125603537 | 125633570 | Il21r |
| Teff_1_peak_35816 | 3.80321 | 4.50137 | 1.82856 | 105 | Intron (ENSMUST000002062 | 125603537 | 125633570 | Il21r |
| Teff_1_peak_28625 | 8.08438 | 16.5758 | 12.5918 | 135 | Distal Intergenic | 135728172 | 135752140 | Il22ra1 |
| Teff_1_peak_3617 | 6.49854 | 9.785 | 6.3163 | 142 | Distal Intergenic | 19621998 | 19634681 | Il22ra2 |
| Teff_1_peak_3618 | 4.58264 | 5.84655 | 2.87288 | 93 | Intron (ENSMUST000000365 | 19621998 | 19634681 | Il22ra2 |
| Teff_1_peak_32502 | 11.3752 | 19.95 | 15.7816 | 153 | Intron (ENSMUST000001183 | 67422932 | 67491855 | Il23r |
| Teff_1_peak_32501 | 5.68622 | 8.24419 | 4.94201 | 98 | Intron (ENSMUST000001183 | 67422932 | 67491855 | Il23r |
| Teff_1_peak_2129 | 4.87509 | 6.76423 | 3.64202 | 106 | Distal Intergenic | 130882074 | 130887454 | Il24 |
| Teff_1_peak_35832 | 6.51595 | 9.47786 | 6.09566 | 128 | Distal Intergenic | 126589010 | 126594941 | Il27 |
| Teff_1_peak_21233 | 13.6253 | 29.2751 | 24.7091 | 154 | Promoter (2-3kb) | 11642807 | 11693193 | Il2ra |
| Teff_1_peak_21234 | 3.80321 | 4.50137 | 1.82856 | 106 | Promoter (<=1kb) | 11679708 | 11680209 | Il2ra |
| Teff_1_peak_6464 | 4.87509 | 6.76423 | 3.64202 | 113 | Promoter (<=1kb) | 54265303 | 54267277 | Il3 |
| Teff_1_peak_30917 | 14.7655 | 29.4594 | 24.883 | 182 | Intron (ENSMUST000001968 | 123480401 | 123489489 | Il31 |
| Teff_1_peak_11492 | 6.54663 | 9.54535 | 6.14107 | 133 | Promoter (1-2kb) | 112522795 | 112580662 | Il31ra |
| Teff_1_peak_11490 | 5.42108 | 8.78878 | 5.43457 | 264 | Intron (ENSMUST000002238 | 112525846 | 112532344 | Il31ra |
| Teff_1_peak_11491 | 3.25006 | 4.00752 | 1.3737 | 68 | Intron (ENSMUST000002238 | 112522795 | 112580662 | Il31ra |
| Teff_1_peak_6448 | 7.07355 | 11.8301 | 8.19063 | 135 | Distal Intergenic | 53612460 | 53618669 | Il4 |
| Teff_1_peak_29333 | 4.87509 | 6.76423 | 3.64202 | 128 | Intron (ENSMUST000000268 | 30013321 | 30019839 | Il6 |
| Teff_1_peak_29332 | 4.06257 | 5.34677 | 2.44727 | 155 | Promoter (<=1kb) | 30013321 | 30019839 | Il6 |
| Teff_1_peak_25433 | 6.50011 | 9.78938 | 6.3163 | 125 | Distal Intergenic | 89864059 | 89913196 | Il6ra |
| Teff_1_peak_25434 | 3.21906 | 3.9246 | 1.3737 | 61 | Distal Intergenic | 89864059 | 89913196 | Il6ra |
| Teff_1_peak_24093 | 7.31263 | 11.3816 | 7.75927 | 129 | Distal Intergenic | 7573182 | 7613760 | Il7 |
| Teff_1_peak_24092 | 4.06257 | 5.34677 | 2.44727 | 124 | Intron (ENSMUST000001812 | 7573182 | 7613760 | Il7 |
| Teff_1_peak_13646 | 13.8127 | 25.4723 | 21.0571 | 143 | Intron (ENSMUST000002287 | 9511898 | 9529766 | Il7r |
| Teff_1_peak_13647 | 5.6876 | 8.24801 | 4.94201 | 122 | Intron (ENSMUST000001602 | 9516409 | 9530176 | Il7r |
| Teff_1_peak_13645 | 4.8739 | 6.76096 | 3.64202 | 152 | Downstream (1-2kb) | 9511898 | 9529766 | Il7r |
| Teff_1_peak_2726 | 4.87509 | 6.76423 | 3.64202 | 167 | Intron (ENSMUST000001114 | 166254193 | 166311270 | Ildr2 |
| Teff_1_peak_38590 | 6.50011 | 9.78938 | 6.3163 | 164 | Promoter (<=1kb) | 21377126 | 21405090 | Ilf3 |

| Table S5: Narrow peaks ChIPseeker annotation of HDAC8 in Teff cells (2) | | | | | | | | |
|---|---|---|---|---|---|---|---|---|
| name | signalValue | '-log10pvalue | '-log10qvalue | peak_summit | annotation | geneStart | geneEnd | geneName |
| Teff_2_peak_30502 | 3.97432 | 4.94962 | 2.1674 | 120 | Distal Intergenic | 43662346 | 43740972 | Cc2d2a |
| Teff_2_peak_30504 | 4.10242 | 5.458 | 2.50376 | 139 | Intron (ENSMUST0000 | 43696582 | 43717017 | Cc2d2a |
| Teff_2_peak_30503 | 3.98284 | 4.81771 | 2.04745 | 192 | Promoter (<=1kb) | 43662346 | 43740972 | Cc2d2a |
| Teff_2_peak_21408 | 13.2761 | 24.5046 | 20.0717 | 149 | Promoter (2-3kb) | 40774370 | 40778148 | Cc2d2b |
| Teff_2_peak_20368 | 5.74473 | 8.40906 | 5.0264 | 106 | Distal Intergenic | 66269021 | 66302741 | Ccbe1 |
| Teff_2_peak_20369 | 6.22957 | 9.18015 | 5.74621 | 127 | Distal Intergenic | 66269021 | 66302741 | Ccbe1 |
| Teff_2_peak_41579 | 4.17367 | 5.17321 | 2.37415 | 148 | Distal Intergenic | 121489824 | 121493564 | Cck |
| Teff_2_peak_41580 | 4.6268 | 7.46535 | 4.22207 | 116 | Distal Intergenic | 121489824 | 121493564 | Cck |
| Teff_2_peak_7231 | 5.74473 | 8.40906 | 5.0264 | 109 | Distal Intergenic | 82176657 | 82179812 | Ccl1 |
| Teff_2_peak_7237 | 14.6038 | 27.761 | 23.2049 | 182 | Distal Intergenic | 82176657 | 82305690 | Ccl1 |
| Teff_2_peak_7233 | 4.92405 | 6.9017 | 3.72503 | 131 | Intron (ENSMUST0000 | 82176657 | 82305690 | Ccl1 |
| Teff_2_peak_7234 | 5.31046 | 7.1641 | 3.94449 | 161 | Intron (ENSMUST0000 | 82176657 | 82305690 | Ccl1 |
| Teff_2_peak_7235 | 4.92405 | 6.9017 | 3.72503 | 147 | Intron (ENSMUST0000 | 82176657 | 82305690 | Ccl1 |
| Teff_2_peak_7232 | 16.4135 | 31.6871 | 26.9664 | 256 | Intron (ENSMUST0000 | 82176659 | 82179812 | Ccl1 |
| Teff_2_peak_7236 | 4.9679 | 6.90231 | 3.72503 | 125 | Promoter (1-2kb) | 82176657 | 82305690 | Ccl1 |
| Teff_2_peak_1281 | 4.36614 | 6.20454 | 3.16549 | 219 | Distal Intergenic | 83116793 | 83119167 | Ccl20 |
| Teff_2_peak_38854 | 11.2936 | 19.5082 | 15.3251 | 132 | Distal Intergenic | 94745590 | 94751699 | Ccl22 |
| Teff_2_peak_38855 | 3.76453 | 4.61687 | 1.89299 | 80 | Distal Intergenic | 94745590 | 94751699 | Ccl22 |
| Teff_2_peak_38856 | 9.2933 | 15.2818 | 11.3219 | 209 | Distal Intergenic | 94745590 | 94751699 | Ccl22 |
| Teff_2_peak_37363 | 7.38436 | 11.5849 | 7.89665 | 147 | Promoter (1-2kb) | 4332259 | 4334807 | Ccl25 |
| Teff_2_peak_12037 | 3.2827 | 4.09797 | 1.41255 | 162 | Promoter (1-2kb) | 119623819 | 119654359 | Ccl28 |
| Teff_2_peak_7254 | 8.28196 | 13.0543 | 9.2873 | 145 | Intron (ENSMUST0000 | 83587882 | 83593087 | Ccl6 |
| Teff_2_peak_7252 | 9.04295 | 14.6368 | 10.7517 | 146 | Distal Intergenic | 83575318 | 83577142 | Ccl9 |
| Teff_2_peak_5953 | 4.92405 | 6.9017 | 3.72503 | 109 | Intron (ENSMUST0000 | 6561230 | 6596742 | Ccm2 |
| Teff_2_peak_14839 | 5.74473 | 8.40906 | 5.0264 | 156 | Distal Intergenic | 54745702 | 54754039 | Ccn3 |
| Teff_2_peak_15053 | 15.5028 | 30.7666 | 26.0951 | 171 | Intron (ENSMUST0000 | 66909311 | 66919235 | Ccn4 |
| Teff_2_peak_27846 | 7.52906 | 11.528 | 7.88604 | 140 | Intron (ENSMUST0000 | 21727767 | 21748554 | Ccnc |
| Teff_2_peak_18337 | 3.31903 | 3.7386 | 1.30114 | 172 | Intron (ENSMUST0000 | 47505241 | 47598649 | Ccnd3 |
| Teff_2_peak_18338 | 5.49671 | 7.74757 | 4.49151 | 112 | Intron (ENSMUST0000 | 47505241 | 47598649 | Ccnd3 |
| Teff_2_peak_18339 | 5.54433 | 7.86753 | 4.60879 | 121 | Intron (ENSMUST0000 | 47505241 | 47598649 | Ccnd3 |
| Teff_2_peak_18340 | 4.92291 | 6.89843 | 3.72503 | 127 | Intron (ENSMUST0000 | 47593436 | 47598174 | Ccnd3 |
| Teff_2_peak_23707 | 4.83251 | 6.30984 | 3.26795 | 104 | Promoter (<=1kb) | 121011055 | 121011756 | Ccndbp1 |
| Teff_2_peak_35515a | 16.8908 | 38.7077 | 33.7558 | 180 | Distal Intergenic | 38097984 | 38107534 | Ccne1 |
| Teff_2_peak_35515b | 5.86615 | 8.58555 | 5.19271 | 549 | Distal Intergenic | 38097984 | 38107534 | Ccne1 |
| Teff_2_peak_6480 | 13.1278 | 23.9695 | 19.5727 | 178 | Distal Intergenic | 40748552 | 40755311 | Ccng1 |
| Teff_2_peak_6481 | 3.47753 | 4.04546 | 1.41255 | 146 | Distal Intergenic | 40748552 | 40755311 | Ccng1 |
| Teff_2_peak_6482 | 4.92405 | 6.9017 | 3.72503 | 137 | Distal Intergenic | 40748552 | 40755311 | Ccng1 |
| Teff_2_peak_6483 | 4.10338 | 5.46071 | 2.50376 | 146 | Distal Intergenic | 40748552 | 40755311 | Ccng1 |
| Teff_2_peak_31302 | 3.82758 | 4.75883 | 2.02433 | 62 | Distal Intergenic | 93268708 | 93273485 | Ccng2 |
| Teff_2_peak_21409 | 3.47753 | 4.04546 | 1.41255 | 203 | Exon (ENSMUST00000 | 40831279 | 40848572 | Ccnj |
| Teff_2_peak_21410 | 3.31903 | 3.7386 | 1.30114 | 101 | Intron (ENSMUST0000 | 40831279 | 40848572 | Ccnj |
| Teff_2_peak_6514 | 6.39177 | 9.49669 | 6.05007 | 140 | Intron (ENSMUST0000 | 43529246 | 43556689 | Ccnjl |
| Teff_2_peak_6515 | 6.77615 | 10.0428 | 6.48531 | 132 | Intron (ENSMUST0000 | 43529246 | 43556689 | Ccnjl |
| Teff_2_peak_25861 | 4.10338 | 5.46071 | 2.50376 | 137 | Promoter (2-3kb) | 65946782 | 65947998 | Ccnl1 |
| Teff_2_peak_7141 | 12.6123 | 22.9075 | 18.548 | 178 | Distal Intergenic | 78750506 | 78751729 | Ccnq |
| Teff_2_peak_41625 | 3.2827 | 4.09797 | 1.41255 | 100 | Distal Intergenic | 123962124 | 123968692 | Ccr1 |
| Teff_2_peak_41626 | 4.87911 | 6.77535 | 3.70633 | 106 | Distal Intergenic | 123977243 | 123978408 | Ccr1l1 |
| Teff_2_peak_41627 | 20.578 | 43.2871 | 38.1892 | 170 | Distal Intergenic | 123977243 | 123978408 | Ccr1l1 |
| Teff_2_peak_41628 | 3.98284 | 4.81771 | 2.04745 | 213 | Distal Intergenic | 124128748 | 124147699 | Ccr5 |
| Teff_2_peak_7551 | 5.74473 | 8.40906 | 5.0264 | 116 | Distal Intergenic | 99144196 | 99155077 | Ccr7 |
| Teff_2_peak_33443 | 3.2827 | 4.09797 | 1.41255 | 189 | Intron (ENSMUST0000 | 61373997 | 61811718 | Ccser1 |
| Teff_2_peak_33446 | 11.4895 | 20.2764 | 16.0329 | 179 | Intron (ENSMUST0000 | 61570670 | 61820582 | Ccser1 |
| Teff_2_peak_33447 | 6.95506 | 11.272 | 7.64474 | 222 | Intron (ENSMUST0000 | 61570670 | 61820582 | Ccser1 |
| Teff_2_peak_33448 | 5.55691 | 9.01087 | 5.58791 | 124 | Intron (ENSMUST0000 | 61785111 | 61820121 | Ccser1 |
| Teff_2_peak_33449 | 7.96568 | 12.4285 | 8.69114 | 155 | Intron (ENSMUST0000 | 61785111 | 61820121 | Ccser1 |
| Teff_2_peak_33450 | 4.10338 | 5.46071 | 2.50376 | 102 | Intron (ENSMUST0000 | 61785111 | 61820121 | Ccser1 |
| Teff_2_peak_33451 | 4.10338 | 5.46071 | 2.50376 | 190 | Intron (ENSMUST0000 | 61956689 | 62382865 | Ccser1 |
| Teff_2_peak_33452 | 3.2827 | 4.09797 | 1.41255 | 84 | Intron (ENSMUST0000 | 61956689 | 62382865 | Ccser1 |
| Teff_2_peak_33444 | 11.2847 | 19.7813 | 15.58 | 146 | Promoter (<=1kb) | 61374040 | 61470265 | Ccser1 |
| Teff_2_peak_33445 | 4.92405 | 6.9017 | 3.72503 | 90 | Promoter (1-2kb) | 61570670 | 61820582 | Ccser1 |
| Teff_2_peak_12577 | 4.64665 | 5.96237 | 2.93175 | 190 | Distal Intergenic | 36874936 | 36968777 | Ccser2 |
| Teff_2_peak_12573 | 4.92405 | 6.9017 | 3.72503 | 114 | Downstream (<1kb) | 36879570 | 36886360 | Ccser2 |
| Teff_2_peak_12576 | 6.5654 | 9.97402 | 6.43068 | 143 | Intron (ENSMUST0000 | 36900790 | 36939945 | Ccser2 |
| Teff_2_peak_12575 | 6.02325 | 8.603 | 5.19669 | 177 | Promoter (<=1kb) | 36878840 | 36906318 | Ccser2 |
| Teff_2_peak_12574 | 4.10338 | 5.46071 | 2.50376 | 148 | Promoter (2-3kb) | 36878840 | 36906318 | Ccser2 |
| Teff_2_peak_26212 | 5.74339 | 8.40523 | 5.0264 | 446 | Intron (ENSMUST0000 | 88302838 | 88320650 | Cct3 |
| Teff_2_peak_26211 | 5.74473 | 8.40906 | 5.0264 | 128 | Promoter (<=1kb) | 88302838 | 88320650 | Cct3 |
| Teff_2_peak_30201 | 4.10242 | 5.458 | 2.50376 | 192 | Promoter (<=1kb) | 25516067 | 25518027 | Cct8l1 |
| Teff_2_peak_32264 | 4.64665 | 5.96237 | 2.93175 | 199 | Promoter (<=1kb) | 143992478 | 144004159 | Ccz1 |
| Teff_2_peak_4098 | 5.74473 | 8.40906 | 5.0264 | 107 | Distal Intergenic | 41520044 | 41526876 | Cd164 |
| Teff_2_peak_11723 | 3.98284 | 4.81771 | 2.04745 | 115 | Distal Intergenic | 102693558 | 102706955 | Cd180 |
| Teff_2_peak_11724 | 3.79795 | 4.49235 | 1.79138 | 105 | Distal Intergenic | 102693558 | 102706955 | Cd180 |
| Teff_2_peak_11725 | 5.74339 | 8.40523 | 5.0264 | 92 | Intron (ENSMUST0000 | 102693611 | 102739504 | Cd180 |
| Teff_2_peak_37052 | 5.97426 | 8.41633 | 5.0264 | 104 | Distal Intergenic | 126409362 | 126409796 | Cd19 |
| Teff_2_peak_26195 | 7.30188 | 11.0524 | 7.43798 | 127 | Distal Intergenic | 86986551 | 86989780 | Cd1d2 |
| Teff_2_peak_26444 | 3.92622 | 4.99313 | 2.20858 | 140 | Promoter (2-3kb) | 101275899 | 101287939 | Cd2 |
| Teff_2_peak_16484 | 8.20675 | 13.2513 | 9.43183 | 132 | Distal Intergenic | 45392245 | 45400312 | Cd200 |
| Teff_2_peak_16485 | 9.02533 | 14.9479 | 11.0176 | 136 | Downstream (1-2kb) | 45392245 | 45400312 | Cd200 |
| Teff_2_peak_16486 | 7.30188 | 11.0524 | 7.43798 | 188 | Intron (ENSMUST0000 | 45392245 | 45400312 | Cd200 |
| Teff_2_peak_16487 | 4.10338 | 5.46071 | 2.50376 | 100 | Promoter (2-3kb) | 45392245 | 45400312 | Cd200 |
| Teff_2_peak_16475 | 4.47111 | 5.90358 | 2.90631 | 115 | Intron (ENSMUST0000 | 44765826 | 44794344 | Cd200r1 |
| Teff_2_peak_16477 | 5.31046 | 7.1641 | 3.94449 | 112 | Distal Intergenic | 44965254 | 44966854 | Cd200r3 |
| Teff_2_peak_16476 | 3.76453 | 4.61687 | 1.89299 | 210 | Intron (ENSMUST0000 | 44820728 | 44839150 | Cd200r4 |
| Teff_2_peak_37354 | 9.2933 | 15.2818 | 11.3219 | 362 | Downstream (1-2kb) | 3743801 | 3748927 | Cd209a |

| | | | | | | | | |
|---|---|---|---|---|---|---|---|---|
| Teff_2_peak_37355 | 3.2827 | 4.09797 | 1.41255 | 93 | Intron (ENSMUST0000 | 3743801 | 3748927 | Cd209a |
| Teff_2_peak_37360 | 4.92291 | 6.89843 | 3.72503 | 113 | Intron (ENSMUST0000 | 3918677 | 3926813 | Cd209b |
| Teff_2_peak_37358 | 4.79382 | 6.54641 | 3.48709 | 133 | Distal Intergenic | 3871824 | 3878555 | Cd209d |
| Teff_2_peak_37359 | 8.20675 | 13.2513 | 9.43183 | 154 | Distal Intergenic | 3871824 | 3878555 | Cd209d |
| Teff_2_peak_37357 | 4.10338 | 5.46071 | 2.50376 | 112 | Distal Intergenic | 3847965 | 3854309 | Cd209e |
| Teff_2_peak_37356 | 5.31046 | 7.1641 | 3.94449 | 171 | Intron (ENSMUST0000 | 3847965 | 3854309 | Cd209e |
| Teff_2_peak_20829 | 4.92291 | 6.89843 | 3.72503 | 108 | Distal Intergenic | 89206101 | 89207338 | Cd226 |
| Teff_2_peak_20827 | 4.92291 | 6.89843 | 3.72503 | 109 | Intron (ENSMUST0000 | 89206101 | 89207338 | Cd226 |
| Teff_2_peak_20828 | 4.64665 | 5.96237 | 2.93175 | 146 | Intron (ENSMUST0000 | 89206101 | 89207338 | Cd226 |
| Teff_2_peak_2907 | 7.1446 | 13.1601 | 9.38856 | 192 | Intron (ENSMUST0000 | 171579063 | 171585318 | Cd244a |
| Teff_2_peak_2908 | 4.64665 | 5.96237 | 2.93175 | 95 | Intron (ENSMUST0000 | 171579063 | 171585318 | Cd244a |
| Teff_2_peak_2768 | 11.4868 | 20.2686 | 16.0329 | 139 | Intron (ENSMUST0000 | 165788765 | 165870249 | Cd247 |
| Teff_2_peak_34523 | 6.95506 | 11.272 | 7.64474 | 123 | Exon (ENSMUST00000 | 125232622 | 125237010 | Cd27 |
| Teff_2_peak_931 | 5.31046 | 7.1641 | 3.94449 | 140 | Intron (ENSMUST0000 | 60746358 | 60773359 | Cd28 |
| Teff_2_peak_18242 | 4.51743 | 5.8824 | 2.89265 | 156 | Distal Intergenic | 42816264 | 42876665 | Cd2ap |
| Teff_2_peak_18243 | 5.97426 | 8.41633 | 5.0264 | 92 | Distal Intergenic | 42816264 | 42876665 | Cd2ap |
| Teff_2_peak_18244 | 4.51743 | 5.8824 | 2.89265 | 152 | Distal Intergenic | 42816264 | 42876665 | Cd2ap |
| Teff_2_peak_18245 | 5.31046 | 7.1641 | 3.94449 | 109 | Distal Intergenic | 42816264 | 42876665 | Cd2ap |
| Teff_2_peak_18246 | 4.64665 | 5.96237 | 2.93175 | 131 | Distal Intergenic | 42816264 | 42876665 | Cd2ap |
| Teff_2_peak_18241 | 7.24474 | 11.1372 | 7.51869 | 129 | Intron (ENSMUST0000 | 42825421 | 42831030 | Cd2ap |
| Teff_2_peak_7883 | 6.22845 | 9.08335 | 5.65213 | 115 | Distal Intergenic | 114890041 | 114904654 | Cd300a |
| Teff_2_peak_7887 | 5.81779 | 8.13128 | 4.85068 | 95 | Exon (ENSMUST00000 | 115120357 | 115126791 | Cd300lf |
| Teff_2_peak_7888 | 7.16347 | 11.976 | 8.27117 | 103 | Intron (ENSMUST0000 | 115120357 | 115126791 | Cd300lf |
| Teff_2_peak_7890 | 4.64665 | 5.96237 | 2.93175 | 111 | Promoter (<=1kb) | 115116214 | 115133992 | Cd300lf |
| Teff_2_peak_7889 | 4.52495 | 5.74368 | 2.77404 | 191 | Promoter (2-3kb) | 115120261 | 115126850 | Cd300lf |
| Teff_2_peak_30085 | 4.17367 | 5.17321 | 2.37415 | 103 | Distal Intergenic | 17782016 | 17835696 | Cd36 |
| Teff_2_peak_30086 | 5.74473 | 8.40906 | 5.0264 | 101 | Intron (ENSMUST0000 | 17782016 | 17835696 | Cd36 |
| Teff_2_peak_40243 | 5.54433 | 7.86753 | 4.60879 | 105 | Promoter (<=1kb) | 44969572 | 44980431 | Cd3g |
| Teff_2_peak_34512 | 3.04976 | 6.1612 | 3.12474 | 129 | Intron (ENSMUST0000 | 124867333 | 124888199 | Cd4 |
| Teff_2_peak_34513 | 3.85728 | 24.2124 | 19.795 | 189 | Intron (ENSMUST0000 | 124867333 | 124888199 | Cd4 |
| Teff_2_peak_34515 | 6.1603 | 15.5774 | 11.6101 | 150 | Promoter (<=1kb) | 124867333 | 124888199 | Cd4 |
| Teff_2_peak_34514 | 15.3321 | 102.437 | 95.8422 | 234 | Promoter (1-2kb) | 124867333 | 124888199 | Cd4 |
| Teff_2_peak_23420 | 7.92048 | 12.4712 | 8.72434 | 143 | Distal Intergenic | 102811141 | 102901665 | Cd44 |
| Teff_2_peak_23419 | 3.96341 | 6.3117 | 3.26967 | 129 | Promoter (1-2kb) | 102811141 | 102901665 | Cd44 |
| Teff_2_peak_3403 | 3.98284 | 4.81771 | 2.04745 | 130 | Promoter (2-3kb) | 195036826 | 195092249 | Cd46 |
| Teff_2_peak_16579 | 5.74339 | 8.40523 | 5.0264 | 131 | Distal Intergenic | 49866833 | 49911091 | Cd47 |
| Teff_2_peak_16580 | 9.2933 | 15.2818 | 11.3219 | 172 | Distal Intergenic | 49866833 | 49911091 | Cd47 |
| Teff_2_peak_16581 | 6.56388 | 9.96963 | 6.43068 | 119 | Distal Intergenic | 49866833 | 49911091 | Cd47 |
| Teff_2_peak_16582 | 4.10338 | 5.46071 | 2.50376 | 179 | Distal Intergenic | 49866833 | 49911091 | Cd47 |
| Teff_2_peak_16583 | 9.02743 | 14.9539 | 11.0176 | 121 | Distal Intergenic | 49866833 | 49911091 | Cd47 |
| Teff_2_peak_16577 | 3.2827 | 4.09797 | 1.41255 | 98 | Intron (ENSMUST0000 | 49866833 | 49911091 | Cd47 |
| Teff_2_peak_16578 | 8.20675 | 13.2513 | 9.43183 | 169 | Intron (ENSMUST0000 | 49866833 | 49911091 | Cd47 |
| Teff_2_peak_16576 | 4.92405 | 6.9017 | 3.72503 | 123 | Intron (ENSMUST0000 | 49866833 | 49911091 | Cd47 |
| Teff_2_peak_26522 | 5.97426 | 8.41633 | 5.0264 | 217 | Intron (ENSMUST0000 | 106759921 | 106790149 | Cd53 |
| Teff_2_peak_26523 | 3.98284 | 4.81771 | 2.04745 | 153 | Intron (ENSMUST0000 | 106759921 | 106790149 | Cd53 |
| Teff_2_peak_2157 | 4.92291 | 6.89843 | 3.72503 | 116 | Promoter (<=1kb) | 130440154 | 130448849 | Cd55 |
| Teff_2_peak_2153 | 5.74473 | 8.40906 | 5.0264 | 117 | Distal Intergenic | 130419601 | 130422740 | Cd55b |
| Teff_2_peak_2154 | 4.47111 | 5.90358 | 2.90631 | 99 | Distal Intergenic | 130419601 | 130422740 | Cd55b |
| Teff_2_peak_2155 | 4.06593 | 5.35599 | 2.50376 | 87 | Intron (ENSMUST0000 | 130419601 | 130422740 | Cd55b |
| Teff_2_peak_2156 | 4.83609 | 6.65819 | 3.594 | 146 | Intron (ENSMUST0000 | 130419601 | 130422740 | Cd55b |
| Teff_2_peak_20957 | 4.64665 | 5.96237 | 2.93175 | 111 | Promoter (<=1kb) | 10789341 | 10830058 | Cd6 |
| Teff_2_peak_20956 | 4.10338 | 5.46071 | 2.50376 | 192 | Promoter (2-3kb) | 10794564 | 10829856 | Cd6 |
| Teff_2_peak_5848 | 4.92405 | 6.9017 | 3.72503 | 145 | Distal Intergenic | 128900989 | 128912822 | Cd63 |
| Teff_2_peak_6983 | 12.1548 | 22.4558 | 18.1218 | 148 | Promoter (<=1kb) | 69664306 | 69666062 | Cd68 |
| Teff_2_peak_34599 | 7.75705 | 12.0436 | 8.3332 | 133 | Distal Intergenic | 129267325 | 129275436 | Cd69 |
| Teff_2_peak_28167 | 3.2827 | 4.09797 | 1.41255 | 98 | Distal Intergenic | 43447724 | 43454628 | Cd72 |
| Teff_2_peak_20244 | 4.10242 | 5.458 | 2.50376 | 150 | Distal Intergenic | 60803848 | 60809253 | Cd74 |
| Teff_2_peak_35301 | 8.97466 | 14.5018 | 10.6298 | 171 | Distal Intergenic | 24897381 | 24902197 | Cd79a |
| Teff_2_peak_7671 | 4.65667 | 5.98066 | 2.94866 | 100 | Downstream (<1kb) | 106311344 | 106314529 | Cd79b |
| Teff_2_peak_10667 | 6.02325 | 8.603 | 5.19669 | 153 | Distal Intergenic | 43784775 | 43803132 | Cd83 |
| Teff_2_peak_10668 | 4.10242 | 5.458 | 2.50376 | 170 | Distal Intergenic | 43785185 | 43803128 | Cd83 |
| Teff_2_peak_2915 | 9.8481 | 16.6943 | 12.6544 | 151 | Intron (ENSMUST0000 | 171840633 | 171886430 | Cd84 |
| Teff_2_peak_16333 | 5.96148 | 9.01831 | 5.58862 | 130 | Intron (ENSMUST0000 | 36603869 | 36666077 | Cd86 |
| Teff_2_peak_16332 | 10.6688 | 18.4693 | 14.3201 | 149 | Intron (ENSMUST0000 | 36603897 | 36604519 | Cd86 |
| Teff_2_peak_34528 | 7.11063 | 10.7008 | 7.11598 | 164 | Exon (ENSMUST00000 | 125460266 | 125494791 | Cd9 |
| Teff_2_peak_21310 | 5.74339 | 8.40523 | 5.0264 | 131 | Distal Intergenic | 34300075 | 34316677 | Fas |
| Teff_2_peak_21307 | 4.92405 | 6.9017 | 3.72503 | 146 | Intron (ENSMUST0000 | 34300075 | 34316677 | Fas |
| Teff_2_peak_21308 | 4.10338 | 5.46071 | 2.50376 | 200 | Intron (ENSMUST0000 | 34300075 | 34316677 | Fas |
| Teff_2_peak_21309 | 4.92405 | 6.9017 | 3.72503 | 189 | Intron (ENSMUST0000 | 34300075 | 34316677 | Fas |
| Teff_2_peak_2683 | 4.10338 | 5.46071 | 2.50376 | 109 | Distal Intergenic | 161780689 | 161788495 | Fasl |
| Teff_2_peak_2682 | 4.92405 | 6.9017 | 3.72503 | 370 | Promoter (2-3kb) | 161780689 | 161788495 | Fasl |
| Teff_2_peak_5570 | 4.64665 | 5.96237 | 2.93175 | 122 | Distal Intergenic | 118441046 | 118445892 | Ifng |
| Teff_2_peak_5575 | 5.81779 | 8.13128 | 4.85068 | 93 | Distal Intergenic | 118502035 | 118556525 | Ifngas1 |
| Teff_2_peak_5574 | 4.10242 | 5.458 | 2.50376 | 107 | Intron (ENSMUST0000 | 118502035 | 118555352 | Ifngas1 |
| Teff_2_peak_5573 | 17.2302 | 33.6584 | 28.8807 | 197 | Intron (ENSMUST0000 | 118502035 | 118555352 | Ifngas1 |
| Teff_2_peak_17305 | 3.31903 | 3.7386 | 1.30114 | 209 | Downstream (1-2kb) | 91561323 | 91565169 | Ifngr2 |
| Teff_2_peak_17297 | 4.10338 | 5.46071 | 2.50376 | 152 | Distal Intergenic | 91406444 | 91414632 | Il10rb |
| Teff_2_peak_25909 | 6.02325 | 8.603 | 5.19669 | 120 | Distal Intergenic | 68690644 | 68698547 | Il12a |
| Teff_2_peak_25911 | 7.24474 | 11.1372 | 7.51869 | 95 | Distal Intergenic | 68694021 | 68698547 | Il12a |
| Teff_2_peak_25910 | 10.2082 | 17.2243 | 13.1606 | 165 | Promoter (2-3kb) | 68690644 | 68698547 | Il12a |
| Teff_2_peak_33540 | 6.56388 | 9.96963 | 6.43068 | 129 | Exon (ENSMUST00000 | 67291318 | 67339694 | Il12rb2 |
| Teff_2_peak_33542 | 11.923 | 24.0808 | 19.6682 | 142 | Intron (ENSMUST0000 | 67292018 | 67376188 | Il12rb2 |
| Teff_2_peak_33541 | 4.92291 | 6.89843 | 3.72503 | 316 | Intron (ENSMUST0000 | 67291318 | 67339694 | Il12rb2 |
| Teff_2_peak_33543 | 7.26475 | 10.9834 | 7.39244 | 152 | Promoter (2-3kb) | 67292018 | 67376188 | Il12rb2 |
| Teff_2_peak_6684 | 6.35075 | 11.0334 | 7.43798 | 124 | Downstream (1-2kb) | 53631324 | 53634702 | Il13 |
| Teff_2_peak_41852 | 3.97432 | 4.94962 | 2.1674 | 119 | Intron (ENSMUST0000 | 36112110 | 36171259 | Il13ra1 |

| | | | | | | | | |
|---|---|---|---|---|---|---|---|---|
| Teff_2_peak_38669 | 8.20675 | 13.2513 | 9.43183 | 117 | Distal Intergenic | 82331914 | 82344473 | Il15 |
| Teff_2_peak_21982 | 4.92405 | 6.9017 | 3.72503 | 169 | Intron (ENSMUST0000 | 11723164 | 11733968 | Il15ra |
| Teff_2_peak_21981 | 11.9485 | 21.3327 | 17.0479 | 198 | Promoter (1-2kb) | 11723164 | 11733968 | Il15ra |
| Teff_2_peak_268 | 23.7996 | 50.1937 | 44.8763 | 239 | Distal Intergenic | 20730905 | 20734496 | Il17a |
| Teff_2_peak_269 | 3.76453 | 4.61687 | 1.89299 | 62 | Promoter (1-2kb) | 20777679 | 20779554 | Il17f |
| Teff_2_peak_34443 | 4.51743 | 5.8824 | 2.89265 | 142 | Distal Intergenic | 120463247 | 120483729 | Il17ra |
| Teff_2_peak_12404 | 4.92405 | 6.9017 | 3.72503 | 94 | Intron (ENSMUST0000 | 27067397 | 27100976 | Il17rd |
| Teff_2_peak_12405 | 6.85184 | 10.1183 | 6.55995 | 154 | Intron (ENSMUST0000 | 27067397 | 27100976 | Il17rd |
| Teff_2_peak_12406 | 12.3101 | 22.1134 | 17.791 | 234 | Intron (ENSMUST0000 | 27087765 | 27101491 | Il17rd |
| Teff_2_peak_40359 | 3.98284 | 4.81771 | 2.04745 | 144 | Distal Intergenic | 50575273 | 50581837 | Il18 |
| Teff_2_peak_626 | 5.74473 | 8.40906 | 5.0264 | 119 | Intron (ENSMUST0000 | 40466006 | 40500854 | Il18r1 |
| Teff_2_peak_628 | 5.31046 | 7.1641 | 3.94449 | 96 | Intron (ENSMUST0000 | 40541692 | 40547903 | Il18rap |
| Teff_2_peak_2164 | 4.10338 | 5.46071 | 2.50376 | 116 | Distal Intergenic | 130932816 | 130940115 | Il19 |
| Teff_2_peak_22207 | 5.31046 | 7.1641 | 3.94449 | 107 | Promoter (2-3kb) | 24186476 | 24193568 | Il1f9 |
| Teff_2_peak_610 | 3.31903 | 3.7386 | 1.30114 | 197 | Distal Intergenic | 40225080 | 40317257 | Il1r1 |
| Teff_2_peak_611 | 6.88964 | 10.3039 | 6.73513 | 111 | Distal Intergenic | 40225080 | 40317257 | Il1r1 |
| Teff_2_peak_612 | 3.2827 | 4.09797 | 1.41255 | 107 | Distal Intergenic | 40225080 | 40317257 | Il1r1 |
| Teff_2_peak_613 | 6.02325 | 8.603 | 5.19669 | 112 | Intron (ENSMUST0000 | 40225080 | 40317257 | Il1r1 |
| Teff_2_peak_614 | 6.45827 | 10.1292 | 6.5638 | 113 | Intron (ENSMUST0000 | 40266657 | 40277178 | Il1r1 |
| Teff_2_peak_615 | 6.63807 | 9.71386 | 6.24177 | 220 | Intron (ENSMUST0000 | 40266657 | 40277178 | Il1r1 |
| Teff_2_peak_608 | 3.40043 | 3.8861 | 1.41255 | 229 | Distal Intergenic | 40074079 | 40112136 | Il1r2 |
| Teff_2_peak_609 | 4.10242 | 5.458 | 2.50376 | 100 | Promoter (2-3kb) | 40084698 | 40125231 | Il1r2 |
| Teff_2_peak_16139 | 4.92405 | 6.9017 | 3.72503 | 114 | Intron (ENSMUST0000 | 26727042 | 26728228 | Il1rap |
| Teff_2_peak_16138 | 5.31046 | 7.1641 | 3.94449 | 61 | Promoter (<=1kb) | 26722434 | 26730117 | Il1rap |
| Teff_2_peak_42068 | 6.5654 | 9.97402 | 6.43068 | 165 | Intron (ENSMUST0000 | 86747242 | 87890235 | Il1rapl1 |
| Teff_2_peak_42069 | 9.78778 | 16.2169 | 12.225 | 151 | Intron (ENSMUST0000 | 86747242 | 87890235 | Il1rapl1 |
| Teff_2_peak_621 | 4.10338 | 5.46071 | 2.50376 | 179 | Distal Intergenic | 40429570 | 40446723 | Il1rl1 |
| Teff_2_peak_622 | 4.03008 | 5.25891 | 2.45255 | 379 | Distal Intergenic | 40429570 | 40446723 | Il1rl1 |
| Teff_2_peak_623 | 7.11063 | 10.7008 | 7.11598 | 118 | Distal Intergenic | 40429570 | 40446723 | Il1rl1 |
| Teff_2_peak_625 | 4.10338 | 5.46071 | 2.50376 | 108 | Intron (ENSMUST0000 | 40440628 | 40465396 | Il1rl1 |
| Teff_2_peak_624 | 5.74473 | 8.40906 | 5.0264 | 118 | Promoter (1-2kb) | 40429570 | 40446723 | Il1rl1 |
| Teff_2_peak_616 | 3.31903 | 3.7386 | 1.30114 | 126 | Intron (ENSMUST0000 | 40325611 | 40329171 | Il1rl2 |
| Teff_2_peak_25398 | 7.92048 | 12.4712 | 8.72434 | 127 | Distal Intergenic | 37120523 | 37125959 | Il2 |
| Teff_2_peak_25397 | 13.9482 | 25.8603 | 21.377 | 148 | Promoter (<=1kb) | 37120523 | 37125959 | Il2 |
| Teff_2_peak_3715 | 8.94221 | 16.1221 | 12.1431 | 175 | Distal Intergenic | 19712570 | 19760053 | Il20ra |
| Teff_2_peak_41163 | 5.97426 | 8.41633 | 5.0264 | 186 | Intron (ENSMUST0000 | 100465708 | 100486385 | Il20rb |
| Teff_2_peak_41164 | 5.74473 | 8.40906 | 5.0264 | 170 | Intron (ENSMUST0000 | 100465708 | 100486385 | Il20rb |
| Teff_2_peak_37036 | 6.44812 | 9.64692 | 6.19689 | 133 | Intron (ENSMUST0000 | 125603537 | 125633570 | Il21r |
| Teff_2_peak_5566 | 4.92405 | 6.9017 | 3.72503 | 120 | Distal Intergenic | 118204942 | 118210047 | Il22 |
| Teff_2_peak_5567 | 6.63807 | 9.71386 | 6.24177 | 112 | Distal Intergenic | 118204942 | 118210047 | Il22 |
| Teff_2_peak_29576 | 6.84411 | 12.6561 | 8.90588 | 138 | Distal Intergenic | 135728172 | 135752140 | Il22ra1 |
| Teff_2_peak_3712 | 5.31046 | 7.1641 | 3.94449 | 247 | Distal Intergenic | 19621998 | 19634681 | Il22ra2 |
| Teff_2_peak_33546 | 8.20485 | 13.2458 | 9.43183 | 133 | Intron (ENSMUST0000 | 67422932 | 67491855 | Il23r |
| Teff_2_peak_33547 | 6.5654 | 9.97402 | 6.43068 | 160 | Intron (ENSMUST0000 | 67422932 | 67491855 | Il23r |
| Teff_2_peak_38701 | 5.64773 | 8.82844 | 5.41566 | 106 | Intron (ENSMUST0000 | 84041769 | 84042540 | Il27ra |
| Teff_2_peak_21980 | 8.94221 | 16.1221 | 12.1431 | 116 | Promoter (2-3kb) | 11642807 | 11693193 | Il2ra |
| Teff_2_peak_6698 | 5.74473 | 8.40906 | 5.0264 | 110 | Distal Intergenic | 54265303 | 54267277 | Il3 |
| Teff_2_peak_31914 | 8.39674 | 13.6699 | 9.83431 | 140 | Intron (ENSMUST0000 | 123480401 | 123489489 | Il31 |
| Teff_2_peak_11911 | 6.95506 | 11.272 | 7.64474 | 415 | Intron (ENSMUST0000 | 112525846 | 112532344 | Il31ra |
| Teff_2_peak_21233 | 4.10338 | 5.46071 | 2.50376 | 101 | Distal Intergenic | 29951816 | 29952813 | Il33 |
| Teff_2_peak_39127 | 3.76453 | 4.61687 | 1.89299 | 226 | Promoter (<=1kb) | 110755525 | 110805924 | Il34 |
| Teff_2_peak_6683 | 11.7062 | 23.7197 | 19.3462 | 153 | Distal Intergenic | 53612460 | 53618669 | Il4 |
| Teff_2_peak_6681 | 3.96024 | 5.07784 | 2.28876 | 89 | Intron (ENSMUST0000 | 53602982 | 53617224 | Il4 |
| Teff_2_peak_6682 | 5.56489 | 7.64554 | 4.39188 | 151 | Promoter (<=1kb) | 53602982 | 53617224 | Il4 |
| Teff_2_peak_24886 | 3.31903 | 3.7386 | 1.30114 | 47 | Distal Intergenic | 7573182 | 7613760 | Il7 |
| Teff_2_peak_24887 | 6.5654 | 9.97402 | 6.43068 | 124 | Distal Intergenic | 7573182 | 7613760 | Il7 |
| Teff_2_peak_24885 | 4.92405 | 6.9017 | 3.72503 | 102 | Intron (ENSMUST0000 | 7573182 | 7613760 | Il7 |
| Teff_2_peak_14147 | 3.28194 | 4.09582 | 1.41255 | 89 | Intron (ENSMUST0000 | 9516409 | 9530176 | Il7r |
| Teff_2_peak_14146 | 13.9515 | 25.8697 | 21.377 | 140 | Intron (ENSMUST0000 | 9511898 | 9529766 | Il7r |
| Teff_2_peak_14145 | 6.63807 | 9.71386 | 6.24177 | 108 | Intron (ENSMUST0000 | 9511898 | 9529766 | Il7r |
| Teff_2_peak_10937 | 5.48147 | 7.48443 | 4.24006 | 110 | Intron (ENSMUST0000 | 56479277 | 56482246 | Il9 |
| Teff_2_peak_16334 | 4.83609 | 6.65819 | 3.594 | 171 | Intron (ENSMUST0000 | 36694038 | 36726741 | Ildr1 |
| Teff_2_peak_39853 | 5.74473 | 8.40906 | 5.0264 | 145 | Promoter (<=1kb) | 21377126 | 21405090 | Ilf3 |
| Teff_2_peak_39854 | 4.51743 | 5.8824 | 2.89265 | 142 | Promoter (1-2kb) | 21384218 | 21388030 | Ilf3 |
| Teff_2_peak_5092 | 4.10338 | 5.46071 | 2.50376 | 107 | Downstream (1-2kb) | 95387667 | 95392967 | Socs2 |
| Teff_2_peak_19113 | 7.11063 | 10.7008 | 7.11598 | 103 | Distal Intergenic | 87107679 | 87137839 | Socs5 |
| Teff_2_peak_19114 | 5.33286 | 7.48319 | 4.23909 | 127 | Distal Intergenic | 87107679 | 87137839 | Socs5 |
| Teff_2_peak_20822 | 10.5407 | 17.8476 | 13.755 | 160 | Intron (ENSMUST0000 | 88665224 | 88758491 | Socs6 |
| Teff_2_peak_20820 | 3.98284 | 4.81771 | 2.04745 | 210 | Intron (ENSMUST0000 | 88665224 | 88758491 | Socs6 |
| Teff_2_peak_20821 | 6.02325 | 8.603 | 5.19669 | 103 | Promoter (1-2kb) | 88665224 | 88758491 | Socs6 |

| Table S6: Narrow peaks ChIPseeker annotation of HDAC8 in Teff cells (3) | | | | | | | | |
|---|---|---|---|---|---|---|---|---|
| name | signalValue | '-log10pvalue | '-log10qvalue | peak_summit | annotation | geneStart | geneEnd | geneName |
| Teff_3_peak_7464 | 7.05578 | 11.6449 | 8.1365 | 110 | Distal Intergenic | 82176657 | 82305690 | Ccl1 |
| Teff_3_peak_7465 | 5.67971 | 7.87313 | 4.72643 | 154 | Distal Intergenic | 82176657 | 82305690 | Ccl1 |
| Teff_3_peak_7462 | 5.67971 | 7.87313 | 4.72643 | 75 | Intron (ENSMUST000 | 82176659 | 82179812 | Ccl1 |
| Teff_3_peak_7463 | 13.2276 | 25.2647 | 21.1312 | 122 | Intron (ENSMUST000 | 82176659 | 82179812 | Ccl1 |
| Teff_3_peak_7460 | 5.82851 | 8.65643 | 5.39801 | 90 | Promoter (2-3kb) | 82057823 | 82062955 | Ccl11 |
| Teff_3_peak_40601 | 4.96975 | 6.57871 | 3.52008 | 72 | Distal Intergenic | 94810453 | 94812036 | Ccl17 |
| Teff_3_peak_1401 | 6.38967 | 9.21819 | 5.92099 | 141 | Distal Intergenic | 83116766 | 83119166 | Ccl20 |
| Teff_3_peak_1402 | 7.15447 | 11.2639 | 7.81453 | 89 | Distal Intergenic | 83116766 | 83119166 | Ccl20 |
| Teff_3_peak_1403 | 4.40986 | 6.4949 | 3.4655 | 141 | Distal Intergenic | 83116793 | 83119167 | Ccl20 |
| Teff_3_peak_1404 | 6.05378 | 8.89083 | 5.62235 | 121 | Distal Intergenic | 83116793 | 83119167 | Ccl20 |
| Teff_3_peak_40599 | 12.2732 | 22.9215 | 18.8843 | 134 | Distal Intergenic | 94745590 | 94751699 | Ccl22 |
| Teff_3_peak_38940 | 11.4124 | 19.8174 | 15.8785 | 142 | Intron (ENSMUST000 | 4349588 | 4357993 | Ccl25 |
| Teff_3_peak_12391 | 5.29103 | 8.14461 | 4.91462 | 73 | Intron (ENSMUST000 | 119649516 | 119651156 | Ccl28 |
| Teff_3_peak_12390 | 8.81973 | 15.3473 | 11.6033 | 94 | Promoter (1-2kb) | 119623819 | 119654359 | Ccl28 |
| Teff_3_peak_12392 | 5.29103 | 8.14461 | 4.91462 | 88 | Promoter (1-2kb) | 119649516 | 119651156 | Ccl28 |
| Teff_3_peak_7484 | 5.67971 | 7.87313 | 4.72643 | 75 | Promoter (1-2kb) | 83647844 | 83649355 | Ccl3 |
| Teff_3_peak_7479 | 5.50344 | 7.75885 | 4.64312 | 112 | Promoter (<=1kb) | 83525778 | 83530518 | Ccl5 |
| Teff_3_peak_7482 | 7.09964 | 10.6086 | 7.18149 | 163 | Intron (ENSMUST000 | 83587882 | 83593087 | Ccl6 |
| Teff_3_peak_7481 | 4.25978 | 5.34154 | 2.48427 | 84 | Promoter (<=1kb) | 83587882 | 83593087 | Ccl6 |
| Teff_3_peak_7461 | 4.40986 | 6.4949 | 3.4655 | 223 | Distal Intergenic | 82115185 | 82116799 | Ccl8 |
| Teff_3_peak_7480a | 9.98583 | 16.6443 | 12.8592 | 155 | Distal Intergenic | 83575318 | 83577142 | Ccl9 |
| Teff_3_peak_7480b | 11.7189 | 20.902 | 16.9364 | 544 | Distal Intergenic | 83575318 | 83577142 | Ccl9 |
| Teff_3_peak_43696 | 6.66115 | 10.2576 | 6.85385 | 243 | Distal Intergenic | 123977243 | 123978408 | Ccr1l1 |
| Teff_3_peak_43410 | 4.92235 | 6.48454 | 3.4655 | 116 | Intron (ENSMUST000 | 114491779 | 114496038 | Ccr4 |
| Teff_3_peak_43697 | 3.52735 | 4.9183 | 2.11459 | 41 | Intron (ENSMUST000 | 124128748 | 124147699 | Ccr5 |
| Teff_3_peak_18293 | 5.52054 | 7.80724 | 4.68311 | 105 | Promoter (<=1kb) | 8236043 | 8256108 | Ccr6 |
| Teff_3_peak_18294 | 7.8096 | 12.0402 | 8.49867 | 99 | Promoter (<=1kb) | 8236212 | 8257141 | Ccr6 |
| Teff_3_peak_18292 | 5.82851 | 8.65643 | 5.39801 | 102 | Promoter (1-2kb) | 8236043 | 8256108 | Ccr6 |
| Teff_3_peak_43588 | 15.2257 | 28.6964 | 24.4616 | 292 | Distal Intergenic | 120092114 | 120094906 | Ccr8 |
| Teff_3_peak_36761 | 5.8412 | 9.13862 | 5.86439 | 90 | Distal Intergenic | 24743983 | 24760311 | Cd177 |
| Teff_3_peak_12052 | 4.96975 | 6.57871 | 3.52008 | 80 | Distal Intergenic | 102693558 | 102706955 | Cd180 |
| Teff_3_peak_12053 | 9.70021 | 17.2565 | 13.4304 | 95 | Distal Intergenic | 102693558 | 102706955 | Cd180 |
| Teff_3_peak_26925 | 8.56535 | 14.3858 | 10.7187 | 97 | Downstream (2-3kb) | 86995834 | 86997947 | Cd1d1 |
| Teff_3_peak_26924 | 5.29184 | 8.14792 | 4.91462 | 82 | Distal Intergenic | 86986551 | 86989780 | Cd1d2 |
| Teff_3_peak_27103 | 15.8202 | 30.4076 | 26.1215 | 105 | Distal Intergenic | 101275899 | 101287939 | Cd2 |
| Teff_3_peak_17176 | 8.46364 | 13.5312 | 9.89627 | 98 | Distal Intergenic | 45392245 | 45400312 | Cd200 |
| Teff_3_peak_17177 | 26.475 | 127.639 | 121.661 | 187 | Distal Intergenic | 45392245 | 45400312 | Cd200 |
| Teff_3_peak_17178 | 7.93775 | 13.4733 | 9.85683 | 82 | Promoter (2-3kb) | 45392245 | 45400312 | Cd200 |
| Teff_3_peak_17172 | 3.52735 | 4.9183 | 2.11459 | 76 | Intron (ENSMUST000 | 44867097 | 44915840 | Cd200r2 |
| Teff_3_peak_17171 | 6.60413 | 10.0602 | 6.67267 | 89 | Promoter (2-3kb) | 44867097 | 44915840 | Cd200r2 |
| Teff_3_peak_17170 | 6.17381 | 9.86754 | 6.4921 | 88 | Promoter (1-2kb) | 44820728 | 44839150 | Cd200r4 |
| Teff_3_peak_38934 | 4.96975 | 6.57871 | 3.52008 | 91 | Downstream (1-2kb) | 3743801 | 3748927 | Cd209a |
| Teff_3_peak_38939 | 5.67971 | 7.87313 | 4.72643 | 73 | Promoter (<=1kb) | 4134098 | 4137516 | Cd209g |
| Teff_3_peak_21510 | 4.94256 | 7.11533 | 4.02182 | 94 | Intron (ENSMUST000 | 89197330 | 89207052 | Cd226 |
| Teff_3_peak_21511 | 5.29184 | 8.14792 | 4.91462 | 82 | Intron (ENSMUST000 | 89206101 | 89207338 | Cd226 |
| Teff_3_peak_3135 | 9.90619 | 17.7217 | 13.8788 | 119 | Intron (ENSMUST000 | 171579063 | 171585318 | Cd244a |
| Teff_3_peak_3003 | 3.56637 | 4.2029 | 1.62158 | 39 | Promoter (<=1kb) | 165788681 | 165871153 | Cd247 |
| Teff_3_peak_4399 | 18.4591 | 36.8848 | 32.4354 | 288 | Distal Intergenic | 43578284 | 43579197 | Cd24a |
| Teff_3_peak_4400 | 10.8244 | 18.9339 | 15.0531 | 102 | Distal Intergenic | 43578284 | 43579197 | Cd24a |
| Teff_3_peak_35935 | 8.67514 | 13.9984 | 10.3379 | 89 | Distal Intergenic | 125232622 | 125237010 | Cd27 |
| Teff_3_peak_986 | 6.38967 | 9.21819 | 5.92099 | 91 | Intron (ENSMUST000 | 60746358 | 60773359 | Cd28 |
| Teff_3_peak_31037 | 6.17286 | 9.86367 | 6.4921 | 85 | Intron (ENSMUST000 | 17782016 | 17835696 | Cd36 |
| Teff_3_peak_42080 | 9.47627 | 16.3962 | 12.6294 | 135 | Promoter (<=1kb) | 44969572 | 44980431 | Cd3g |
| Teff_3_peak_35931 | 8.23619 | 27.6783 | 23.4732 | 124 | Promoter (2-3kb) | 124867333 | 124888199 | Cd4 |
| Teff_3_peak_24047 | 30.371 | 102.493 | 96.9088 | 173 | Promoter (1-2kb) | 102811141 | 102901665 | Cd44 |
| Teff_3_peak_3678 | 11.4639 | 21.1961 | 17.2103 | 113 | Intron (ENSMUST000 | 195041835 | 195069114 | Cd46 |
| Teff_3_peak_17264 | 11.3594 | 19.708 | 15.7709 | 279 | Distal Intergenic | 49866833 | 49911091 | Cd47 |
| Teff_3_peak_17265 | 9.70021 | 17.2565 | 13.4304 | 106 | Distal Intergenic | 49866833 | 49911091 | Cd47 |
| Teff_3_peak_17261 | 3.85241 | 4.62535 | 1.99578 | 101 | Intron (ENSMUST000 | 49855366 | 49911091 | Cd47 |
| Teff_3_peak_17262 | 7.0547 | 11.6405 | 8.1365 | 100 | Promoter (1-2kb) | 49866833 | 49911091 | Cd47 |
| Teff_3_peak_17263 | 6.17381 | 9.86754 | 6.4921 | 89 | Promoter (2-3kb) | 49866833 | 49911091 | Cd47 |
| Teff_3_peak_27198 | 8.32644 | 13.6072 | 9.9597 | 100 | Distal Intergenic | 106759921 | 106790149 | Cd53 |
| Teff_3_peak_2412 | 5.29184 | 8.14792 | 4.91462 | 96 | Distal Intergenic | 130419601 | 130422740 | Cd55b |
| Teff_3_peak_2413 | 3.85241 | 4.62535 | 1.99578 | 187 | Distal Intergenic | 130419601 | 130422740 | Cd55b |
| Teff_3_peak_2414 | 7.48606 | 11.558 | 8.09739 | 103 | Promoter (1-2kb) | 130388537 | 130423009 | Cd55b |
| Teff_3_peak_24073a | 4.55565 | 6.77297 | 3.70269 | 69 | Intron (ENSMUST000 | 104095801 | 104115349 | Cd59a |
| Teff_3_peak_24073b | 6.66447 | 14.475 | 10.807 | 271 | Intron (ENSMUST000 | 104095801 | 104115349 | Cd59a |
| Teff_3_peak_21586 | 3.52789 | 4.92049 | 2.11459 | 76 | Intron (ENSMUST000 | 10794564 | 10829856 | Cd6 |
| Teff_3_peak_36024 | 8.8055 | 15.0582 | 11.36 | 203 | Distal Intergenic | 129267325 | 129275436 | Cd69 |
| Teff_3_peak_8219 | 7.05578 | 11.6449 | 8.1365 | 212 | Promoter (1-2kb) | 121036747 | 121039418 | Cd7 |
| Teff_3_peak_7903 | 3.82208 | 4.74621 | 2.11142 | 129 | Downstream (<1kb) | 106311344 | 106314529 | Cd79b |
| Teff_3_peak_38879 | 13.3223 | 24.5518 | 20.4528 | 98 | Intron (ENSMUST000 | 143052739 | 143067934 | Cd81 |
| Teff_3_peak_17020 | 7.09964 | 10.6086 | 7.18149 | 79 | Intron (ENSMUST000 | 36603869 | 36666077 | Cd86 |
| Teff_3_peak_17021 | 8.25516 | 13.7649 | 10.1104 | 126 | Intron (ENSMUST000 | 36603869 | 36666077 | Cd86 |
| Teff_3_peak_17018 | 7.93775 | 13.4733 | 9.85683 | 216 | Intron (ENSMUST000 | 36603897 | 36604519 | Cd86 |
| Teff_3_peak_17019 | 6.17381 | 9.86754 | 6.4921 | 188 | Intron (ENSMUST000 | 36603897 | 36604519 | Cd86 |
| Teff_3_peak_34917 | 3.33058 | 4.23708 | 1.63193 | 80 | Exon (ENSMUST000( | 71322788 | 71337494 | Cd8b1 |
| Teff_3_peak_34916 | 7.09964 | 10.6086 | 7.18149 | 91 | Promoter (<=1kb) | 71322788 | 71337494 | Cd8b1 |
| Teff_3_peak_35939 | 6.51854 | 11.7023 | 8.19319 | 114 | Distal Intergenic | 125462116 | 125463024 | Cd9 |
| Teff_3_peak_35940 | 5.10989 | 7.47587 | 4.37033 | 85 | Distal Intergenic | 125462116 | 125463024 | Cd9 |
| Teff_3_peak_35941 | 6.74163 | 9.96522 | 6.58591 | 87 | Intron (ENSMUST000 | 125460266 | 125494791 | Cd9 |
| Teff_3_peak_21973 | 5.67971 | 7.87313 | 4.72643 | 75 | Distal Intergenic | 34300075 | 34316677 | Fas |
| Teff_3_peak_2911 | 4.40986 | 6.4949 | 3.4655 | 77 | Distal Intergenic | 161780689 | 161788495 | Fasl |
| Teff_3_peak_2912 | 8.81973 | 15.3473 | 11.6033 | 123 | Distal Intergenic | 161780689 | 161788495 | Fasl |
| Teff_3_peak_2910 | 6.17286 | 9.86367 | 6.4921 | 104 | Downstream (2-3kb) | 161781422 | 161788358 | Fasl |

| | | | | | | | | |
|---|---|---|---|---|---|---|---|---|
| Teff_3_peak_5832 | 5.82851 | 8.65643 | 5.39801 | 170 | Distal Intergenic | 118441046 | 118445892 | Ifng |
| Teff_3_peak_5833 | 5.29184 | 8.14792 | 4.91462 | 81 | Distal Intergenic | 118441046 | 118445892 | Ifng |
| Teff_3_peak_5834 | 4.25978 | 5.34154 | 2.48427 | 89 | Distal Intergenic | 118441046 | 118445892 | Ifng |
| Teff_3_peak_5838 | 13.3223 | 24.5518 | 20.4528 | 132 | Distal Intergenic | 118502035 | 118556525 | Ifngas1 |
| Teff_3_peak_5839 | 4.40275 | 5.62147 | 2.74648 | 162 | Distal Intergenic | 118502035 | 118556525 | Ifngas1 |
| Teff_3_peak_5840 | 7.09784 | 10.8113 | 7.37327 | 83 | Distal Intergenic | 118502035 | 118556525 | Ifngas1 |
| Teff_3_peak_5841 | 9.93949 | 16.5488 | 12.7662 | 97 | Distal Intergenic | 118502035 | 118556525 | Ifngas1 |
| Teff_3_peak_5837 | 4.95309 | 6.66767 | 3.59937 | 80 | Intron (ENSMUST000 | 118502035 | 118555352 | Ifngas1 |
| Teff_3_peak_5835 | 15.8731 | 31.5738 | 27.2616 | 92 | Intron (ENSMUST000 | 118502035 | 118555352 | Ifngas1 |
| Teff_3_peak_5836 | 4.40986 | 6.4949 | 3.4655 | 85 | Intron (ENSMUST000 | 118502035 | 118555352 | Ifngas1 |
| Teff_3_peak_2417 | 13.3223 | 24.5518 | 20.4528 | 162 | Distal Intergenic | 131019845 | 131024974 | Il10 |
| Teff_3_peak_18006 | 5.67971 | 7.87313 | 4.72643 | 112 | Intron (ENSMUST000 | 91406444 | 91414632 | Il10rb |
| Teff_3_peak_26617 | 5.29184 | 8.14792 | 4.91462 | 115 | Distal Intergenic | 68690644 | 68698547 | Il12a |
| Teff_3_peak_26618 | 4.99586 | 7.11293 | 4.0208 | 88 | Distal Intergenic | 68690644 | 68698547 | Il12a |
| Teff_3_peak_26619a | 4.94256 | 7.11533 | 4.02182 | 101 | Distal Intergenic | 68694021 | 68698547 | Il12a |
| Teff_3_peak_26619b | 14.1993 | 26.3307 | 22.1625 | 282 | Distal Intergenic | 68694021 | 68698547 | Il12a |
| Teff_3_peak_34868 | 4.95309 | 6.66767 | 3.59937 | 145 | Intron (ENSMUST000 | 67292018 | 67376188 | Il12rb2 |
| Teff_3_peak_34867 | 3.52789 | 4.92049 | 2.11459 | 22 | Intron (ENSMUST000 | 67291318 | 67339694 | Il12rb2 |
| Teff_3_peak_6955 | 6.82605 | 10.7251 | 7.29485 | 86 | Downstream (1-2kb) | 53631324 | 53634702 | Il13 |
| Teff_3_peak_44227 | 5.29103 | 8.14461 | 4.91462 | 81 | Intron (ENSMUST000 | 36112110 | 36171259 | Il13ra1 |
| Teff_3_peak_44228 | 6.05378 | 8.89083 | 5.62235 | 96 | Intron (ENSMUST000 | 36112110 | 36171259 | Il13ra1 |
| Teff_3_peak_45287 | 17.6395 | 35.9109 | 31.4793 | 176 | Distal Intergenic | 147383478 | 147403832 | Il13ra2 |
| Teff_3_peak_45288 | 6.17286 | 9.86367 | 6.4921 | 85 | Intron (ENSMUST000 | 147383478 | 147403832 | Il13ra2 |
| Teff_3_peak_22697 | 6.17381 | 9.86754 | 6.4921 | 86 | Distal Intergenic | 11705290 | 11734317 | Il15ra |
| Teff_3_peak_22698 | 4.96975 | 6.57871 | 3.52008 | 84 | Intron (ENSMUST000 | 11705848 | 11733249 | Il15ra |
| Teff_3_peak_37834 | 6.17381 | 9.86754 | 6.4921 | 80 | Exon (ENSMUST0000 | 83649576 | 83655325 | Il16 |
| Teff_3_peak_37838 | 5.10989 | 7.47587 | 4.37033 | 91 | Promoter (1-2kb) | 83722510 | 83739937 | Il16 |
| Teff_3_peak_270 | 6.17381 | 9.86754 | 6.4921 | 205 | Distal Intergenic | 20730905 | 20734496 | Il17a |
| Teff_3_peak_12765 | 5.29184 | 8.14792 | 4.91462 | 89 | Intron (ENSMUST000 | 27087765 | 27101491 | Il17rd |
| Teff_3_peak_12766 | 5.99575 | 9.198 | 5.92099 | 84 | Promoter (1-2kb) | 27087765 | 27101491 | Il17rd |
| Teff_3_peak_42220 | 5.67971 | 7.87313 | 4.72643 | 82 | Distal Intergenic | 50575273 | 50581837 | Il18 |
| Teff_3_peak_38106 | 3.50663 | 4.83586 | 2.11459 | 37 | Promoter (<=1kb) | 102015315 | 102018690 | Il18bp |
| Teff_3_peak_642 | 4.99071 | 6.59507 | 3.53514 | 73 | Intron (ENSMUST000 | 40466006 | 40500854 | Il18r1 |
| Teff_3_peak_644 | 4.99586 | 7.11293 | 4.0208 | 146 | Intron (ENSMUST000 | 40515362 | 40551705 | Il18rap |
| Teff_3_peak_645 | 5.82851 | 8.65643 | 5.39801 | 128 | Intron (ENSMUST000 | 40515362 | 40551705 | Il18rap |
| Teff_3_peak_643 | 4.25978 | 5.34154 | 2.48427 | 88 | Intron (ENSMUST000 | 40515362 | 40551705 | Il18rap |
| Teff_3_peak_22890 | 5.67971 | 7.87313 | 4.72643 | 80 | Distal Intergenic | 24291196 | 24293820 | Il1f10 |
| Teff_3_peak_637 | 10.9465 | 19.3181 | 15.4007 | 138 | Distal Intergenic | 40225080 | 40317257 | Il1r1 |
| Teff_3_peak_638 | 10.2111 | 17.2399 | 13.4304 | 81 | Distal Intergenic | 40225080 | 40317257 | Il1r1 |
| Teff_3_peak_639 | 11.657 | 20.7773 | 16.8176 | 155 | Intron (ENSMUST000 | 40266657 | 40277178 | Il1r1 |
| Teff_3_peak_633a | 15.3909 | 29.1494 | 24.9098 | 127 | Distal Intergenic | 40074079 | 40112136 | Il1r2 |
| Teff_3_peak_633b | 26.5337 | 57.3123 | 52.4599 | 554 | Distal Intergenic | 40074079 | 40112136 | Il1r2 |
| Teff_3_peak_634 | 4.96975 | 6.57871 | 3.52008 | 92 | Intron (ENSMUST000 | 40084698 | 40125231 | Il1r2 |
| Teff_3_peak_635 | 3.52735 | 4.9183 | 2.11459 | 70 | Promoter (2-3kb) | 40084698 | 40125231 | Il1r2 |
| Teff_3_peak_16826 | 9.65268 | 18.2254 | 14.3607 | 91 | Distal Intergenic | 26581704 | 26725264 | Il1rap |
| Teff_3_peak_16827 | 7.4938 | 11.9096 | 8.38827 | 102 | Intron (ENSMUST000 | 26624156 | 26715068 | Il1rap |
| Teff_3_peak_16828 | 6.17381 | 9.86754 | 6.4921 | 90 | Intron (ENSMUST000 | 26722434 | 26730117 | Il1rap |
| Teff_3_peak_16829 | 4.99586 | 7.11293 | 4.0208 | 81 | Intron (ENSMUST000 | 26722434 | 26730117 | Il1rap |
| Teff_3_peak_16830 | 5.29184 | 8.14792 | 4.91462 | 109 | Intron (ENSMUST000 | 26727042 | 26728228 | Il1rap |
| Teff_3_peak_44721 | 6.66115 | 10.2576 | 6.85385 | 111 | Distal Intergenic | 86769118 | 88115645 | Il1rapl1 |
| Teff_3_peak_44718 | 5.29184 | 8.14792 | 4.91462 | 145 | Intron (ENSMUST000 | 86747242 | 87890235 | Il1rapl1 |
| Teff_3_peak_44719 | 16.6529 | 32.4056 | 28.0653 | 176 | Intron (ENSMUST000 | 86747242 | 87890235 | Il1rapl1 |
| Teff_3_peak_45180 | 5.29184 | 8.14792 | 4.91462 | 103 | Distal Intergenic | 137570608 | 138846946 | Il1rapl2 |
| Teff_3_peak_641 | 11.236 | 19.3773 | 15.4563 | 122 | Distal Intergenic | 40429570 | 40446723 | Il1rl1 |
| Teff_3_peak_22891 | 8.98884 | 14.5141 | 10.8433 | 144 | Distal Intergenic | 24345348 | 24346354 | Il1rn |
| Teff_3_peak_26089 | 19.0626 | 38.9834 | 34.4849 | 127 | Distal Intergenic | 37120523 | 37125959 | Il2 |
| Teff_3_peak_26088 | 9.70021 | 17.2565 | 13.4304 | 95 | Promoter (<=1kb) | 37120523 | 37125959 | Il2 |
| Teff_3_peak_3987 | 7.70481 | 12.4996 | 8.93984 | 127 | Distal Intergenic | 19712570 | 19760053 | Il20ra |
| Teff_3_peak_3988 | 3.52735 | 4.9183 | 2.11459 | 120 | Distal Intergenic | 19712570 | 19760053 | Il20ra |
| Teff_3_peak_30427 | 11.0095 | 21.4816 | 17.474 | 107 | Distal Intergenic | 135728172 | 135752140 | Il22ra1 |
| Teff_3_peak_3983 | 24.6915 | 53.9818 | 49.1824 | 276 | Distal Intergenic | 19621998 | 19634681 | Il22ra2 |
| Teff_3_peak_34871 | 9.7017 | 17.2626 | 13.4304 | 113 | Intron (ENSMUST000 | 67422932 | 67491855 | Il23r |
| Teff_3_peak_38554 | 5.99008 | 8.44561 | 5.20726 | 187 | Promoter (1-2kb) | 126589010 | 126594941 | Il27 |
| Teff_3_peak_22695 | 6.58765 | 10.0385 | 6.65643 | 108 | Distal Intergenic | 11642807 | 11693193 | Il2ra |
| Teff_3_peak_22696 | 9.11469 | 14.9493 | 11.2708 | 192 | Promoter (2-3kb) | 11642807 | 11693193 | Il2ra |
| Teff_3_peak_15838 | 3.85241 | 4.62535 | 1.99578 | 133 | Distal Intergenic | 78479256 | 78495271 | Il2rb |
| Teff_3_peak_6965 | 6.17381 | 9.86754 | 6.4921 | 80 | Distal Intergenic | 54265303 | 54267277 | Il3 |
| Teff_3_peak_6966 | 5.29184 | 8.14792 | 4.91462 | 80 | Promoter (1-2kb) | 54265303 | 54267277 | Il3 |
| Teff_3_peak_33090 | 5.70619 | 7.92666 | 4.77616 | 78 | Intron (ENSMUST000 | 123480401 | 123489489 | Il31 |
| Teff_3_peak_12248 | 7.05578 | 11.6449 | 8.1365 | 105 | Intron (ENSMUST000 | 112522795 | 112580662 | Il31ra |
| Teff_3_peak_12247 | 6.86222 | 10.2574 | 6.85385 | 80 | Intron (ENSMUST000 | 112523557 | 112562074 | Il31ra |
| Teff_3_peak_21910 | 3.52789 | 4.92049 | 2.11459 | 79 | Distal Intergenic | 29951816 | 29952813 | Il33 |
| Teff_3_peak_40880 | 7.70481 | 12.4996 | 8.93984 | 95 | Intron (ENSMUST000 | 110755236 | 110790888 | Il34 |
| Teff_3_peak_6954 | 21.1279 | 46.6864 | 42.0369 | 316 | Distal Intergenic | 53612460 | 53618669 | Il4 |
| Teff_3_peak_25565 | 5.29184 | 8.14792 | 4.91462 | 103 | Distal Intergenic | 7573182 | 7613760 | Il7 |
| Teff_3_peak_25566 | 6.17381 | 9.86754 | 6.4921 | 80 | Distal Intergenic | 7573182 | 7613760 | Il7 |
| Teff_3_peak_14676 | 5.29103 | 8.14461 | 4.91462 | 100 | Intron (ENSMUST000 | 9516409 | 9530176 | Il7r |
| Teff_3_peak_14675 | 7.0547 | 11.6405 | 8.1365 | 99 | Intron (ENSMUST000 | 9511898 | 9529766 | Il7r |
| Teff_3_peak_14674 | 6.66115 | 10.2576 | 6.85385 | 115 | Intron (ENSMUST000 | 9511898 | 9529766 | Il7r |
| Teff_3_peak_11239 | 4.8603 | 6.72367 | 3.65433 | 72 | Downstream (1-2kb) | 56479277 | 56482246 | Il9 |
| Teff_3_peak_16580 | 6.09765 | 8.96271 | 5.69345 | 105 | Distal Intergenic | 10782240 | 10785536 | Socs1 |
| Teff_3_peak_16581 | 12.0694 | 21.3279 | 17.3227 | 94 | Distal Intergenic | 10782240 | 10785536 | Socs1 |
| Teff_3_peak_16579 | 5.54714 | 7.61096 | 4.50306 | 122 | Intron (ENSMUST000 | 10782240 | 10785536 | Socs1 |
| Teff_3_peak_21504 | 6.66115 | 10.2576 | 6.85385 | 104 | Intron (ENSMUST000 | 88665224 | 88758491 | Socs6 |

| Table S7: Narrow peaks ChIPseeker annotation of HDAC8 in Treg cells (1) | | | | | | | | |
|---|---|---|---|---|---|---|---|---|
| name | signalValue | '-log10pvalue | '-log10qvalue | peak_summit | annotation | geneStart | geneEnd | geneName |
| Treg_1_peak_12060 | 13.192 | 23.8427 | 19.8383 | 160 | Distal Intergenic | 82176657 | 82305690 | Ccl1 |
| Treg_1_peak_12057 | 6.89044 | 10.3071 | 7.07804 | 210 | Intron (ENSMUST000 | 82176657 | 82305690 | Ccl1 |
| Treg_1_peak_12058 | 3.08117 | 3.58522 | 1.34861 | 100 | Intron (ENSMUST000 | 82176657 | 82305690 | Ccl1 |
| Treg_1_peak_12056 | 4.74975 | 6.43314 | 3.57669 | 117 | Intron (ENSMUST000 | 82176657 | 82305690 | Ccl1 |
| Treg_1_peak_12053 | 8.47322 | 13.5001 | 10.0067 | 199 | Intron (ENSMUST000 | 82176659 | 82179812 | Ccl1 |
| Treg_1_peak_12054 | 26.19 | 56.4623 | 51.6429 | 212 | Intron (ENSMUST000 | 82176659 | 82179812 | Ccl1 |
| Treg_1_peak_12055 | 5.54138 | 7.86 | 4.82256 | 151 | Intron (ENSMUST000 | 82176659 | 82179812 | Ccl1 |
| Treg_1_peak_12052 | 4.65598 | 6.20215 | 3.43047 | 100 | Promoter (<=1kb) | 82176657 | 82179812 | Ccl1 |
| Treg_1_peak_12059 | 7.70293 | 11.9322 | 8.5491 | 173 | Promoter (1-2kb) | 82176657 | 82305690 | Ccl1 |
| Treg_1_peak_12048 | 3.95813 | 5.07252 | 2.42876 | 120 | Distal Intergenic | 82057823 | 82062955 | Ccl11 |
| Treg_1_peak_12049 | 3.1665 | 3.78996 | 1.3917 | 99 | Distal Intergenic | 82057823 | 82062955 | Ccl11 |
| Treg_1_peak_12050 | 6.26404 | 9.03921 | 5.90404 | 126 | Distal Intergenic | 82057823 | 82062955 | Ccl11 |
| Treg_1_peak_12046 | 4.6822 | 6.26545 | 3.49061 | 120 | Intron (ENSMUST000 | 82035571 | 82037453 | Ccl2 |
| Treg_1_peak_2292 | 3.95813 | 5.07252 | 2.42876 | 158 | Distal Intergenic | 83116793 | 83119167 | Ccl20 |
| Treg_1_peak_2293 | 5.39205 | 7.49389 | 4.54135 | 174 | Distal Intergenic | 83116793 | 83119167 | Ccl20 |
| Treg_1_peak_65213a | 5.45622 | 7.49968 | 4.54589 | 132 | Distal Intergenic | 94745590 | 94751699 | Ccl22 |
| Treg_1_peak_65213b | 5.58271 | 7.68039 | 4.71114 | 492 | Distal Intergenic | 94745590 | 94751699 | Ccl22 |
| Treg_1_peak_53662 | 4.38483 | 5.49927 | 2.81361 | 182 | Distal Intergenic | 135570580 | 135573049 | Ccl24 |
| Treg_1_peak_53661 | 4.91827 | 6.6116 | 3.75113 | 119 | Promoter (<=1kb) | 135570580 | 135573049 | Ccl24 |
| Treg_1_peak_62625 | 6.93264 | 10.4055 | 7.14268 | 204 | Promoter (1-2kb) | 4332259 | 4334807 | Ccl25 |
| Treg_1_peak_20110 | 5.5248 | 7.81796 | 4.82256 | 121 | Promoter (1-2kb) | 119623819 | 119654359 | Ccl28 |
| Treg_1_peak_20111 | 4.40907 | 5.64739 | 2.94543 | 146 | Promoter (2-3kb) | 119623819 | 119654359 | Ccl28 |
| Treg_1_peak_12086 | 4.38483 | 5.49927 | 2.81361 | 188 | Distal Intergenic | 83525778 | 83530518 | Ccl5 |
| Treg_1_peak_12091 | 9.24352 | 15.1056 | 11.5129 | 161 | Intron (ENSMUST000 | 83587882 | 83593087 | Ccl6 |
| Treg_1_peak_12090 | 5.54138 | 7.86 | 4.82256 | 125 | Promoter (<=1kb) | 83588395 | 83589875 | Ccl6 |
| Treg_1_peak_12047 | 4.75029 | 6.34321 | 3.56123 | 203 | Distal Intergenic | 82045712 | 82047525 | Ccl7 |
| Treg_1_peak_12051 | 4.62176 | 6.12093 | 3.35997 | 141 | Promoter (<=1kb) | 82115185 | 82116799 | Ccl8 |
| Treg_1_peak_12087 | 5.07187 | 6.73627 | 3.85677 | 183 | Distal Intergenic | 83575318 | 83577142 | Ccl9 |
| Treg_1_peak_12088 | 6.97382 | 10.4549 | 7.18992 | 150 | Distal Intergenic | 83575318 | 83577142 | Ccl9 |
| Treg_1_peak_70077 | 6.93264 | 10.4055 | 7.14268 | 114 | Distal Intergenic | 123977243 | 123978408 | Ccr1l1 |
| Treg_1_peak_70078 | 15.6601 | 31.0509 | 26.8041 | 199 | Distal Intergenic | 123977243 | 123978408 | Ccr1l1 |
| Treg_1_peak_70079 | 3.95813 | 5.07252 | 2.42876 | 183 | Distal Intergenic | 124101950 | 124113557 | Ccr2 |
| Treg_1_peak_70080 | 3.95813 | 5.07252 | 2.42876 | 141 | Distal Intergenic | 124101950 | 124113557 | Ccr2 |
| Treg_1_peak_69689 | 5.78315 | 8.45152 | 5.38751 | 134 | Distal Intergenic | 114491779 | 114496038 | Ccr4 |
| Treg_1_peak_69690 | 5.81222 | 8.12127 | 5.0776 | 151 | Promoter (<=1kb) | 114490316 | 114496562 | Ccr4 |
| Treg_1_peak_29260 | 4.62176 | 6.12093 | 3.35997 | 167 | Promoter (<=1kb) | 8236043 | 8256108 | Ccr6 |
| Treg_1_peak_12554 | 3.48778 | 4.38374 | 1.95309 | 104 | Distal Intergenic | 99144196 | 99155077 | Ccr7 |
| Treg_1_peak_69926 | 4.52563 | 5.9889 | 3.27461 | 215 | Distal Intergenic | 120092114 | 120094906 | Ccr8 |
| Treg_1_peak_70074 | 3.95813 | 5.07252 | 2.42876 | 235 | Intron (ENSMUST000 | 123774540 | 123783457 | Ccr9 |
| Treg_1_peak_70073 | 5.80591 | 8.48822 | 5.41921 | 145 | Promoter (<=1kb) | 123774540 | 123783457 | Ccr9 |
| Treg_1_peak_68636 | 4.62176 | 6.12093 | 3.35997 | 76 | Distal Intergenic | 78615546 | 78716253 | Cd109 |
| Treg_1_peak_68637 | 6.16235 | 8.9243 | 5.81442 | 192 | Distal Intergenic | 78615546 | 78716253 | Cd109 |
| Treg_1_peak_68638 | 5.5248 | 7.81796 | 4.82256 | 164 | Distal Intergenic | 78615546 | 78716253 | Cd109 |
| Treg_1_peak_32774 | 5.31504 | 7.17252 | 4.27037 | 112 | Distal Intergenic | 36725871 | 36726738 | Cd14 |
| Treg_1_peak_62482 | 5.02534 | 14.3214 | 10.771 | 138 | Promoter (2-3kb) | 140218541 | 140230982 | Cd163l1 |
| Treg_1_peak_59078 | 4.40907 | 5.64739 | 2.94543 | 125 | Distal Intergenic | 24743983 | 24760311 | Cd177 |
| Treg_1_peak_59076 | 8.136 | 14.7988 | 11.2302 | 187 | Downstream (1-2kb) | 24745127 | 24750821 | Cd177 |
| Treg_1_peak_59077 | 4.17919 | 5.66768 | 2.96525 | 211 | Promoter (1-2kb) | 24745127 | 24750821 | Cd177 |
| Treg_1_peak_19630 | 10.0344 | 17.9375 | 14.1933 | 149 | Distal Intergenic | 102693558 | 102706955 | Cd180 |
| Treg_1_peak_19631 | 9.24352 | 15.1056 | 11.5129 | 153 | Intron (ENSMUST000 | 102693611 | 102739504 | Cd180 |
| Treg_1_peak_62059 | 4.22248 | 5.33598 | 2.6712 | 149 | Promoter (1-2kb) | 126408451 | 126414889 | Cd19 |
| Treg_1_peak_43525 | 4.74975 | 6.43314 | 3.57669 | 126 | Distal Intergenic | 86986551 | 86989780 | Cd1d2 |
| Treg_1_peak_43526 | 7.70293 | 11.9322 | 8.5491 | 159 | Distal Intergenic | 86986551 | 86989780 | Cd1d2 |
| Treg_1_peak_43527 | 4.74975 | 6.43314 | 3.57669 | 171 | Distal Intergenic | 86986551 | 86989780 | Cd1d2 |
| Treg_1_peak_27595 | 5.94666 | 15.505 | 11.9005 | 200 | Distal Intergenic | 45392245 | 45400312 | Cd200 |
| Treg_1_peak_27596 | 6.89044 | 10.3071 | 7.07804 | 130 | Downstream (1-2kb) | 45392245 | 45400312 | Cd200 |
| Treg_1_peak_27597 | 8.76966 | 14.3242 | 10.771 | 188 | Intron (ENSMUST000 | 45392245 | 45400312 | Cd200 |
| Treg_1_peak_27598 | 7.70293 | 11.9322 | 8.5491 | 369 | Promoter (2-3kb) | 45392245 | 45400312 | Cd200 |
| Treg_1_peak_27592 | 4.62176 | 6.12093 | 3.35997 | 216 | Distal Intergenic | 44943678 | 44964682 | Cd200r3 |
| Treg_1_peak_27593 | 5.2781 | 7.39534 | 4.48065 | 208 | Distal Intergenic | 44965254 | 44966854 | Cd200r3 |
| Treg_1_peak_62606 | 7.12463 | 10.8796 | 7.56521 | 129 | Downstream (1-2kb) | 3743801 | 3748927 | Cd209a |
| Treg_1_peak_62613 | 3.08117 | 3.58522 | 1.34861 | 81 | Distal Intergenic | 3918677 | 3926813 | Cd209b |
| Treg_1_peak_62614 | 3.08117 | 3.58522 | 1.34861 | 226 | Distal Intergenic | 3918677 | 3926813 | Cd209b |
| Treg_1_peak_62615 | 6.333 | 9.34439 | 6.16031 | 137 | Distal Intergenic | 3917655 | 3926844 | Cd209b |
| Treg_1_peak_62610 | 8.70788 | 14.0823 | 10.5478 | 162 | Distal Intergenic | 3871824 | 3878555 | Cd209d |
| Treg_1_peak_62611 | 4.74975 | 6.43314 | 3.57669 | 214 | Distal Intergenic | 3871824 | 3878555 | Cd209d |
| Treg_1_peak_62612 | 5.54138 | 7.86 | 4.82256 | 179 | Distal Intergenic | 3871824 | 3878555 | Cd209d |
| Treg_1_peak_62608 | 4.62176 | 6.12093 | 3.35997 | 119 | Distal Intergenic | 3847965 | 3854309 | Cd209e |
| Treg_1_peak_62609 | 7.91625 | 12.4603 | 9.03111 | 167 | Distal Intergenic | 3847965 | 3854309 | Cd209e |
| Treg_1_peak_62607 | 5.54138 | 7.86 | 4.82256 | 110 | Intron (ENSMUST000 | 3847965 | 3854309 | Cd209e |
| Treg_1_peak_62621 | 6.06246 | 8.68979 | 5.61559 | 134 | Distal Intergenic | 4134652 | 4137707 | Cd209g |
| Treg_1_peak_34310 | 3.95813 | 5.07252 | 2.42876 | 219 | Intron (ENSMUST000 | 89206101 | 89207338 | Cd226 |
| Treg_1_peak_5099 | 7.51685 | 11.6125 | 8.26253 | 159 | Intron (ENSMUST000 | 171579063 | 171585318 | Cd244a |
| Treg_1_peak_4894 | 8.37406 | 13.2159 | 9.74736 | 127 | Intron (ENSMUST000 | 165788765 | 165870249 | Cd247 |
| Treg_1_peak_4893 | 5.01123 | 6.63059 | 3.75453 | 114 | Promoter (<=1kb) | 165788681 | 165871153 | Cd247 |
| Treg_1_peak_7085 | 5.37988 | 7.55887 | 4.60357 | 387 | Distal Intergenic | 43578284 | 43579197 | Cd24a |
| Treg_1_peak_7086 | 4.62176 | 6.12093 | 3.35997 | 260 | Distal Intergenic | 43578284 | 43579197 | Cd24a |
| Treg_1_peak_68006 | 6.20798 | 9.03363 | 5.90404 | 192 | Distal Intergenic | 58535576 | 58537471 | Cd276 |
| Treg_1_peak_30215 | 7.12463 | 10.8796 | 7.56521 | 150 | Distal Intergenic | 42792952 | 42799888 | Cd2ap |
| Treg_1_peak_30218 | 13.8653 | 25.3877 | 21.3241 | 171 | Distal Intergenic | 42816264 | 42876665 | Cd2ap |
| Treg_1_peak_30219 | 6.61086 | 9.82156 | 6.61457 | 119 | Distal Intergenic | 42816264 | 42876665 | Cd2ap |
| Treg_1_peak_30220 | 7.70293 | 11.9322 | 8.5491 | 157 | Distal Intergenic | 42816264 | 42876665 | Cd2ap |
| Treg_1_peak_30216 | 11.5544 | 20.1201 | 16.2607 | 181 | Intron (ENSMUST000 | 42825421 | 42831030 | Cd2ap |
| Treg_1_peak_30217 | 4.62176 | 6.12093 | 3.35997 | 113 | Intron (ENSMUST000 | 42792951 | 42876389 | Cd2ap |
| Treg_1_peak_13085 | 7.8745 | 12.1733 | 8.77774 | 154 | Distal Intergenic | 114890041 | 114904654 | Cd300a |
| Treg_1_peak_13086 | 9.24352 | 15.1056 | 11.5129 | 146 | Distal Intergenic | 114890041 | 114904654 | Cd300a |

| | | | | | | | | |
|---|---|---|---|---|---|---|---|---|
| Treg_1_peak_13089 | 4.62176 | 6.12093 | 3.35997 | 124 | Downstream (<1kb) | 114982274 | 114989922 | Cd300ld |
| Treg_1_peak_13088 | 4.40907 | 5.64739 | 2.94543 | 139 | Downstream (1-2kb) | 114982274 | 114989922 | Cd300ld |
| Treg_1_peak_13090 | 6.26404 | 9.03921 | 5.90404 | 414 | Promoter (<=1kb) | 114982274 | 114989922 | Cd300ld |
| Treg_1_peak_13097 | 3.75842 | 4.42484 | 1.96438 | 117 | Intron (ENSMUST000 | 115120357 | 115126791 | Cd300lf |
| Treg_1_peak_13098 | 5.40173 | 7.51681 | 4.56243 | 192 | Intron (ENSMUST000 | 115120357 | 115126791 | Cd300lf |
| Treg_1_peak_13099 | 5.63764 | 7.8123 | 4.82256 | 154 | Intron (ENSMUST000 | 115120357 | 115126791 | Cd300lf |
| Treg_1_peak_13096 | 3.85147 | 4.81413 | 2.28385 | 94 | Intron (ENSMUST000 | 115120357 | 115126791 | Cd300lf |
| Treg_1_peak_13102 | 8.68183 | 14.0155 | 10.5057 | 165 | Promoter (<=1kb) | 115118848 | 115133991 | Cd300lf |
| Treg_1_peak_13103 | 11.64 | 20.3281 | 16.4631 | 139 | Promoter (<=1kb) | 115116214 | 115133992 | Cd300lf |
| Treg_1_peak_13101 | 4.65066 | 5.96969 | 3.25562 | 117 | Promoter (2-3kb) | 115120261 | 115126850 | Cd300lf |
| Treg_1_peak_13100 | 13.9621 | 25.6451 | 21.5714 | 204 | Promoter (2-3kb) | 115120357 | 115126791 | Cd300lf |
| Treg_1_peak_37478 | 3.95813 | 5.07252 | 2.42876 | 229 | Intron (ENSMUST000 | 60251993 | 60284488 | Cd302 |
| Treg_1_peak_59606 | 8.14325 | 12.9523 | 9.49681 | 195 | Promoter (<=1kb) | 43528526 | 43532678 | Cd33 |
| Treg_1_peak_59607 | 3.75842 | 4.42484 | 1.96438 | 151 | Promoter (<=1kb) | 43524216 | 43533171 | Cd33 |
| Treg_1_peak_5865 | 5.54138 | 7.86 | 4.82256 | 124 | Distal Intergenic | 194938819 | 194961279 | Cd34 |
| Treg_1_peak_50170 | 5.39205 | 7.49389 | 4.54135 | 115 | Distal Intergenic | 17814675 | 17888801 | Cd36 |
| Treg_1_peak_50169 | 4.74975 | 6.43314 | 3.57669 | 129 | Intron (ENSMUST000 | 17781690 | 17849792 | Cd36 |
| Treg_1_peak_50168 | 5.54138 | 7.86 | 4.82256 | 150 | Intron (ENSMUST000 | 17782016 | 17835696 | Cd36 |
| Treg_1_peak_50875 | 8.14325 | 12.9523 | 9.49681 | 137 | Promoter (2-3kb) | 43868553 | 43912375 | Cd38 |
| Treg_1_peak_67545 | 4.74975 | 6.43314 | 3.57669 | 116 | Promoter (<=1kb) | 44981786 | 44987339 | Cd3d |
| Treg_1_peak_57756 | 6.61916 | 41.3825 | 36.8683 | 223 | Promoter (2-3kb) | 124867333 | 124888199 | Cd4 |
| Treg_1_peak_40489 | 5.86393 | 8.86216 | 5.78445 | 191 | Intron (ENSMUST000 | 165062676 | 165066695 | Cd40 |
| Treg_1_peak_40487 | 7.50479 | 11.5911 | 8.25233 | 144 | Promoter (1-2kb) | 165062676 | 165066695 | Cd40 |
| Treg_1_peak_40488 | 4.12763 | 5.1875 | 2.54049 | 117 | Promoter (2-3kb) | 165062676 | 165066695 | Cd40 |
| Treg_1_peak_38656 | 7.34339 | 11.1301 | 7.8015 | 119 | Distal Intergenic | 102811141 | 102901665 | Cd44 |
| Treg_1_peak_38657 | 8.81814 | 14.1372 | 10.596 | 139 | Distal Intergenic | 102811141 | 102901665 | Cd44 |
| Treg_1_peak_38658 | 6.16235 | 8.9243 | 5.81442 | 141 | Distal Intergenic | 102811141 | 102901665 | Cd44 |
| Treg_1_peak_38659 | 3.85147 | 4.81413 | 2.28385 | 90 | Distal Intergenic | 102811141 | 102901665 | Cd44 |
| Treg_1_peak_38655 | 7.22591 | 13.2958 | 9.8262 | 250 | Promoter (1-2kb) | 102811141 | 102901665 | Cd44 |
| Treg_1_peak_5867 | 4.31749 | 5.45752 | 2.78881 | 119 | Intron (ENSMUST000 | 195041835 | 195069114 | Cd46 |
| Treg_1_peak_5868 | 5.54138 | 7.86 | 4.82256 | 150 | Intron (ENSMUST000 | 195041835 | 195069114 | Cd46 |
| Treg_1_peak_5870 | 4.74975 | 6.43314 | 3.57669 | 184 | Promoter (<=1kb) | 195041835 | 195069114 | Cd46 |
| Treg_1_peak_5869 | 4.74975 | 6.43314 | 3.57669 | 130 | Promoter (2-3kb) | 195041835 | 195069114 | Cd46 |
| Treg_1_peak_27717 | 10.6489 | 18.6112 | 14.8258 | 213 | Distal Intergenic | 49866833 | 49911091 | Cd47 |
| Treg_1_peak_27718 | 5.54138 | 7.86 | 4.82256 | 126 | Distal Intergenic | 49866833 | 49911091 | Cd47 |
| Treg_1_peak_27716 | 11.9017 | 21.5956 | 17.6791 | 150 | Intron (ENSMUST000 | 49866833 | 49911091 | Cd47 |
| Treg_1_peak_27715 | 5.39205 | 7.49389 | 4.54135 | 149 | Intron (ENSMUST000 | 49800533 | 49911046 | Cd47 |
| Treg_1_peak_44059 | 3.95813 | 5.07252 | 2.42876 | 93 | Distal Intergenic | 106759921 | 106790149 | Cd53 |
| Treg_1_peak_3941 | 6.33372 | 9.6184 | 6.42472 | 117 | Distal Intergenic | 130419601 | 130422740 | Cd55b |
| Treg_1_peak_3942 | 5.63764 | 7.8123 | 4.82256 | 111 | Distal Intergenic | 130419601 | 130422740 | Cd55b |
| Treg_1_peak_3943 | 3.85147 | 4.81413 | 2.28385 | 87 | Distal Intergenic | 130419601 | 130422740 | Cd55b |
| Treg_1_peak_3944 | 3.85147 | 4.81413 | 2.28385 | 106 | Intron (ENSMUST000 | 130419601 | 130422740 | Cd55b |
| Treg_1_peak_3945 | 6.98398 | 10.5289 | 7.25751 | 142 | Intron (ENSMUST000 | 130419601 | 130422740 | Cd55b |
| Treg_1_peak_43540 | 4.18703 | 5.19897 | 2.54561 | 172 | Intron (ENSMUST000 | 87357881 | 87371073 | Cd5l |
| Treg_1_peak_34494 | 5.14392 | 6.93808 | 4.0439 | 164 | Promoter (<=1kb) | 10789341 | 10830058 | Cd6 |
| Treg_1_peak_11669 | 9.73065 | 17.8784 | 14.135 | 180 | Promoter (<=1kb) | 69664306 | 69666062 | Cd68 |
| Treg_1_peak_57891 | 6.2783 | 9.20629 | 6.06554 | 159 | Distal Intergenic | 129267325 | 129275436 | Cd69 |
| Treg_1_peak_30604 | 3.1665 | 3.78996 | 1.3917 | 106 | Distal Intergenic | 57145997 | 57149777 | Cd70 |
| Treg_1_peak_33422 | 3.75842 | 4.42484 | 1.96438 | 399 | Promoter (2-3kb) | 60803848 | 60809253 | Cd74 |
| Treg_1_peak_12751 | 5.09926 | 6.90493 | 4.02125 | 231 | Downstream (<1kb) | 106311344 | 106314529 | Cd79b |
| Treg_1_peak_12750 | 3.19222 | 3.61568 | 1.37597 | 96 | Downstream (1-2kb) | 106311344 | 106314529 | Cd79b |
| Treg_1_peak_27440 | 5.54138 | 7.86 | 4.82256 | 136 | Intron (ENSMUST000 | 38459118 | 38486447 | Cd80 |
| Treg_1_peak_62534 | 10.864 | 18.6118 | 14.8258 | 186 | Intron (ENSMUST000 | 143052739 | 143067934 | Cd81 |
| Treg_1_peak_62533 | 6.26404 | 9.03921 | 5.90404 | 129 | Intron (ENSMUST000 | 143052739 | 143067934 | Cd81 |
| Treg_1_peak_17776 | 3.08117 | 3.58522 | 1.34861 | 259 | Distal Intergenic | 43784775 | 43803132 | Cd83 |
| Treg_1_peak_5110 | 6.24294 | 9.11882 | 5.9812 | 150 | Distal Intergenic | 171839697 | 171890718 | Cd84 |
| Treg_1_peak_5112 | 5.39205 | 7.49389 | 4.54135 | 124 | Intron (ENSMUST000 | 171840633 | 171886430 | Cd84 |
| Treg_1_peak_27386 | 14.6969 | 27.3892 | 23.262 | 205 | Intron (ENSMUST000 | 36603869 | 36666077 | Cd86 |
| Treg_1_peak_27384 | 5.01123 | 6.63059 | 3.75453 | 127 | Intron (ENSMUST000 | 36620713 | 36642805 | Cd86 |
| Treg_1_peak_27387 | 7.12463 | 10.8796 | 7.56521 | 166 | Promoter (1-2kb) | 36603869 | 36666077 | Cd86 |
| Treg_1_peak_27385 | 3.0527 | 3.52025 | 1.34861 | 71 | Promoter (2-3kb) | 36620713 | 36642805 | Cd86 |
| Treg_1_peak_56106 | 6.26404 | 9.03921 | 5.90404 | 136 | Exon (ENSMUST0000 | 71322788 | 71337494 | Cd8b1 |
| Treg_1_peak_57767 | 6.2783 | 9.20629 | 6.06554 | 173 | Distal Intergenic | 125462116 | 125463024 | Cd9 |
| Treg_1_peak_57768 | 5.87876 | 8.28611 | 5.22734 | 124 | Distal Intergenic | 125462116 | 125463024 | Cd9 |
| Treg_1_peak_57769 | 4.57905 | 6.02171 | 3.30528 | 104 | Distal Intergenic | 125462116 | 125463024 | Cd9 |
| Treg_1_peak_57770 | 5.45622 | 7.49968 | 4.54589 | 153 | Intron (ENSMUST000 | 125460589 | 125478302 | Cd9 |
| Treg_1_peak_40011 | 6.77133 | 10.3366 | 7.10727 | 146 | Distal Intergenic | 148436640 | 148443563 | Cd93 |
| Treg_1_peak_35090 | 4.74975 | 6.43314 | 3.57669 | 128 | Distal Intergenic | 34300075 | 34316677 | Fas |
| Treg_1_peak_35089 | 6.333 | 9.34439 | 6.16031 | 163 | Intron (ENSMUST000 | 34300075 | 34316677 | Fas |
| Treg_1_peak_4756 | 4.62176 | 6.12093 | 3.35997 | 138 | Promoter (2-3kb) | 161780689 | 161788495 | Fasl |
| Treg_1_peak_3968 | 3.95813 | 5.07252 | 2.42876 | 125 | Distal Intergenic | 131019845 | 131024974 | Il10 |
| Treg_1_peak_67550 | 3.95813 | 5.07252 | 2.42876 | 123 | Distal Intergenic | 45253840 | 45269149 | Il10ra |
| Treg_1_peak_46743 | 7.62573 | 12.7279 | 9.28642 | 128 | Intron (ENSMUST000 | 41699989 | 41769474 | Il11ra1 |
| Treg_1_peak_43044 | 5.39205 | 7.49389 | 4.54135 | 140 | Distal Intergenic | 68690644 | 68698547 | Il12a |
| Treg_1_peak_43045 | 5.14392 | 6.93808 | 4.0439 | 124 | Distal Intergenic | 68690644 | 68698547 | Il12a |
| Treg_1_peak_43047 | 6.66871 | 9.92084 | 6.71096 | 279 | Distal Intergenic | 68694021 | 68698547 | Il12a |
| Treg_1_peak_43048 | 4.62176 | 6.12093 | 3.35997 | 128 | Distal Intergenic | 68694021 | 68698547 | Il12a |
| Treg_1_peak_43046 | 9.81319 | 16.275 | 12.6162 | 154 | Promoter (2-3kb) | 68690644 | 68698547 | Il12a |
| Treg_1_peak_56008 | 6.333 | 9.34439 | 6.16031 | 211 | Intron (ENSMUST000 | 67292018 | 67376188 | Il12rb2 |
| Treg_1_peak_56009 | 9.50058 | 17.0445 | 13.35 | 152 | Intron (ENSMUST000 | 67292018 | 67376188 | Il12rb2 |
| Treg_1_peak_56005 | 5.54138 | 7.86 | 4.82256 | 143 | Intron (ENSMUST000 | 67291318 | 67339694 | Il12rb2 |
| Treg_1_peak_56006 | 4.38483 | 5.49927 | 2.81361 | 231 | Intron (ENSMUST000 | 67291318 | 67339694 | Il12rb2 |
| Treg_1_peak_56007 | 5.54138 | 7.86 | 4.82256 | 128 | Promoter (2-3kb) | 67291318 | 67339694 | Il12rb2 |
| Treg_1_peak_11123 | 5.88864 | 11.0129 | 7.69541 | 156 | Downstream (1-2kb) | 53631324 | 53634702 | Il13 |
| Treg_1_peak_70429 | 5.39205 | 7.49389 | 4.54135 | 191 | Intron (ENSMUST000 | 36112110 | 36171259 | Il13ra1 |
| Treg_1_peak_71230 | 4.74975 | 6.43314 | 3.57669 | 114 | Distal Intergenic | 147383478 | 147403832 | Il13ra2 |
| Treg_1_peak_71231 | 8.70788 | 14.0823 | 10.5478 | 156 | Distal Intergenic | 147383478 | 147403832 | Il13ra2 |
| Treg_1_peak_64852 | 11.0828 | 19.1625 | 15.347 | 167 | Distal Intergenic | 82331914 | 82344473 | Il15 |

| | | | | | | | | |
|---|---|---|---|---|---|---|---|---|
| Treg_1_peak_64853 | 5.01123 | 6.63059 | 3.75453 | 152 | Promoter (<=1kb) | 82345662 | 82398627 | Il15 |
| Treg_1_peak_36218 | 7.75997 | 12.0697 | 8.67583 | 143 | Distal Intergenic | 11705290 | 11734317 | Il15ra |
| Treg_1_peak_36219 | 11.084 | 21.0945 | 17.1941 | 235 | Promoter (1-2kb) | 11723164 | 11733968 | Il15ra |
| Treg_1_peak_60754 | 4.73554 | 6.39728 | 3.57669 | 135 | Promoter (1-2kb) | 83722510 | 83739937 | Il16 |
| Treg_1_peak_497 | 3.1665 | 3.78996 | 1.3917 | 160 | Distal Intergenic | 20730905 | 20734496 | Il17a |
| Treg_1_peak_498 | 12.6674 | 25.3261 | 21.2737 | 177 | Distal Intergenic | 20730905 | 20734496 | Il17a |
| Treg_1_peak_499 | 5.54138 | 7.86 | 4.82256 | 150 | Promoter (1-2kb) | 20777679 | 20779554 | Il17f |
| Treg_1_peak_57601 | 7.70293 | 11.9322 | 8.5491 | 188 | Distal Intergenic | 120463247 | 120483729 | Il17ra |
| Treg_1_peak_20744 | 4.74975 | 6.43314 | 3.57669 | 111 | Distal Intergenic | 27038941 | 27107286 | Il17rd |
| Treg_1_peak_20745 | 6.93264 | 10.4055 | 7.14268 | 157 | Intron (ENSMUST000 | 27039256 | 27102509 | Il17rd |
| Treg_1_peak_20746 | 4.74975 | 6.43314 | 3.57669 | 114 | Intron (ENSMUST000 | 27039256 | 27102509 | Il17rd |
| Treg_1_peak_20747 | 7.12463 | 10.8796 | 7.56521 | 144 | Intron (ENSMUST000 | 27067397 | 27100976 | Il17rd |
| Treg_1_peak_20748 | 6.61361 | 9.68484 | 6.48229 | 154 | Intron (ENSMUST000 | 27087765 | 27101491 | Il17rd |
| Treg_1_peak_57386 | 4.75029 | 6.34321 | 3.56123 | 115 | Distal Intergenic | 113458484 | 113470758 | Il17re |
| Treg_1_peak_57387 | 4.47476 | 6.91967 | 4.03572 | 124 | Promoter (<=1kb) | 113466360 | 113469987 | Il17re |
| Treg_1_peak_67764 | 10.0225 | 17.1554 | 13.4511 | 152 | Distal Intergenic | 50575273 | 50581837 | Il18 |
| Treg_1_peak_3964 | 5.39205 | 7.49389 | 4.54135 | 123 | Distal Intergenic | 130932816 | 130940115 | Il19 |
| Treg_1_peak_3965 | 8.14325 | 12.9523 | 9.49681 | 168 | Distal Intergenic | 130932816 | 130940115 | Il19 |
| Treg_1_peak_3966 | 4.62176 | 6.12093 | 3.35997 | 231 | Distal Intergenic | 130932816 | 130940115 | Il19 |
| Treg_1_peak_3967 | 6.94508 | 10.4352 | 7.1716 | 280 | Distal Intergenic | 130932816 | 130940115 | Il19 |
| Treg_1_peak_36582 | 5.80591 | 8.48822 | 5.41921 | 139 | Distal Intergenic | 24291196 | 24293820 | Il1f10 |
| Treg_1_peak_36581 | 4.62176 | 6.12093 | 3.35997 | 118 | Distal Intergenic | 24276954 | 24282432 | Il1f5 |
| Treg_1_peak_36580 | 4.62176 | 6.12093 | 3.35997 | 134 | Promoter (2-3kb) | 24215505 | 24225525 | Il1f6 |
| Treg_1_peak_36578 | 4.74975 | 6.43314 | 3.57669 | 123 | Distal Intergenic | 24153161 | 24160519 | Il1f8 |
| Treg_1_peak_36579 | 4.74975 | 6.43314 | 3.57669 | 140 | Promoter (<=1kb) | 24153161 | 24160519 | Il1f8 |
| Treg_1_peak_1093 | 5.54138 | 7.86 | 4.82256 | 177 | Distal Intergenic | 40225080 | 40317257 | Il1r1 |
| Treg_1_peak_1094 | 4.41584 | 6.28336 | 3.50757 | 139 | Distal Intergenic | 40225080 | 40317257 | Il1r1 |
| Treg_1_peak_1095 | 7.12463 | 10.8796 | 7.56521 | 166 | Distal Intergenic | 40225080 | 40317257 | Il1r1 |
| Treg_1_peak_1096 | 3.1665 | 3.78996 | 1.3917 | 115 | Distal Intergenic | 40225080 | 40317257 | Il1r1 |
| Treg_1_peak_1097 | 5.39205 | 7.49389 | 4.54135 | 237 | Intron (ENSMUST000 | 40225080 | 40317257 | Il1r1 |
| Treg_1_peak_1099 | 6.16235 | 8.9243 | 5.81442 | 146 | Intron (ENSMUST000 | 40266657 | 40277178 | Il1r1 |
| Treg_1_peak_1102 | 7.84787 | 12.2867 | 8.88865 | 168 | Intron (ENSMUST000 | 40266657 | 40277178 | Il1r1 |
| Treg_1_peak_1100 | 4.75029 | 6.34321 | 3.56123 | 157 | Intron (ENSMUST000 | 40266657 | 40277178 | Il1r1 |
| Treg_1_peak_1101 | 5.5248 | 7.81796 | 4.82256 | 95 | Intron (ENSMUST000 | 40266657 | 40277178 | Il1r1 |
| Treg_1_peak_1087a | 5.65411 | 7.8411 | 4.82256 | 120 | Distal Intergenic | 40074079 | 40112136 | Il1r2 |
| Treg_1_peak_1087b | 5.36237 | 7.34092 | 4.43196 | 520 | Distal Intergenic | 40074079 | 40112136 | Il1r2 |
| Treg_1_peak_1088 | 3.85147 | 4.81413 | 2.28385 | 65 | Promoter (2-3kb) | 40084698 | 40125231 | Il1r2 |
| Treg_1_peak_27079 | 5.91033 | 10.6722 | 7.39786 | 143 | Distal Intergenic | 26581704 | 26725264 | Il1rap |
| Treg_1_peak_27080 | 5.84775 | 8.22008 | 5.17329 | 134 | Distal Intergenic | 26581704 | 26725264 | Il1rap |
| Treg_1_peak_27081 | 4.62176 | 6.12093 | 3.35997 | 148 | Distal Intergenic | 26581704 | 26725264 | Il1rap |
| Treg_1_peak_27082 | 5.14392 | 6.93808 | 4.0439 | 109 | Intron (ENSMUST000 | 26727042 | 26728228 | Il1rap |
| Treg_1_peak_70793 | 7.51685 | 11.6125 | 8.26253 | 196 | Intron (ENSMUST000 | 86747242 | 87890235 | Il1rapl1 |
| Treg_1_peak_70794 | 11.8744 | 20.9188 | 17.0233 | 217 | Intron (ENSMUST000 | 86747242 | 87890235 | Il1rapl1 |
| Treg_1_peak_71153 | 4.62176 | 6.12093 | 3.35997 | 117 | Distal Intergenic | 137570608 | 138846946 | Il1rapl2 |
| Treg_1_peak_71154 | 4.74975 | 6.43314 | 3.57669 | 119 | Intron (ENSMUST000 | 137571019 | 138506311 | Il1rapl2 |
| Treg_1_peak_1107 | 3.95813 | 5.07252 | 2.42876 | 126 | Intron (ENSMUST000 | 40440628 | 40465396 | Il1rl1 |
| Treg_1_peak_1103 | 3.19222 | 3.61568 | 1.37597 | 117 | Intron (ENSMUST000 | 40324610 | 40329441 | Il1rl2 |
| Treg_1_peak_42167 | 5.63764 | 7.8123 | 4.82256 | 158 | Distal Intergenic | 37120523 | 37125959 | Il2 |
| Treg_1_peak_42168 | 10.4542 | 17.6518 | 13.9137 | 131 | Distal Intergenic | 37120523 | 37125959 | Il2 |
| Treg_1_peak_42166 | 14.2493 | 26.3487 | 22.2482 | 309 | Promoter (<=1kb) | 37120523 | 37125959 | Il2 |
| Treg_1_peak_3963 | 5.49351 | 7.73962 | 4.76883 | 164 | Distal Intergenic | 130906985 | 130911451 | Il20 |
| Treg_1_peak_3962 | 5.70585 | 7.932 | 4.89178 | 113 | Promoter (1-2kb) | 130906985 | 130911451 | Il20 |
| Treg_1_peak_6391 | 9.39606 | 15.7258 | 12.0953 | 249 | Distal Intergenic | 19712570 | 19760053 | Il20ra |
| Treg_1_peak_69235 | 6.333 | 9.34439 | 6.16031 | 147 | Intron (ENSMUST000 | 100465708 | 100486385 | Il20rb |
| Treg_1_peak_69236 | 7.91625 | 12.4603 | 9.03111 | 395 | Promoter (2-3kb) | 100465708 | 100486385 | Il20rb |
| Treg_1_peak_9343 | 4.62176 | 6.12093 | 3.35997 | 108 | Distal Intergenic | 118204942 | 118210047 | Il22 |
| Treg_1_peak_9344 | 6.6438 | 9.72451 | 6.51922 | 125 | Distal Intergenic | 118204942 | 118210047 | Il22 |
| Treg_1_peak_49290 | 10.6446 | 26.5882 | 22.4837 | 227 | Distal Intergenic | 135728172 | 135752140 | Il22ra1 |
| Treg_1_peak_49291 | 11.1654 | 19.3137 | 15.4938 | 126 | Intron (ENSMUST000 | 135728172 | 135752140 | Il22ra1 |
| Treg_1_peak_6387 | 10.5562 | 19.723 | 15.8944 | 220 | Distal Intergenic | 19621998 | 19634681 | Il22ra2 |
| Treg_1_peak_56012 | 6.89044 | 10.3071 | 7.07804 | 153 | Intron (ENSMUST000 | 67422932 | 67491855 | Il23r |
| Treg_1_peak_56013 | 9.24352 | 15.1056 | 11.5129 | 164 | Intron (ENSMUST000 | 67422932 | 67491855 | Il23r |
| Treg_1_peak_62061 | 5.54138 | 7.86 | 4.82256 | 145 | Distal Intergenic | 126589010 | 126594941 | Il27 |
| Treg_1_peak_36217 | 4.75029 | 6.34321 | 3.56123 | 129 | Promoter (2-3kb) | 11642807 | 11693193 | Il2ra |
| Treg_1_peak_25544 | 6.38918 | 9.4461 | 6.25977 | 148 | Exon (ENSMUST0000 | 78481324 | 78493756 | Il2rb |
| Treg_1_peak_11144 | 6.16235 | 8.9243 | 5.81442 | 316 | Distal Intergenic | 54265303 | 54267277 | Il3 |
| Treg_1_peak_11143 | 4.74975 | 6.43314 | 3.57669 | 163 | Promoter (<=1kb) | 54265303 | 54267277 | Il3 |
| Treg_1_peak_53228 | 5.39205 | 7.49389 | 4.54135 | 134 | Downstream (2-3kb) | 123480157 | 123482101 | Il31 |
| Treg_1_peak_53230 | 7.82953 | 14.2949 | 10.7518 | 149 | Intron (ENSMUST000 | 123480401 | 123489489 | Il31 |
| Treg_1_peak_19915 | 3.06961 | 3.55865 | 1.34861 | 283 | Intron (ENSMUST000 | 112525846 | 112532344 | Il31ra |
| Treg_1_peak_19914 | 11.4008 | 23.7739 | 19.7727 | 446 | Intron (ENSMUST000 | 112525846 | 112532344 | Il31ra |
| Treg_1_peak_34975 | 4.62176 | 6.12093 | 3.35997 | 210 | Distal Intergenic | 29925114 | 29960718 | Il33 |
| Treg_1_peak_11122 | 11.5623 | 25.8014 | 21.7264 | 234 | Distal Intergenic | 53612460 | 53618669 | Il4 |
| Treg_1_peak_11120 | 6.26404 | 9.03921 | 5.90404 | 133 | Intron (ENSMUST000 | 53602982 | 53617224 | Il4 |
| Treg_1_peak_11121 | 6.89044 | 10.3071 | 7.07804 | 161 | Promoter (<=1kb) | 53602982 | 53617224 | Il4 |
| Treg_1_peak_57224 | 3.75842 | 4.42484 | 1.96438 | 252 | Promoter (<=1kb) | 106710357 | 106749037 | Il5ra |
| Treg_1_peak_50523 | 4.74975 | 6.43314 | 3.57669 | 107 | Distal Intergenic | 30013321 | 30019839 | Il6 |
| Treg_1_peak_41238 | 8.47322 | 13.5001 | 10.0067 | 230 | Distal Intergenic | 7573182 | 7613760 | Il7 |
| Treg_1_peak_41239 | 4.74975 | 6.43314 | 3.57669 | 110 | Distal Intergenic | 7573182 | 7613760 | Il7 |
| Treg_1_peak_41235 | 3.1665 | 3.78996 | 1.3917 | 104 | Intron (ENSMUST000 | 7573182 | 7613760 | Il7 |
| Treg_1_peak_41236 | 5.39205 | 7.49389 | 4.54135 | 180 | Intron (ENSMUST000 | 7573182 | 7613760 | Il7 |
| Treg_1_peak_41237 | 4.65598 | 6.20215 | 3.43047 | 147 | Intron (ENSMUST000 | 7573182 | 7613760 | Il7 |
| Treg_1_peak_41234 | 3.85147 | 4.81413 | 2.28385 | 117 | Intron (ENSMUST000 | 7572028 | 7605864 | Il7 |
| Treg_1_peak_23685 | 5.01123 | 6.63059 | 3.75453 | 191 | Intron (ENSMUST000 | 9516409 | 9530176 | Il7r |
| Treg_1_peak_23684 | 6.16235 | 8.9243 | 5.81442 | 124 | Intron (ENSMUST000 | 9511898 | 9529766 | Il7r |
| Treg_1_peak_26640 | 4.56032 | 6.26269 | 3.48849 | 153 | Distal Intergenic | 10782240 | 10785536 | Socs1 |
| Treg_1_peak_8671 | 6.16235 | 8.9243 | 5.81442 | 200 | Distal Intergenic | 95387667 | 95392967 | Socs2 |
| Treg_1_peak_8674 | 5.24951 | 7.15236 | 4.25278 | 127 | Exon (ENSMUST0000 | 95387667 | 95392967 | Socs2 |

| | | | | | | | | |
|---|---|---|---|---|---|---|---|---|
| Treg_1_peak_8673 | 10.0344 | 17.9375 | 14.1933 | 151 | Intron (ENSMUST000 | 95387667 | 95392967 | Socs2 |
| Treg_1_peak_8672 | 5.80591 | 8.48822 | 5.41921 | 142 | Promoter (<=1kb) | 95387667 | 95392967 | Socs2 |
| Treg_1_peak_31551 | 7.75255 | 13.7376 | 10.2352 | 179 | Distal Intergenic | 87107679 | 87137839 | Socs5 |
| Treg_1_peak_31552 | 6.33372 | 9.6184 | 6.42472 | 130 | Distal Intergenic | 87107858 | 87122990 | Socs5 |
| Treg_1_peak_34304 | 10.088 | 16.9255 | 13.2351 | 136 | Intron (ENSMUST000 | 88665224 | 88758491 | Socs6 |
| Treg_1_peak_34303 | 4.74975 | 6.43314 | 3.57669 | 110 | Promoter (<=1kb) | 88665224 | 88758491 | Socs6 |
| Treg_1_peak_34302 | 4.62176 | 6.12093 | 3.35997 | 115 | Promoter (1-2kb) | 88665224 | 88758491 | Socs6 |
| Treg_1_peak_34308 | 3.95813 | 5.07252 | 2.42876 | 162 | Promoter (1-2kb) | 88870765 | 88927481 | Socs6 |

| Table S8: Narrow peaks ChIPseeker annotation of HDAC8 in Treg cells (2) | | | | | | | | |
|---|---|---|---|---|---|---|---|---|
| name | signalValue | '-log10pvalue | '-log10qvalue | peak_summit | annotation | geneStart | geneEnd | geneName |
| Treg_2_peak_24111 | 3.69374 | 5.73445 | 3.33556 | 99 | Distal Intergenic | 82176657 | 82179812 | Ccl1 |
| Treg_2_peak_24112 | 4.61717 | 7.516 | 4.89772 | 137 | Distal Intergenic | 82176657 | 82179812 | Ccl1 |
| Treg_2_peak_24113 | 3.6359 | 5.41249 | 3.16527 | 98 | Distal Intergenic | 82176657 | 82179812 | Ccl1 |
| Treg_2_peak_24114 | 3.69374 | 5.73445 | 3.33556 | 97 | Distal Intergenic | 82176657 | 82179812 | Ccl1 |
| Treg_2_peak_24123 | 4.54487 | 7.11231 | 4.62813 | 113 | Distal Intergenic | 82176657 | 82305690 | Ccl1 |
| Treg_2_peak_24124 | 3.6666 | 5.57696 | 3.31807 | 199 | Distal Intergenic | 82176657 | 82305690 | Ccl1 |
| Treg_2_peak_24125 | 4.61717 | 7.516 | 4.89772 | 115 | Distal Intergenic | 82176657 | 82305690 | Ccl1 |
| Treg_2_peak_24126 | 6.41655 | 11.0261 | 8.13533 | 104 | Distal Intergenic | 82176657 | 82305690 | Ccl1 |
| Treg_2_peak_24127 | 6.15184 | 8.89942 | 6.19613 | 130 | Distal Intergenic | 82176657 | 82305690 | Ccl1 |
| Treg_2_peak_24120 | 5.48847 | 9.07546 | 6.36882 | 121 | Intron (ENSMUST000001 | 82176657 | 82305690 | Ccl1 |
| Treg_2_peak_24119 | 3.69374 | 5.73445 | 3.33556 | 122 | Intron (ENSMUST000001 | 82176657 | 82305690 | Ccl1 |
| Treg_2_peak_24118 | 3.69374 | 5.73445 | 3.33556 | 114 | Intron (ENSMUST000001 | 82176657 | 82305690 | Ccl1 |
| Treg_2_peak_24116 | 12.7256 | 24.9724 | 21.2937 | 157 | Intron (ENSMUST000001 | 82176659 | 82179812 | Ccl1 |
| Treg_2_peak_24117 | 4.59282 | 7.37271 | 4.8659 | 110 | Intron (ENSMUST000001 | 82176659 | 82179812 | Ccl1 |
| Treg_2_peak_24115 | 4.54487 | 7.11231 | 4.62813 | 314 | Intron (ENSMUST000001 | 82176659 | 82179812 | Ccl1 |
| Treg_2_peak_24121 | 4.39989 | 6.45451 | 4.03544 | 201 | Promoter (<=1kb) | 82176657 | 82305690 | Ccl1 |
| Treg_2_peak_24122 | 8.9794 | 16.0485 | 12.7895 | 174 | Promoter (1-2kb) | 82176657 | 82305690 | Ccl1 |
| Treg_2_peak_24102 | 4.61717 | 7.516 | 4.89772 | 209 | Distal Intergenic | 82057823 | 82062955 | Ccl11 |
| Treg_2_peak_24103 | 5.5406 | 9.37639 | 6.57036 | 194 | Distal Intergenic | 82057823 | 82062955 | Ccl11 |
| Treg_2_peak_24104 | 3.6359 | 5.41249 | 3.16527 | 102 | Distal Intergenic | 82101845 | 82103400 | Ccl12 |
| Treg_2_peak_129840 | 7.49823 | 11.9232 | 8.93461 | 146 | Promoter (<=1kb) | 94810453 | 94812036 | Ccl17 |
| Treg_2_peak_24099 | 4.48055 | 6.80003 | 4.37459 | 237 | Intron (ENSMUST000001 | 82035571 | 82037453 | Ccl2 |
| Treg_2_peak_24100 | 3.59176 | 5.19719 | 3.00172 | 121 | Intron (ENSMUST000001 | 82035571 | 82037453 | Ccl2 |
| Treg_2_peak_4995 | 3.69374 | 5.73445 | 3.33556 | 120 | Distal Intergenic | 83116766 | 83119166 | Ccl20 |
| Treg_2_peak_4996 | 4.54487 | 7.11231 | 4.62813 | 205 | Distal Intergenic | 83116766 | 83119166 | Ccl20 |
| Treg_2_peak_129835 | 6.15984 | 9.81068 | 6.99648 | 121 | Distal Intergenic | 94745590 | 94751699 | Ccl22 |
| Treg_2_peak_129836 | 10.9996 | 19.4911 | 16.0704 | 143 | Distal Intergenic | 94745590 | 94751699 | Ccl22 |
| Treg_2_peak_129837 | 7.49823 | 11.9232 | 8.93461 | 120 | Distal Intergenic | 94745590 | 94751699 | Ccl22 |
| Treg_2_peak_106999 | 2.7703 | 4.04921 | 1.90871 | 242 | Distal Intergenic | 135570580 | 135573049 | Ccl24 |
| Treg_2_peak_106998 | 4.39989 | 6.45451 | 4.03544 | 202 | Promoter (2-3kb) | 135569937 | 135573049 | Ccl24 |
| Treg_2_peak_124388 | 7.27179 | 12.6392 | 9.60486 | 479 | Intron (ENSMUST000001 | 4349588 | 4360020 | Ccl25 |
| Treg_2_peak_124387 | 7.49823 | 11.9232 | 8.93461 | 195 | Promoter (1-2kb) | 4332259 | 4334807 | Ccl25 |
| Treg_2_peak_39684 | 5.78926 | 8.5388 | 5.90073 | 109 | Intron (ENSMUST000000 | 119623819 | 119654359 | Ccl28 |
| Treg_2_peak_39683 | 5.83196 | 8.66693 | 6.00946 | 139 | Promoter (<=1kb) | 119623819 | 119654359 | Ccl28 |
| Treg_2_peak_24175 | 5.5406 | 9.37639 | 6.57036 | 156 | Promoter (<=1kb) | 83647844 | 83649355 | Ccl3 |
| Treg_2_peak_24101 | 4.54487 | 7.11231 | 4.62813 | 129 | Promoter (<=1kb) | 82045712 | 82047525 | Ccl7 |
| Treg_2_peak_24105 | 3.6359 | 5.41249 | 3.16527 | 107 | Distal Intergenic | 82115185 | 82116799 | Ccl8 |
| Treg_2_peak_24106 | 3.69374 | 5.73445 | 3.33556 | 101 | Distal Intergenic | 82115185 | 82116799 | Ccl8 |
| Treg_2_peak_24107 | 3.69374 | 5.73445 | 3.33556 | 156 | Distal Intergenic | 82115185 | 82116799 | Ccl8 |
| Treg_2_peak_24108 | 4.99882 | 7.12189 | 4.62813 | 204 | Distal Intergenic | 82115185 | 82116799 | Ccl8 |
| Treg_2_peak_24109 | 5.45384 | 8.8909 | 6.19058 | 113 | Distal Intergenic | 82115185 | 82116799 | Ccl8 |
| Treg_2_peak_24110 | 7.27179 | 12.6392 | 9.60486 | 162 | Distal Intergenic | 82115185 | 82116799 | Ccl8 |
| Treg_2_peak_24168 | 4.54487 | 7.11231 | 4.62813 | 84 | Distal Intergenic | 83575318 | 83577142 | Ccl9 |
| Treg_2_peak_24169 | 4.4897 | 6.84221 | 4.40836 | 118 | Promoter (1-2kb) | 83572919 | 83578636 | Ccl9 |
| Treg_2_peak_138512 | 3.51991 | 4.88833 | 2.70632 | 12 | Distal Intergenic | 123962124 | 123968692 | Ccr1 |
| Treg_2_peak_138513 | 3.69374 | 5.73445 | 3.33556 | 84 | Distal Intergenic | 123962124 | 123968692 | Ccr1 |
| Treg_2_peak_138514 | 3.69374 | 5.73445 | 3.33556 | 180 | Distal Intergenic | 123962124 | 123968692 | Ccr1 |
| Treg_2_peak_138515 | 3.69374 | 5.73445 | 3.33556 | 130 | Distal Intergenic | 123962124 | 123968692 | Ccr1 |
| Treg_2_peak_138521 | 4.61717 | 7.516 | 4.89772 | 188 | Distal Intergenic | 124101950 | 124113557 | Ccr2 |
| Treg_2_peak_138522 | 5.5406 | 9.37639 | 6.57036 | 734 | Distal Intergenic | 124101950 | 124113557 | Ccr2 |
| Treg_2_peak_138523 | 4.99882 | 7.12189 | 4.62813 | 187 | Distal Intergenic | 124101950 | 124113557 | Ccr2 |
| Treg_2_peak_138524 | 3.6359 | 5.41249 | 3.16527 | 109 | Distal Intergenic | 124101950 | 124113557 | Ccr2 |
| Treg_2_peak_138525 | 3.69374 | 5.73445 | 3.33556 | 106 | Promoter (<=1kb) | 124105626 | 124109140 | Ccr2 |
| Treg_2_peak_138516 | 4.99882 | 7.12189 | 4.62813 | 113 | Distal Intergenic | 124021972 | 124031689 | Ccr3 |
| Treg_2_peak_138517 | 5.35089 | 7.32162 | 4.81655 | 107 | Distal Intergenic | 124021972 | 124031689 | Ccr3 |
| Treg_2_peak_138518 | 4.99882 | 7.12189 | 4.62813 | 125 | Distal Intergenic | 124021972 | 124031689 | Ccr3 |
| Treg_2_peak_138519 | 6.46404 | 11.3035 | 8.33659 | 134 | Distal Intergenic | 124021972 | 124031689 | Ccr3 |
| Treg_2_peak_138520 | 4.58325 | 7.31856 | 4.81351 | 85 | Distal Intergenic | 124021972 | 124031689 | Ccr3 |
| Treg_2_peak_138527 | 10.7753 | 20.0586 | 16.5916 | 167 | Distal Intergenic | 124128748 | 124147699 | Ccr5 |
| Treg_2_peak_138526 | 4.61717 | 7.516 | 4.89772 | 136 | Promoter (<=1kb) | 124126684 | 124130250 | Ccr5 |
| Treg_2_peak_58230 | 7.27179 | 12.6392 | 9.60486 | 174 | Promoter (<=1kb) | 8236043 | 8256108 | Ccr6 |
| Treg_2_peak_24904 | 7.49823 | 11.9232 | 8.93461 | 142 | Distal Intergenic | 99144196 | 99155077 | Ccr7 |
| Treg_2_peak_24905 | 3.59176 | 5.19719 | 3.00172 | 154 | Distal Intergenic | 99144196 | 99155077 | Ccr7 |
| Treg_2_peak_24903 | 4.37364 | 6.35102 | 3.93704 | 107 | Promoter (1-2kb) | 99144196 | 99155077 | Ccr7 |
| Treg_2_peak_87666 | 4.61717 | 7.516 | 4.89772 | 137 | Promoter (1-2kb) | 100993529 | 101029556 | Cd101 |
| Treg_2_peak_136039 | 7.38747 | 13.2885 | 10.182 | 490 | Distal Intergenic | 78615546 | 78716253 | Cd109 |
| Treg_2_peak_65103 | 3.6359 | 5.41249 | 3.16527 | 125 | Distal Intergenic | 36725871 | 36726738 | Cd14 |
| Treg_2_peak_87414 | 3.69374 | 5.73445 | 3.33556 | 123 | Intron (ENSMUST000001 | 96800155 | 96814521 | Cd160 |
| Treg_2_peak_87413 | 3.69374 | 5.73445 | 3.33556 | 102 | Promoter (<=1kb) | 96800155 | 96814521 | Cd160 |
| Treg_2_peak_87412 | 12.0046 | 23.8855 | 20.2454 | 155 | Promoter (2-3kb) | 96800155 | 96814521 | Cd160 |
| Treg_2_peak_115068 | 4.99882 | 7.12189 | 4.62813 | 195 | Exon (ENSMUST0000003 | 124304700 | 124330527 | Cd163 |
| Treg_2_peak_115067 | 4.61717 | 7.516 | 4.89772 | 114 | Promoter (2-3kb) | 124304700 | 124330527 | Cd163 |
| Treg_2_peak_124141 | 6.10727 | 8.9785 | 6.27358 | 128 | Intron (ENSMUST000002 | 140224943 | 140228400 | Cd163l1 |
| Treg_2_peak_99082 | 9.0839 | 17.2866 | 13.9723 | 152 | Distal Intergenic | 133221534 | 133224242 | Cd164l2 |
| Treg_2_peak_117618 | 5.27987 | 8.0993 | 5.46746 | 134 | Distal Intergenic | 24743983 | 24760311 | Cd177 |
| Treg_2_peak_38562 | 4.61717 | 7.516 | 4.89772 | 117 | Distal Intergenic | 102693558 | 102706955 | Cd180 |
| Treg_2_peak_38564 | 3.69374 | 5.73445 | 3.33556 | 30 | Intron (ENSMUST000001 | 102693611 | 102739504 | Cd180 |
| Treg_2_peak_38563 | 3.6359 | 5.41249 | 3.16527 | 116 | Promoter (<=1kb) | 102693558 | 102706955 | Cd180 |
| Treg_2_peak_123432 | 6.6651 | 10.2696 | 7.44017 | 113 | Promoter (<=1kb) | 126409362 | 126409796 | Cd19 |
| Treg_2_peak_86915 | 3.59176 | 5.19719 | 3.00172 | 110 | Distal Intergenic | 86986551 | 86989780 | Cd1d2 |
| Treg_2_peak_86916 | 3.69374 | 5.73445 | 3.33556 | 98 | Distal Intergenic | 86986551 | 86989780 | Cd1d2 |
| Treg_2_peak_86917 | 3.6666 | 5.57696 | 3.31807 | 111 | Distal Intergenic | 86986551 | 86989780 | Cd1d2 |
| Treg_2_peak_86918 | 6.36282 | 10.7362 | 7.85556 | 136 | Distal Intergenic | 86986551 | 86989780 | Cd1d2 |
| Treg_2_peak_87676 | 3.51991 | 4.88833 | 2.70632 | 275 | Distal Intergenic | 101275908 | 101280389 | Cd2 |
| Treg_2_peak_87677 | 4.28398 | 5.38986 | 3.16527 | 165 | Distal Intergenic | 101275908 | 101280389 | Cd2 |
| Treg_2_peak_87678 | 6.35351 | 9.39706 | 6.59071 | 122 | Promoter (1-2kb) | 101275908 | 101280389 | Cd2 |
| Treg_2_peak_54762 | 13.7718 | 25.2339 | 21.5516 | 202 | Distal Intergenic | 45392245 | 45400312 | Cd200 |

| | | | | | | | | |
|---|---|---|---|---|---|---|---|---|
| Treg_2_peak_54763 | 6.46404 | 11.3035 | 8.33659 | 136 | Distal Intergenic | 45392245 | 45400312 | Cd200 |
| Treg_2_peak_54764 | 5.38764 | 8.56594 | 5.92038 | 107 | Downstream (1-2kb) | 45392245 | 45400312 | Cd200 |
| Treg_2_peak_54765 | 21.6616 | 44.8466 | 40.604 | 209 | Intron (ENSMUST000001 | 45392245 | 45400312 | Cd200 |
| Treg_2_peak_54767 | 2.7703 | 4.04921 | 1.90871 | 110 | Promoter (<=1kb) | 45392245 | 45400312 | Cd200 |
| Treg_2_peak_54768 | 3.69374 | 5.73445 | 3.33556 | 106 | Promoter (<=1kb) | 45392245 | 45400312 | Cd200 |
| Treg_2_peak_54766 | 8.33137 | 13.6223 | 10.4966 | 150 | Promoter (2-3kb) | 45392245 | 45400312 | Cd200 |
| Treg_2_peak_112716 | 6.6651 | 10.2696 | 7.44017 | 129 | Exon (ENSMUST0000003 | 83671215 | 83677857 | Cd207 |
| Treg_2_peak_112717 | 4.54487 | 7.11231 | 4.62813 | 112 | Promoter (2-3kb) | 83671215 | 83677857 | Cd207 |
| Treg_2_peak_10131 | 7.49823 | 11.9232 | 8.93461 | 160 | Intron (ENSMUST000001 | 165788765 | 165870249 | Cd247 |
| Treg_2_peak_10132 | 3.69374 | 5.73445 | 3.33556 | 144 | Intron (ENSMUST000001 | 165788765 | 165870249 | Cd247 |
| Treg_2_peak_10133 | 4.61717 | 7.516 | 4.89772 | 116 | Intron (ENSMUST000001 | 165788765 | 165870249 | Cd247 |
| Treg_2_peak_10138 | 8.49082 | 13.5423 | 10.4274 | 204 | Promoter (<=1kb) | 165858216 | 165861555 | Cd247 |
| Treg_2_peak_14385 | 7.78725 | 12.1363 | 9.14491 | 140 | Distal Intergenic | 43578284 | 43579197 | Cd24a |
| Treg_2_peak_134984 | 3.65138 | 5.49377 | 3.2443 | 109 | Downstream (1-2kb) | 58535576 | 58537471 | Cd276 |
| Treg_2_peak_3673 | 24.6074 | 52.2592 | 47.8487 | 170 | Intron (ENSMUST000001 | 60746358 | 60773359 | Cd28 |
| Treg_2_peak_25628 | 15.1108 | 29.9533 | 26.1103 | 169 | Distal Intergenic | 114890041 | 114904654 | Cd300a |
| Treg_2_peak_25629 | 4.20636 | 5.76872 | 3.36924 | 113 | Intron (ENSMUST000000 | 114890294 | 114893470 | Cd300a |
| Treg_2_peak_25631 | 3.68967 | 5.71003 | 3.33556 | 192 | Distal Intergenic | 114956116 | 114969157 | Cd300c |
| Treg_2_peak_25635 | 4.61717 | 7.516 | 4.89772 | 137 | Intron (ENSMUST000000 | 115051917 | 115062177 | Cd300e |
| Treg_2_peak_25632 | 4.16569 | 5.64327 | 3.33556 | 155 | Promoter (1-2kb) | 114982274 | 114989922 | Cd300ld |
| Treg_2_peak_25633 | 3.6359 | 5.41249 | 3.16527 | 197 | Promoter (2-3kb) | 114982274 | 114989922 | Cd300ld |
| Treg_2_peak_25634 | 4.61717 | 7.516 | 4.89772 | 117 | Promoter (2-3kb) | 115031486 | 115037769 | Cd300ld5 |
| Treg_2_peak_25637 | 5.45384 | 8.8909 | 6.19058 | 127 | Intron (ENSMUST000001 | 115116214 | 115133992 | Cd300lf |
| Treg_2_peak_25636 | 9.22417 | 17.3445 | 14.0296 | 151 | Promoter (<=1kb) | 115118848 | 115133991 | Cd300lf |
| Treg_2_peak_74524 | 3.6666 | 5.57696 | 3.31807 | 112 | Intron (ENSMUST000001 | 60251993 | 60284488 | Cd302 |
| Treg_2_peak_59452 | 3.6359 | 5.41249 | 3.16527 | 108 | Distal Intergenic | 33843426 | 33848751 | Cd320 |
| Treg_2_peak_11845 | 8.18077 | 14.5934 | 11.4246 | 126 | Distal Intergenic | 194938819 | 194961279 | Cd34 |
| Treg_2_peak_11846 | 3.6359 | 5.41249 | 3.16527 | 116 | Intron (ENSMUST000000 | 194938995 | 194958550 | Cd34 |
| Treg_2_peak_100601 | 3.69374 | 5.73445 | 3.33556 | 111 | Distal Intergenic | 17782016 | 17835696 | Cd36 |
| Treg_2_peak_100605 | 6.36282 | 10.7362 | 7.85556 | 119 | Distal Intergenic | 17814675 | 17888801 | Cd36 |
| Treg_2_peak_100606 | 5.24837 | 7.9747 | 5.34802 | 276 | Distal Intergenic | 17814675 | 17888801 | Cd36 |
| Treg_2_peak_100603 | 3.6359 | 5.41249 | 3.16527 | 115 | Intron (ENSMUST000001 | 17781690 | 17849792 | Cd36 |
| Treg_2_peak_100604 | 3.6359 | 5.41249 | 3.16527 | 96 | Intron (ENSMUST000001 | 17782367 | 17888747 | Cd36 |
| Treg_2_peak_100602 | 3.69374 | 5.73445 | 3.33556 | 112 | Promoter (1-2kb) | 17781690 | 17849792 | Cd36 |
| Treg_2_peak_102022 | 8.24985 | 14.967 | 11.7883 | 149 | Distal Intergenic | 43868553 | 43912375 | Cd38 |
| Treg_2_peak_102023 | 4.54487 | 7.11231 | 4.62813 | 135 | Distal Intergenic | 43868553 | 43912375 | Cd38 |
| Treg_2_peak_102024 | 3.69374 | 5.73445 | 3.33556 | 109 | Distal Intergenic | 43868553 | 43912375 | Cd38 |
| Treg_2_peak_102025 | 9.23434 | 17.4062 | 14.0484 | 194 | Promoter (2-3kb) | 43868860 | 43869740 | Cd38 |
| Treg_2_peak_134345 | 3.69374 | 5.73445 | 3.33556 | 96 | Promoter (1-2kb) | 44985823 | 44986421 | Cd3d |
| Treg_2_peak_134346 | 5.45384 | 8.8909 | 6.19058 | 153 | Promoter (1-2kb) | 44985952 | 44986422 | Cd3d |
| Treg_2_peak_134347 | 5.5406 | 9.37639 | 6.57036 | 121 | Distal Intergenic | 45003159 | 45009627 | Cd3e |
| Treg_2_peak_115095 | 6.04117 | 15.3112 | 12.0866 | 120 | Promoter (2-3kb) | 124867333 | 124888199 | Cd4 |
| Treg_2_peak_80904 | 9.16451 | 15.3628 | 12.1145 | 139 | Promoter (2-3kb) | 165055704 | 165071000 | Cd40 |
| Treg_2_peak_140914 | 5.5406 | 9.37639 | 6.57036 | 143 | Intron (ENSMUST000000 | 57212143 | 57224042 | Cd40lg |
| Treg_2_peak_77113 | 4.61717 | 7.516 | 4.89772 | 147 | Distal Intergenic | 102811141 | 102901665 | Cd44 |
| Treg_2_peak_77114 | 3.69374 | 5.73445 | 3.33556 | 150 | Distal Intergenic | 102811141 | 102901665 | Cd44 |
| Treg_2_peak_77115 | 3.69374 | 5.73445 | 3.33556 | 117 | Distal Intergenic | 102811141 | 102901665 | Cd44 |
| Treg_2_peak_77116 | 2.7703 | 4.04921 | 1.90871 | 110 | Distal Intergenic | 102811141 | 102901665 | Cd44 |
| Treg_2_peak_77117 | 3.69374 | 5.73445 | 3.33556 | 213 | Distal Intergenic | 102811141 | 102901665 | Cd44 |
| Treg_2_peak_11851 | 8.72992 | 14.989 | 11.8101 | 318 | Promoter (<=1kb) | 195036826 | 195092249 | Cd46 |
| Treg_2_peak_11852 | 4.99882 | 7.12189 | 4.62813 | 130 | Promoter (2-3kb) | 195036826 | 195092249 | Cd46 |
| Treg_2_peak_55081 | 3.69374 | 5.73445 | 3.33556 | 107 | Distal Intergenic | 49866833 | 49911091 | Cd47 |
| Treg_2_peak_55079 | 3.69374 | 5.73445 | 3.33556 | 102 | Intron (ENSMUST000002 | 49866833 | 49911091 | Cd47 |
| Treg_2_peak_55076 | 4.61717 | 7.516 | 4.89772 | 287 | Intron (ENSMUST000002 | 49800533 | 49911046 | Cd47 |
| Treg_2_peak_55077 | 4.61717 | 7.516 | 4.89772 | 119 | Intron (ENSMUST000002 | 49855366 | 49911091 | Cd47 |
| Treg_2_peak_55078 | 5.5406 | 9.37639 | 6.57036 | 145 | Promoter (<=1kb) | 49866833 | 49911091 | Cd47 |
| Treg_2_peak_87981 | 9.1665 | 17.0087 | 13.7075 | 177 | Intron (ENSMUST000000 | 106759921 | 106790149 | Cd53 |
| Treg_2_peak_87980 | 4.61717 | 7.516 | 4.89772 | 125 | Intron (ENSMUST000000 | 106759921 | 106790149 | Cd53 |
| Treg_2_peak_87982 | 7.38747 | 13.2885 | 10.182 | 144 | Promoter (1-2kb) | 106759921 | 106790149 | Cd53 |
| Treg_2_peak_8203 | 20.8284 | 42.7332 | 38.5282 | 173 | Distal Intergenic | 130419601 | 130422740 | Cd55b |
| Treg_2_peak_8204 | 8.18077 | 14.5934 | 11.4246 | 284 | Distal Intergenic | 130419601 | 130422740 | Cd55b |
| Treg_2_peak_77182 | 5.80017 | 8.57116 | 5.92547 | 110 | Intron (ENSMUST000001 | 104071066 | 104084820 | Cd59b |
| Treg_2_peak_77183 | 6.15184 | 8.89942 | 6.19613 | 333 | Intron (ENSMUST000001 | 104071066 | 104084820 | Cd59b |
| Treg_2_peak_86933 | 4.58325 | 7.31856 | 4.81351 | 102 | Distal Intergenic | 87357881 | 87371073 | Cd5l |
| Treg_2_peak_86934 | 7.3332 | 12.971 | 9.92589 | 164 | Distal Intergenic | 87357881 | 87371073 | Cd5l |
| Treg_2_peak_68447 | 4.49888 | 6.88526 | 4.45067 | 148 | Exon (ENSMUST0000023 | 10794564 | 10829856 | Cd6 |
| Treg_2_peak_68446 | 8.18077 | 14.5934 | 11.4246 | 130 | Intron (ENSMUST000000 | 10794564 | 10829856 | Cd6 |
| Treg_2_peak_65898 | 6.76901 | 10.5978 | 7.75479 | 127 | Promoter (<=1kb) | 50134726 | 50135600 | Cd63-ps |
| Treg_2_peak_65899 | 8.79978 | 15.2657 | 12.0779 | 237 | Promoter (<=1kb) | 50134726 | 50135600 | Cd63-ps |
| Treg_2_peak_65900 | 5.5406 | 9.37639 | 6.57036 | 169 | Promoter (1-2kb) | 50134795 | 50135511 | Cd63-ps |
| Treg_2_peak_115290 | 16.5688 | 34.9863 | 30.9936 | 161 | Distal Intergenic | 129267325 | 129275436 | Cd69 |
| Treg_2_peak_115291 | 3.69374 | 5.73445 | 3.33556 | 106 | Distal Intergenic | 129267325 | 129275436 | Cd69 |
| Treg_2_peak_115292 | 3.6666 | 5.57696 | 3.31807 | 109 | Distal Intergenic | 129267325 | 129275436 | Cd69 |
| Treg_2_peak_115289 | 2.69382 | 3.64824 | 1.70993 | 261 | Promoter (<=1kb) | 129267325 | 129275436 | Cd69 |
| Treg_2_peak_60623 | 3.69374 | 5.73445 | 3.33556 | 110 | Distal Intergenic | 57145997 | 57149777 | Cd70 |
| Treg_2_peak_60624 | 6.15984 | 9.81068 | 6.99648 | 140 | Distal Intergenic | 57145997 | 57149777 | Cd70 |
| Treg_2_peak_94068 | 6.46682 | 9.57675 | 6.76836 | 115 | Distal Intergenic | 43447724 | 43454628 | Cd72 |
| Treg_2_peak_117628 | 4.35632 | 6.2848 | 3.87192 | 138 | Distal Intergenic | 24897381 | 24902197 | Cd79a |
| Treg_2_peak_25187 | 6.39973 | 9.51766 | 6.71009 | 145 | Promoter (<=1kb) | 106311341 | 106314762 | Cd79b |
| Treg_2_peak_54331 | 3.69374 | 5.73445 | 3.33556 | 136 | Intron (ENSMUST000002 | 38459118 | 38486447 | Cd80 |
| Treg_2_peak_124255 | 3.07592 | 3.57312 | 1.64459 | 153 | Intron (ENSMUST000001 | 143052739 | 143067934 | Cd81 |
| Treg_2_peak_76459 | 5.68624 | 7.8863 | 5.26161 | 121 | Exon (ENSMUST0000013 | 93419154 | 93420287 | Cd82 |
| Treg_2_peak_76458 | 6.6651 | 10.2696 | 7.44017 | 155 | Intron (ENSMUST000001 | 93419154 | 93420287 | Cd82 |
| Treg_2_peak_76460 | 5.6567 | 7.84563 | 5.22151 | 105 | Promoter (<=1kb) | 93430127 | 93463140 | Cd82 |
| Treg_2_peak_35112 | 5.5406 | 9.37639 | 6.57036 | 123 | Distal Intergenic | 43784775 | 43803132 | Cd83 |
| Treg_2_peak_35113 | 3.59911 | 5.2315 | 3.03527 | 123 | Distal Intergenic | 43784775 | 43803132 | Cd83 |
| Treg_2_peak_35114 | 9.28197 | 15.1209 | 11.9376 | 201 | Distal Intergenic | 43784775 | 43803132 | Cd83 |
| Treg_2_peak_10496 | 3.69374 | 5.73445 | 3.33556 | 165 | Distal Intergenic | 171839697 | 171890718 | Cd84 |
| Treg_2_peak_10497 | 9.99872 | 18.6343 | 15.2353 | 158 | Distal Intergenic | 171839697 | 171890718 | Cd84 |

| | | | | | | | | | |
|---|---|---|---|---|---|---|---|---|---|
| Treg_2_peak_10498 | 4.60243 | 7.4283 | 4.89772 | 375 | Distal Intergenic | 171839697 | 171890718 | Cd84 | |
| Treg_2_peak_10499 | 4.85274 | 6.70311 | 4.27939 | 169 | Distal Intergenic | 171839697 | 171890718 | Cd84 | |
| Treg_2_peak_54242 | 25.1423 | 55.9747 | 51.4976 | 151 | Intron (ENSMUST00000( | 36603869 | 36666077 | Cd86 | |
| Treg_2_peak_54241 | 9.16451 | 15.3628 | 12.1145 | 194 | Intron (ENSMUST00000( | 36620713 | 36642805 | Cd86 | |
| Treg_2_peak_54240 | 4.16569 | 5.64327 | 3.33556 | 150 | Intron (ENSMUST00000( | 36603897 | 36604519 | Cd86 | |
| Treg_2_peak_54243 | 3.6359 | 5.41249 | 3.16527 | 103 | Promoter (<=1kb) | 36603869 | 36666077 | Cd86 | |
| Treg_2_peak_111871 | 11.6732 | 22.1172 | 18.5566 | 135 | Exon (ENSMUST0000006 | 71322788 | 71337494 | Cd8b1 | |
| Treg_2_peak_111870 | 3.33255 | 4.24299 | 2.08194 | 74 | Promoter (<=1kb) | 71322788 | 71337494 | Cd8b1 | |
| Treg_2_peak_115110 | 7.03982 | 11.5798 | 8.60681 | 137 | Intron (ENSMUST000002 | 125460266 | 125494791 | Cd9 | |
| Treg_2_peak_80020 | 16.8914 | 32.7027 | 28.7754 | 198 | Distal Intergenic | 148436640 | 148443563 | Cd93 | |
| Treg_2_peak_80021 | 4.54487 | 7.11231 | 4.62813 | 186 | Distal Intergenic | 148436640 | 148443563 | Cd93 | |
| Treg_2_peak_80019 | 9.99764 | 17.1409 | 13.8303 | 169 | Downstream (<1kb) | 148436640 | 148443563 | Cd93 | |
| Treg_2_peak_54819 | 5.45384 | 8.8909 | 6.19058 | 121 | Distal Intergenic | 46035657 | 46120251 | Cd96 | |
| Treg_2_peak_54818 | 4.51736 | 6.97411 | 4.53802 | 111 | Intron (ENSMUST00000( | 46035657 | 46120251 | Cd96 | |
| Treg_2_peak_69872 | 3.69374 | 5.73445 | 3.33556 | 112 | Distal Intergenic | 34300075 | 34316677 | Fas | |
| Treg_2_peak_69873 | 6.46404 | 11.3035 | 8.33659 | 167 | Distal Intergenic | 34300075 | 34316677 | Fas | |
| Treg_2_peak_69874 | 3.6359 | 5.41249 | 3.16527 | 94 | Distal Intergenic | 34300075 | 34316677 | Fas | |
| Treg_2_peak_69871 | 4.61717 | 7.516 | 4.89772 | 83 | Intron (ENSMUST00000( | 34300075 | 34316677 | Fas | |
| Treg_2_peak_69869 | 5.5406 | 9.37639 | 6.57036 | 159 | Promoter (1-2kb) | 34300075 | 34316677 | Fas | |
| Treg_2_peak_9912 | 3.69374 | 5.73445 | 3.33556 | 119 | Distal Intergenic | 161781422 | 161788358 | Fasl | |
| Treg_2_peak_9913 | 4.61717 | 7.516 | 4.89772 | 120 | Distal Intergenic | 161781422 | 161788358 | Fasl | |
| Treg_2_peak_9914 | 5.45384 | 8.8909 | 6.19058 | 131 | Promoter (2-3kb) | 161780689 | 161788495 | Fasl | |
| Treg_2_peak_18974 | 3.59176 | 5.19719 | 3.00172 | 112 | Distal Intergenic | 118441046 | 118445892 | Ifng | |
| Treg_2_peak_18975 | 14.367 | 28.4773 | 24.6642 | 205 | Distal Intergenic | 118441046 | 118445892 | Ifng | |
| Treg_2_peak_18976 | 3.69374 | 5.73445 | 3.33556 | 108 | Distal Intergenic | 118441046 | 118445892 | Ifng | |
| Treg_2_peak_18977 | 4.99882 | 7.12189 | 4.62813 | 146 | Distal Intergenic | 118441046 | 118445892 | Ifng | |
| Treg_2_peak_18987 | 4.61717 | 7.516 | 4.89772 | 129 | Distal Intergenic | 118502035 | 118556525 | Ifngas1 | |
| Treg_2_peak_18988 | 4.45334 | 6.67838 | 4.25491 | 121 | Distal Intergenic | 118502035 | 118556525 | Ifngas1 | |
| Treg_2_peak_18989 | 3.69374 | 5.73445 | 3.33556 | 150 | Distal Intergenic | 118502035 | 118556525 | Ifngas1 | |
| Treg_2_peak_18990 | 30.9051 | 72.4915 | 67.7773 | 166 | Distal Intergenic | 118502035 | 118556525 | Ifngas1 | |
| Treg_2_peak_18991 | 9.99872 | 18.6343 | 15.2353 | 236 | Distal Intergenic | 118502035 | 118556525 | Ifngas1 | |
| Treg_2_peak_18985 | 5.27987 | 8.0993 | 5.46746 | 142 | Intron (ENSMUST000002 | 118502035 | 118555352 | Ifngas1 | |
| Treg_2_peak_18986 | 4.4897 | 6.84221 | 4.40836 | 272 | Intron (ENSMUST000002 | 118502035 | 118555352 | Ifngas1 | |
| Treg_2_peak_18984 | 12.6919 | 23.2946 | 19.6948 | 155 | Intron (ENSMUST000002 | 118502035 | 118555352 | Ifngas1 | |
| Treg_2_peak_18983 | 3.69374 | 5.73445 | 3.33556 | 110 | Intron (ENSMUST000002 | 118502035 | 118555352 | Ifngas1 | |
| Treg_2_peak_12912 | 3.69374 | 5.73445 | 3.33556 | 102 | Distal Intergenic | 19591949 | 19610229 | Ifngr1 | |
| Treg_2_peak_12913 | 2.72692 | 3.80885 | 1.81786 | 59 | Distal Intergenic | 19591949 | 19610229 | Ifngr1 | |
| Treg_2_peak_12914 | 10.7533 | 19.9563 | 16.495 | 136 | Distal Intergenic | 19605919 | 19609256 | Ifngr1 | |
| Treg_2_peak_12915 | 7.76448 | 12.8063 | 9.76846 | 146 | Distal Intergenic | 19605919 | 19609256 | Ifngr1 | |
| Treg_2_peak_57647 | 4.99798 | 6.6355 | 4.21298 | 109 | Downstream (1-2kb) | 91561323 | 91565169 | Ifngr2 | |
| Treg_2_peak_57646 | 6.24064 | 8.99819 | 6.29309 | 119 | Promoter (2-3kb) | 91547072 | 91565623 | Ifngr2 | |
| Treg_2_peak_8244 | 4.54487 | 7.11231 | 4.62813 | 102 | Distal Intergenic | 131019845 | 131024974 | Il10 | |
| Treg_2_peak_8245 | 3.69374 | 5.73445 | 3.33556 | 96 | Distal Intergenic | 131019845 | 131024974 | Il10 | |
| Treg_2_peak_8246 | 4.54487 | 7.11231 | 4.62813 | 199 | Distal Intergenic | 131019845 | 131024974 | Il10 | |
| Treg_2_peak_8247 | 4.4897 | 6.84221 | 4.40836 | 116 | Distal Intergenic | 131019845 | 131024974 | Il10 | |
| Treg_2_peak_8248 | 4.98095 | 7.06806 | 4.62813 | 111 | Distal Intergenic | 131019845 | 131024974 | Il10 | |
| Treg_2_peak_94026 | 10.388 | 18.1473 | 14.7785 | 165 | Promoter (<=1kb) | 41767799 | 41769474 | Il11ra1 | |
| Treg_2_peak_85743 | 4.61717 | 7.516 | 4.89772 | 123 | Distal Intergenic | 68690644 | 68698547 | Il12a | |
| Treg_2_peak_85744 | 5.5406 | 9.37639 | 6.57036 | 116 | Distal Intergenic | 68690644 | 68698547 | Il12a | |
| Treg_2_peak_85746 | 6.15184 | 8.89942 | 6.19613 | 462 | Distal Intergenic | 68694021 | 68698547 | Il12a | |
| Treg_2_peak_85745 | 3.62133 | 5.33889 | 3.14027 | 94 | Promoter (2-3kb) | 68690644 | 68698547 | Il12a | |
| Treg_2_peak_22154 | 3.69374 | 5.73445 | 3.33556 | 119 | Distal Intergenic | 44400063 | 44414033 | Il12b | |
| Treg_2_peak_22152 | 3.69374 | 5.73445 | 3.33556 | 99 | Exon (ENSMUST0000017 | 44400063 | 44414033 | Il12b | |
| Treg_2_peak_22153 | 4.61717 | 7.516 | 4.89772 | 118 | Intron (ENSMUST000001 | 44400063 | 44414033 | Il12b | |
| Treg_2_peak_111628 | 14.7749 | 30.6564 | 26.7723 | 151 | Intron (ENSMUST00000( | 67291318 | 67339694 | Il12rb2 | |
| Treg_2_peak_111626 | 7.38747 | 13.2885 | 10.182 | 181 | Intron (ENSMUST000001 | 67291318 | 67339694 | Il12rb2 | |
| Treg_2_peak_111627 | 3.69374 | 5.73445 | 3.33556 | 138 | Intron (ENSMUST000001 | 67291318 | 67339694 | Il12rb2 | |
| Treg_2_peak_111625 | 3.69374 | 5.73445 | 3.33556 | 186 | Intron (ENSMUST000001 | 67291318 | 67339694 | Il12rb2 | |
| Treg_2_peak_139991 | 5.5406 | 9.37639 | 6.57036 | 128 | Distal Intergenic | 36112110 | 36171259 | Il13ra1 | |
| Treg_2_peak_139992 | 9.99872 | 18.6343 | 15.2353 | 140 | Distal Intergenic | 36112110 | 36171259 | Il13ra1 | |
| Treg_2_peak_139993 | 3.69374 | 5.73445 | 3.33556 | 110 | Intron (ENSMUST00000( | 36112110 | 36171259 | Il13ra1 | |
| Treg_2_peak_145119 | 4.65586 | 6.20186 | 3.79026 | 106 | Distal Intergenic | 147383478 | 147403832 | Il13ra2 | |
| Treg_2_peak_145120 | 7.97318 | 12.3792 | 9.37861 | 121 | Distal Intergenic | 147383478 | 147403832 | Il13ra2 | |
| Treg_2_peak_145121 | 3.69374 | 5.73445 | 3.33556 | 131 | Distal Intergenic | 147383478 | 147403832 | Il13ra2 | |
| Treg_2_peak_145122 | 3.69374 | 5.73445 | 3.33556 | 143 | Distal Intergenic | 147383478 | 147403832 | Il13ra2 | |
| Treg_2_peak_145123 | 3.8449 | 4.79884 | 2.62253 | 109 | Distal Intergenic | 147383478 | 147403832 | Il13ra2 | |
| Treg_2_peak_145124 | 5.5406 | 9.37639 | 6.57036 | 131 | Distal Intergenic | 147383478 | 147403832 | Il13ra2 | |
| Treg_2_peak_145125 | 3.69374 | 5.73445 | 3.33556 | 97 | Distal Intergenic | 147383478 | 147403832 | Il13ra2 | |
| Treg_2_peak_145126 | 5.83196 | 8.66693 | 6.00946 | 138 | Distal Intergenic | 147383478 | 147403832 | Il13ra2 | |
| Treg_2_peak_145127 | 2.7703 | 4.04921 | 1.90871 | 46 | Distal Intergenic | 147383478 | 147403832 | Il13ra2 | |
| Treg_2_peak_145128 | 4.61717 | 7.516 | 4.89772 | 107 | Distal Intergenic | 147383478 | 147403832 | Il13ra2 | |
| Treg_2_peak_145130 | 4.54487 | 7.11231 | 4.62813 | 137 | Intron (ENSMUST00000( | 147383478 | 147403832 | Il13ra2 | |
| Treg_2_peak_145129 | 2.72692 | 3.80885 | 1.81786 | 112 | Promoter (1-2kb) | 147383478 | 147403832 | Il13ra2 | |
| Treg_2_peak_129177 | 5.5406 | 9.37639 | 6.57036 | 76 | Distal Intergenic | 82331914 | 82344473 | Il15 | |
| Treg_2_peak_129183 | 2.72692 | 3.80885 | 1.81786 | 112 | Intron (ENSMUST000002 | 82345662 | 82398627 | Il15 | |
| Treg_2_peak_129179 | 3.69374 | 5.73445 | 3.33556 | 103 | Intron (ENSMUST000002 | 82331739 | 82345683 | Il15 | |
| Treg_2_peak_129180 | 2.72692 | 3.80885 | 1.81786 | 127 | Intron (ENSMUST000002 | 82331739 | 82345683 | Il15 | |
| Treg_2_peak_129181 | 5.5406 | 9.37639 | 6.57036 | 133 | Intron (ENSMUST000002 | 82331739 | 82345683 | Il15 | |
| Treg_2_peak_129182 | 3.69374 | 5.73445 | 3.33556 | 103 | Intron (ENSMUST000002 | 82331739 | 82345683 | Il15 | |
| Treg_2_peak_129178 | 3.69374 | 5.73445 | 3.33556 | 99 | Intron (ENSMUST000002 | 82331914 | 82344473 | Il15 | |
| Treg_2_peak_129184 | 15.5595 | 30.3521 | 26.5025 | 135 | Promoter (<=1kb) | 82345662 | 82398627 | Il15 | |
| Treg_2_peak_71792 | 3.6359 | 5.41249 | 3.16527 | 196 | Promoter (<=1kb) | 11718431 | 11733417 | Il15ra | |
| Treg_2_peak_120953 | 3.6666 | 5.57696 | 3.31807 | 225 | Distal Intergenic | 83660897 | 83745726 | Il16 | |
| Treg_2_peak_120951 | 8.09799 | 14.1818 | 11.045 | 202 | Intron (ENSMUST00000( | 83722510 | 83732407 | Il16 | |
| Treg_2_peak_120947 | 5.5406 | 9.37639 | 6.57036 | 203 | Promoter (<=1kb) | 83649576 | 83655325 | Il16 | |
| Treg_2_peak_120952 | 3.5699 | 5.09832 | 2.91222 | 380 | Promoter (2-3kb) | 83722510 | 83732407 | Il16 | |
| Treg_2_peak_1286 | 3.69374 | 5.73445 | 3.33556 | 359 | Promoter (<=1kb) | 20777679 | 20779554 | Il17f | |
| Treg_2_peak_1285 | 9.08974 | 16.5931 | 13.3034 | 174 | Promoter (2-3kb) | 20777679 | 20779554 | Il17f | |
| Treg_2_peak_114818 | 3.59176 | 5.19719 | 3.00172 | 148 | Distal Intergenic | 120463247 | 120483729 | Il17ra | |

| | | | | | | | | |
|---|---|---|---|---|---|---|---|---|
| Treg_2_peak_114819 | 5.5406 | 9.37639 | 6.57036 | 121 | Distal Intergenic | 120463247 | 120483729 | Il17ra |
| Treg_2_peak_114820 | 9.08974 | 16.5931 | 13.3034 | 205 | Distal Intergenic | 120463247 | 120483729 | Il17ra |
| Treg_2_peak_41166 | 3.68967 | 5.71003 | 3.33556 | 116 | Promoter (<=1kb) | 29996471 | 30000094 | Il17rb |
| Treg_2_peak_41167 | 6.45965 | 9.36103 | 6.57036 | 139 | Promoter (2-3kb) | 29996199 | 30008809 | Il17rb |
| Treg_2_peak_40973 | 8.18077 | 14.5934 | 11.4246 | 495 | Intron (ENSMUST000000 | 27067397 | 27100976 | Il17rd |
| Treg_2_peak_40974 | 4.99882 | 7.12189 | 4.62813 | 110 | Intron (ENSMUST000000 | 27067397 | 27100976 | Il17rd |
| Treg_2_peak_40975 | 4.4897 | 6.84221 | 4.40836 | 203 | Intron (ENSMUST000000 | 27067397 | 27100976 | Il17rd |
| Treg_2_peak_40976 | 4.16569 | 5.64327 | 3.33556 | 116 | Intron (ENSMUST000000 | 27067397 | 27100976 | Il17rd |
| Treg_2_peak_40977 | 6.28558 | 10.3563 | 7.5188 | 132 | Intron (ENSMUST000000 | 27087765 | 27101491 | Il17rd |
| Treg_2_peak_40978 | 10.9077 | 20.7131 | 17.2181 | 148 | Promoter (1-2kb) | 27087765 | 27101491 | Il17rd |
| Treg_2_peak_114479 | 3.29576 | 4.13512 | 1.99322 | 32 | Distal Intergenic | 113458484 | 113470758 | Il17re |
| Treg_2_peak_134606 | 4.61717 | 7.516 | 4.89772 | 117 | Intron (ENSMUST000001 | 50554827 | 50581840 | Il18 |
| Treg_2_peak_134607 | 4.61717 | 7.516 | 4.89772 | 246 | Promoter (2-3kb) | 50565374 | 50581837 | Il18 |
| Treg_2_peak_2439 | 4.54487 | 7.11231 | 4.62813 | 134 | Intron (ENSMUST000001 | 40466006 | 40500854 | Il18r1 |
| Treg_2_peak_2440 | 4.61717 | 7.516 | 4.89772 | 165 | Intron (ENSMUST000001 | 40466006 | 40500854 | Il18r1 |
| Treg_2_peak_2441 | 4.54487 | 7.11231 | 4.62813 | 89 | Intron (ENSMUST000001 | 40466006 | 40500854 | Il18r1 |
| Treg_2_peak_2446 | 3.6359 | 5.41249 | 3.16527 | 139 | Distal Intergenic | 40541692 | 40547903 | Il18rap |
| Treg_2_peak_2444 | 3.6359 | 5.41249 | 3.16527 | 153 | Exon (ENSMUST0000015 | 40515448 | 40526917 | Il18rap |
| Treg_2_peak_2442 | 9.16451 | 15.3628 | 12.1145 | 491 | Intron (ENSMUST000000 | 40515362 | 40551705 | Il18rap |
| Treg_2_peak_2443 | 4.54487 | 7.11231 | 4.62813 | 129 | Intron (ENSMUST000000 | 40515362 | 40551705 | Il18rap |
| Treg_2_peak_2445 | 3.69374 | 5.73445 | 3.33556 | 102 | Promoter (1-2kb) | 40541692 | 40547903 | Il18rap |
| Treg_2_peak_8239 | 5.5406 | 9.37639 | 6.57036 | 388 | Distal Intergenic | 130932816 | 130940115 | Il19 |
| Treg_2_peak_8240 | 9.1665 | 17.0087 | 13.7075 | 159 | Distal Intergenic | 130932816 | 130940115 | Il19 |
| Treg_2_peak_8241 | 3.69374 | 5.73445 | 3.33556 | 126 | Distal Intergenic | 130932816 | 130940115 | Il19 |
| Treg_2_peak_8242 | 4.58325 | 7.31856 | 4.81351 | 123 | Distal Intergenic | 130932816 | 130940115 | Il19 |
| Treg_2_peak_8243 | 6.42995 | 11.1022 | 8.20899 | 128 | Distal Intergenic | 130932816 | 130940115 | Il19 |
| Treg_2_peak_8237 | 4.54487 | 7.11231 | 4.62813 | 128 | Exon (ENSMUST0000018 | 130932658 | 130939241 | Il19 |
| Treg_2_peak_78744 | 3.6359 | 5.41249 | 3.16527 | 172 | Promoter (<=1kb) | 129369897 | 129375748 | Il1bos |
| Treg_2_peak_72630 | 5.38286 | 7.47221 | 4.89772 | 120 | Distal Intergenic | 24291196 | 24293820 | Il1f10 |
| Treg_2_peak_72628 | 3.6359 | 5.41249 | 3.16527 | 134 | Promoter (<=1kb) | 24277282 | 24281629 | Il1f5 |
| Treg_2_peak_72619 | 6.36282 | 10.7362 | 7.85556 | 151 | Intron (ENSMUST000000 | 24215505 | 24225525 | Il1f6 |
| Treg_2_peak_72620 | 4.99882 | 7.12189 | 4.62813 | 125 | Intron (ENSMUST000000 | 24215505 | 24225525 | Il1f6 |
| Treg_2_peak_72618 | 3.6666 | 5.57696 | 3.31807 | 108 | Promoter (1-2kb) | 24215505 | 24225525 | Il1f6 |
| Treg_2_peak_72613 | 6.46404 | 11.3035 | 8.33659 | 239 | Distal Intergenic | 24153161 | 24160519 | Il1f8 |
| Treg_2_peak_72614 | 5.45384 | 8.8909 | 6.19058 | 207 | Distal Intergenic | 24153161 | 24160519 | Il1f8 |
| Treg_2_peak_72615 | 6.36282 | 10.7362 | 7.85556 | 151 | Distal Intergenic | 24153161 | 24160519 | Il1f8 |
| Treg_2_peak_72617 | 5.68243 | 8.23372 | 5.6002 | 186 | Distal Intergenic | 24153161 | 24160519 | Il1f8 |
| Treg_2_peak_72616 | 4.61717 | 7.516 | 4.89772 | 222 | Promoter (<=1kb) | 24153161 | 24160519 | Il1f8 |
| Treg_2_peak_2421 | 3.6359 | 5.41249 | 3.16527 | 131 | Distal Intergenic | 40225080 | 40317257 | Il1r1 |
| Treg_2_peak_2422 | 3.69374 | 5.73445 | 3.33556 | 104 | Distal Intergenic | 40225080 | 40317257 | Il1r1 |
| Treg_2_peak_2423 | 4.61717 | 7.516 | 4.89772 | 127 | Distal Intergenic | 40225080 | 40317257 | Il1r1 |
| Treg_2_peak_2424 | 3.69374 | 5.73445 | 3.33556 | 255 | Promoter (<=1kb) | 40225080 | 40317257 | Il1r1 |
| Treg_2_peak_2425 | 3.68967 | 5.71003 | 3.33556 | 57 | Promoter (1-2kb) | 40225080 | 40317257 | Il1r1 |
| Treg_2_peak_2411 | 8.3109 | 15.3245 | 12.0866 | 127 | Distal Intergenic | 40074079 | 40112136 | Il1r2 |
| Treg_2_peak_2412 | 9.53279 | 15.651 | 12.4006 | 178 | Promoter (<=1kb) | 40074079 | 40112136 | Il1r2 |
| Treg_2_peak_2413 | 5.4999 | 9.13896 | 6.42463 | 128 | Promoter (<=1kb) | 40084698 | 40125231 | Il1r2 |
| Treg_2_peak_53715 | 6.46404 | 11.3035 | 8.33659 | 213 | Distal Intergenic | 26581704 | 26725264 | Il1rap |
| Treg_2_peak_53716 | 3.69374 | 5.73445 | 3.33556 | 123 | Intron (ENSMUST000000 | 26624156 | 26715068 | Il1rap |
| Treg_2_peak_53717 | 7.38747 | 13.2885 | 10.182 | 119 | Intron (ENSMUST000000 | 26624156 | 26715068 | Il1rap |
| Treg_2_peak_53720 | 3.69374 | 5.73445 | 3.33556 | 94 | Intron (ENSMUST000000 | 26722434 | 26730117 | Il1rap |
| Treg_2_peak_53718 | 6.36282 | 10.7362 | 7.85556 | 178 | Intron (ENSMUST000000 | 26624156 | 26715068 | Il1rap |
| Treg_2_peak_53719 | 5.5406 | 9.37639 | 6.57036 | 118 | Intron (ENSMUST000000 | 26624156 | 26715068 | Il1rap |
| Treg_2_peak_53723 | 17.0609 | 35.0754 | 31.0786 | 277 | Intron (ENSMUST000001 | 26727042 | 26728228 | Il1rap |
| Treg_2_peak_53722 | 3.6359 | 5.41249 | 3.16527 | 196 | Promoter (<=1kb) | 26722434 | 26730117 | Il1rap |
| Treg_2_peak_142371 | 4.61717 | 7.516 | 4.89772 | 141 | Intron (ENSMUST000001 | 86740870 | 88115645 | Il1rapl1 |
| Treg_2_peak_142370 | 3.69374 | 5.73445 | 3.33556 | 172 | Intron (ENSMUST000001 | 86740870 | 88115645 | Il1rapl1 |
| Treg_2_peak_142357 | 4.41756 | 6.52649 | 4.10642 | 135 | Intron (ENSMUST000001 | 86747242 | 87890235 | Il1rapl1 |
| Treg_2_peak_142358 | 9.23434 | 17.4062 | 14.0484 | 179 | Intron (ENSMUST000001 | 86747242 | 87890235 | Il1rapl1 |
| Treg_2_peak_144669 | 5.5406 | 9.37639 | 6.57036 | 138 | Distal Intergenic | 137570608 | 138846946 | Il1rapl2 |
| Treg_2_peak_144670 | 5.25883 | 8.01554 | 5.38817 | 122 | Distal Intergenic | 137570608 | 138846946 | Il1rapl2 |
| Treg_2_peak_144671 | 4.61717 | 7.516 | 4.89772 | 116 | Distal Intergenic | 137570608 | 138846946 | Il1rapl2 |
| Treg_2_peak_144672 | 3.69374 | 5.73445 | 3.33556 | 110 | Distal Intergenic | 137570608 | 138846946 | Il1rapl2 |
| Treg_2_peak_144673 | 3.69374 | 5.73445 | 3.33556 | 112 | Distal Intergenic | 137570608 | 138846946 | Il1rapl2 |
| Treg_2_peak_144674 | 8.18077 | 14.5934 | 11.4246 | 166 | Distal Intergenic | 137570608 | 138846946 | Il1rapl2 |
| Treg_2_peak_144675 | 3.69374 | 5.73445 | 3.33556 | 116 | Distal Intergenic | 137570608 | 138846946 | Il1rapl2 |
| Treg_2_peak_144676 | 4.61717 | 7.516 | 4.89772 | 157 | Distal Intergenic | 137570608 | 138846946 | Il1rapl2 |
| Treg_2_peak_144677 | 8.3109 | 15.3245 | 12.0866 | 217 | Distal Intergenic | 137570608 | 138846946 | Il1rapl2 |
| Treg_2_peak_144678 | 4.61717 | 7.516 | 4.89772 | 107 | Distal Intergenic | 137570608 | 138846946 | Il1rapl2 |
| Treg_2_peak_144679 | 3.69374 | 5.73445 | 3.33556 | 165 | Distal Intergenic | 137570608 | 138846946 | Il1rapl2 |
| Treg_2_peak_144680 | 17.4527 | 37.1153 | 33.0614 | 172 | Intron (ENSMUST000001 | 137571019 | 138506311 | Il1rapl2 |
| Treg_2_peak_144681 | 4.61717 | 7.516 | 4.89772 | 243 | Intron (ENSMUST000001 | 137571019 | 138506311 | Il1rapl2 |
| Treg_2_peak_144682 | 2.7703 | 4.04921 | 1.90871 | 134 | Intron (ENSMUST000001 | 137571019 | 138506311 | Il1rapl2 |
| Treg_2_peak_144687 | 4.61717 | 7.516 | 4.89772 | 140 | Intron (ENSMUST000001 | 137651720 | 138846624 | Il1rapl2 |
| Treg_2_peak_144685 | 4.61717 | 7.516 | 4.89772 | 228 | Intron (ENSMUST000001 | 137651720 | 138846624 | Il1rapl2 |
| Treg_2_peak_144686 | 6.46404 | 11.3035 | 8.33659 | 679 | Intron (ENSMUST000001 | 137651720 | 138846624 | Il1rapl2 |
| Treg_2_peak_2433 | 7.38747 | 13.2885 | 10.182 | 130 | Distal Intergenic | 40429570 | 40446723 | Il1rl1 |
| Treg_2_peak_2434 | 8.79978 | 15.2657 | 12.0779 | 638 | Distal Intergenic | 40429570 | 40446723 | Il1rl1 |
| Treg_2_peak_2435 | 12.9281 | 26.1132 | 22.3932 | 273 | Distal Intergenic | 40429570 | 40446723 | Il1rl1 |
| Treg_2_peak_2436 | 7.27179 | 12.6392 | 9.60486 | 130 | Distal Intergenic | 40429570 | 40446723 | Il1rl1 |
| Treg_2_peak_2437 | 4.54487 | 7.11231 | 4.62813 | 101 | Distal Intergenic | 40429570 | 40446723 | Il1rl1 |
| Treg_2_peak_2438 | 8.3109 | 15.3245 | 12.0866 | 372 | Intron (ENSMUST000000 | 40440628 | 40465396 | Il1rl1 |
| Treg_2_peak_2428 | 6.6651 | 10.2696 | 7.44017 | 213 | Distal Intergenic | 40324610 | 40329441 | Il1rl2 |
| Treg_2_peak_2429 | 4.43538 | 6.60105 | 4.17962 | 158 | Distal Intergenic | 40324610 | 40329441 | Il1rl2 |
| Treg_2_peak_2427 | 8.3109 | 15.3245 | 12.0866 | 142 | Intron (ENSMUST000000 | 40324610 | 40329441 | Il1rl2 |
| Treg_2_peak_2430 | 6.26002 | 10.2388 | 7.41941 | 166 | Intron (ENSMUST000000 | 40325611 | 40329171 | Il1rl2 |
| Treg_2_peak_72633 | 4.61717 | 7.516 | 4.89772 | 137 | Intron (ENSMUST000001 | 24345288 | 24349807 | Il1rn |
| Treg_2_peak_83703 | 7.38747 | 13.2885 | 10.182 | 176 | Distal Intergenic | 37120523 | 37125959 | Il2 |
| Treg_2_peak_8234 | 7.49823 | 11.9232 | 8.93461 | 128 | Distal Intergenic | 130907415 | 130911226 | Il20 |
| Treg_2_peak_8235 | 3.69374 | 5.73445 | 3.33556 | 109 | Distal Intergenic | 130907415 | 130911226 | Il20 |

| | | | | | | | | |
|---|---|---|---|---|---|---|---|---|
| Treg_2_peak_8236 | 6.36282 | 10.7362 | 7.85556 | 140 | Distal Intergenic | 130906985 | 130911451 | Il20 |
| Treg_2_peak_123405 | 3.59176 | 5.19719 | 3.00172 | 109 | Intron (ENSMUST000002 | 125603537 | 125633570 | Il21r |
| Treg_2_peak_123406 | 2.74995 | 3.9316 | 1.90871 | 83 | Intron (ENSMUST000002 | 125603537 | 125633570 | Il21r |
| Treg_2_peak_18964 | 5.38764 | 8.56594 | 5.92038 | 165 | Distal Intergenic | 118204942 | 118210047 | Il22 |
| Treg_2_peak_12916 | 4.16569 | 5.64327 | 3.33556 | 110 | Promoter (<=1kb) | 19621998 | 19634681 | Il22ra2 |
| Treg_2_peak_12917 | 5.83196 | 8.66693 | 6.00946 | 115 | Promoter (2-3kb) | 19621998 | 19634681 | Il22ra2 |
| Treg_2_peak_111636 | 3.65898 | 5.53485 | 3.28437 | 138 | Distal Intergenic | 67422932 | 67491855 | Il23r |
| Treg_2_peak_111635 | 19.9974 | 43.0789 | 38.8611 | 223 | Intron (ENSMUST000001 | 67422932 | 67491855 | Il23r |
| Treg_2_peak_8233 | 4.61717 | 7.516 | 4.89772 | 158 | Promoter (<=1kb) | 130882455 | 130887339 | Il24 |
| Treg_2_peak_123435 | 6.35594 | 9.15023 | 6.43567 | 174 | Distal Intergenic | 126589010 | 126594941 | Il27 |
| Treg_2_peak_123436 | 7.38747 | 13.2885 | 10.182 | 217 | Distal Intergenic | 126589010 | 126594941 | Il27 |
| Treg_2_peak_129277 | 8.40711 | 13.2819 | 10.182 | 112 | Intron (ENSMUST000000 | 84041769 | 84042540 | Il27ra |
| Treg_2_peak_71789 | 4.61717 | 7.516 | 4.89772 | 121 | Intron (ENSMUST000000 | 11642849 | 11648768 | Il2ra |
| Treg_2_peak_71788 | 13.0727 | 23.5527 | 19.9453 | 139 | Promoter (2-3kb) | 11642807 | 11693193 | Il2ra |
| Treg_2_peak_51239 | 8.1207 | 12.9123 | 9.87353 | 248 | Promoter (2-3kb) | 78481324 | 78493756 | Il2rb |
| Treg_2_peak_22666 | 3.69374 | 5.73445 | 3.33556 | 130 | Downstream (1-2kb) | 54265303 | 54267277 | Il3 |
| Treg_2_peak_22665 | 5.45384 | 8.8909 | 6.19058 | 129 | Downstream (2-3kb) | 54265303 | 54267277 | Il3 |
| Treg_2_peak_22667 | 7.38747 | 13.2885 | 10.182 | 143 | Promoter (<=1kb) | 54265303 | 54267277 | Il3 |
| Treg_2_peak_39187 | 5.81111 | 8.60389 | 5.95757 | 115 | Intron (ENSMUST000002 | 112546319 | 112565666 | Il31ra |
| Treg_2_peak_39185 | 3.67426 | 5.62015 | 3.33556 | 110 | Intron (ENSMUST000002 | 112523557 | 112562074 | Il31ra |
| Treg_2_peak_39186 | 12.138 | 21.4698 | 17.9541 | 130 | Intron (ENSMUST000002 | 112523557 | 112562074 | Il31ra |
| Treg_2_peak_39184 | 5.5406 | 9.37639 | 6.57036 | 133 | Intron (ENSMUST000002 | 112525846 | 112532344 | Il31ra |
| Treg_2_peak_39183 | 15.8296 | 30.4365 | 26.5795 | 148 | Intron (ENSMUST000002 | 112525846 | 112532344 | Il31ra |
| Treg_2_peak_69596 | 3.69374 | 5.73445 | 3.33556 | 125 | Distal Intergenic | 29925114 | 29960718 | Il33 |
| Treg_2_peak_69597 | 3.51991 | 4.88833 | 2.70632 | 111 | Distal Intergenic | 29925114 | 29960718 | Il33 |
| Treg_2_peak_69599 | 7.38747 | 13.2885 | 10.182 | 114 | Distal Intergenic | 29951816 | 29952813 | Il33 |
| Treg_2_peak_40283 | 4.93439 | 6.93139 | 4.49599 | 479 | Distal Intergenic | 14349621 | 14350757 | Il3ra |
| Treg_2_peak_22638 | 8.79923 | 14.683 | 11.5122 | 165 | Distal Intergenic | 53612460 | 53618669 | Il4 |
| Treg_2_peak_22639 | 7.5502 | 11.5246 | 8.55523 | 189 | Distal Intergenic | 53612460 | 53618669 | Il4 |
| Treg_2_peak_22637 | 7.49823 | 11.9232 | 8.93461 | 121 | Intron (ENSMUST000001 | 53602982 | 53617224 | Il4 |
| Treg_2_peak_114026 | 2.72692 | 3.80885 | 1.81786 | 188 | Intron (ENSMUST000002 | 106711975 | 106745109 | Il5ra |
| Treg_2_peak_114027 | 3.69374 | 5.73445 | 3.33556 | 89 | Intron (ENSMUST000002 | 106711975 | 106745109 | Il5ra |
| Treg_2_peak_114028 | 3.69374 | 5.73445 | 3.33556 | 107 | Promoter (1-2kb) | 106710357 | 106749037 | Il5ra |
| Treg_2_peak_87031 | 5.07676 | 7.36654 | 4.86038 | 117 | Distal Intergenic | 89864059 | 89913196 | Il6ra |
| Treg_2_peak_87032 | 4.4897 | 6.84221 | 4.40836 | 97 | Distal Intergenic | 89864059 | 89913196 | Il6ra |
| Treg_2_peak_39181 | 5.45384 | 8.8909 | 6.19058 | 142 | Intron (ENSMUST000001 | 112475046 | 112504445 | Il6st |
| Treg_2_peak_39182 | 4.16569 | 5.64327 | 3.33556 | 201 | Intron (ENSMUST000001 | 112475046 | 112504445 | Il6st |
| Treg_2_peak_39180 | 7.97526 | 12.4465 | 9.44523 | 214 | Promoter (<=1kb) | 112467593 | 112480057 | Il6st |
| Treg_2_peak_81823 | 10.0832 | 19.0917 | 15.6769 | 127 | Distal Intergenic | 7573182 | 7613760 | Il7 |
| Treg_2_peak_81824 | 9.99764 | 17.1409 | 13.8303 | 152 | Distal Intergenic | 7573182 | 7613760 | Il7 |
| Treg_2_peak_81825 | 2.69382 | 3.64824 | 1.70993 | 141 | Distal Intergenic | 7573182 | 7613760 | Il7 |
| Treg_2_peak_81822 | 3.6359 | 5.41249 | 3.16527 | 152 | Exon (ENSMUST0000018 | 7573182 | 7613760 | Il7 |
| Treg_2_peak_81813 | 3.6666 | 5.57696 | 3.31807 | 109 | Exon (ENSMUST0000019 | 7572028 | 7605864 | Il7 |
| Treg_2_peak_81815 | 5.5406 | 9.37639 | 6.57036 | 149 | Intron (ENSMUST000001 | 7573182 | 7613760 | Il7 |
| Treg_2_peak_81816 | 3.69374 | 5.73445 | 3.33556 | 111 | Intron (ENSMUST000001 | 7573182 | 7613760 | Il7 |
| Treg_2_peak_81817 | 4.61717 | 7.516 | 4.89772 | 153 | Intron (ENSMUST000001 | 7573182 | 7613760 | Il7 |
| Treg_2_peak_81818 | 3.6359 | 5.41249 | 3.16527 | 103 | Intron (ENSMUST000001 | 7573182 | 7613760 | Il7 |
| Treg_2_peak_81819 | 3.69374 | 5.73445 | 3.33556 | 116 | Intron (ENSMUST000001 | 7573182 | 7613760 | Il7 |
| Treg_2_peak_81820 | 5.83196 | 8.66693 | 6.00946 | 152 | Intron (ENSMUST000001 | 7573182 | 7613760 | Il7 |
| Treg_2_peak_81821 | 3.69374 | 5.73445 | 3.33556 | 129 | Intron (ENSMUST000001 | 7573182 | 7613760 | Il7 |
| Treg_2_peak_81814 | 2.7703 | 4.04921 | 1.90871 | 68 | Intron (ENSMUST000001 | 7572028 | 7605864 | Il7 |
| Treg_2_peak_47126 | 5.0573 | 7.30389 | 4.8066 | 101 | Distal Intergenic | 9516409 | 9530176 | Il7r |
| Treg_2_peak_47127 | 3.69374 | 5.73445 | 3.33556 | 122 | Distal Intergenic | 9516409 | 9530176 | Il7r |
| Treg_2_peak_47128 | 11.5347 | 20.0727 | 16.6033 | 399 | Distal Intergenic | 9516409 | 9530176 | Il7r |
| Treg_2_peak_47129 | 3.69374 | 5.73445 | 3.33556 | 109 | Downstream (2-3kb) | 9516409 | 9530176 | Il7r |
| Treg_2_peak_47130 | 3.69374 | 5.73445 | 3.33556 | 114 | Intron (ENSMUST000001 | 9516409 | 9530176 | Il7r |
| Treg_2_peak_47125 | 3.6359 | 5.41249 | 3.16527 | 112 | Intron (ENSMUST000002 | 9511898 | 9529766 | Il7r |
| Treg_2_peak_35751 | 3.6359 | 5.41249 | 3.16527 | 96 | Distal Intergenic | 56479277 | 56482246 | Il9 |
| Treg_2_peak_35752 | 3.69374 | 5.73445 | 3.33556 | 110 | Distal Intergenic | 56479277 | 56482246 | Il9 |
| Treg_2_peak_52788 | 4.99882 | 7.12189 | 4.62813 | 123 | Distal Intergenic | 10782240 | 10785536 | Socs1 |
| Treg_2_peak_17404 | 4.54487 | 7.11231 | 4.62813 | 108 | Downstream (<1kb) | 95387667 | 95392967 | Socs2 |
| Treg_2_peak_17406 | 8.76471 | 15.1251 | 11.9416 | 197 | Intron (ENSMUST000001 | 95388354 | 95412734 | Socs2 |
| Treg_2_peak_68062 | 4.54487 | 7.11231 | 4.62813 | 146 | Intron (ENSMUST000001 | 88665224 | 88758491 | Socs6 |
| Treg_2_peak_68063 | 3.6359 | 5.41249 | 3.16527 | 110 | Intron (ENSMUST000001 | 88665224 | 88758491 | Socs6 |
| Treg_2_peak_68064 | 6.46404 | 11.3035 | 8.33659 | 203 | Intron (ENSMUST000001 | 88665224 | 88758491 | Socs6 |
| Treg_2_peak_68070 | 5.45384 | 8.8909 | 6.19058 | 110 | Intron (ENSMUST000001 | 88868495 | 88894352 | Socs6 |
| Treg_2_peak_68071 | 5.5406 | 9.37639 | 6.57036 | 130 | Intron (ENSMUST000001 | 88868495 | 88894352 | Socs6 |
| Treg_2_peak_68056 | 4.61717 | 7.516 | 4.89772 | 155 | Intron (ENSMUST000001 | 88665224 | 88758491 | Socs6 |
| Treg_2_peak_68057 | 3.69374 | 5.73445 | 3.33556 | 134 | Intron (ENSMUST000001 | 88665224 | 88758491 | Socs6 |
| Treg_2_peak_68058 | 4.54487 | 7.11231 | 4.62813 | 154 | Intron (ENSMUST000001 | 88665224 | 88758491 | Socs6 |
| Treg_2_peak_68059 | 7.38747 | 13.2885 | 10.182 | 140 | Intron (ENSMUST000001 | 88665224 | 88758491 | Socs6 |
| Treg_2_peak_68060 | 5.45384 | 8.8909 | 6.19058 | 124 | Intron (ENSMUST000001 | 88665224 | 88758491 | Socs6 |
| Treg_2_peak_68061 | 6.36282 | 10.7362 | 7.85556 | 148 | Intron (ENSMUST000001 | 88665224 | 88758491 | Socs6 |
| Treg_2_peak_68069 | 6.42598 | 9.29198 | 6.57036 | 118 | Promoter (2-3kb) | 88870595 | 88887337 | Socs6 |

| Table S9: Narrow peaks ChIPseeker annotation of HDAC8 in Treg cells (3) | | | | | | | | |
|---|---|---|---|---|---|---|---|---|
| name | signalValue | '-log10pvalue | '-log10qvalue | peak_summit | annotation | geneStart | geneEnd | geneName |
| Treg_3_peak_8968 | 12.3944 | 22.362 | 18.1596 | 161 | Distal Intergenic | 82176657 | 82305690 | Ccl1 |
| Treg_3_peak_8960 | 5.40459 | 7.41211 | 4.32469 | 130 | Distal Intergenic | 82176657 | 82179812 | Ccl1 |
| Treg_3_peak_8961 | 4.19571 | 5.73537 | 2.89041 | 118 | Distal Intergenic | 82176657 | 82179812 | Ccl1 |
| Treg_3_peak_8966 | 4.15656 | 5.14039 | 2.50004 | 257 | Intron (ENSMUST0000013 | 82176657 | 82305690 | Ccl1 |
| Treg_3_peak_8965 | 9.23057 | 15.5688 | 11.7215 | 218 | Intron (ENSMUST0000013 | 82176659 | 82179812 | Ccl1 |
| Treg_3_peak_8962 | 5.72948 | 8.36552 | 5.18008 | 129 | Intron (ENSMUST0000013 | 82176659 | 82179812 | Ccl1 |
| Treg_3_peak_8963 | 5.72948 | 8.36552 | 5.18008 | 58 | Intron (ENSMUST0000013 | 82176659 | 82179812 | Ccl1 |
| Treg_3_peak_8964 | 3.35657 | 4.31618 | 1.74912 | 159 | Intron (ENSMUST0000013 | 82176659 | 82179812 | Ccl1 |
| Treg_3_peak_8967 | 4.75144 | 6.43742 | 3.52783 | 114 | Promoter (1-2kb) | 82176657 | 82305690 | Ccl1 |
| Treg_3_peak_8957 | 6.84239 | 10.194 | 6.80657 | 292 | Distal Intergenic | 82057823 | 82062955 | Ccl11 |
| Treg_3_peak_48919 | 13.4624 | 24.5259 | 20.2379 | 150 | Distal Intergenic | 94810453 | 94812036 | Ccl17 |
| Treg_3_peak_1649 | 7.36648 | 11.5336 | 8.01027 | 142 | Distal Intergenic | 83116793 | 83119167 | Ccl20 |
| Treg_3_peak_1648 | 4.09249 | 5.4299 | 2.7156 | 175 | Distal Intergenic | 83116766 | 83119166 | Ccl20 |
| Treg_3_peak_48915 | 4.19571 | 5.73537 | 2.89041 | 80 | Distal Intergenic | 94745590 | 94751699 | Ccl22 |
| Treg_3_peak_48916 | 3.60306 | 4.16554 | 1.74912 | 321 | Distal Intergenic | 94745590 | 94751699 | Ccl22 |
| Treg_3_peak_48917 | 3.24875 | 4.00398 | 1.68449 | 113 | Distal Intergenic | 94745590 | 94751699 | Ccl22 |
| Treg_3_peak_48918 | 4.91099 | 6.86453 | 3.89362 | 113 | Intron (ENSMUST0000003 | 94745680 | 94749873 | Ccl22 |
| Treg_3_peak_40213 | 5.874 | 8.79695 | 5.52216 | 146 | Distal Intergenic | 135570580 | 135573049 | Ccl24 |
| Treg_3_peak_40212 | 3.4638 | 4.00435 | 1.68449 | 206 | Distal Intergenic | 135570580 | 135573049 | Ccl24 |
| Treg_3_peak_40211 | 5.32186 | 7.33064 | 4.25169 | 106 | Downstream (<1kb) | 135569937 | 135573049 | Ccl24 |
| Treg_3_peak_46947 | 10.6405 | 18.3874 | 14.3776 | 268 | Promoter (1-2kb) | 4332259 | 4334807 | Ccl25 |
| Treg_3_peak_8995 | 16.37 | 31.5605 | 27.0276 | 170 | Intron (ENSMUST0000012 | 83587882 | 83593087 | Ccl6 |
| Treg_3_peak_8958 | 7.12716 | 10.8861 | 7.41243 | 119 | Distal Intergenic | 82115185 | 82116799 | Ccl8 |
| Treg_3_peak_8959 | 5.03486 | 7.23294 | 4.1628 | 136 | Distal Intergenic | 82115185 | 82116799 | Ccl8 |
| Treg_3_peak_8994 | 4.38634 | 5.59955 | 2.86402 | 202 | Distal Intergenic | 83575318 | 83577142 | Ccl9 |
| Treg_3_peak_52720 | 4.19571 | 5.73537 | 2.89041 | 90 | Promoter (<=1kb) | 123962124 | 123968692 | Ccr1 |
| Treg_3_peak_52721 | 12.4697 | 22.3446 | 18.1451 | 147 | Distal Intergenic | 123977243 | 123978408 | Ccr1l1 |
| Treg_3_peak_52723 | 4.19571 | 5.73537 | 2.89041 | 155 | Promoter (<=1kb) | 124101950 | 124113557 | Ccr2 |
| Treg_3_peak_52722 | 5.72948 | 8.36552 | 5.18008 | 122 | Distal Intergenic | 124021972 | 124031689 | Ccr3 |
| Treg_3_peak_52724 | 6.23484 | 8.91071 | 5.61864 | 202 | Distal Intergenic | 124128748 | 124147699 | Ccr5 |
| Treg_3_peak_21824 | 5.874 | 8.79695 | 5.52216 | 152 | Promoter (<=1kb) | 8236043 | 8256108 | Ccr6 |
| Treg_3_peak_21825 | 3.91071 | 4.95521 | 2.35137 | 109 | Promoter (<=1kb) | 8245065 | 8257120 | Ccr6 |
| Treg_3_peak_21826 | 6.13188 | 8.71216 | 5.50146 | 159 | Promoter (2-3kb) | 8245065 | 8257120 | Ccr6 |
| Treg_3_peak_9337 | 4.89402 | 6.8168 | 3.89225 | 110 | Distal Intergenic | 99144196 | 99155077 | Ccr7 |
| Treg_3_peak_52610 | 7.04128 | 10.7895 | 7.32935 | 122 | Distal Intergenic | 120092114 | 120094906 | Ccr8 |
| Treg_3_peak_3453 | 4.91099 | 6.86453 | 3.89362 | 122 | Distal Intergenic | 161780689 | 161788495 | Fasl |
| Treg_3_peak_6921 | 4.13148 | 5.54171 | 2.81239 | 162 | Distal Intergenic | 118441046 | 118445892 | Ifng |
| Treg_3_peak_6929 | 7.31056 | 11.0477 | 7.56841 | 233 | Distal Intergenic | 118502035 | 118556525 | Ifngas1 |
| Treg_3_peak_6933 | 5.874 | 8.79695 | 5.52216 | 135 | Distal Intergenic | 118502035 | 118556525 | Ifngas1 |
| Treg_3_peak_6928 | 4.91099 | 6.86453 | 3.89362 | 113 | Distal Intergenic | 118502035 | 118556525 | Ifngas1 |
| Treg_3_peak_6930 | 3.65528 | 4.38317 | 1.80968 | 103 | Distal Intergenic | 118502035 | 118556525 | Ifngas1 |
| Treg_3_peak_6927 | 3.27399 | 4.07351 | 1.6853 | 194 | Distal Intergenic | 118502035 | 118556525 | Ifngas1 |
| Treg_3_peak_6932 | 3.27399 | 4.07351 | 1.6853 | 117 | Distal Intergenic | 118502035 | 118556525 | Ifngas1 |
| Treg_3_peak_6931 | 3.4638 | 4.00435 | 1.68449 | 127 | Distal Intergenic | 118502035 | 118556525 | Ifngas1 |
| Treg_3_peak_6926 | 3.4638 | 4.00435 | 1.68449 | 114 | Intron (ENSMUST0000023 | 118502035 | 118555352 | Ifngas1 |
| Treg_3_peak_6925 | 9.1521 | 14.8555 | 11.0764 | 184 | Intron (ENSMUST0000023 | 118502035 | 118555352 | Ifngas1 |
| Treg_3_peak_6924 | 4.91099 | 6.86453 | 3.89362 | 168 | Intron (ENSMUST0000023 | 118502035 | 118555352 | Ifngas1 |
| Treg_3_peak_4673 | 5.03486 | 7.23294 | 4.1628 | 97 | Distal Intergenic | 19605919 | 19609256 | Ifngr1 |
| Treg_3_peak_21488 | 4.98017 | 7.17466 | 4.1628 | 246 | Intron (ENSMUST0000002 | 91547147 | 91560649 | Ifngr2 |
| Treg_3_peak_35085 | 5.67657 | 9.2058 | 5.90597 | 120 | Promoter (<=1kb) | 41767799 | 41769474 | Il11ra1 |
| Treg_3_peak_32251 | 20.9761 | 44.5818 | 39.7466 | 194 | Distal Intergenic | 68694021 | 68698547 | Il12a |
| Treg_3_peak_32250 | 7.43667 | 11.7378 | 8.18836 | 136 | Distal Intergenic | 68694021 | 68698547 | Il12a |
| Treg_3_peak_32249 | 12.9398 | 23.4361 | 19.1964 | 215 | Promoter (2-3kb) | 68690644 | 68698547 | Il12a |
| Treg_3_peak_8041 | 4.79132 | 6.5399 | 3.62572 | 110 | Distal Intergenic | 44400063 | 44414010 | Il12b |
| Treg_3_peak_42009 | 3.70959 | 4.40186 | 1.82607 | 97 | Intron (ENSMUST0000001 | 67292018 | 67376188 | Il12rb2 |
| Treg_3_peak_42007 | 3.4638 | 4.00435 | 1.68449 | 127 | Intron (ENSMUST0000011 | 67291318 | 67339694 | Il12rb2 |
| Treg_3_peak_42010 | 7.65907 | 11.738 | 8.18836 | 132 | Promoter (2-3kb) | 67292018 | 67376188 | Il12rb2 |
| Treg_3_peak_42008 | 4.19571 | 5.73537 | 2.89041 | 223 | Promoter (2-3kb) | 67291318 | 67339694 | Il12rb2 |
| Treg_3_peak_8256 | 6.4056 | 11.124 | 7.63862 | 157 | Downstream (1-2kb) | 53631324 | 53634702 | Il13 |
| Treg_3_peak_53055 | 4.09249 | 5.4299 | 2.7156 | 98 | Distal Intergenic | 36112110 | 36171259 | Il13ra1 |
| Treg_3_peak_53846 | 4.19571 | 5.73537 | 2.89041 | 132 | Distal Intergenic | 147383478 | 147403832 | Il13ra2 |
| Treg_3_peak_48619 | 4.19571 | 5.73537 | 2.89041 | 159 | Distal Intergenic | 82331914 | 82344473 | Il15 |
| Treg_3_peak_48621 | 5.72948 | 8.36552 | 5.18008 | 148 | Intron (ENSMUST0000020 | 82331739 | 82345683 | Il15 |
| Treg_3_peak_48620 | 5.72948 | 8.36552 | 5.18008 | 117 | Promoter (<=1kb) | 82331739 | 82345683 | Il15 |
| Treg_3_peak_27130 | 8.26296 | 13.416 | 9.72605 | 141 | Intron (ENSMUST0000013 | 11723164 | 11733968 | Il15ra |
| Treg_3_peak_382 | 6.0051 | 8.59188 | 5.38576 | 148 | Distal Intergenic | 20730905 | 20734496 | Il17a |
| Treg_3_peak_381 | 4.76947 | 6.37369 | 3.47838 | 140 | Distal Intergenic | 20730905 | 20734496 | Il17a |
| Treg_3_peak_16249 | 10.1833 | 17.1861 | 13.2553 | 182 | Distal Intergenic | 57524777 | 57543166 | Il17d |
| Treg_3_peak_15623 | 15.1046 | 28.7983 | 24.3496 | 141 | Intron (ENSMUST0000003 | 27067397 | 27100976 | Il17rd |
| Treg_3_peak_15625 | 8.41695 | 13.3016 | 9.62041 | 161 | Intron (ENSMUST0000003 | 27067397 | 27100976 | Il17rd |
| Treg_3_peak_15624 | 4.15656 | 5.14039 | 2.50004 | 106 | Intron (ENSMUST0000003 | 27067397 | 27100976 | Il17rd |
| Treg_3_peak_15626 | 8.85717 | 14.2727 | 10.5215 | 117 | Promoter (1-2kb) | 27067397 | 27100976 | Il17rd |
| Treg_3_peak_50974 | 9.60816 | 16.4245 | 12.5385 | 176 | Distal Intergenic | 50575273 | 50581837 | Il18 |
| Treg_3_peak_2859 | 7.36648 | 11.5336 | 8.01027 | 131 | Distal Intergenic | 130932816 | 130940115 | Il19 |
| Treg_3_peak_2860 | 4.15656 | 5.14039 | 2.50004 | 141 | Distal Intergenic | 130932816 | 130940115 | Il19 |
| Treg_3_peak_27427 | 5.03486 | 7.23294 | 4.1628 | 131 | Exon (ENSMUST0000005 | 24186476 | 24193568 | Il1f9 |
| Treg_3_peak_772 | 4.91099 | 6.86453 | 3.89362 | 142 | Distal Intergenic | 40225080 | 40317257 | Il1r1 |
| Treg_3_peak_773 | 4.19571 | 5.73537 | 2.89041 | 118 | Distal Intergenic | 40225080 | 40317257 | Il1r1 |
| Treg_3_peak_775 | 6.54798 | 9.9241 | 6.55583 | 186 | Intron (ENSMUST0000002 | 40266657 | 40277178 | Il1r1 |
| Treg_3_peak_776 | 6.66829 | 10.2794 | 6.87692 | 173 | Intron (ENSMUST0000002 | 40266657 | 40277178 | Il1r1 |
| Treg_3_peak_770 | 3.43117 | 3.94307 | 1.64231 | 139 | Distal Intergenic | 40074079 | 40112136 | Il1r2 |
| Treg_3_peak_20231 | 6.86547 | 11.879 | 8.326 | 135 | Distal Intergenic | 26581704 | 26725264 | Il1rap |
| Treg_3_peak_20230 | 4.09249 | 5.4299 | 2.7156 | 115 | Distal Intergenic | 26581704 | 26725264 | Il1rap |
| Treg_3_peak_20232 | 8.31312 | 13.0952 | 9.44846 | 168 | Intron (ENSMUST0000009 | 26624156 | 26715068 | Il1rap |
| Treg_3_peak_20233 | 5.78408 | 8.52349 | 5.32538 | 164 | Intron (ENSMUST0000009 | 26722434 | 26730117 | Il1rap |
| Treg_3_peak_53408 | 10.0697 | 17.3659 | 13.4112 | 134 | Intron (ENSMUST0000011 | 86747242 | 87890235 | Il1rapl1 |
| Treg_3_peak_53407 | 8.39143 | 13.8094 | 10.0778 | 141 | Intron (ENSMUST0000011 | 86747242 | 87890235 | Il1rapl1 |

| | | | | | | | | | | |
|---|---|---|---|---|---|---|---|---|---|---|
| Treg_3_peak_53766 | 4.19571 | 5.73537 | 2.89041 | 114 | Distal Intergenic | 137570608 | 138846946 | Il1rapl2 |
| Treg_3_peak_31622 | 12.0288 | 21.3264 | 17.1697 | 402 | Distal Intergenic | 37120523 | 37125959 | Il2 |
| Treg_3_peak_31621 | 7.55229 | 12.0912 | 8.49257 | 134 | Promoter (<=1kb) | 37120523 | 37125959 | Il2 |
| Treg_3_peak_2858 | 4.75144 | 6.43742 | 3.52783 | 147 | Distal Intergenic | 130906985 | 130911451 | Il20 |
| Treg_3_peak_4678 | 4.19571 | 5.73537 | 2.89041 | 154 | Distal Intergenic | 19712570 | 19760053 | Il20ra |
| Treg_3_peak_4679 | 5.2804 | 7.10905 | 4.11776 | 111 | Promoter (1-2kb) | 19712634 | 19755824 | Il20ra |
| Treg_3_peak_52084 | 6.9276 | 10.2656 | 6.86394 | 133 | Promoter (2-3kb) | 100458530 | 100461735 | Il20rb |
| Treg_3_peak_36999 | 9.32579 | 19.199 | 15.1372 | 194 | Distal Intergenic | 135728172 | 135752140 | Il22ra1 |
| Treg_3_peak_37000 | 13.6848 | 24.9583 | 20.6477 | 220 | Intron (ENSMUST0000010 | 135728172 | 135752140 | Il22ra1 |
| Treg_3_peak_4674 | 5.03486 | 7.23294 | 4.1628 | 180 | Intron (ENSMUST0000003 | 19621998 | 19634681 | Il22ra2 |
| Treg_3_peak_4675 | 4.75144 | 6.43742 | 3.52783 | 109 | Intron (ENSMUST0000003 | 19621998 | 19634681 | Il22ra2 |
| Treg_3_peak_42014 | 12.2775 | 22.0188 | 17.8354 | 150 | Intron (ENSMUST0000011 | 67422932 | 67491855 | Il23r |
| Treg_3_peak_27129 | 8.40714 | 13.6879 | 9.98886 | 136 | Promoter (2-3kb) | 11642807 | 11693193 | Il2ra |
| Treg_3_peak_19107 | 5.82573 | 8.5202 | 5.32538 | 120 | Intron (ENSMUST0000008 | 78481324 | 78493756 | Il2rb |
| Treg_3_peak_8279 | 5.72948 | 8.36552 | 5.18008 | 112 | Distal Intergenic | 54265303 | 54267277 | Il3 |
| Treg_3_peak_8278 | 4.95778 | 6.9994 | 4.0125 | 56 | Distal Intergenic | 54265303 | 54267277 | Il3 |
| Treg_3_peak_39917 | 6.00438 | 9.0871 | 5.79161 | 146 | Intron (ENSMUST0000019 | 123480401 | 123489489 | Il31 |
| Treg_3_peak_15009 | 3.70959 | 4.40186 | 1.82607 | 245 | Intron (ENSMUST0000022 | 112525846 | 112532344 | Il31ra |
| Treg_3_peak_15010 | 4.19571 | 5.73537 | 2.89041 | 219 | Promoter (1-2kb) | 112546319 | 112565666 | Il31ra |
| Treg_3_peak_15011 | 5.03486 | 7.23294 | 4.1628 | 113 | Promoter (2-3kb) | 112522795 | 112580662 | Il31ra |
| Treg_3_peak_26181 | 5.03486 | 7.23294 | 4.1628 | 172 | Distal Intergenic | 29951816 | 29952813 | Il33 |
| Treg_3_peak_26180 | 3.35657 | 4.31618 | 1.74912 | 116 | Promoter (<=1kb) | 29951816 | 29952813 | Il33 |
| Treg_3_peak_8255 | 6.38717 | 10.0147 | 6.64294 | 184 | Distal Intergenic | 53612460 | 53618669 | Il4 |
| Treg_3_peak_8254 | 5.43291 | 8.17514 | 5.03677 | 135 | Distal Intergenic | 53612460 | 53618669 | Il4 |
| Treg_3_peak_8258 | 5.00122 | 7.12916 | 4.13687 | 111 | Distal Intergenic | 53720794 | 53725106 | Il5 |
| Treg_3_peak_42920 | 5.03486 | 7.23294 | 4.1628 | 189 | Intron (ENSMUST0000020 | 106711975 | 106745109 | Il5ra |
| Treg_3_peak_32703 | 7.43667 | 11.7378 | 8.18836 | 387 | Distal Intergenic | 89864059 | 89913196 | Il6ra |
| Treg_3_peak_15008 | 5.8383 | 9.46841 | 6.15325 | 122 | Distal Intergenic | 112464070 | 112510086 | Il6st |
| Treg_3_peak_30957 | 7.36648 | 11.5336 | 8.01027 | 128 | Distal Intergenic | 7573182 | 7613760 | Il7 |
| Treg_3_peak_30958 | 7.01413 | 10.4366 | 6.98562 | 155 | Distal Intergenic | 7573182 | 7613760 | Il7 |
| Treg_3_peak_30956 | 5.03486 | 7.23294 | 4.1628 | 148 | Intron (ENSMUST0000018 | 7573182 | 7613760 | Il7 |
| Treg_3_peak_17745 | 4.84932 | 6.34217 | 3.44919 | 187 | Distal Intergenic | 9516409 | 9530176 | Il7r |
| Treg_3_peak_6410 | 5.874 | 8.79695 | 5.52216 | 153 | Distal Intergenic | 95387667 | 95392967 | Socs2 |
| Treg_3_peak_6411 | 5.72948 | 8.36552 | 5.18008 | 172 | Distal Intergenic | 95387667 | 95392967 | Socs2 |
| Treg_3_peak_6412 | 4.07835 | 5.39034 | 2.7156 | 116 | Intron (ENSMUST0000013 | 95388354 | 95412734 | Socs2 |
| Treg_3_peak_25647 | 7.36648 | 11.5336 | 8.01027 | 113 | Intron (ENSMUST0000015 | 88665224 | 88758491 | Socs6 |
| Treg_3_peak_25648 | 4.09249 | 5.4299 | 2.7156 | 141 | Promoter (1-2kb) | 88665224 | 88758491 | Socs6 |

| Table S10: Mass spectrometry analysis of HDAC8 in effector T cells | | | | | | | |
|---|---|---|---|---|---|---|---|
| **Gene** | **Uniprot** | **logFC** | **AveExpr** | **t** | **P.Value** | **adj.P.Val** | **B** |
| Hdac8 | Q8VH37 | 8.85192778 | 0.49991862 | 22.8304362 | 7.69E-09 | 8.73E-06 | 10.4619104 |
| Tbc1d32 | Q3URV1 | -8.7887671 | 0.47925633 | -22.528179 | 8.59E-09 | 8.73E-06 | 10.3820991 |
| Grxcr2 | Q3TYR5 | -7.480524 | 0.22449481 | -18.638069 | 4.09E-08 | 2.77E-05 | 9.17547566 |
| Hipk2 | Q9QZR5 | 7.27246846 | -0.6960613 | 16.5362087 | 1.09E-07 | 3.38E-05 | 8.3529301 |
| Mlycd | Q99J39 | 5.78719058 | -1.1044241 | 16.1798072 | 1.30E-07 | 3.38E-05 | 8.19880753 |
| Rnf213 | E9Q555 | 5.72705883 | 2.4891938 | 16.8026455 | 9.54E-08 | 3.38E-05 | 8.46519086 |
| Cep43 | Q66JX5 | 5.56778692 | -1.0299836 | 15.6960001 | 1.66E-07 | 3.38E-05 | 7.98204543 |
| Myo1h | Q9D6A1 | -4.4421816 | -1.4370263 | -15.85433 | 1.53E-07 | 3.38E-05 | 8.05396313 |
| Pak6 | Q3ULB5 | -4.5417699 | -1.6416173 | -16.134756 | 1.33E-07 | 3.38E-05 | 8.17899542 |
| Trpm8 | Q8R4D5 | -7.7135505 | 0.40396411 | -15.95944 | 1.45E-07 | 3.38E-05 | 8.10117601 |
| Ikzf5 | Q8BU00 | 6.99683818 | 0.39500405 | 15.2312807 | 2.12E-07 | 3.92E-05 | 7.76524823 |
| Atad3 | Q925I1 | 6.02245498 | 0.9955935 | 14.8991519 | 2.54E-07 | 4.10E-05 | 7.60489595 |
| Ppip5k1 | A2ARP1 | 5.60313141 | -1.025129 | 14.8407919 | 2.62E-07 | 4.10E-05 | 7.57623836 |
| Nfat5 | Q9WV30-2 | 7.2710007 | -0.0687546 | 13.703435 | 4.99E-07 | 5.64E-05 | 6.98724543 |
| Fhod1 | Q6P9Q4 | 4.56093041 | -1.6673361 | 13.929649 | 4.37E-07 | 5.64E-05 | 7.10919469 |
| Mbd3 | Q9Z2D8 | 3.86672276 | -1.4774475 | 13.7529365 | 4.85E-07 | 5.64E-05 | 7.01414388 |
| Hip1r | Q9JKY5 | -3.7548227 | -2.0070911 | -14.038259 | 4.11E-07 | 5.64E-05 | 7.16687081 |
| Mccc2 | Q3ULD5 | -4.2047622 | -0.4415687 | -13.773048 | 4.79E-07 | 5.64E-05 | 7.0250377 |
| Fbxl20 | Q9CZV8 | 5.38987092 | -1.6650425 | 13.089133 | 7.21E-07 | 7.72E-05 | 6.64317754 |
| Sptan1 | P16546 | 3.89852251 | 1.36019298 | 13.0018786 | 7.61E-07 | 7.74E-05 | 6.59271743 |
| Msmo1 | Q9CRA4 | 6.94721682 | -0.301253 | 12.9063107 | 8.07E-07 | 7.82E-05 | 6.53698078 |
| Stambpl1 | Q76N33 | 4.17898307 | -1.9183055 | 12.798661 | 8.63E-07 | 7.94E-05 | 6.47360363 |
| Stom | P54116 | -4.3401405 | -1.4097818 | -12.736399 | 8.98E-07 | 7.94E-05 | 6.43665743 |
| P18529 | NA | -7.1183992 | -0.1554083 | -12.667195 | 9.38E-07 | 7.95E-05 | 6.39533906 |
| Stag3 | O70576 | -3.4291087 | -1.9197895 | -12.365266 | 1.14E-06 | 9.25E-05 | 6.2119057 |
| Znf451 | Q8C0P7 | 5.58550578 | -1.4350307 | 11.7998781 | 1.65E-06 | 1.29E-04 | 5.85397917 |
| Sptbn1 | Q62261 | 4.27836658 | -0.060564 | 11.7007789 | 1.77E-06 | 1.33E-04 | 5.78922208 |
| Mau2 | Q9D2X5-2 | 3.76664663 | -1.6812172 | 11.3090726 | 2.31E-06 | 1.62E-04 | 5.52707732 |
| Spata18 | Q0P557 | -5.304239 | -0.8662537 | -11.315437 | 2.30E-06 | 1.62E-04 | 5.53141722 |
| Asf1a | Q9CQE6 | 5.61247442 | -1.1011746 | 11.1485382 | 2.59E-06 | 1.76E-04 | 5.4167066 |
| Zzef1 | Q5SSH7 | 4.22831977 | -1.4088872 | 10.9515793 | 2.98E-06 | 1.96E-04 | 5.27888221 |
| Otulinl | Q3TVP5 | 3.65977568 | -2.3781672 | 10.8782433 | 3.14E-06 | 1.99E-04 | 5.22687222 |
| P01652 | NA | -4.8251136 | 3.51074918 | -10.839316 | 3.23E-06 | 1.99E-04 | 5.1991103 |
| Il2rb | P16297 | 5.46350051 | -1.5670535 | 10.3731238 | 4.57E-06 | 2.51E-04 | 4.85806457 |
| Sun1 | Q9D666 | 4.60480818 | -1.9482857 | 10.4672432 | 4.25E-06 | 2.51E-04 | 4.92821674 |
| Rere | Q80TZ9 | 4.50380652 | -1.5096966 | 10.4010207 | 4.47E-06 | 2.51E-04 | 4.87892755 |
| Acot8 | P58137 | -3.5953089 | -2.2017977 | -10.374309 | 4.56E-06 | 2.51E-04 | 4.85895229 |
| Lrrcc1 | Q69ZB0 | 4.63910829 | -2.0930101 | 10.3027886 | 4.82E-06 | 2.58E-04 | 4.80520025 |
| Crtc3 | Q91X84 | 7.9311297 | 0.80157509 | 10.1998388 | 5.21E-06 | 2.67E-04 | 4.72713568 |
| Ipo9 | Q91YE6 | 5.32953092 | -1.452841 | 10.1899966 | 5.25E-06 | 2.67E-04 | 4.71962943 |
| Fuom | Q8R2K1 | -3.1471251 | -1.8646986 | -10.052349 | 5.84E-06 | 2.90E-04 | 4.61385374 |
| Top2b | Q64511 | 4.40246116 | -2.0154562 | 9.99685407 | 6.09E-06 | 2.95E-04 | 4.57078302 |
| Hectd3 | Q3U487 | 4.22141274 | -1.4739642 | 9.95844574 | 6.28E-06 | 2.97E-04 | 4.54082896 |
| Fhl3 | Q9R059 | 4.28916195 | -2.0299047 | 9.83598558 | 6.91E-06 | 3.20E-04 | 4.4445257 |
| Znf639 | Q99KZ6 | 7.72771112 | 0.12506901 | 9.65192075 | 8.01E-06 | 3.56E-04 | 4.2974513 |
| Rfc3 | Q8R323 | 4.17284268 | -1.5341965 | 9.64541531 | 8.05E-06 | 3.56E-04 | 4.29220136 |
| Pgm3 | Q9CYR6 | 4.15508683 | -2.4059937 | 9.60499491 | 8.31E-06 | 3.60E-04 | 4.25950158 |
| Rad52 | P43352 | 4.26914185 | -1.1935502 | 9.52299188 | 8.88E-06 | 3.76E-04 | 4.1927345 |
| Exoc7 | O35250 | 2.9803268 | -2.3784448 | 9.45302313 | 9.41E-06 | 3.90E-04 | 4.13530899 |
| Arhgef2 | Q60875 | 5.29728082 | 0.00580801 | 9.38529152 | 9.94E-06 | 4.05E-04 | 4.07931464 |
| Ppp1ca | P62137 | 2.67670121 | 2.24974429 | 9.01656228 | 1.35E-05 | 5.40E-04 | 3.76732095 |
| Ttc39c | Q8VE09 | -3.329359 | -2.7718211 | -8.9285308 | 1.46E-05 | 5.71E-04 | 3.69099898 |
| Phf20l1 | Q8CCJ9 | 4.72321353 | -1.6205695 | 8.75691879 | 1.69E-05 | 6.50E-04 | 3.54011647 |
| Dcun1d1 | Q9QZ73 | 3.0722771 | -2.614304 | 8.69950547 | 1.78E-05 | 6.70E-04 | 3.48900958 |
| Wdr18 | Q4VBE8 | 2.77196926 | -1.6475494 | 8.54602899 | 2.04E-05 | 7.40E-04 | 3.35081455 |
| Paxbp1 | P58501 | 2.53487993 | -3.0517119 | 8.56112242 | 2.01E-05 | 7.40E-04 | 3.36450788 |
| Rfc2 | Q9WUK4 | 6.98750601 | 0.36145969 | 8.38755322 | 2.35E-05 | 8.11E-04 | 3.20566539 |

| | | | | | | | |
|---|---|---|---|---|---|---|---|
| Kansl2 | Q8BQR4 | 3.94352837 | -2.1368023 | 8.41231556 | 2.30E-05 | 8.11E-04 | 3.228512 |
| Dytn | A2CI98 | -3.5631469 | -2.3333076 | -8.3856026 | 2.35E-05 | 8.11E-04 | 3.20386307 |
| Adnp | Q9Z103 | 4.86611299 | -1.803663 | 8.24477293 | 2.67E-05 | 9.07E-04 | 3.0727097 |
| Nipbl | Q6KCD5 | 3.45600317 | -1.9553394 | 8.00075178 | 3.35E-05 | 0.00111794 | 2.84056086 |
| Eif2b5 | Q8CHW4 | 3.34185881 | -2.8359791 | 7.97600636 | 3.43E-05 | 0.00112576 | 2.81666575 |
| Rfc5 | Q9D0F6 | 4.45488723 | -1.7327107 | 7.8968934 | 3.70E-05 | 0.00115717 | 2.73982693 |
| Pdhx | Q8BKZ9 | -2.211096 | -0.4379974 | -7.9146692 | 3.64E-05 | 0.00115717 | 2.75715093 |
| Tsc22d4 | Q9EQN3 | -2.771583 | -1.76619 | -7.8967584 | 3.70E-05 | 0.00115717 | 2.73969527 |
| Trpv2 | Q9WTR1 | 3.83260587 | -2.1641477 | 7.65559941 | 4.66E-05 | 0.0014361 | 2.50121682 |
| Nbn | Q9R207 | 2.39066832 | -3.0827278 | 7.57391012 | 5.05E-05 | 0.00153189 | 2.41895641 |
| Eif2b4 | Q61749 | 4.90804079 | -1.3695495 | 7.53714853 | 5.23E-05 | 0.00155342 | 2.38168932 |
| Znf609 | Q8BZ47 | 3.13559003 | -2.5069696 | 7.52969329 | 5.27E-05 | 0.00155342 | 2.37411265 |
| Gstt3 | Q99L20 | 5.00901017 | -0.5243421 | 7.49141517 | 5.47E-05 | 0.00159006 | 2.3351101 |
| Herc4 | Q6PAV2 | 3.7123998 | -1.3945869 | 7.44409904 | 5.73E-05 | 0.00161997 | 2.28666404 |
| Dlat | Q8BMF4 | -2.5341084 | 1.96371577 | -7.4552698 | 5.67E-05 | 0.00161997 | 2.29812504 |
| Ablim1 | Q8K4G5 | 2.94043965 | 0.35255523 | 7.41334558 | 5.91E-05 | 0.00164734 | 2.25503639 |
| Plbd1 | Q8VCI0 | -2.9531637 | -2.1212998 | -7.1362629 | 7.82E-05 | 0.00214941 | 1.96502733 |
| Mcrs1 | Q99L90 | 3.52189247 | -2.636775 | 7.07311747 | 8.34E-05 | 0.00226101 | 1.89764175 |
| Adh5 | P28474 | 2.12560977 | 2.55135996 | 7.06107482 | 8.45E-05 | 0.00226101 | 1.884735 |
| Lgals3bp | Q07797 | -1.6081203 | -0.9925133 | -6.8911421 | 1.01E-04 | 0.00266362 | 1.70069386 |
| Rfc1 | P35601 | 3.04390342 | -2.2095272 | 6.85895377 | 1.04E-04 | 0.00272008 | 1.6654263 |
| Mrgbp | Q9DAT2 | 1.99622206 | 1.11766176 | 6.83027502 | 1.08E-04 | 0.00276821 | 1.63389375 |
| Fastkd1 | Q6DI86 | 4.01295616 | -2.4202153 | 6.81787207 | 1.09E-04 | 0.00276888 | 1.6202243 |
| Hcfc1 | Q61191 | 2.10260919 | 0.209372 | 6.80643675 | 1.10E-04 | 0.00276888 | 1.60760396 |
| Cenpx | Q8C4X1 | 3.03658663 | -2.3167662 | 6.75419608 | 1.17E-04 | 0.00288763 | 1.54973762 |
| Trerf1 | Q8BXJ2 | -3.9124229 | -1.9786062 | -6.7440282 | 1.18E-04 | 0.00288763 | 1.53843418 |
| Ric8a | Q3TIR3 | 3.69325201 | -2.0853983 | 6.56359009 | 1.43E-04 | 0.00330817 | 1.33562176 |
| Lrpprc | Q6PB66 | 2.30158066 | -1.1960235 | 6.57562086 | 1.41E-04 | 0.00330817 | 1.34927632 |
| Celf2 | Q9Z0H4 | 1.61092572 | 0.33712672 | 6.57191 | 1.42E-04 | 0.00330817 | 1.34506663 |
| Dhx9 | O70133-2 | -1.7018068 | 1.67139041 | -6.5681448 | 1.42E-04 | 0.00330817 | 1.34079345 |
| Ddx20 | Q9JJY4 | -2.1949337 | -2.7964667 | -6.5712732 | 1.42E-04 | 0.00330817 | 1.34434408 |
| Syne3 | Q4FZC9 | 4.05322475 | -1.7110493 | 6.40417919 | 1.70E-04 | 0.00389648 | 1.15288941 |
| Clint1 | Q99KN9 | 4.32444925 | 1.96061751 | 6.38687209 | 1.74E-04 | 0.0039278 | 1.13284666 |
| Rad50 | P70388 | 2.59633866 | -0.0552446 | 6.36014625 | 1.79E-04 | 0.00398798 | 1.10181737 |
| Tardbp | Q921F2 | 2.47497416 | 3.80567449 | 6.35341348 | 1.80E-04 | 0.00398798 | 1.09398533 |
| Rev1 | Q920Q2 | 3.82259845 | -2.3701735 | 6.32470651 | 1.86E-04 | 0.00407342 | 1.06052268 |
| Btbd9 | Q8C726 | -2.875914 | -3.0737819 | -6.2505759 | 2.02E-04 | 0.00437941 | 0.973595 |
| Kat8 | Q9D1P2 | 1.90558782 | -2.8944791 | 6.17180064 | 2.21E-04 | 0.00473703 | 0.88039936 |
| Snx6 | Q6P8X1 | 2.34198945 | -0.5210728 | 6.14204253 | 2.29E-04 | 0.00484909 | 0.84497198 |
| Supt6h | Q62383 | 2.89308747 | -2.9970416 | 6.12063693 | 2.35E-04 | 0.00486755 | 0.81941279 |
| Smg1 | Q8BKX6 | -4.1984818 | -1.3674564 | -6.1289501 | 2.32E-04 | 0.00486755 | 0.82934654 |
| Pusl1 | A2ADA5 | 2.02888027 | -2.9182288 | 6.10999044 | 2.37E-04 | 0.00487736 | 0.80667683 |
| Eml3 | Q8VC03 | -2.5997629 | -2.2553181 | -6.0640493 | 2.50E-04 | 0.0050897 | 0.75153897 |
| Immt.1 | NA | -1.5099719 | 0.85231397 | -5.9584795 | 2.83E-04 | 0.00569339 | 0.62372003 |
| Fancc | P50652 | 3.08894028 | -2.7625273 | 5.92952976 | 2.92E-04 | 0.00583088 | 0.58839569 |
| Ubac2 | Q8R1K1 | 2.8427336 | -1.2155258 | 5.85280019 | 3.20E-04 | 0.00631734 | 0.49419752 |
| Stoml2 | Q99JB2 | -1.4425773 | -0.3904443 | -5.7786145 | 3.49E-04 | 0.00682964 | 0.40232646 |
| Ppp2r1a | Q76MZ3 | 1.32550765 | 1.1219516 | 5.72311418 | 3.73E-04 | 0.0072264 | 0.33308049 |
| Osbpl8 | B9EJ86 | 2.05626452 | -2.8884043 | 5.64877421 | 4.08E-04 | 0.00782528 | 0.23963437 |
| Rbm7 | Q9CQT2 | -1.5498966 | -1.5376811 | -5.6067107 | 4.29E-04 | 0.00815565 | 0.18640609 |
| Diaph1 | O08808 | 1.33212935 | -1.5742745 | 5.55481547 | 4.57E-04 | 0.00856108 | 0.12038267 |
| Tax1bp1 | Q3UKC1 | -2.1702226 | -3.1545518 | -5.5514421 | 4.59E-04 | 0.00856108 | 0.11607733 |
| Babam1 | Q3UI43 | -2.518933 | 0.11493715 | -5.4944978 | 4.92E-04 | 0.00909381 | 0.04315063 |
| Kansl1 | Q80TG1 | 2.9054946 | -1.9384516 | 5.48110212 | 5.00E-04 | 0.00916103 | 0.02592632 |
| Exosc5 | Q9CRA8 | 2.31576464 | -0.2156698 | 5.37933239 | 5.67E-04 | 0.01021334 | -0.1057896 |
| Mccc1 | Q99MR8 | -3.4517419 | -0.7230318 | -5.3784538 | 5.67E-04 | 0.01021334 | -0.1069333 |
| Golga3 | P55937 | -4.0241896 | -1.3051814 | -5.3608545 | 5.80E-04 | 0.0103474 | -0.129868 |
| Clta | O08585 | 3.95111957 | 3.3046576 | 5.34656735 | 5.90E-04 | 0.01044133 | -0.1485201 |
| Cltc | Q68FD5 | 3.63789992 | 3.59065047 | 5.24859887 | 6.67E-04 | 0.0116017 | -0.2772311 |

| | | | | | | | |
|---|---|---|---|---|---|---|---|
| Mrpl48 | Q8JZS9 | -1.8892536 | -1.5804177 | -5.2538308 | 6.63E-04 | 0.0116017 | -0.2703216 |
| Trmt1l | A2RSY6 | 1.98302996 | -3.475624 | 5.22540708 | 6.87E-04 | 0.01184452 | -0.3079084 |
| Exoc5 | Q3TPX4 | 2.463025 | -2.6537279 | 5.21184914 | 6.99E-04 | 0.01187238 | -0.3258793 |
| Pan2 | Q8BGF7 | 1.25429937 | -3.8741384 | 5.21023796 | 7.00E-04 | 0.01187238 | -0.3280167 |
| Med13 | Q5SWW4 | 2.01819843 | -3.3366165 | 5.10562966 | 8.00E-04 | 0.013449 | -0.4676165 |
| Cep350 | E9Q309 | 5.09603503 | -0.7186528 | 5.07733316 | 8.30E-04 | 0.01371858 | -0.5056576 |
| Sirt7 | Q8BKJ9 | -1.9108607 | -0.4686099 | -5.0783345 | 8.29E-04 | 0.01371858 | -0.5043094 |
| Hspa1l | P16627 | 2.04112097 | -0.2851052 | 5.05324651 | 8.56E-04 | 0.01403555 | -0.5381329 |
| Cltb | Q6IRU5-2 | 4.40761123 | 0.41727229 | 5.03404622 | 8.77E-04 | 0.0141585 | -0.564082 |
| Il2rg | P34902 | 2.77311303 | -3.0988062 | 5.03903154 | 8.71E-04 | 0.0141585 | -0.5573391 |
| Ehd1 | Q9WVK4 | 5.0625724 | -0.2377201 | 5.01878972 | 8.95E-04 | 0.01419713 | -0.5847402 |
| Pan3 | Q640Q5 | 2.66830166 | -2.6178866 | 5.02017536 | 8.93E-04 | 0.01419713 | -0.5828625 |
| Hdac1 | O09106 | 1.66800175 | 2.97680644 | 5.0136997 | 9.00E-04 | 0.01419713 | -0.59164 |
| Sbf1 | Q6ZPE2 | 2.90825091 | -1.0867155 | 4.98605659 | 9.33E-04 | 0.01460092 | -0.6291796 |
| Ndufaf6 | A2AIL4 | -2.0684515 | -2.6413733 | -4.9732874 | 9.49E-04 | 0.01473138 | -0.6465586 |
| Fcgr4 | A0A0B4J1G0 | -3.3542076 | -0.3240445 | -4.9037776 | 0.0010387 | 0.0160054 | -0.7415878 |
| Lrrfip1.1 | NA | 2.58779111 | -2.3245705 | 4.89379383 | 0.00105236 | 0.01609393 | -0.7552961 |
| Appl1 | Q8K3H0 | -1.7819409 | -2.6584407 | -4.867738 | 0.00108893 | 0.01652892 | -0.7911417 |
| Ccdc9 | Q8VC31 | 2.41530354 | -2.4780213 | 4.8525945 | 0.00111081 | 0.01673623 | -0.8120214 |
| Rif1 | Q6PR54 | 6.39847503 | 1.00767131 | 4.84301329 | 0.0011249 | 0.01680517 | -0.8252494 |
| Aldh16a1 | Q571I9 | -2.3334663 | -2.440404 | -4.838298 | 0.00113191 | 0.01680517 | -0.8317645 |
| Isg15 | Q64339 | 1.8522287 | 1.23927358 | 4.81564063 | 0.00116625 | 0.01718945 | -0.8631158 |
| Slfn5 | Q8CBA2 | 1.77348906 | -2.6845301 | 4.80997761 | 0.001175 | 0.01719392 | -0.8709637 |
| Chd4 | Q6PDQ2 | 2.14752361 | -1.1261599 | 4.79588177 | 0.00119711 | 0.01739229 | -0.8905186 |
| Ssh2 | Q5SW75 | 2.95300574 | -2.3771248 | 4.77898939 | 0.0012242 | 0.01765971 | -0.9139919 |
| Arl1 | P61211 | -1.9978526 | 0.58273595 | -4.7644235 | 0.00124809 | 0.01787756 | -0.9342664 |
| Cisd2 | Q9CQB5 | 4.11121218 | -1.5299979 | 4.75542985 | 0.00126309 | 0.01796594 | -0.9468004 |
| Vac14 | Q80WQ2 | -3.0620533 | -1.0884739 | -4.672842 | 0.00141036 | 0.01992131 | -1.0624581 |
| Hdac2 | P70288 | 2.13999814 | -0.9724864 | 4.66309108 | 0.00142894 | 0.02004452 | -1.0761798 |
| Rabif | Q91X96 | 3.72089534 | -1.9326694 | 4.65549805 | 0.00144359 | 0.02011132 | -1.0868746 |
| Fastkd3 | Q8BSN9 | 5.11201918 | 0.22120142 | 4.63261789 | 0.00148872 | 0.02059902 | -1.1191527 |
| Mtrex | Q9CZU3 | -1.7222829 | -1.5861578 | -4.6180615 | 0.00151823 | 0.02086536 | -1.139728 |
| Nip7 | Q9CXK8 | 1.41352131 | -3.1672091 | 4.60649021 | 0.00154213 | 0.02105168 | -1.1561062 |
| Nup188 | Q6ZQH8 | 2.9358496 | -2.7491365 | 4.59740847 | 0.00156118 | 0.02116963 | -1.1689743 |
| Dlst | Q9D2G2 | -1.0603252 | 1.9858495 | -4.5367598 | 0.00169509 | 0.02283318 | -1.2552177 |
| Vps4a | Q8VEJ9 | 1.11290544 | 0.0654804 | 4.5217174 | 0.00173019 | 0.02315263 | -1.2766912 |
| Zcchc8 | Q9CYA6 | -2.2343366 | -2.3336691 | -4.4878919 | 0.00181201 | 0.02408903 | -1.3250982 |
| Nme3 | Q9WV85 | -2.9004295 | -2.0230074 | -4.479428 | 0.00183313 | 0.02421156 | -1.3372365 |
| Nup210 | Q9QY81 | -1.0874423 | 0.01348324 | -4.4234067 | 0.00197978 | 0.02591625 | -1.4178394 |
| Emg1 | O35130 | -2.5751347 | -1.8553387 | -4.4205197 | 0.00198768 | 0.02591625 | -1.4220055 |
| Rbbp4 | Q60972 | 0.99568407 | 1.51779797 | 4.40226514 | 0.00203838 | 0.026408 | -1.4483749 |
| Mta2 | Q9R190 | 1.72725773 | -2.2156957 | 4.37294658 | 0.00212274 | 0.0273269 | -1.4908264 |
| Trim12a | Q99PQ1 | 1.65001379 | -0.1041728 | 4.36123909 | 0.00215747 | 0.02759934 | -1.5078123 |
| Jak1 | P52332 | 2.20609339 | -1.5238957 | 4.33801672 | 0.00222819 | 0.02832581 | -1.5415623 |
| Paf1 | Q8K2T8 | 3.07462988 | -2.1889063 | 4.2948264 | 0.00236642 | 0.02970937 | -1.6045351 |
| Samm50 | Q8BGH2 | -1.2820422 | -0.6090978 | -4.2861042 | 0.00239545 | 0.02970937 | -1.6172843 |
| Srrm1 | Q52KI8 | -1.5806458 | -0.6614122 | -4.2883782 | 0.00238784 | 0.02970937 | -1.6139593 |
| Tle3 | Q08122 | -3.1554358 | -0.6738031 | -4.2921388 | 0.00237533 | 0.02970937 | -1.6084624 |
| Rfc4 | Q99J62 | 3.41048855 | -1.2236705 | 4.26805209 | 0.00245673 | 0.0302848 | -1.6437044 |
| Nrbp1 | Q99J45 | -3.7612486 | -0.3008711 | -4.2608523 | 0.00248164 | 0.03040759 | -1.6542544 |
| Mvp | Q9EQK5 | -2.0693647 | -2.5320949 | -4.2145676 | 0.00264841 | 0.03225666 | -1.7222481 |
| Acat2 | Q8CAY6 | 2.779663 | -1.2065374 | 4.16568722 | 0.00283767 | 0.03435608 | -1.7943757 |
| Zmym2 | Q9CU65 | 3.00257594 | -2.9271533 | 4.15821082 | 0.00286788 | 0.03451633 | -1.8054366 |
| Prkcd | P28867 | 2.39467003 | -2.0033868 | 4.14675181 | 0.00291484 | 0.03487523 | -1.8224044 |
| Esco1 | Q69Z69 | 2.27694356 | -2.7780904 | 4.12403382 | 0.00301041 | 0.03559987 | -1.8560962 |
| Dbt | P53395 | -2.297218 | 0.53432344 | -4.1251851 | 0.00300549 | 0.03559987 | -1.854387 |
| Pak1ip1 | Q9DCE5 | -1.8993874 | -2.2168287 | -4.1102572 | 0.00307 | 0.03609466 | -1.8765615 |
| Rbbp7 | Q60973 | 0.88194719 | 1.68703609 | 4.10500862 | 0.00309303 | 0.03615646 | -1.8843649 |
| Ipo7 | Q9EPL8 | 2.23660643 | -2.2565615 | 4.07112478 | 0.00324625 | 0.0375341 | -1.9348315 |

| | | | | | | | |
|---|---|---|---|---|---|---|---|
| Gsdma | Q9EST1 | -1.9315638 | -2.6653518 | -4.0668305 | 0.00326624 | 0.0375341 | -1.9412382 |
| Traf5 | P70191 | -1.9359766 | -3.6721905 | -4.0680735 | 0.00326044 | 0.0375341 | -1.9393836 |
| Fxr1 | Q61584 | 2.99575374 | -1.6154838 | 4.03321791 | 0.00342735 | 0.03895431 | -1.9914702 |
| Smchd1 | Q6P5D8 | -1.6344579 | -2.3499417 | -4.0330582 | 0.00342813 | 0.03895431 | -1.9917092 |
| Ptgr3 | Q8BGC4 | 2.16275926 | -2.4375413 | 4.00396669 | 0.00357447 | 0.04018992 | -2.0353048 |
| Chchd3 | Q9CRB9 | -1.230215 | 1.61937354 | -4.0035942 | 0.00357639 | 0.04018992 | -2.0358636 |
| Ctps2 | P70303 | 1.01188303 | 0.31351885 | 3.99572199 | 0.00361715 | 0.04042465 | -2.04768 |
| Ppp1r2 | Q9DCL8 | 2.04846733 | -3.2356105 | 3.98719257 | 0.00366188 | 0.04070086 | -2.0604919 |
| Hacd3 | Q8K2C9 | 1.02004618 | -0.880819 | 3.97923518 | 0.00370414 | 0.04084222 | -2.0724529 |
| Jup | Q02257 | -1.5092863 | 0.87820655 | -3.977251 | 0.00371475 | 0.04084222 | -2.0754367 |
| Stard9 | Q80TF6 | -1.8365765 | -2.4338377 | -3.9635285 | 0.00378909 | 0.0414355 | -2.0960861 |
| Purb | O35295 | 2.00113832 | -2.1115627 | 3.94262206 | 0.0039054 | 0.04229857 | -2.1275919 |
| Ndufa10 | Q99LC3 | 1.19990572 | 0.41458847 | 3.93907562 | 0.00392551 | 0.04229857 | -2.1329418 |
| Cad | B2RQC6 | -1.2840243 | 0.85850501 | -3.9345735 | 0.00395119 | 0.04229857 | -2.1397357 |
| Mrpl45 | Q9D0Q7 | -2.0650574 | -2.375484 | -3.9354126 | 0.00394639 | 0.04229857 | -2.1384693 |
| Camk2d | Q6PHZ2 | -2.3903924 | -0.441749 | -3.929244 | 0.00398183 | 0.04240337 | -2.1477816 |
| Pccb | Q99MN9 | -2.0429246 | -2.3171645 | -3.9158596 | 0.00405989 | 0.04300949 | -2.1680033 |
| Tap1 | P21958 | 1.22192031 | -1.8038739 | 3.89239496 | 0.00420072 | 0.04409349 | -2.2035089 |
| Cand1 | Q6ZQ38 | 0.90629349 | 0.76636965 | 3.89160055 | 0.00420557 | 0.04409349 | -2.2047122 |
| Lef1 | P27782 | 0.98087335 | 0.70394509 | 3.85902028 | 0.00441006 | 0.04596266 | -2.2541272 |
| Mrpl55 | Q9CZ83 | -1.3256897 | -0.0809774 | -3.8560766 | 0.00442905 | 0.04596266 | -2.2585984 |
| Fam162a | Q9D6U8 | 1.66479789 | -3.3128755 | 3.8448252 | 0.00450244 | 0.04625234 | -2.2756978 |
| Ncoa5 | Q91W39 | -1.4323683 | -0.709447 | -3.8452311 | 0.00449977 | 0.04625234 | -2.2750806 |
| Sec16a | E9QAT4 | 1.9021204 | -3.0045495 | 3.80572894 | 0.0047677 | 0.04873119 | -2.3352333 |

| Table S11: Mass spectrometry analysis of HDAC8 in Foxp3+ Treg cells | | | | | | | |
|---|---|---|---|---|---|---|---|
| **Gene** | **Uniprot** | **logFC** | **AveExpr** | **t** | **P.Value** | **adj.P.Val** | **B** |
| Tbc1d32 | Q3URV1 | -9.2171103 | 0.58511509 | -21.264038 | 8.36E-09 | 1.12E-05 | 10.1432826 |
| Hdac8 | Q8VH37 | 6.5112272 | -0.5110879 | 18.2308001 | 3.11E-08 | 1.28E-05 | 9.19239834 |
| Nfat5 | Q9WV30-2 | 5.90378586 | -0.446268 | 17.3202126 | 4.80E-08 | 1.28E-05 | 8.85597797 |
| P18529 | NA | -7.9186305 | -0.3409494 | -17.549278 | 4.29E-08 | 1.28E-05 | 8.9431252 |
| Grxcr2 | Q3TYR5 | -8.1243706 | 0.20147637 | -19.025644 | 2.16E-08 | 1.28E-05 | 9.46531336 |
| Hipk2 | Q9QZR5 | 6.72985129 | -0.499112 | 16.1327016 | 8.76E-08 | 1.95E-05 | 8.37488154 |
| Pkn1 | P70268 | 4.75905633 | -1.3008851 | 15.2062859 | 1.44E-07 | 2.75E-05 | 7.96228317 |
| Fastkd3 | Q8BSN9 | 5.19768198 | -1.1977817 | 13.5959211 | 3.68E-07 | 5.46E-05 | 7.15519633 |
| Smg1 | Q8BKX6 | -4.9348099 | -1.2774477 | -13.606711 | 3.66E-07 | 5.46E-05 | 7.16102369 |
| Rfc2 | Q9WUK4 | 4.58925139 | -1.5100213 | 12.9076987 | 5.68E-07 | 7.57E-05 | 6.77066555 |
| Gstt3 | Q99L20 | 5.73534857 | -0.9877441 | 12.2921418 | 8.51E-07 | 1.03E-04 | 6.40408237 |
| Rif1 | Q6PR54 | 6.62442235 | -0.8139971 | 11.9768522 | 1.06E-06 | 1.17E-04 | 6.20744132 |
| Il2rb | P16297 | 5.01355123 | -1.1278094 | 11.5833812 | 1.39E-06 | 1.32E-04 | 5.95310444 |
| Myo1h | Q9D6A1 | -5.2239267 | -1.4813609 | -11.648022 | 1.33E-06 | 1.32E-04 | 5.99558852 |
| Add3 | Q9QYB5 | 4.07107079 | -1.9321136 | 10.240341 | 3.80E-06 | 3.38E-04 | 5.00296814 |
| Sptan1 | P16546 | 4.53999418 | 0.88085507 | 10.0286051 | 4.50E-06 | 3.65E-04 | 4.84052383 |
| Fhl3 | Q9R059 | 4.42105398 | -1.6021185 | 9.84967129 | 5.20E-06 | 3.65E-04 | 4.70033627 |
| Wdr18 | Q4VBE8 | 4.04856019 | -2.0927587 | 9.94166624 | 4.83E-06 | 3.65E-04 | 4.77274823 |
| Mccc2 | Q3ULD5 | -3.4075842 | -2.3381211 | -9.85486 | 5.18E-06 | 3.65E-04 | 4.70443959 |
| Ipo9 | Q91YE6 | 4.54468797 | -1.356958 | 9.66365597 | 6.06E-06 | 4.04E-04 | 4.55169745 |
| Znf639 | Q99KZ6 | 6.983813 | -0.5969511 | 9.20079945 | 8.98E-06 | 5.44E-04 | 4.16848254 |
| Rbm7 | Q9CQT2 | -2.9506942 | -2.5936387 | -9.2328654 | 8.73E-06 | 5.44E-04 | 4.19566392 |
| Mlycd | Q99J39 | 5.23183545 | -1.6075918 | 9.05437272 | 1.02E-05 | 5.91E-04 | 4.04312807 |
| Sptbn1 | Q62261 | 4.37139647 | 0.2211007 | 8.96699671 | 1.10E-05 | 6.07E-04 | 3.96735037 |
| Ppip5k1 | A2ARP1 | 3.8473939 | -1.6258978 | 8.93020012 | 1.14E-05 | 6.07E-04 | 3.93521701 |
| Hip1r | Q9JKY5 | -2.9983238 | -2.682695 | -8.8160009 | 1.26E-05 | 6.43E-04 | 3.83464607 |
| Stag3 | O70576 | -3.268442 | -2.1515763 | -8.7798821 | 1.30E-05 | 6.43E-04 | 3.80256934 |
| Mtmr3 | Q8K296 | -3.3523058 | -2.561703 | -8.5702838 | 1.57E-05 | 7.50E-04 | 3.61383869 |
| Cep43 | Q66JX5 | 3.97692839 | -1.9419724 | 8.51366462 | 1.66E-05 | 7.63E-04 | 3.56208775 |
| Ikzf5 | Q8BU00 | 4.21272613 | -1.2658958 | 8.37163179 | 1.89E-05 | 8.42E-04 | 3.4307995 |
| Herc4 | Q6PAV2 | 5.32225639 | -1.8611514 | 8.1765932 | 2.28E-05 | 9.49E-04 | 3.24703016 |
| Eprs1 | Q8CGC7 | -2.8267388 | -1.7956409 | -8.1965115 | 2.23E-05 | 9.49E-04 | 3.26598534 |
| Fbxl20 | Q9CZV8 | 3.8817575 | -1.8561179 | 7.90727751 | 2.95E-05 | 0.00115782 | 2.98644989 |
| Prkd2 | Q8BZ03 | -3.106049 | -2.2851675 | -7.9372297 | 2.87E-05 | 0.00115782 | 3.01583001 |
| Strn | O55106 | -2.8086373 | -2.3358745 | -7.8107036 | 3.24E-05 | 0.00123675 | 2.89102757 |
| Skap1 | Q3UUV5 | -3.0721301 | -3.0955055 | -7.6683481 | 3.74E-05 | 0.00138532 | 2.74841658 |
| Pycr3 | Q9DCC4 | -2.8365138 | -2.7060895 | -7.4733265 | 4.55E-05 | 0.0016419 | 2.5491888 |
| Aldh2 | P47738 | -3.4832907 | -2.1179884 | -7.4200823 | 4.81E-05 | 0.00168832 | 2.49400738 |
| Cad | B2RQC6 | -2.1586255 | 0.14650222 | -7.1219359 | 6.56E-05 | 0.00224514 | 2.17860139 |
| Mccc1 | Q99MR8 | -3.2078766 | -2.3733947 | -7.01856 | 7.33E-05 | 0.00241039 | 2.066648 |
| Pak6 | Q3ULB5 | -4.3796554 | -1.6098034 | -7.0083482 | 7.41E-05 | 0.00241039 | 2.05551512 |
| Top2b | Q64511 | 2.65223375 | -2.2487796 | 6.97445745 | 7.68E-05 | 0.00241095 | 2.01847165 |
| Ranbp2 | Q9ERU9 | -3.1510811 | -2.488014 | -6.9637941 | 7.77E-05 | 0.00241095 | 2.00678587 |
| Vars1 | Q9Z1Q9 | -2.06824 | -0.6763276 | -6.9286732 | 8.07E-05 | 0.00244714 | 1.96819385 |
| Ckb | Q04447 | 3.67497379 | 0.46407987 | 6.88590431 | 8.45E-05 | 0.00250622 | 1.92098254 |
| Cops3 | O88543 | -3.1140619 | -1.6001112 | -6.811883 | 9.16E-05 | 0.00265771 | 1.83871004 |
| Atad3 | Q925I1 | 5.5146529 | 1.35704851 | 6.78061243 | 9.48E-05 | 0.00269185 | 1.80373804 |
| Hspa1b | P17879 | 3.67241899 | -0.2566687 | 6.68630276 | 1.05E-04 | 0.00271191 | 1.69748304 |
| Eif2b5 | Q8CHW4 | 2.88352356 | -2.9048711 | 6.68226444 | 1.06E-04 | 0.00271191 | 1.69290686 |
| Dock10 | Q8BZN6 | -2.5071902 | -2.573146 | -6.6849863 | 1.05E-04 | 0.00271191 | 1.69599153 |
| Hpf1 | Q8CFE2 | -3.2819349 | -2.6720283 | -6.7529739 | 9.78E-05 | 0.00271191 | 1.77272085 |
| Camk2d | Q6PHZ2 | -3.5861593 | -1.6049668 | -6.7112671 | 1.02E-04 | 0.00271191 | 1.7257242 |
| NARS1 | Q8BP47 | -2.7011565 | -2.1757469 | -6.5968693 | 1.16E-04 | 0.00292633 | 1.59562692 |
| Rad52 | P43352 | 2.62781716 | -2.2995915 | 6.50776711 | 1.29E-04 | 0.00311713 | 1.49307617 |
| Ubash3a | Q3V3E1 | -3.611197 | -2.437163 | -6.5135316 | 1.28E-04 | 0.00311713 | 1.49974335 |
| Fhod1 | Q6P9Q4 | 3.49642423 | -2.5007409 | 6.41931506 | 1.42E-04 | 0.00332548 | 1.39020433 |
| Aldh16a1 | Q571I9 | -2.0961532 | -2.5317996 | -6.4247469 | 1.41E-04 | 0.00332548 | 1.39655263 |

| | | | | | | | |
|---|---|---|---|---|---|---|---|
| Dytn | A2CI98 | -3.0248328 | -1.978599 | -6.3809539 | 1.48E-04 | 0.00341463 | 1.34525533 |
| Uck1 | P52623 | -2.0323174 | -3.2827573 | -6.0844933 | 2.10E-04 | 0.00473989 | 0.99095967 |
| Ogdh | Q60597 | -2.1161285 | -0.1343181 | -5.9570434 | 2.44E-04 | 0.00542478 | 0.83481632 |
| Nup210 | Q9QY81 | -2.0731104 | -1.1762274 | -5.81998 | 2.88E-04 | 0.00629675 | 0.66427845 |
| Hspa1l | P16627 | 3.9566946 | 0.32858234 | 5.69615537 | 3.35E-04 | 0.00721028 | 0.50785492 |
| Snx6 | Q6P8X1 | 2.93629081 | -1.7107014 | 5.66249403 | 3.49E-04 | 0.00739728 | 0.46494174 |
| Pgm3 | Q9CYR6 | 2.51591936 | -2.3164289 | 5.63340208 | 3.62E-04 | 0.00754923 | 0.42771898 |
| Zzef1 | Q5SSH7 | 2.22673873 | -2.9751936 | 5.50212801 | 4.27E-04 | 0.00858733 | 0.25819361 |
| P01753 | NA | -2.4222609 | 1.22509541 | -5.4938742 | 4.31E-04 | 0.00858733 | 0.24744907 |
| Dbt | P53395 | -3.0006093 | -1.3510696 | -5.5086079 | 4.23E-04 | 0.00858733 | 0.26662186 |
| Flna | Q8BTM8 | -1.5845116 | 1.30965062 | -5.4443747 | 4.59E-04 | 0.00900768 | 0.18279832 |
| Dera | Q91YP3 | -2.5011601 | -1.9441655 | -5.394876 | 4.89E-04 | 0.00934206 | 0.11778198 |
| Uqcrb | Q9D855 | -3.0221008 | -2.6588726 | -5.3929199 | 4.90E-04 | 0.00934206 | 0.11520512 |
| Ablim1 | Q8K4G5 | 2.47132632 | -1.5815901 | 5.27531836 | 5.70E-04 | 0.0107112 | -0.0407739 |
| Adh5 | P28474 | 1.81465853 | 1.66464741 | 5.2551347 | 5.85E-04 | 0.01084177 | -0.0677534 |
| Dlst | Q9D2G2 | -1.3533533 | 1.97565435 | -5.1395295 | 6.80E-04 | 0.01243147 | -0.2234668 |
| Hcfc1 | Q61191 | 1.47527342 | -0.200427 | 5.09891561 | 7.18E-04 | 0.01293571 | -0.2786503 |
| Plrg1 | Q922V4 | -1.4540781 | -2.7985984 | -5.0746221 | 7.41E-04 | 0.01317871 | -0.3117778 |
| Arhgef1 | Q61210-5 | -1.9285331 | -1.7668988 | -4.9806306 | 8.39E-04 | 0.01473309 | -0.4407872 |
| Zcchc8 | Q9CYA6 | -2.0353739 | -2.5894452 | -4.9133359 | 9.18E-04 | 0.01591265 | -0.5339724 |
| Me2 | Q99KE1 | 1.68877919 | 0.39628313 | 4.87899665 | 9.62E-04 | 0.01645181 | -0.5817861 |
| Chchd3 | Q9CRB9 | -1.5887198 | -0.183773 | -4.8370308 | 0.00101809 | 0.01676709 | -0.6404597 |
| Polr2b | Q8CFI7 | -2.2477415 | -2.4290096 | -4.8448844 | 0.00100732 | 0.01676709 | -0.6294592 |
| Nat10 | Q8K224 | -2.6846396 | -2.4992497 | -4.8465653 | 0.00100503 | 0.01676709 | -0.627106 |
| Rars1 | Q9D0I9 | -2.5195233 | -1.8347253 | -4.8002349 | 0.00107023 | 0.01741084 | -0.6921227 |
| Tars1 | Q9D0R2 | -1.936486 | -1.335687 | -4.7819443 | 0.00109722 | 0.01763476 | -0.717879 |
| Stom | P54116 | -4.1226948 | -2.0310758 | -4.7539934 | 0.00113988 | 0.01810235 | -0.7573355 |
| Septin11 | Q8C1B7 | -1.3282799 | -1.3203314 | -4.7385229 | 0.00116426 | 0.01827199 | -0.7792243 |
| Smarcb1 | Q9Z0H3 | -2.0548619 | -1.7813746 | -4.7026557 | 0.00122297 | 0.01897023 | -0.8301094 |
| Tmem160 | Q9D938 | -2.3625764 | -3.2821994 | -4.6656089 | 0.00128697 | 0.01973359 | -0.882869 |
| Ppp6r1 | Q7TSI3 | -2.3072515 | -3.3663411 | -4.5824222 | 0.00144422 | 0.02189307 | -1.0020792 |
| Nfkb1 | P25799 | -2.2790234 | -2.578925 | -4.5397638 | 0.00153277 | 0.02297432 | -1.0636061 |
| Ankrd44 | B2RXR6 | -1.6823506 | -2.9432827 | -4.5059037 | 0.00160721 | 0.0238224 | -1.1126327 |
| Rnh1 | Q91VI7 | -1.1398905 | -0.2617634 | -4.4470079 | 0.0017461 | 0.02512836 | -1.1983066 |
| Nop56 | Q9D6Z1 | -2.2463694 | -1.3325496 | -4.4554078 | 0.00172553 | 0.02512836 | -1.1860567 |
| Mcm7 | Q61881 | -4.4653859 | -2.0861558 | -4.4446882 | 0.00175183 | 0.02512836 | -1.2016913 |
| Igtp | Q9DCE9 | -1.6583707 | -2.5583047 | -4.3789901 | 0.00192271 | 0.02728616 | -1.2978729 |
| Msmo1 | Q9CRA4 | 4.32618867 | -0.6749881 | 4.31707678 | 0.00210019 | 0.0285883 | -1.3890777 |
| Snx2 | Q9CWK8 | 2.91942059 | -1.2290442 | 4.33557609 | 0.00204539 | 0.0285883 | -1.3617694 |
| Evl | P70429 | -1.675071 | -1.5177607 | -4.3217349 | 0.00208625 | 0.0285883 | -1.3821969 |
| Paics | Q9DCL9 | -2.4594089 | -1.7032983 | -4.3212378 | 0.00208773 | 0.0285883 | -1.3829311 |
| Eef1e1 | Q9D1M4 | -1.2187241 | -2.1360067 | -4.3087213 | 0.00212545 | 0.02863997 | -1.4014278 |
| Prmt5 | Q8CIG8 | -2.7808355 | -1.7860408 | -4.2999852 | 0.00215222 | 0.0287106 | -1.414351 |
| Eif3d | O70194 | -2.9043207 | -1.5598194 | -4.2767923 | 0.00222504 | 0.02938813 | -1.4487115 |
| Hectd3 | Q3U487 | 2.2081946 | -2.43698 | 4.23334968 | 0.00236863 | 0.03097793 | -1.5132745 |
| Caprin1 | Q60865 | -2.2617794 | -2.6809251 | -4.0904714 | 0.00291509 | 0.0377547 | -1.7274388 |
| Septin7 | O55131 | -1.5506679 | 0.43591036 | -4.0823263 | 0.00295005 | 0.03784013 | -1.7397304 |
| Mthfd1l | Q3V3R1 | -1.164332 | -0.6756065 | -4.0129769 | 0.00326648 | 0.04149982 | -1.8447368 |
| Sumo2 | P61957 | 1.90755238 | -0.8978 | 3.96989898 | 0.00348108 | 0.04339967 | -1.9102768 |
| Gbp2 | Q9Z0E6 | -3.3262855 | -1.6868169 | -3.9742904 | 0.00345853 | 0.04339967 | -1.9035847 |
| Dhx15 | O35286 | -1.052466 | 0.52919883 | -3.908498 | 0.00381327 | 0.04710096 | -2.004099 |
| Dpysl2 | O08553 | -1.4271247 | 0.02892789 | -3.8816942 | 0.0039687 | 0.04812951 | -2.0452022 |
| Arhgap45 | Q3TBD2 | -1.8173176 | -0.223951 | -3.8834412 | 0.00395837 | 0.04812951 | -2.0425207 |
| Dars1 | Q922B2 | -1.1216214 | 0.56195581 | -3.8581262 | 0.00411093 | 0.04896408 | -2.0814156 |
| Mvp | Q9EQK5 | -1.6196985 | -2.674093 | -3.8601746 | 0.00409835 | 0.04896408 | -2.0782655 |

**Table S12: Antibodies used in this study**

| Protein targets | Assays | Company and product code |
|---|---|---|
| **Socs3** | WB | Cell Signaling Technology #52113 |
| **HDAC8** | WB/ChIP | Cell Signaling Technology #66042 |
| **p-HDAC8** | WB | Thermo Fisher Scientific # PA5-105031 |
| **Ace-SMC3** | WB | Thermo Fisher Scientific #65728 |
| **p-ERK** | WB | Cell Signaling Technology #4370 |
| **p-p38** | WB | Cell Signaling Technology #4511 |
| **p-LCK** | WB | Cell Signaling Technology #70926 |
| **p-AKT** | WB | Cell Signaling Technology #4060 |
| **p-Zap70** | WB | Cell Signaling Technology #2717 |
| **p-SLP76** | WB | Cell Signaling Technology #76384 |
| **H3K27ac** | ChIP | Cell Signaling Technology #8173 |
| **β-Actin** | WB | Cell Signaling Technology #8457 |
| **Goat anti-rabbit IgG** | WB | Cell Signaling Technology #7074 |
| **Pacific Blue anti-CD4** | Flow cytometry | BD Biosciences #558107 |
| **FITC anti-CD8a** | Flow cytometry | Thermo Fisher Scientific #11-0087-42 |
| **FITC anti-CD25** | Flow cytometry | BD Biosciences #553072 |
| **Alexa Fluor™ 700 anti-Ki-67** | Flow cytometry | Thermo Fisher Scientific #56-5698-82 |
| **PE-Cyanine 5.5 anti-Foxp3** | Flow cytometry | Thermo Fisher Scientific #35-5773-82 |
| **APC anti-IL-2** | Flow cytometry | Thermo Fisher Scientific # 17-7021-82 |
| **PE anti-IFN-γ** | Flow cytometry | BioLegend # 505808 |
| **Alexa Fluor™ 700 anti-IFN-γ** | Flow cytometry | Thermo Fisher Scientific # 56-7311-82 |

## Table S13: The number of cells for each cell subset per flow cytometry

| Fig.1f: Flow cytometric analysis of cell numbers in TC-1 tumors from CD4-Cre and $HDAC8^{-/-}$ groups | | | | | | |
|---|---|---|---|---|---|---|
| | CD4-Cre | | | $HDAC8^{-/-}$ | | |
| **CD4** | 3484 | 5680 | 2744 | 12416 | 18720 | 14520 |
| **CD8** | 8040 | 12496 | 6272 | 18236 | 24960 | 20592 |

| Fig.1g: Flow cytometric analysis of cell numbers of IFNg expression by CD4/CD8+ T cells within TC-1 tumors in CD4-Cre and $HDAC8^{-/-}$ groups | | | | | | |
|---|---|---|---|---|---|---|
| | CD4-Cre | | | $HDAC8^{-/-}$ | | |
| **CD4+IFNg+** | 1578 | 2885 | 1413 | 7797 | 11194 | 9031 |
| **CD8+IFNg+** | 5008 | 8234 | 4001 | 14497 | 18769 | 14846 |

| Fig.2b: Flow cytometric analysis of cell numbers of CD4/CD8+ T cells within HCC tumors in DMSO and HDAC8i groups(10 days) | | | | | | |
|---|---|---|---|---|---|---|
| | DMSO | | | HDAC8i | | |
| **CD4** | 53676 | 49588 | 44748 | 37706 | 45920 | 45342 |
| **CD8** | 23643 | 26059 | 20988 | 33516 | 65520 | 35838 |
| **CD4+CD25+** | 6656 | 4949 | 2381 | 2289 | 2177 | 2231 |
| **CD4+Foxp3+** | 1487 | 988 | 1782 | 1231 | 967 | 850 |

| Fig.2c: Flow cytometric analysis of cell numbers of CD4/CD8+ T cells within HCC tumors in DMSO and HDAC8i groups(21 days) | | | | | | |
|---|---|---|---|---|---|---|
| | DMSO | | | HDAC8i | | |
| **CD4** | 19511 | 4509 | 69560 | 52160 | 98604 | 48146 |
| **CD8** | 43164 | 38105 | 57152 | 84353 | 97235 | 81450 |
| **CD4+CD25+** | 6263 | 1551 | 13286 | 19091 | 34413 | 15022 |
| **CD4+Foxp3+** | 755 | 985 | 538 | 340 | 3878 | 745 |

| Fig.3a: Flow cytometric analysis of cell numbers of Ki-67, IL-2and IFN-γ+ T cells within HCC tumors in DMSO and HDAC8i groups(10 days) | | | | | | |
|---|---|---|---|---|---|---|
| | DMSO | | | HDAC8i | | |
| Ki-67 in CD4+ T cells | 27003 | 21902 | 17295 | 45758 | 21066 | 21177 |
| Ki-67 in CD8+ T cells | 7827 | 5339 | 8251 | 6063 | 3542 | 3442 |
| IL-2 in CD4+ T cells | 263 | 608 | 609 | 270 | 379 | 257 |
| IL-2 in CD8+ T cells | 271 | 341 | 334 | 328 | 242 | 361 |
| IFN-γ in CD4+ T cells | 3127 | 2268 | 2898 | 2978 | 982 | 1418 |
| IFN-γ in CD8+ T cells | 149 | 168 | 118 | 67 | 154 | 65 |

| Fig.3b: Flow cytometric analysis of cell numbers of Ki-67, IL-2and IFN-γ+ T cells within HCC tumors in DMSO and HDAC8i groups(21 days) | | | | | | |
|---|---|---|---|---|---|---|
| | DMSO | | | HDAC8i | | |
| Ki-67 in CD4+ T cells | 3521 | 2704 | 2920 | 1081 | 3523 | 918 |
| Ki-67 in CD8+ T cells | 2819 | 3159 | 1914 | 1486 | 11175 | 1637 |
| IL-2 in CD4+ T cells | 1492 | 1627 | 406 | 1647 | 1694 | 2926 |
| IL-2 in CD8+ T cells | 1590 | 1841 | 1794 | 2385 | 1058 | 3996 |
| IFN-γ in CD4+ T cells | 761 | 734 | 219 | 1169 | 1325 | 2035 |
| IFN-γ in CD8+ T cells | 1018 | 1523 | 828 | 901 | 449 | 1916 |

| Fig.4a: Flow cytometric analysis of cell numbers of CD4+CD25+and CD8+CD25+ T cells in CD4-Cre and HDAC8$^{-/-}$ groups | | | | | | |
|---|---|---|---|---|---|---|
| | CD4-Cre | | | HDAC8-/- | | |
| CD4+CD25+ T cells | 81411 | 88953 | 145476 | 296140 | 180429 | 145143 |
| CD8+CD25+ T cells | 43887 | 41471 | 63489 | 158722 | 82152 | 61912 |

| Fig.4c: Flow cytometric analysis of cell numbers of CD4+IL-2+ T cells in CD4-Cre and HDAC8$^{-/-}$ groups | | | | | | |
|---|---|---|---|---|---|---|
| | CD4-Cre | | | HDAC8-/- | | |
| CD4+IL-2(Fresh) | 15639 | 17116 | 18231 | 127636 | 75179 | 79211 |
| CD4+IL-2(PMA/ion 4h) | 192815 | 238640 | 320416 | 1181599 | 625863 | 618693 |

| Fig.4d-e: Flow cytometric analysis of cell numbers of IL-2+ T cells in CD4-Cre and HDAC8$^{-/-}$ groups | | | | | | |
|---|---|---|---|---|---|---|
| | CD4-Cre | | | HDAC8-/- | | |
| CD4+IFNg(Treg) | 181 | 329 | 392 | 5235 | 5232 | 3284.4 |
| CD4+IFNg(Teff) | 226 | 408 | 313 | 4633 | 4380 | 3141.6 |
| CD8+IFNg | 2490 | 3731 | 2720 | 11881 | 10176 | 9765 |

| Fig.6a: The stability of Foxp3 in lymph nodes and spleen was analyzed by flow cytometry 10 days after CD4-Cre and HDAC8$^{-/-}$ Treg cells were injected into immunodeficient Rag1$^{-/-}$mice (n=3/group) | | | | | | |
|---|---|---|---|---|---|---|
| | CD4-Cre | | | HDAC8-/- | | |
| CD4+Foxp3+(LN) | 206018 | 144296 | 144775 | 152404 | 99172 | 120868 |
| CD4+Foxp3+(SP) | 191084 | 257961 | 111860 | 121334 | 108709 | 51060 |

| Fig.6b: Flow cytometry was used to analyze Foxp3 within pooled (n=3/group) Treg cells from lymph nodes and spleens of CD4-Cre and HDAC8$^{-/-}$ mice, as indicated (CD3/CD28 stimulation 24 h) | | | | | | |
|---|---|---|---|---|---|---|
| | CD4-Cre | | | HDAC8-/- | | |
| CD4+Foxp3+(0h) | 182965 | 179310 | 193758 | 188985 | 198036.5 | 183180 |
| CD4+Foxp3+(24h) | 161035 | 165571.5 | 149210 | 112015 | 97610 | 115240 |

| Fig. S1: Conditional knockout of HDAC8 has negligible effects on T cell development. | | | | | | |
|---|---|---|---|---|---|---|
| | Lymph nodes | | | | | |
| Group | CD4-Cre | | | HDAC8$^{-/-}$ | | |
| CD4+ T cells | 1511395 | 1192556 | 1796754 | 1653718 | 2149616 | 2989350 |
| CD8+ T cells | 2274290 | 1556785 | 2571825 | 1634489 | 1760252 | 2212119 |
| CD4+CD25+ T cells | 190436 | 132374 | 166739 | 188524 | 211092 | 334807 |
| CD4+Foxp3+ T cells | 198713 | 129122 | 177150 | 194750 | 232117 | 336477 |
| Foxp3+Ki-67+ | 23846 | 24404 | 36493 | 43624 | 44566 | 98588 |
| CD4+Ki-67+ | 186096 | 131448 | 175336 | 188204 | 264964 | 409624 |
| CD8+Ki-67+ | 153667 | 109735 | 212885 | 78264 | 122978 | 128439 |
| CD4+CD69+ | 175322 | 143107 | 206627 | 234828 | 270852 | 388616 |
| CD8+CD69+ | 221971 | 160349 | 233522 | 148738 | 154198 | 205285 |
| CD4+CD44+CD62L- | 90986 | 70957 | 110321 | 114437 | 124678 | 188030 |
| CD8+CD44+CD62L- | 90062 | 62116 | 129106 | 56553 | 91885 | 98439 |
| | Spleen | | | | | |
| | CD4-Cre | | | HDAC8$^{-/-}$ | | |
| CD4+ T cells | 4300156 | 4555431 | 6286342 | 6103082 | 6617638 | 7794909 |
| CD8+ T cells | 4409482 | 3973158 | 5837317 | 3614599 | 3933036 | 4061296 |
| CD4+CD25+ T cells | 481617 | 417733 | 588402 | 671339 | 681617 | 787286 |
| CD4+Foxp3+ T cells | 572069 | 640402 | 653730 | 785836 | 707118 | 763219 |
| Foxp3+Ki-67+ | 89815 | 104385 | 72564 | 113160 | 65479 | 106851 |
| CD4+Ki-67+ | 559722 | 792440 | 704891 | 733447 | 920473 | 947209 |
| CD8+Ki-67+ | 278785 | 395720 | 440167 | 300477 | 400736 | 229232 |
| CD4+CD69+ | 411525 | 642316 | 491592 | 707957 | 620734 | 622034 |
| CD8+CD69+ | 146836 | 137471 | 165196 | 260251 | 314643 | 235149 |
| CD4+CD44+CD62L- | 915933 | 1179857 | 1307559 | 1440327 | 1343380 | 1520007 |
| CD8+CD44+CD62L- | 440948 | 480752 | 723827 | 437367 | 668616 | 592949 |
| | Thymus | | | | | |
| | CD4-Cre | | | HDAC8$^{-/-}$ | | |
| CD4+ T cells | 6800112 | 4785178 | 5613294 | 6007949 | 9428932 | 7629518 |
| CD8+ T cells | 1520402 | 790884 | 1168982 | 1493238 | 2343540 | 1759601 |
| CD4+CD25+ T cells | 201283 | 69864 | 221164 | 112349 | 236666 | 204471 |
| CD4+Foxp3+ T cells | 44548 | 28946 | 47718 | 37544 | 94159 | 57022 |
| Foxp3+Ki-67+ | 6058 | 1931 | 9162 | 3942 | 7241 | 5702 |
| CD4+Ki-67+ | 966632 | 792031 | 793861 | 762216 | 1336826 | 1090635 |
| CD8+Ki-67+ | 35201 | 54902 | 58502 | 16067 | 93996 | 69223 |
| CD4+CD69+ | 5392489 | 3713298 | 4400822 | 4523985 | 7128273 | 5577178 |
| CD8+CD69+ | 588396 | 240429 | 403299 | 530099 | 857735 | 652812 |
| CD4+CD44+CD62L- | 248204 | 245480 | 315467 | 326832 | 467675 | 466164 |
| CD8+CD44+CD62L- | 86967 | 48718 | 107196 | 61671 | 70541 | 82349 |

| Fig.S2a: Flow cytometric analysis of cell numbers of CD4/CD8+ T cells within HCC spleen in DMSO and HDAC8i groups(10 days) | | | | | | |
|---|---|---|---|---|---|---|
| | DMSO | | | HDAC8i | | |
| **CD4** | 189476 | 196443 | 184470 | 234438 | 252120 | 206079 |
| **CD8** | 98490 | 109135 | 107844 | 123890 | 114600 | 104451 |
| **CD4+CD25+** | 29937 | 25145 | 34127 | 28367 | 26977 | 23905 |
| **CD4+Foxp3+** | 1342 | 980 | 1183 | 434 | 649 | 522 |

| Fig.S2b: Flow cytometric analysis of cell numbers of CD4/CD8+ T cells within HCC spleen in DMSO and HDAC8i groups(21 days) | | | | | | |
|---|---|---|---|---|---|---|
| | DMSO | | | HDAC8i | | |
| **CD4** | 154574 | 142926 | 188395 | 107120 | 233996 | 155568 |
| **CD8** | 58670 | 48388 | 72142 | 50182 | 99736 | 73247 |
| **CD4+CD25+** | 18858 | 21296 | 19970 | 15211 | 28548 | 21468 |
| **CD4+Foxp3+** | 984 | 897 | 905 | 1421 | 2990 | 1033 |

| Fig.S3a: Flow cytometric analysis of cell numbers of Ki-67, IL-2and IFN-γ+ T cells within HCC spleen in DMSO and HDAC8i groups(10 days) | | | | | | |
|---|---|---|---|---|---|---|
| | DMSO | | | HDAC8i | | |
| Ki-67 in CD4+ T cells | 27788 | 23203 | 26995 | 15702 | 17986 | 18248 |
| Ki-67 in CD8+ T cells | 11281 | 8307 | 7835 | 4564 | 5367 | 5649 |
| IL-2 in CD4+ T cells | 4459 | 7468 | 5059 | 9621 | 5853 | 5828 |
| IL-2 in CD8+ T cells | 1302 | 1562 | 1200 | 1842 | 1242 | 1280 |
| IFN-γ in CD4+ T cells | 899 | 922 | 750 | 1122 | 906 | 890 |
| IFN-γ in CD8+ T cells | 1009 | 1163 | 909 | 1051 | 878 | 744 |

| Fig.S3b: Flow cytometric analysis of cell numbers of Ki-67, IL-2and IFN-γ+ T cells within HCC spleen in DMSO and HDAC8i groups(21 days) | | | | | | |
|---|---|---|---|---|---|---|
| | DMSO | | | HDAC8i | | |
| Ki-67 in CD4+ T cells | 26187 | 15539 | 21756 | 26050 | 23765 | 18570 |
| Ki-67 in CD8+ T cells | 6148 | 5715 | 4642 | 7681 | 22150 | 6186 |
| IL-2 in CD4+ T cells | 8973 | 6970 | 2935 | 1432 | 6166 | 6829 |
| IL-2 in CD8+ T cells | 3970 | 4543 | 1795 | 759 | 2124 | 2921 |
| IFN-γ in CD4+ T cells | 3015 | 3050 | 1963 | 1359 | 2943 | 2549 |
| IFN-γ in CD8+ T cells | 6477 | 8411 | 5409 | 3793 | 6014 | 6873 |

| Fig.S4a: Flow cytometric analysis of cell numbers of CD4/CD8+ T cells within HCC lymph nodes in DMSO and HDAC8i groups(10 days) | | | | | | |
|---|---|---|---|---|---|---|
| | DMSO | | | HDAC8i | | |
| **CD4** | 490896 | 302400 | 409812 | 439760 | 612155 | 541872 |
| **CD8** | 190904 | 132480 | 137566 | 170168 | 185270 | 176490 |
| **CD4+CD25+** | 55471 | 32659 | 40448 | 38743 | 42177 | 36793 |
| **CD4+Foxp3+** | 810 | 416 | 342 | 273 | 271 | 194 |

| Fig.S4b: Flow cytometric analysis of cell numbers of CD4/CD8+ T cells within HCC lymph nodes in DMSO and HDAC8i groups(21 days) | | | | | | |
|---|---|---|---|---|---|---|
| | DMSO | | | HDAC8i | | |
| **CD4** | 558540 | 488796 | 395932 | 561974 | 410975 | 332304 |
| **CD8** | 189711 | 159390 | 145111 | 238791 | 178895 | 140070 |
| **CD4+CD25+** | 44236 | 32945 | 34486 | 43103 | 36906 | 35557 |
| **CD4+Foxp3+** | 1242 | 1170 | 1100 | 975 | 720 | 1584 |

| Fig.S5a: Flow cytometric analysis of cell numbers of Ki-67, IL-2and IFN-γ+ T cells within HCC lymph nodes in DMSO and HDAC8i groups(10 days) | | | | | | |
|---|---|---|---|---|---|---|
| | DMSO | | | HDAC8i | | |
| Ki-67 in CD4+ T cells | 30665 | 22462 | 18136 | 18246 | 14991 | 16327 |
| Ki-67 in CD8+ T cells | 6865 | 4481 | 3972 | 3009 | 3410 | 2827 |
| IL-2 in CD4+ T cells | 36688 | 11132 | 26070 | 25458 | 29841 | 32907 |
| IL-2 in CD8+ T cells | 2703 | 2604 | 3218 | 2811 | 3122 | 3667 |
| IFN-γ in CD4+ T cells | 4267 | 1033 | 1560 | 1238 | 2638 | 2003 |
| IFN-γ in CD8+ T cells | 2413 | 625 | 1905 | 1554 | 1329 | 1834 |

| Fig.S5b: Flow cytometric analysis of cell numbers of Ki-67, IL-2and IFN-γ+ T cells within HCC lymph nodes in DMSO and HDAC8i groups(21 days) | | | | | | |
|---|---|---|---|---|---|---|
| | DMSO | | | HDAC8i | | |
| Ki-67 in CD4+ T cells | 34902 | 24916 | 31462 | 24153 | 20276 | 28258 |
| Ki-67 in CD8+ T cells | 10704 | 9433 | 10091 | 7988 | 7625 | 13890 |
| IL-2 in CD4+ T cells | 78864 | 70762 | 54174 | 16403 | 24657 | 21921 |
| IL-2 in CD8+ T cells | 26911 | 23293 | 16696 | 2373 | 3009 | 14900 |
| IFN-γ in CD4+ T cells | 4433 | 5097 | 3902 | 1975 | 3158 | 3581 |
| IFN-γ in CD8+ T cells | 12843 | 14538 | 13009 | 6176 | 9174 | 11572 |

| Fig.S6b-c: Flow cytometric analysis of cell numbers of CD4/CD8/Foxp3+ T cells within HCC tumors in CD4-Cre and HDAC8$^{-/-}$ groups | | | | | | |
|---|---|---|---|---|---|---|
| | CD4-Cre | | | HDAC8-/- | | |
| **CD4(Tumor)** | 21952 | 21280 | 13440 | 28224 | 25648 | 35168 |
| **CD4(Spleen)** | 148736 | 150192 | 128352 | 176960 | 176064 | 157136 |
| **CD4(Lymph nodes)** | 288176 | 237776 | 240576 | 291200 | 362208 | 374864 |
| **CD8(Tumor)** | 23968 | 29456 | 16128 | 41552 | 42336 | 32032 |
| **CD8(Spleen)** | 76832 | 74928 | 96208 | 124208 | 123536 | 101472 |
| **CD8(Lymph nodes)** | 317520 | 498624 | 503664 | 346528 | 256928 | 282688 |
| **CD4+Foxp3(Tumor)** | 2432 | 2662 | 1431 | 2266 | 2216 | 3577 |
| **CD4+Foxp3(Spleen)** | 33480 | 34935 | 24284 | 33941 | 34456 | 26336 |
| **CD4+Foxp3(Lymph nodes)** | 46454 | 25941 | 25669 | 32207 | 52737 | 55367 |

| Fig.S7: Flow cytometric analysis of cell numbers of Ki-67 within CD4/CD8/Foxp3+ T cells in CD4-Cre and HDAC8$^{-/-}$ groups | | | | | | |
|---|---|---|---|---|---|---|
| | CD4-Cre | | | HDAC8-/- | | |
| **Ki-67/CD4(Tumor)** | 2465 | 5077 | 2148 | 7381 | 8028 | 15752 |
| **Ki-67/CD4(Spleen)** | 34254 | 35580 | 26081 | 30933 | 29913 | 31082 |
| **Ki-67/CD4(Lymph nodes)** | 29769 | 21495 | 23384 | 26587 | 27310 | 26428 |
| **Ki-67/CD8(Tumor)** | 4321 | 14389 | 5772 | 24391 | 26261 | 18998 |
| **Ki-67/CD8(Spleen)** | 12040 | 11427 | 14210 | 25177 | 24238 | 25064 |
| **Ki-67/CD8(Lymph nodes)** | 24894 | 38643 | 40343 | 20341 | 16752 | 19279 |
| **Ki-67/CD4+Foxp3+(Tumor)** | 410 | 1122 | 382 | 1263 | 1243 | 1703 |
| **Ki-67/CD4+Foxp3+(Spleen)** | 11209 | 11818 | 8463 | 11662 | 11422 | 9002 |
| **Ki-67/CD4+Foxp3+(Lymph nodes)** | 8603 | 5199 | 5845 | 6039 | 7552 | 8421 |
| **Ki-67/CD4+Foxp3-(Tumor)** | 1601 | 3549 | 1559 | 5879 | 6812 | 13963 |
| **Ki-67/CD4+Foxp3-(Spleen)** | 22475 | 23236 | 17317 | 18821 | 18013 | 21543 |
| **Ki-67/CD4+Foxp3-(Lymph nodes)** | 20546 | 15633 | 16569 | 19865 | 18940 | 17317 |

| Fig.S8: Flow cytometric analysis of cell numbers of IL-2 within CD4/CD8/Foxp3+ T cells in CD4-Cre and HDAC8$^{-/-}$ groups | | | | | | |
|---|---|---|---|---|---|---|
| | CD4-Cre | | | HDAC8-/- | | |
| **IL-2/CD4(Tumor)** | 134 | 72 | 99 | 541 | 545 | 580 |
| **IL-2/CD4(Spleen)** | 7090 | 11769 | 9296 | 18442 | 16363 | 18485 |
| **IL-2/CD4(Lymph nodes)** | 1192 | 1286 | 1663 | 2112 | 1755 | 1975 |
| **IL-2/CD8(Tumor)** | 17 | 24 | 15 | 71 | 215 | 162 |
| **IL-2/CD8(Spleen)** | 2592 | 3699 | 4079 | 7308 | 6016 | 6714 |
| **IL-2/CD8(Lymph nodes)** | 1879 | 2711 | 3415 | 1232 | 1474 | 1270 |
| **IL-2/CD4+Foxp3+(Tumor)** | 9 | 0 | 7 | 177 | 159 | 138 |
| **IL-2/CD4+Foxp3+(Spleen)** | 361 | 1015 | 1499 | 4888 | 3473 | 3787 |
| **IL-2/CD4+Foxp3+(Lymph nodes)** | 137 | 430 | 617 | 63 | 57 | 120 |
| **IL-2/CD4+Foxp3-(Tumor)** | 160 | 88 | 135 | 483 | 545 | 531 |

| | | | | | | |
|---|---|---|---|---|---|---|
| **IL-2/CD4+Foxp3-(Spleen)** | 6667 | 5780 | 7853 | 14724 | 13909 | 15558 |
| **IL-2/CD4+Foxp3-(Lymph nodes)** | 2473 | 2789 | 2654 | 3188 | 3408 | 3477 |

| **Fig.S9: Flow cytometric analysis of cell numbers of IFNg within CD4/CD8/Foxp3+ T cells in CD4-Cre and HDAC8$^{-/-}$ groups** | | | | | | |
|---|---|---|---|---|---|---|
| | CD4-Cre | | | HDAC8-/- | | |
| **IFNg/CD4(Tumor)** | 1864 | 917 | 841 | 1872 | 3112 | 3005 |
| **IFNg/CD4(Spleen)** | 13231 | 12167 | 12464 | 26040 | 21917 | 21863 |
| **IFNg/CD4(Lymph nodes)** | 3889 | 4013 | 2978 | 5234 | 5329 | 4776 |
| **IFNg/CD8(Tumor)** | 661 | 394 | 332 | 516 | 3912 | 3344 |
| **IFNg/CD8(Spleen)** | 23442 | 23257 | 25658 | 29958 | 24724 | 24917 |
| **IFNg/CD8(Lymph nodes)** | 19389 | 7277 | 7790 | 22177 | 25343 | 21168 |
| **IFNg/CD4+Foxp3+(Tumor)** | 0 | 8 | 7 | 130 | 184 | 192 |
| **IFNg/CD4+Foxp3+(Spleen)** | 481 | 239 | 240 | 1175 | 1002 | 1063 |
| **IFNg/CD4+Foxp3+(Lymph nodes)** | 638 | 1108 | 865 | 813 | 1024 | 896 |
| **IFNg/CD4+Foxp3-(Tumor)** | 1606 | 805 | 728 | 1425 | 2585 | 2483 |
| **IFNg/CD4+Foxp3-(Spleen)** | 12739 | 11451 | 12229 | 24693 | 20529 | 20488 |
| **IFNg/CD4+Foxp3-(Lymph nodes)** | 3649 | 3458 | 2654 | 4555 | 4759 | 4554 |

| **Fig.S11: Conditional knockout of HDAC8 impairs the immunosuppressive function of Treg cells in ConA-mediated autoimmune hepatitis(n=3/group)** | | | | | | |
|---|---|---|---|---|---|---|
| | **Teff(WT)+Treg(WT)** | | | **Teff(WT)+Treg(HDAC8$^{-/-}$)** | | |
| **CD4+T cells** | 332800 | 262000 | 302800 | 340800 | 326000 | 361200 |
| **CD4+Foxp3+** | 21299 | 18733 | 25072 | 19153 | 17669 | 11956 |
| **IFNg(Teff)** | 24577 | 30895 | 16414 | 40206 | 33916 | 24307 |
| **IFNg(Treg)** | 1201 | 654 | 677 | 1107 | 1873 | 742 |
| **IL-2(Teff)** | 13332 | 6106 | 8971 | 4664 | 8695 | 21793 |
| **IL-2(Treg)** | 109 | 0 | 338 | 222 | 134 | 405 |
| | **Teff(HDAC8$^{-/-}$)+Treg(WT)** | | | **Teff(HDAC8$^{-/-}$)+Treg(HDAC8$^{-/-}$)** | | |
| **CD4+T cells** | 344800 | 172800 | 345200 | 373200 | 376800 | 374400 |
| **CD4+Foxp3+** | 18654 | 10558 | 20677 | 29520 | 17823 | 31075 |
| **IFNg(Teff)** | 16862 | 5029 | 13241 | 82139 | 59949 | 20496 |
| **IFNg(Treg)** | 1319 | 285 | 744 | 6406 | 3582 | 2467 |
| **IL-2(Teff)** | 4599 | 3423 | 9379 | 9726 | 14180 | 12634 |
| **IL-2(Treg)** | 95 | 95 | 372 | 744 | 1130 | 469 |

| Fig.S13a: Flow cytometry analysis of CD4-Cre and HDAC8-/- iTreg cells were stimulated with CD3/CD28 beads (1:1) plus TGF-β and IL-2 for the indicated times(n=3/group) | | | | | | |
|---|---|---|---|---|---|---|
| | CD4-Cre | | | $HDAC8^{-/-}$ | | |
| **0h** | 34800 | 27600 | 25500 | 18900 | 21900 | 22400 |
| **24h** | 259400 | 199000 | 153600 | 251600 | 204000 | 164300 |
| **48h** | 549700 | 618000 | 661900 | 569100 | 629000 | 670800 |
| **72h** | 801600 | 735000 | 670400 | 854600 | 783000 | 702200 |

**Table S14: Primers sequences used for real-time PCR and ChIP PCR**

| Target Genes | Primers sequences used for real-time PCR |
|---|---|
| **CD4** | Forward: AGATACCCCAGGTCTCGCTT<br>Reverse: TGCCTGGCGCTGTTGG |
| **CD8** | Forward: TGAAGTGTTGGGGTCCGTTT<br>Reverse: CATTTGCAAACACGCTTTCGG |
| **Foxp3** | Forward: ATATGCGACCCCCTTTCACC<br>Reverse: TTGGCTCCTCTTCTTGCGAA |
| **Granzyme B** | Forward: GGACATGAAGTCAAGCCCCA<br>Reverse: CCCGAAAGGAAGCACGTTTG |
| **IFN-γ** | Forward: CGGCACAGTCATTGAAAGCC<br>Reverse: TGCATCCTTTTTCGCCTTGC |
| **Fas** | Forward: TGCTTGCTGGCTCACAGTTA<br>Reverse: GAATCACTCCAACGGGCTGA |
| **Fasl** | Forward: AGCAGTCAGCGTCAGAGTTC<br>Reverse: GTACTGGGGTTGGCTCACG |
| **IL-2** | Forward: TGAGTCAGCAACTGTGGTGG<br>Reverse: GCCCTTGGGGCTTACAAAAAG |
| **IL-6** | Forward: GGGACTGATGCTGGTGACAA<br>Reverse: AGCATTGGAAATTGGGGTAGGA |
| **IL-17f** | Forward: ACGTGAATTCCAGAACCGCT<br>Reverse: TTGGAGATCGGGCTTCACAC |
| **β-Actin** | Forward: TGAGCTGCGTTTTACACCCT<br>Reverse: AAGTCAGTGTACAGGCCAGC |
| **Target Genes** | **Primers sequences used for ChIP-PCR** |
| **Fas** | Forward: CCTGAGGCTTCTTCATGGCA<br>Reverse: GTCACCCTCCTTCCTCTCCT |
| **Fasl** | Forward: CTAGCTGTGAGCACTCTCCC<br>Reverse: AGCTGCAGATGTTTGCACTG |
| **IL-2** | Forward: TGCAGGAAGTTGTCCAGTCC<br>Reverse: TGGTTCCCAGTGTGATGGTG |
| **IL-6** | Forward: AGACCTTCAAGCCTCCTTGC<br>Reverse: GCATTCTCCCCAGTGGTCTC |
| **IL-17** | Forward: TGTATTGGCAGTAGCCACCC<br>Reverse: TGCACTGTATCCAGGCAAGG |
