# Supplementary material for: Differential effects of HDAC8 targeting on Foxp3+ Tregs and effector T cells promote antitumor immunity
Source: JCI Insight. 2025 Dec 11;11(3):e186461. doi: 10.1172/jci.insight.186461 (PMC12892887; doi:10.1172/jci.insight.186461)

## 1. Full unedited blot/gel for Figure 6c:

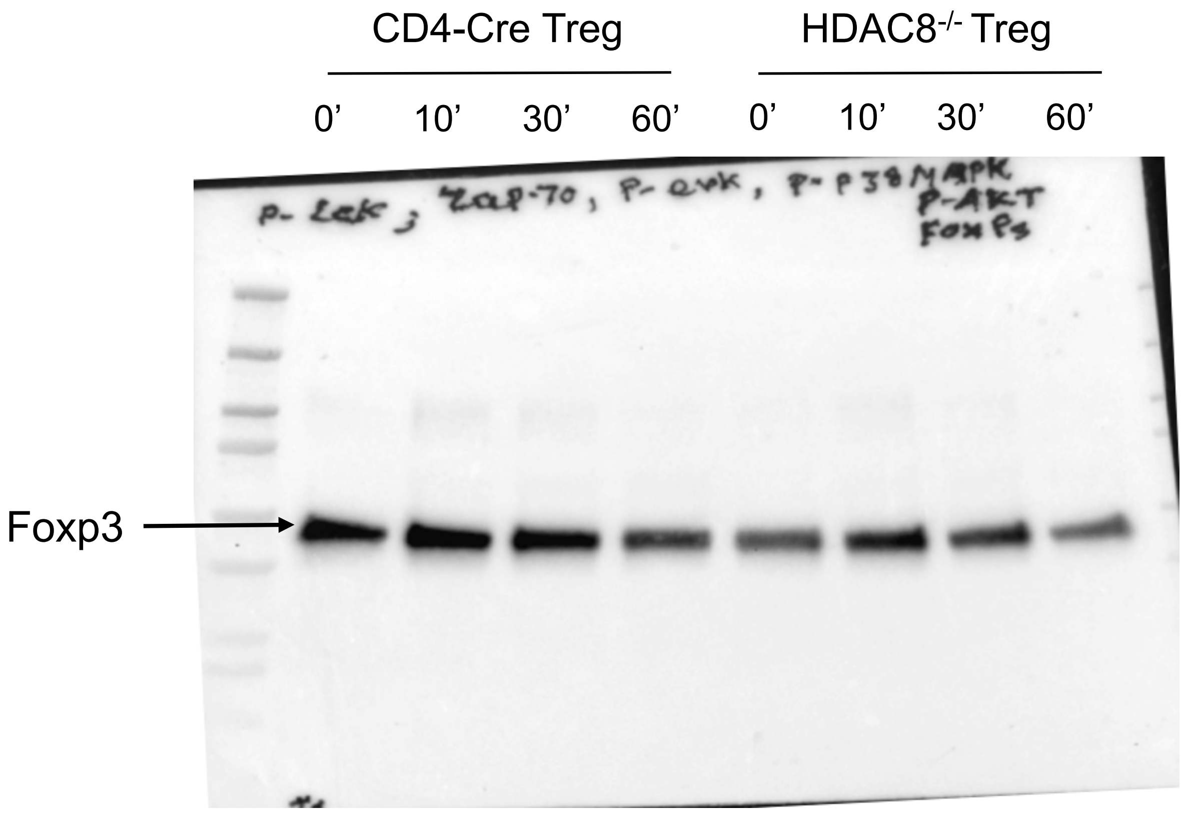


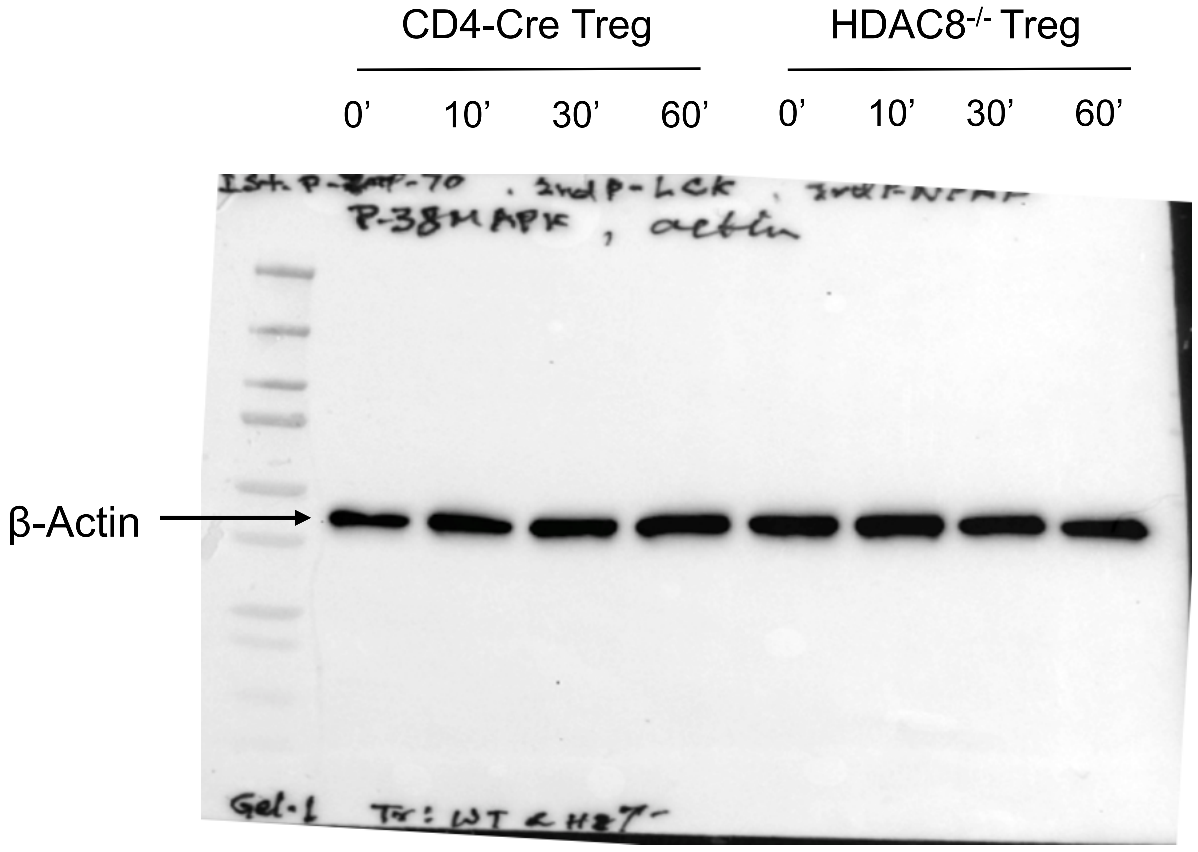


## 2. Full unedited blot/gel for Figure 7a:

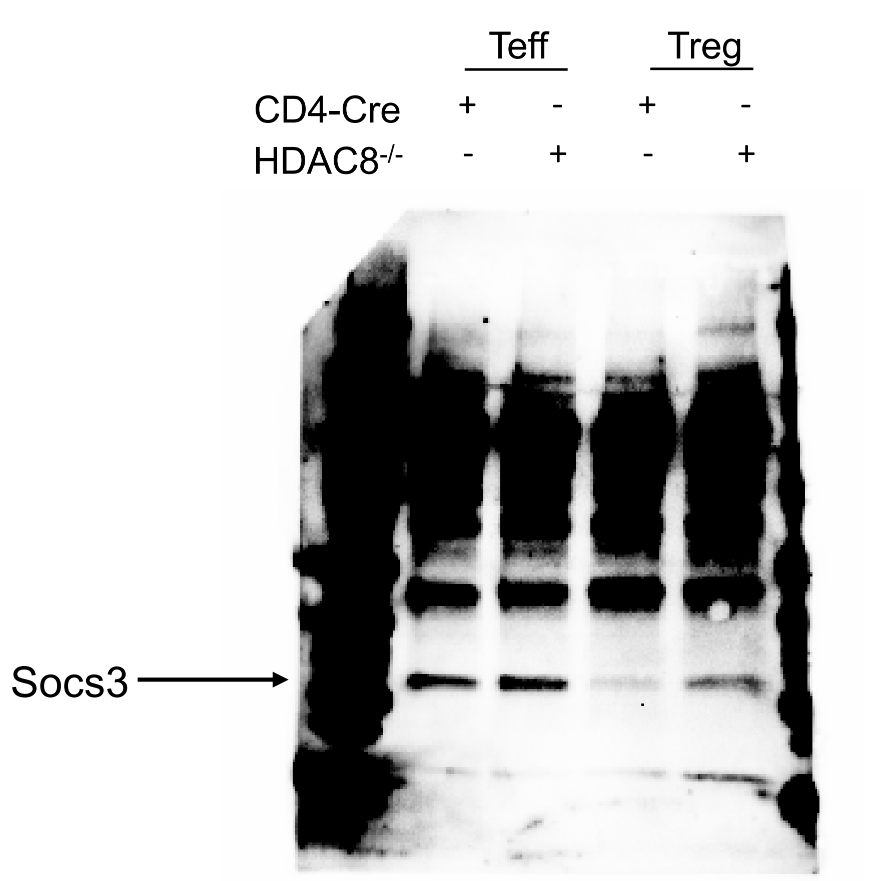


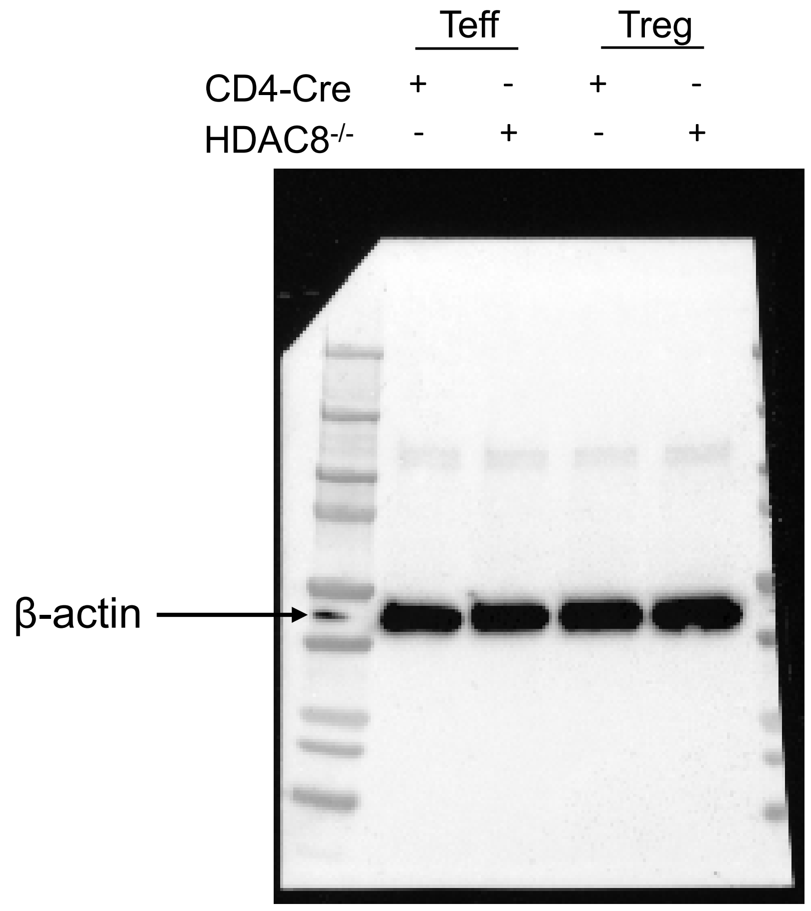


## 3. Full unedited blot/gel for Figure 7c:

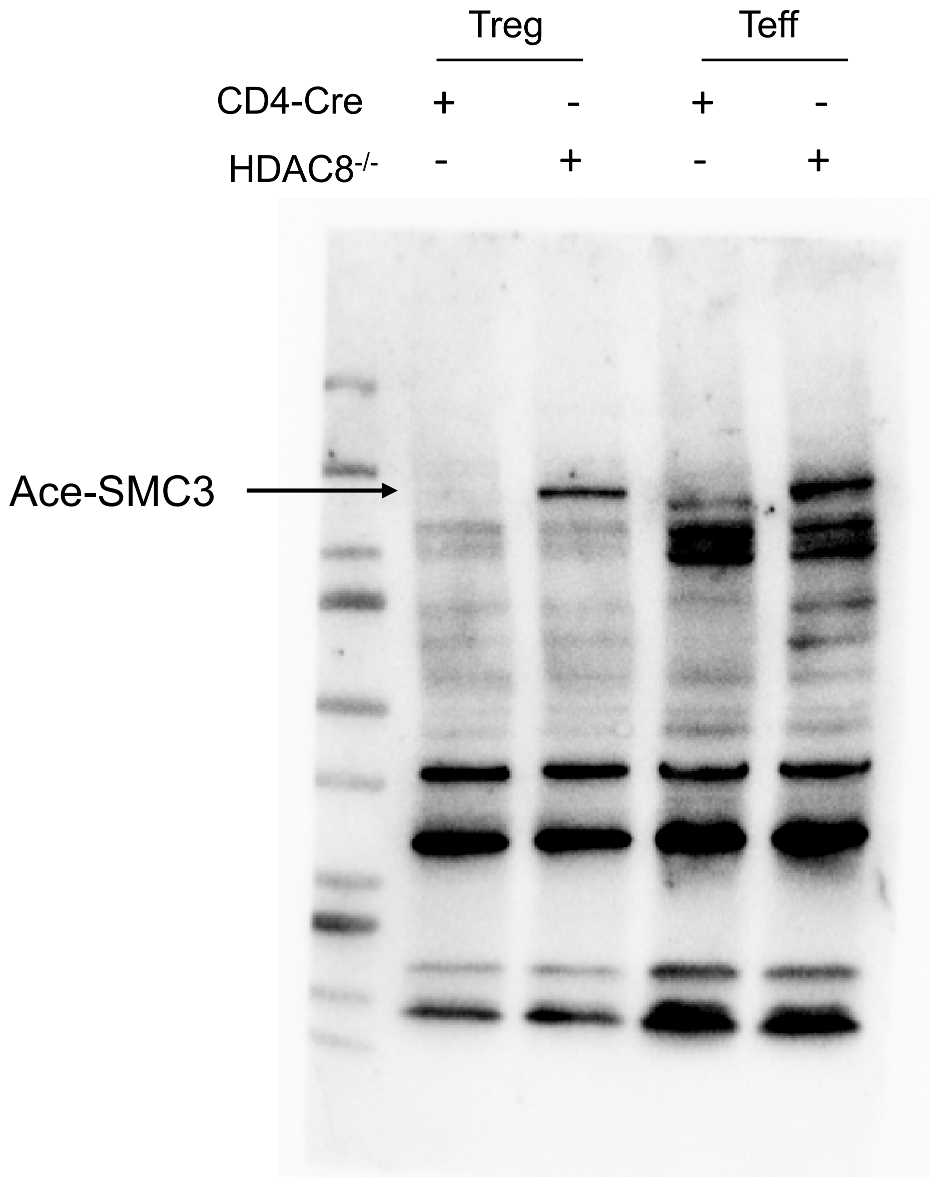


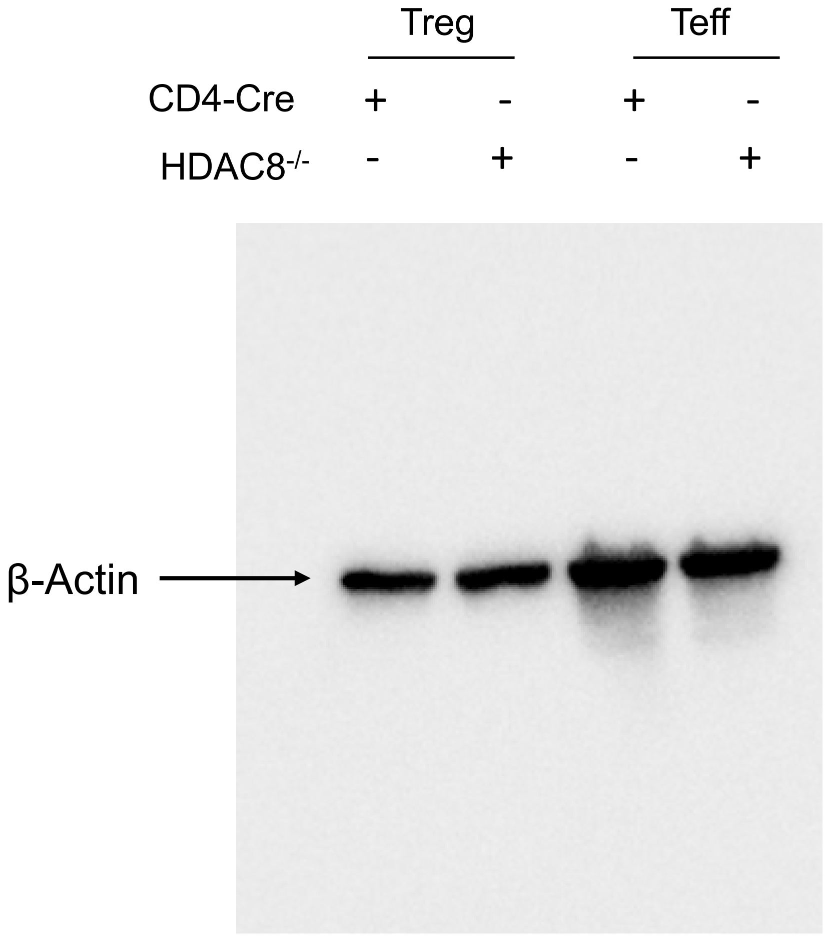

## 4. Full unedited blot/gel for Figure 7d:

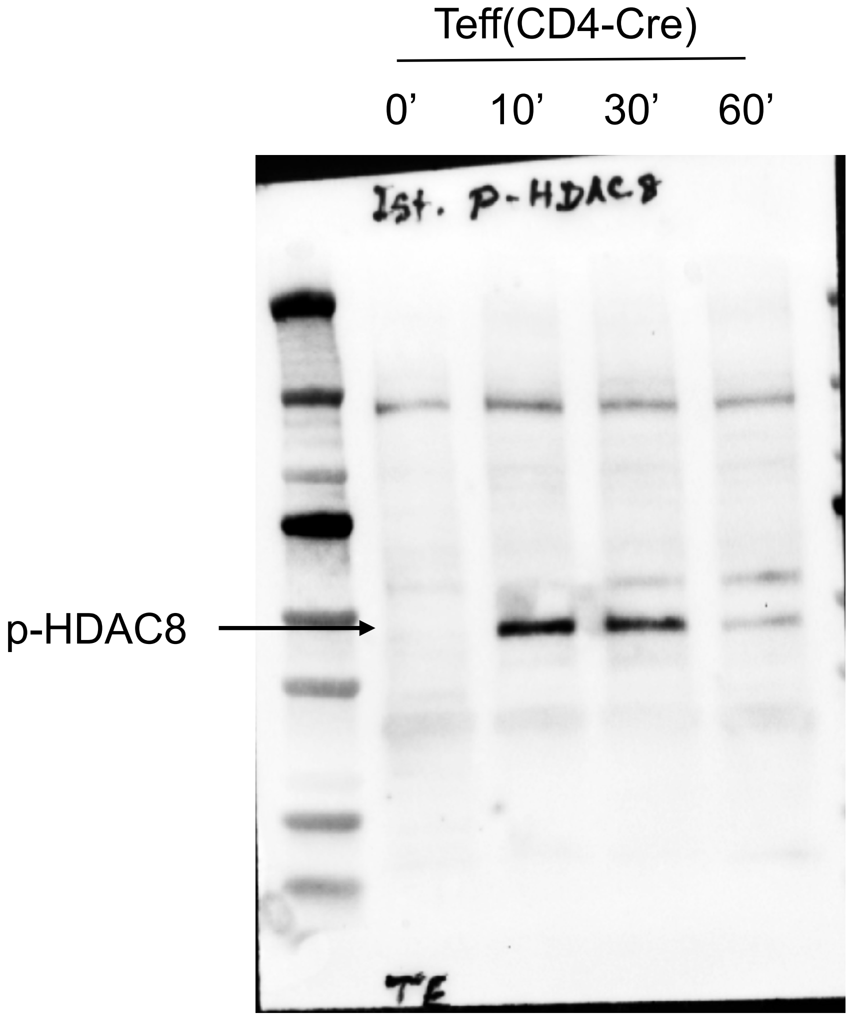


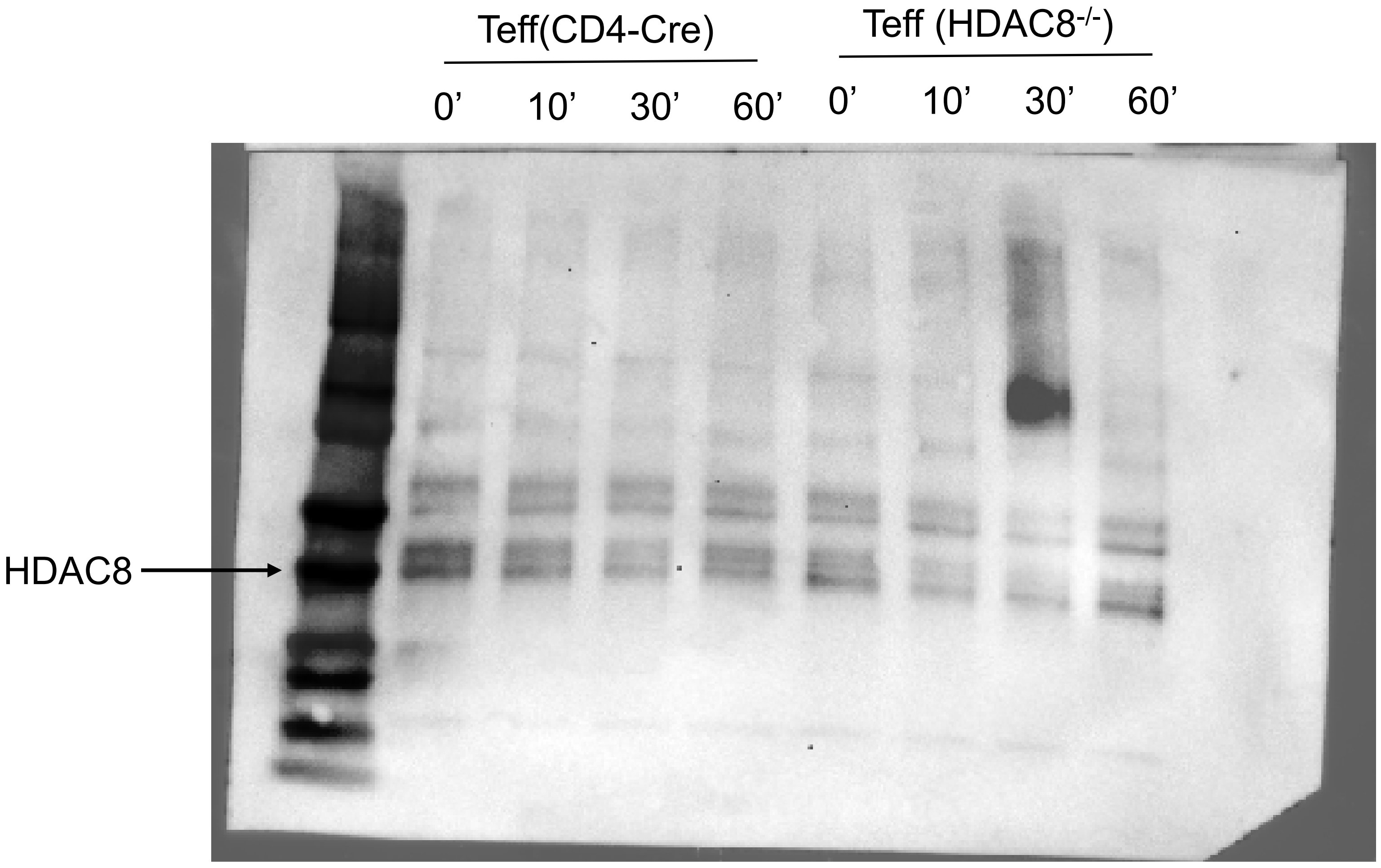


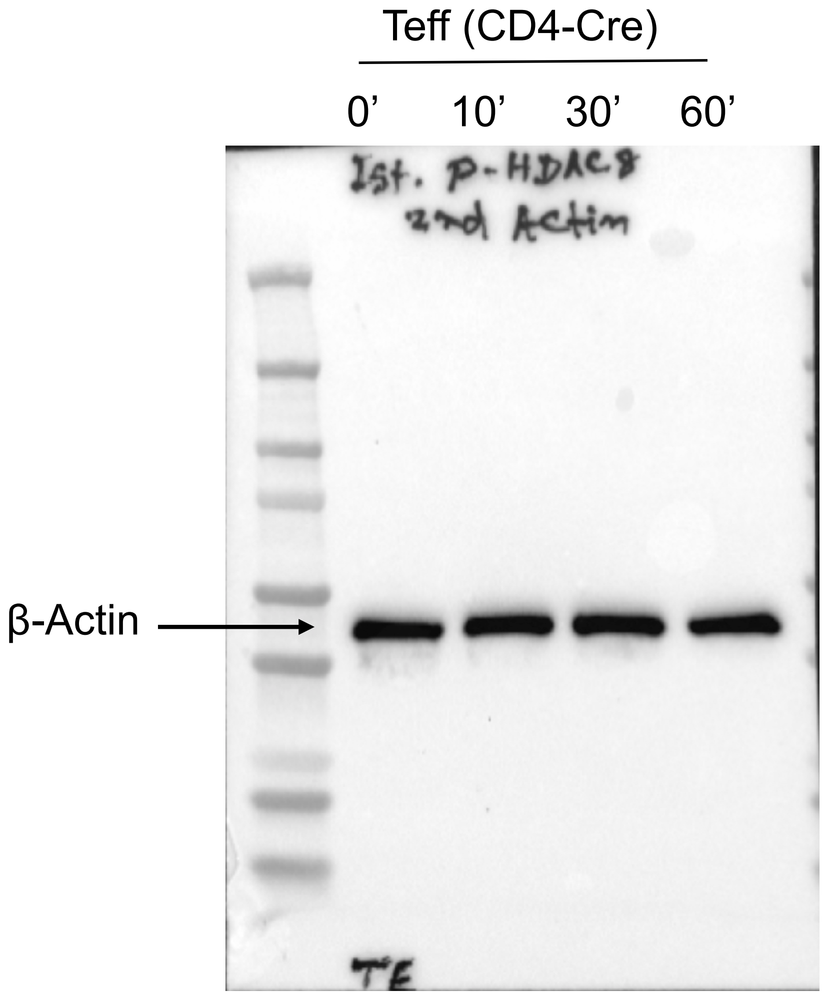


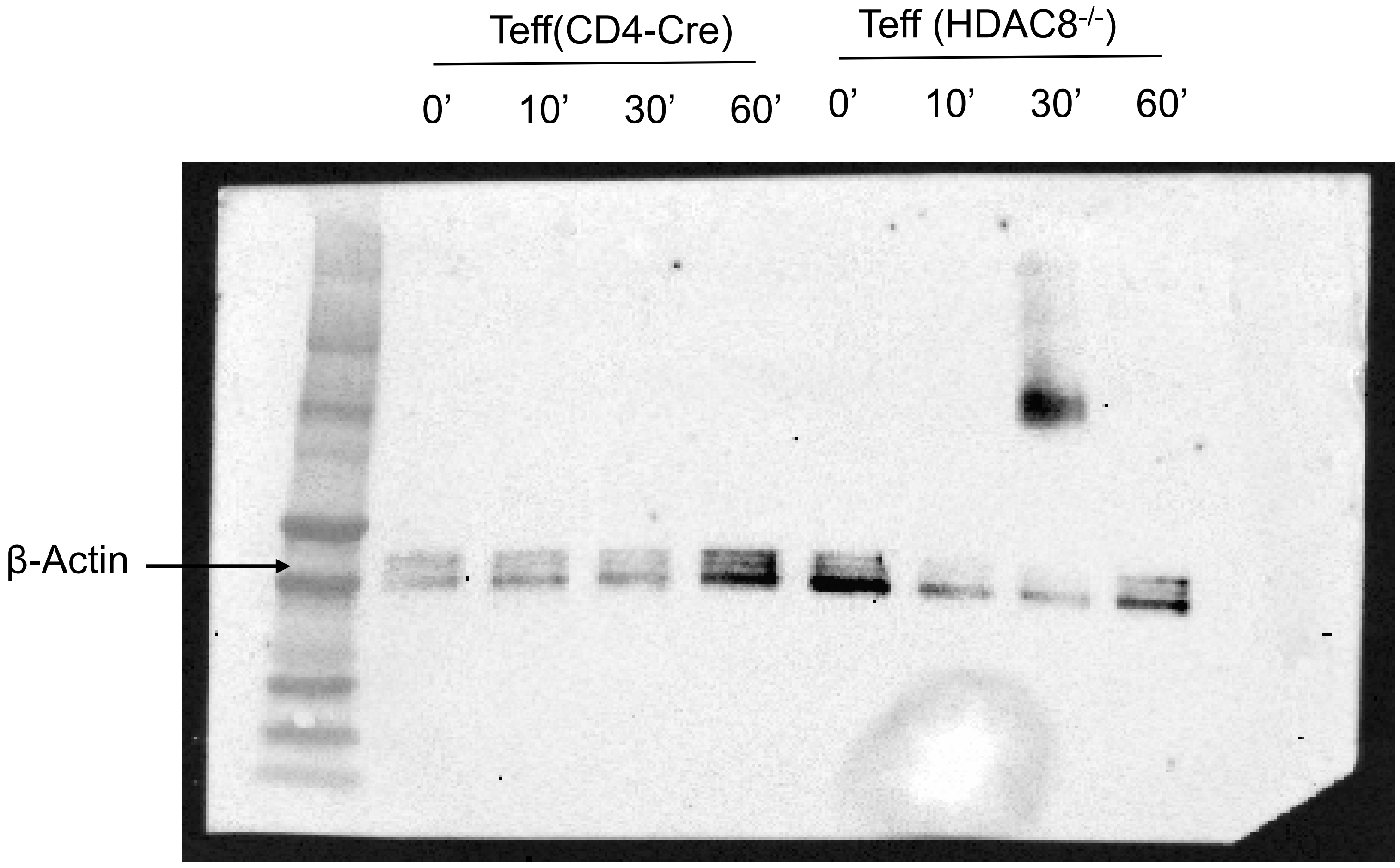

## 4. Full unedited blot/gel for Figure 7d:

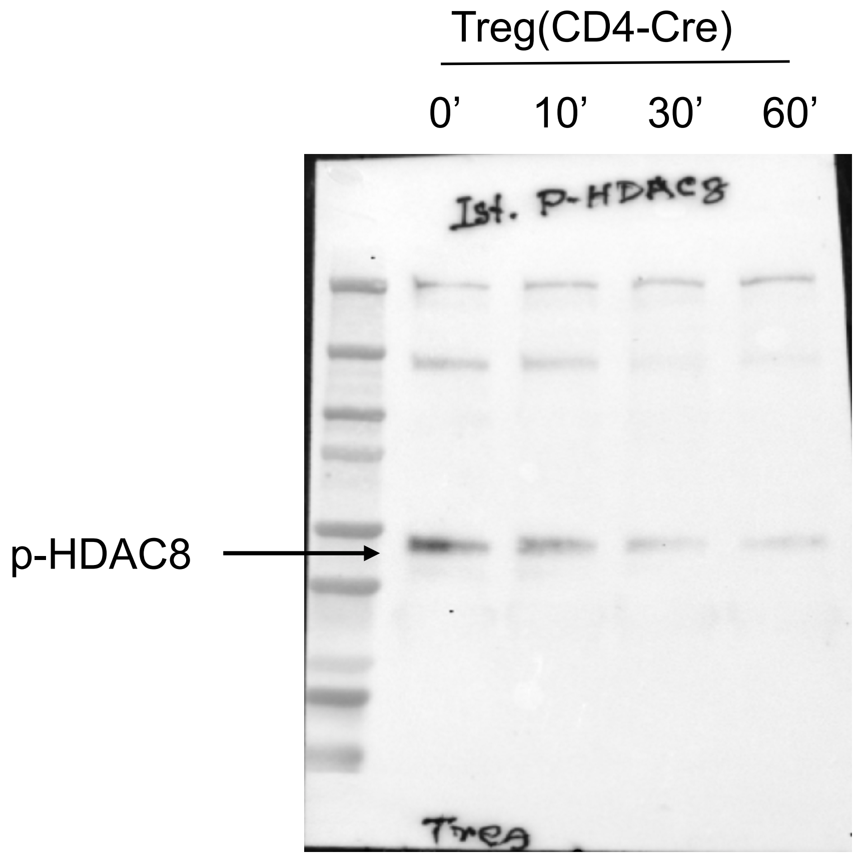


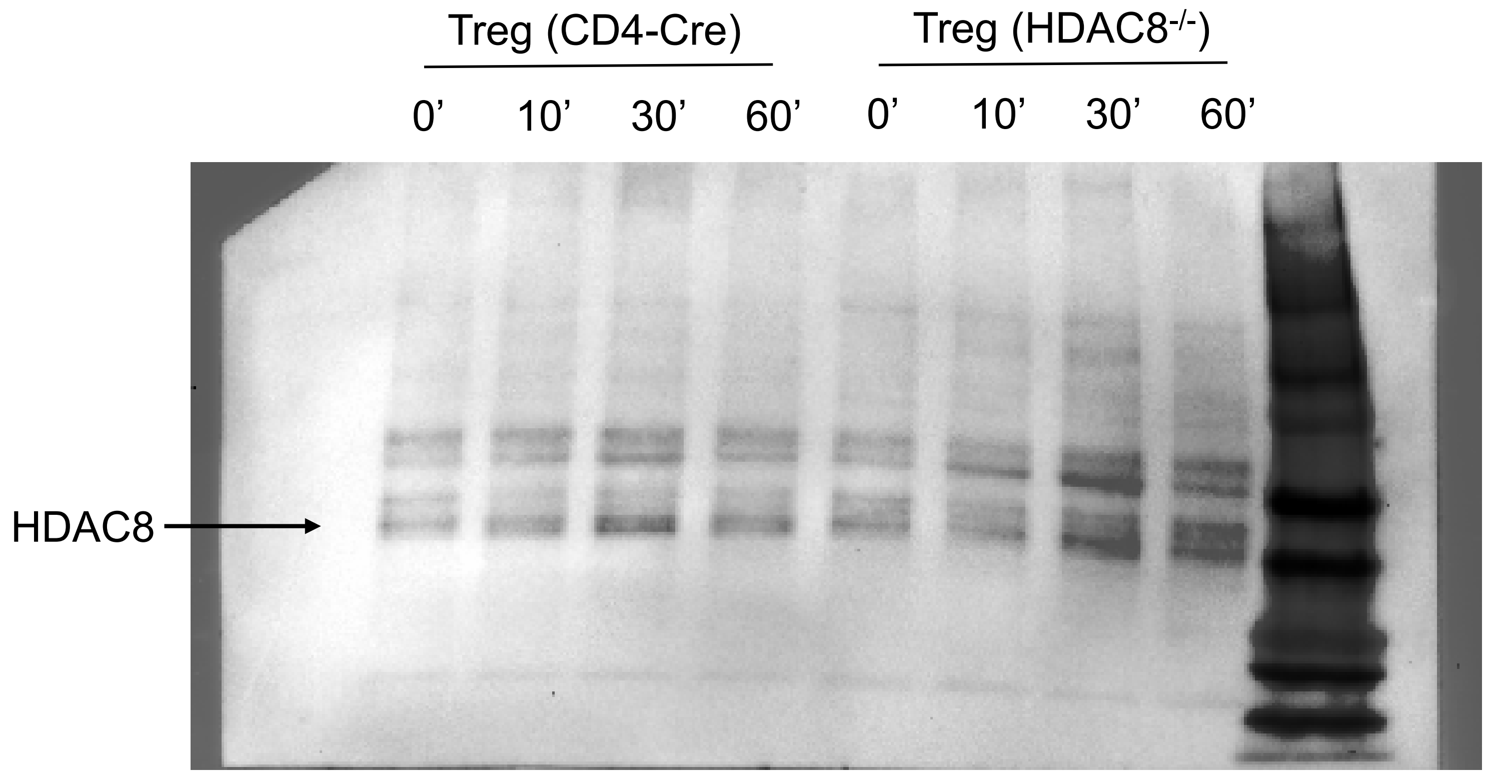


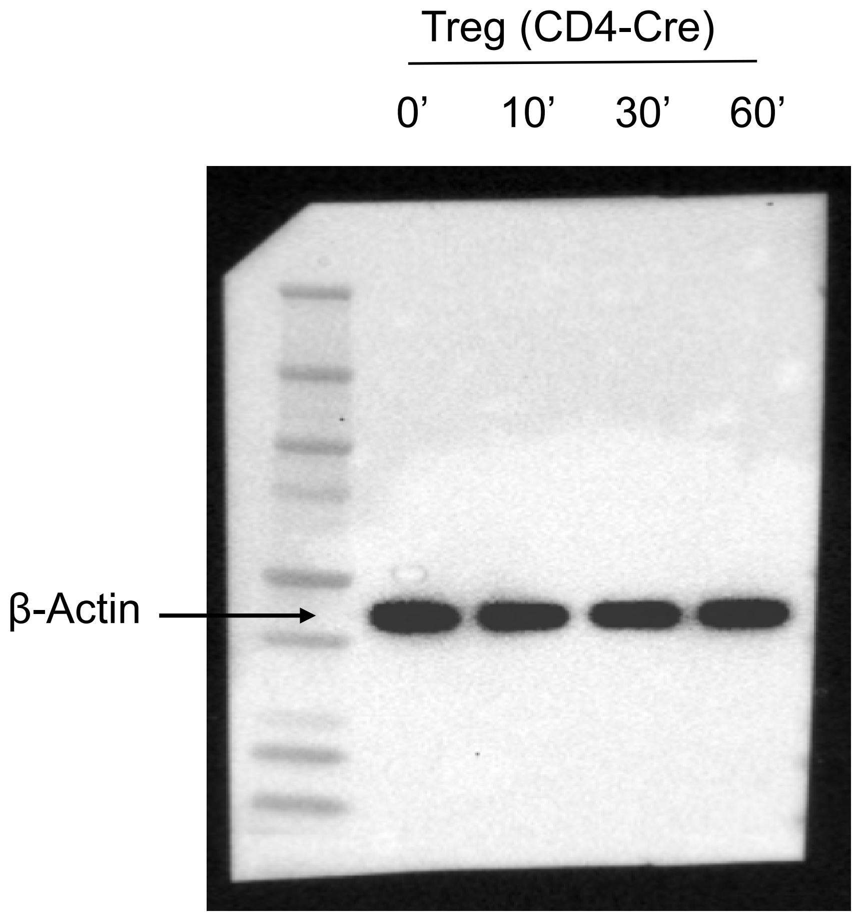


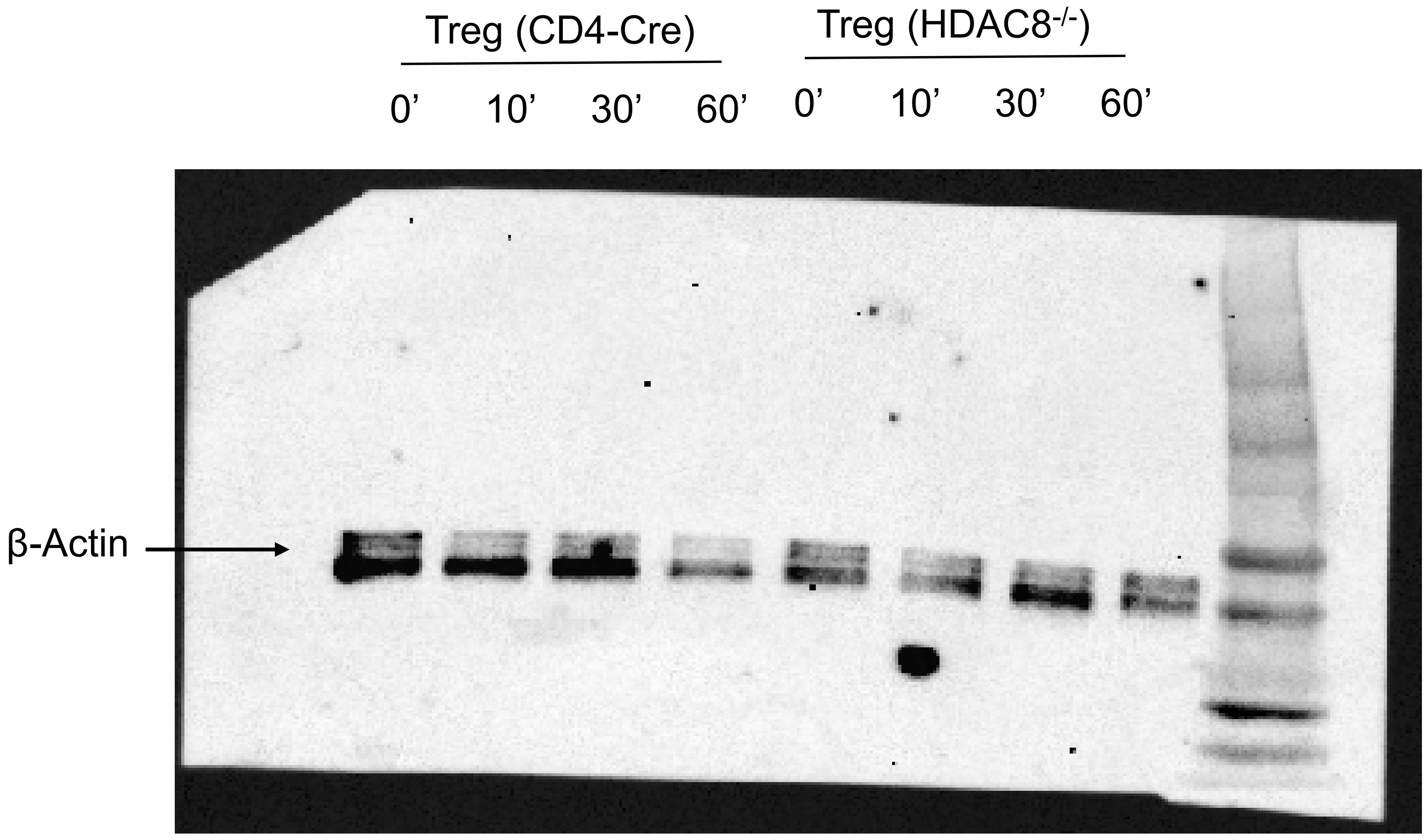

## 5. Full unedited blot/gel for Figure 7e:

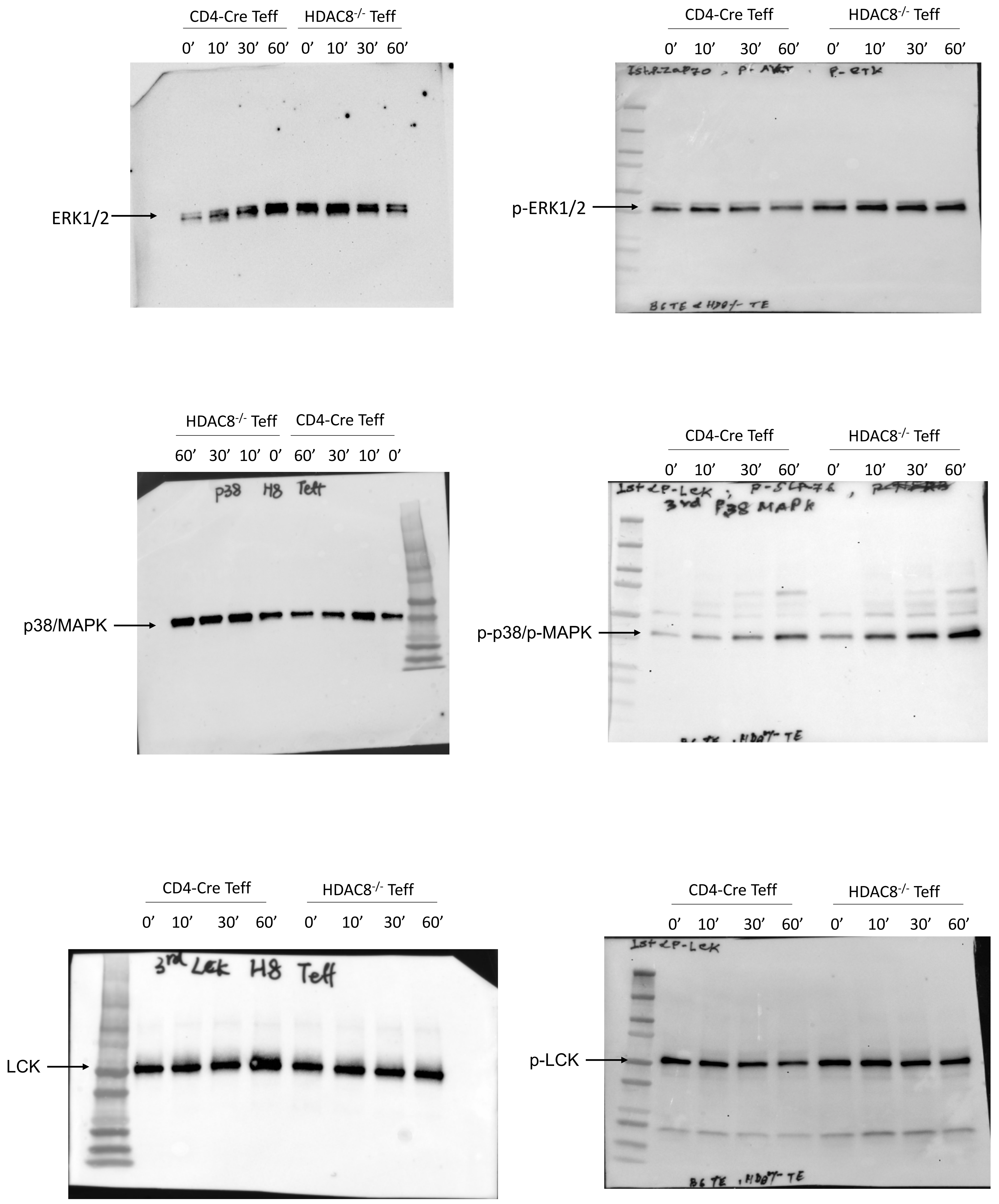

## 5. Full unedited blot/gel for Figure 7e:

CD4-Cre Teff | HDAC8$^{-/-}$ Teff

0' 10' 30' 60' 0' 10' 30' 60'

AKT →

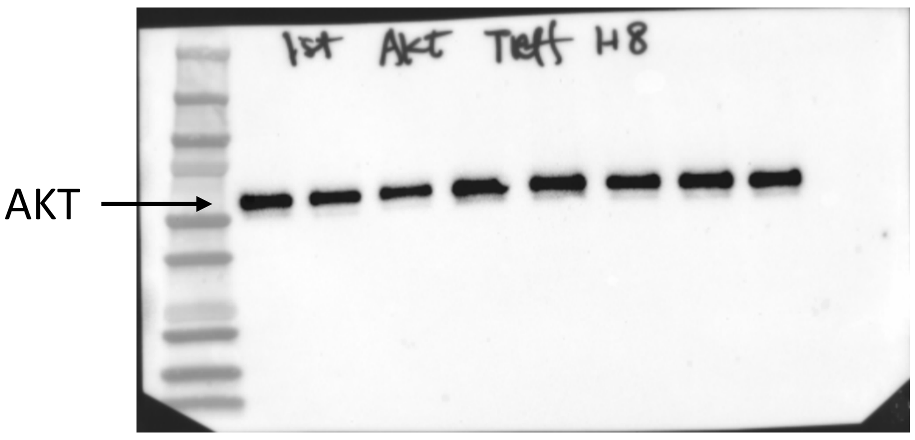

CD4-Cre Teff | HDAC8$^{-/-}$ Teff

0' 10' 30' 60' 0' 10' 30' 60'

p-AKT →

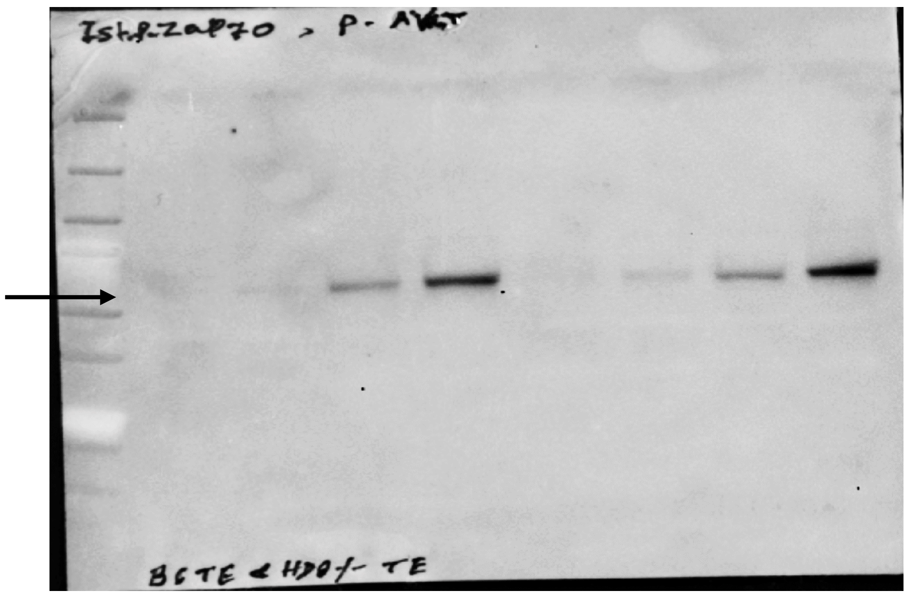

CD4-Cre Teff | HDAC8$^{-/-}$ Teff

0' 10' 30' 60' 0' 10' 30' 60'

Zap70 →

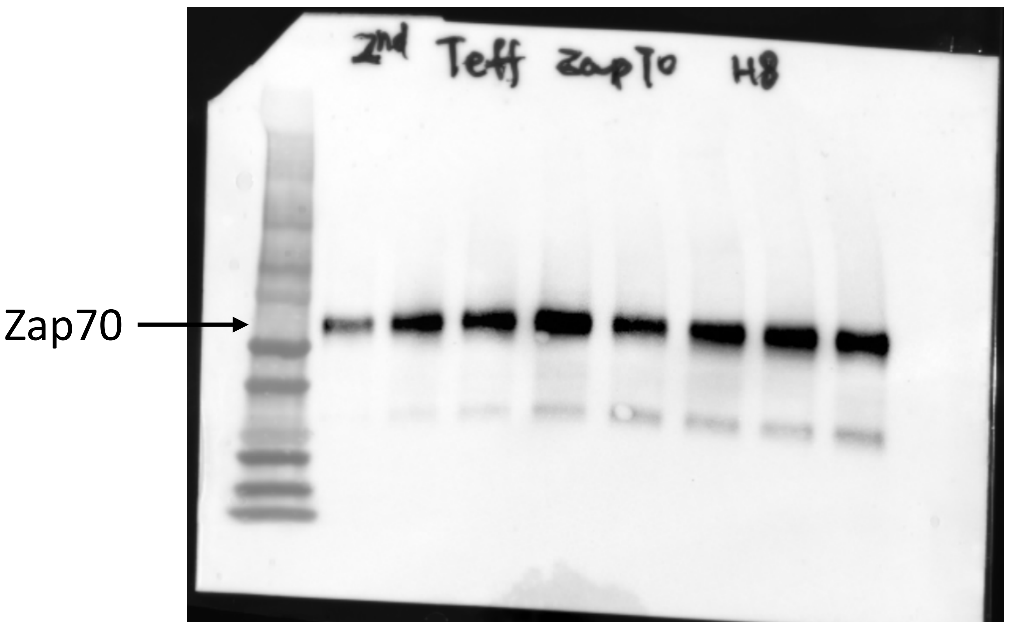

CD4-Cre Teff | HDAC8$^{-/-}$ Teff

0' 10' 30' 60' 0' 10' 30' 60'

p-Zap70 →

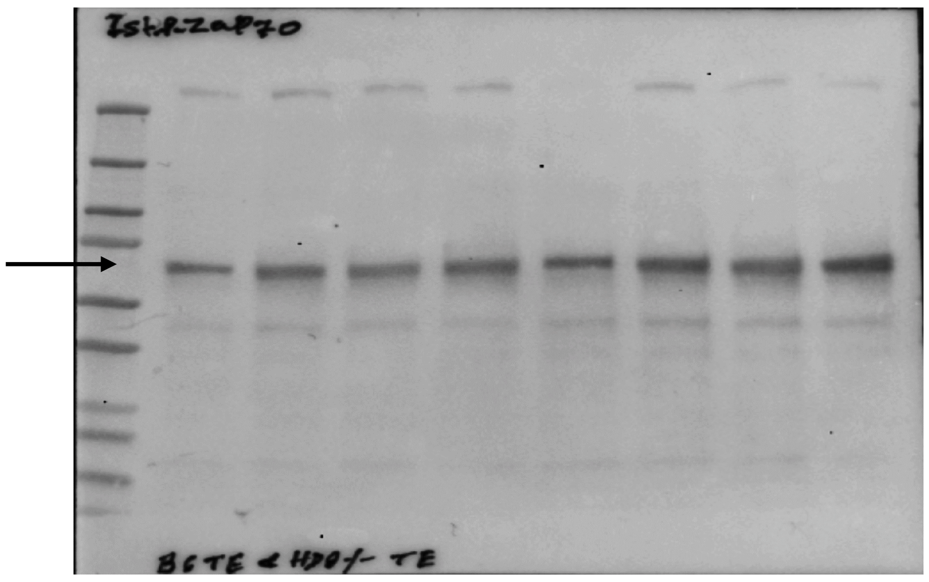

CD4-Cre Teff | HDAC8$^{-/-}$ Teff

0' 10' 30' 60' 0' 10' 30' 60'

SLP76 →

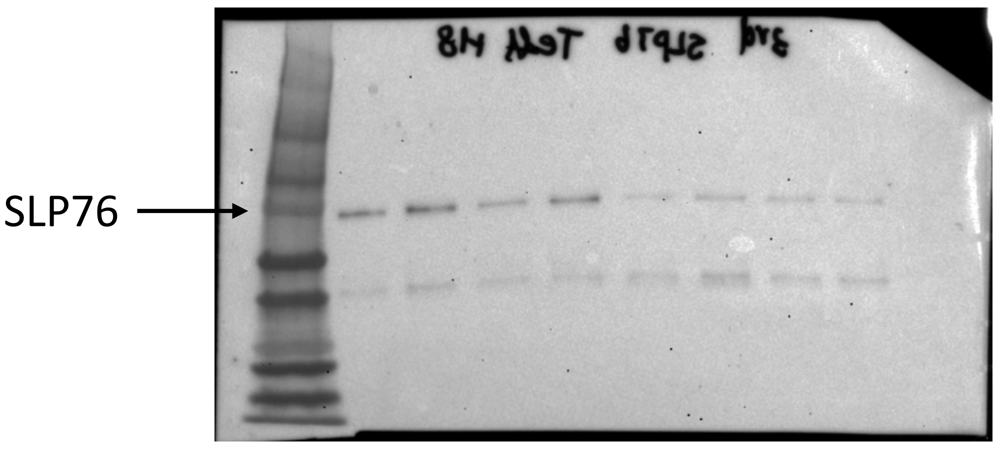

CD4-Cre Teff | HDAC8$^{-/-}$ Teff

0' 10' 30' 60' 0' 10' 30' 60'

p-SLP76 →

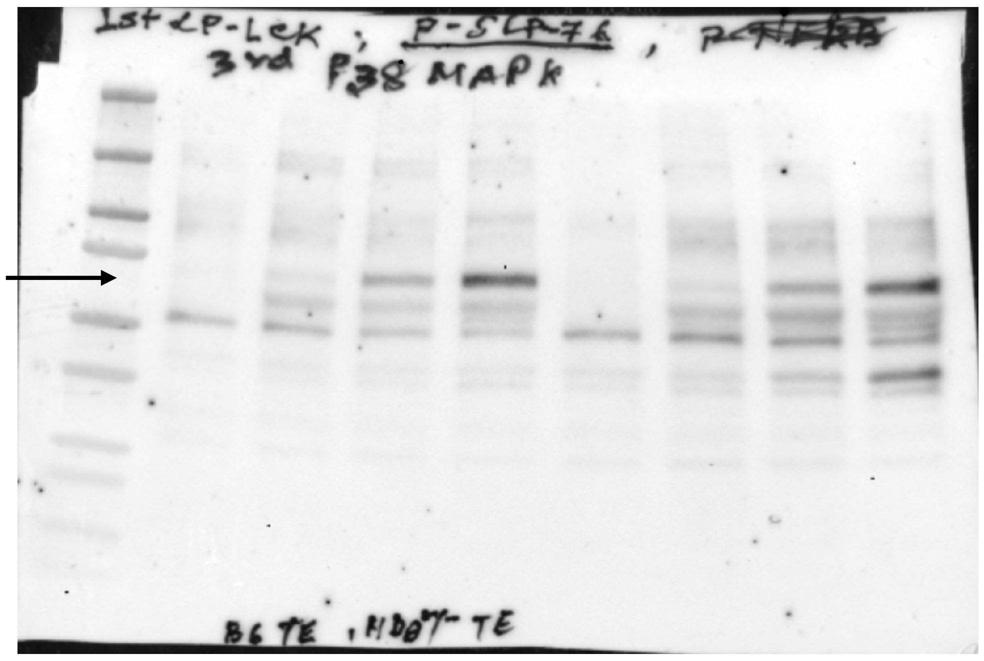

CD4-Cre Teff | HDAC8$^{-/-}$ Teff

0' 10' 30' 60' 0' 10' 30' 60'

β-Actin →

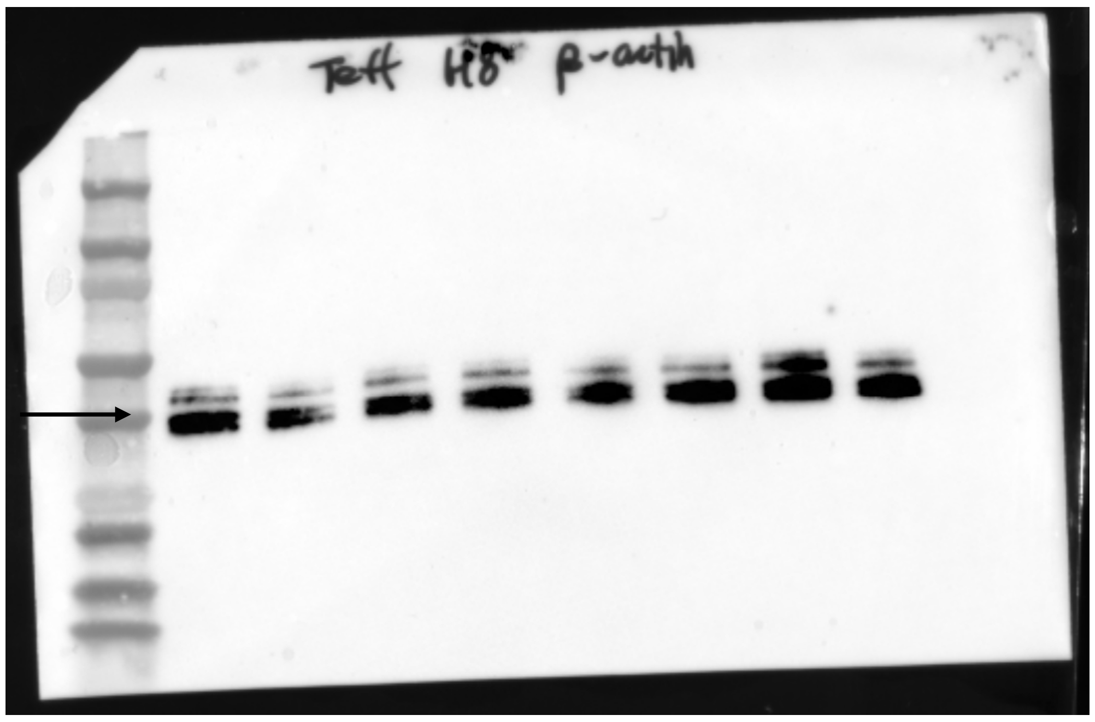

CD4-Cre Teff | HDAC8$^{-/-}$ Teff

0' 10' 30' 60' 0' 10' 30' 60'

β-Actin →

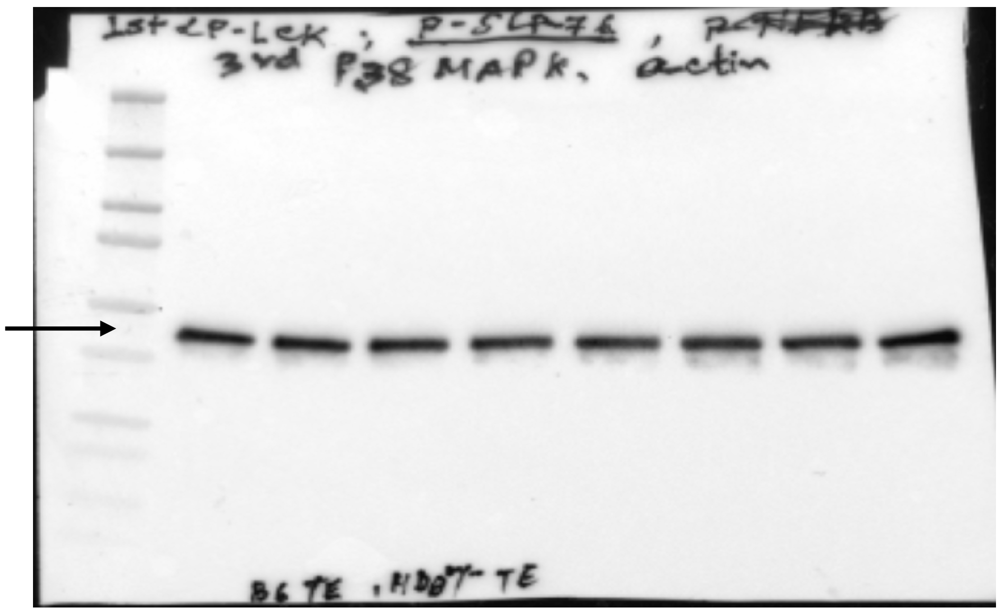

# 6. Full unedited blot/gel for Figure 7f:

CD4-Cre Treg | HDAC8$^{-/-}$ Treg

0’ 10’ 30’ 60’ 0’ 10’ 30’ 60’

ERK1/2

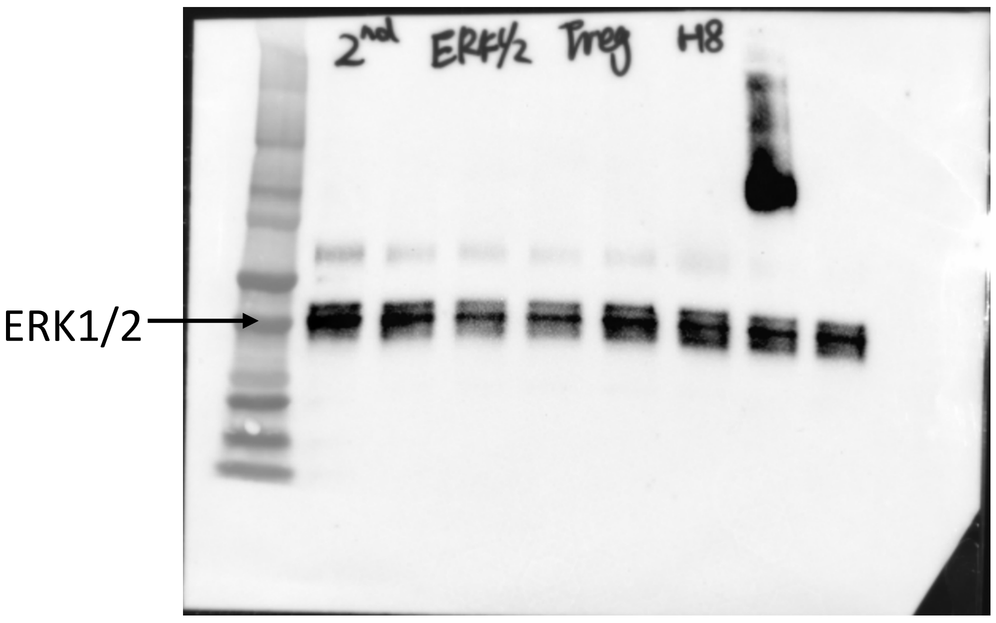


CD4-Cre Treg | HDAC8$^{-/-}$ Treg

0’ 10’ 30’ 60’ 0’ 10’ 30’ 60’

p-ERK1/2

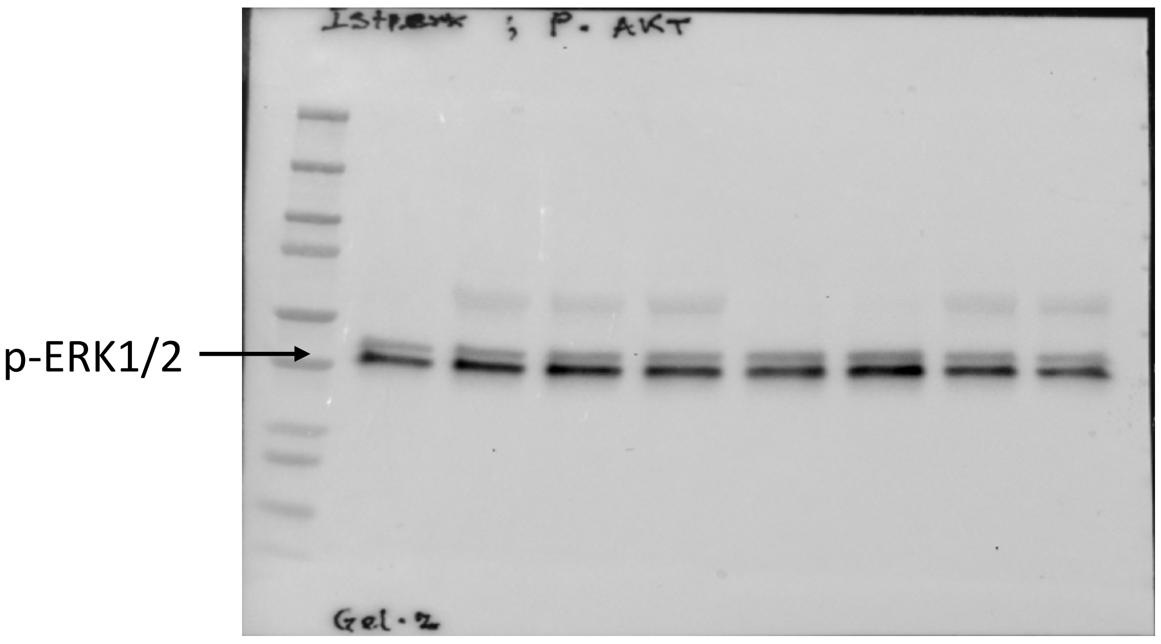


CD4-Cre Treg | HDAC8$^{-/-}$ Treg

0’ 10’ 30’ 60’ 0’ 10’ 30’ 60’

p38/MAPK

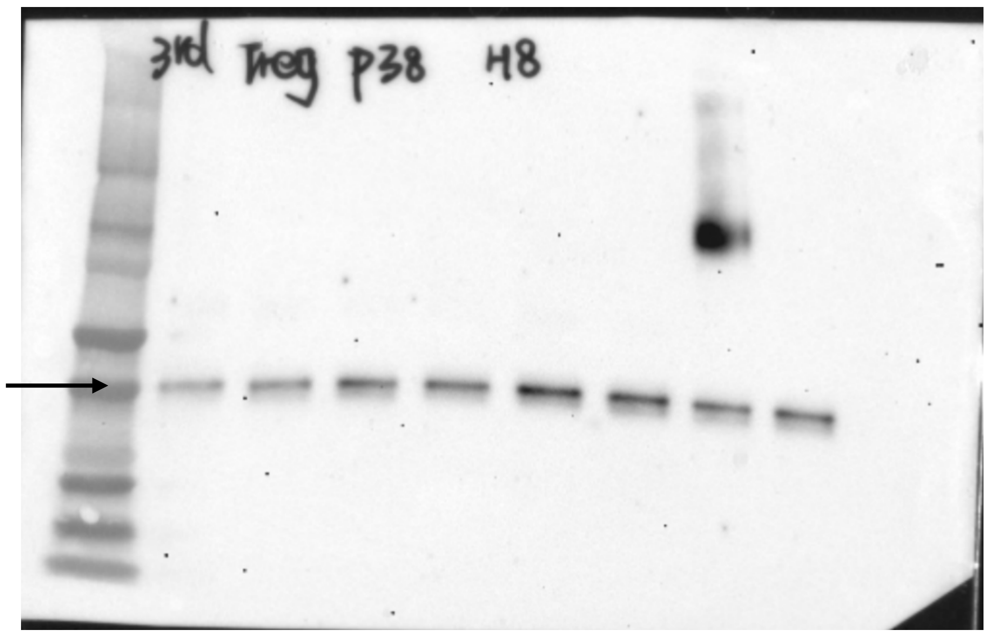


CD4-Cre Treg | HDAC8$^{-/-}$ Treg

0’ 10’ 30’ 60’ 0’ 10’ 30’ 60’

p-p38/p-MAPK

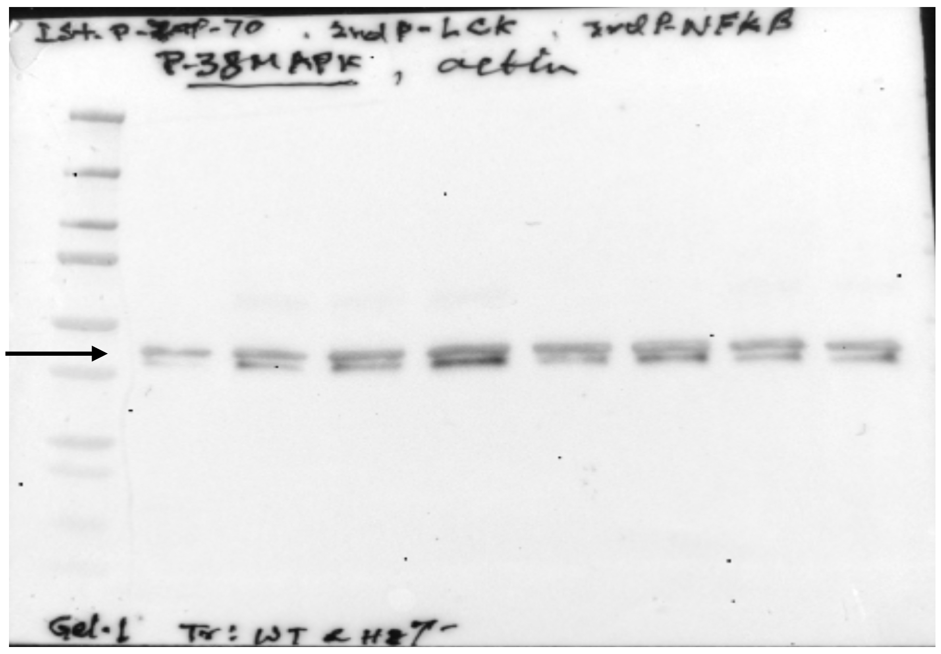


CD4-Cre Treg | HDAC8$^{-/-}$ Treg

0’ 10’ 30’ 60’ 0’ 10’ 30’ 60’

LCK

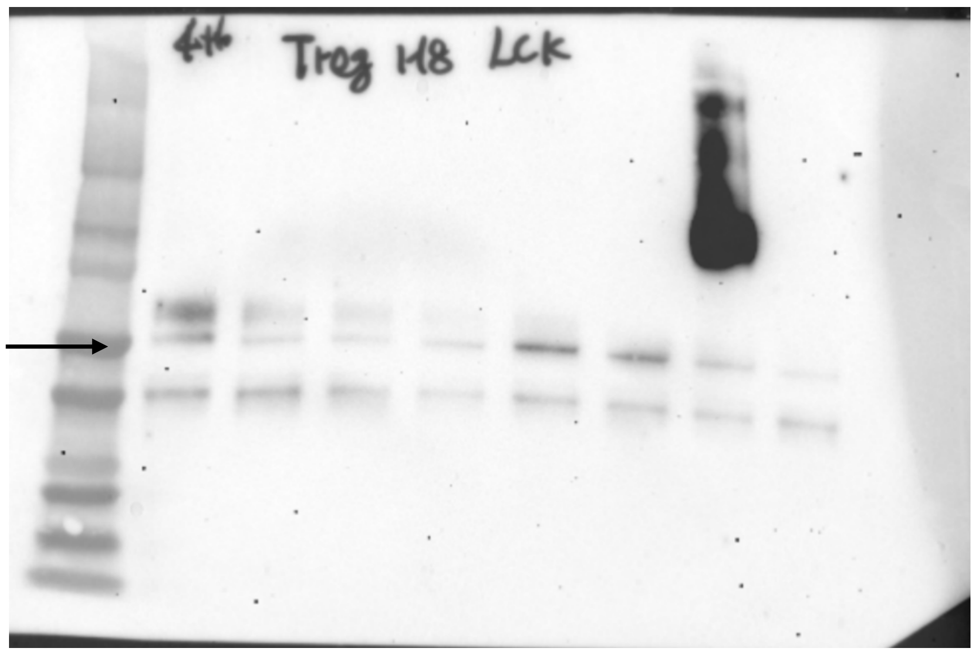


CD4-Cre Treg | HDAC8$^{-/-}$ Treg

0’ 10’ 30’ 60’ 0’ 10’ 30’ 60’

p-LCK

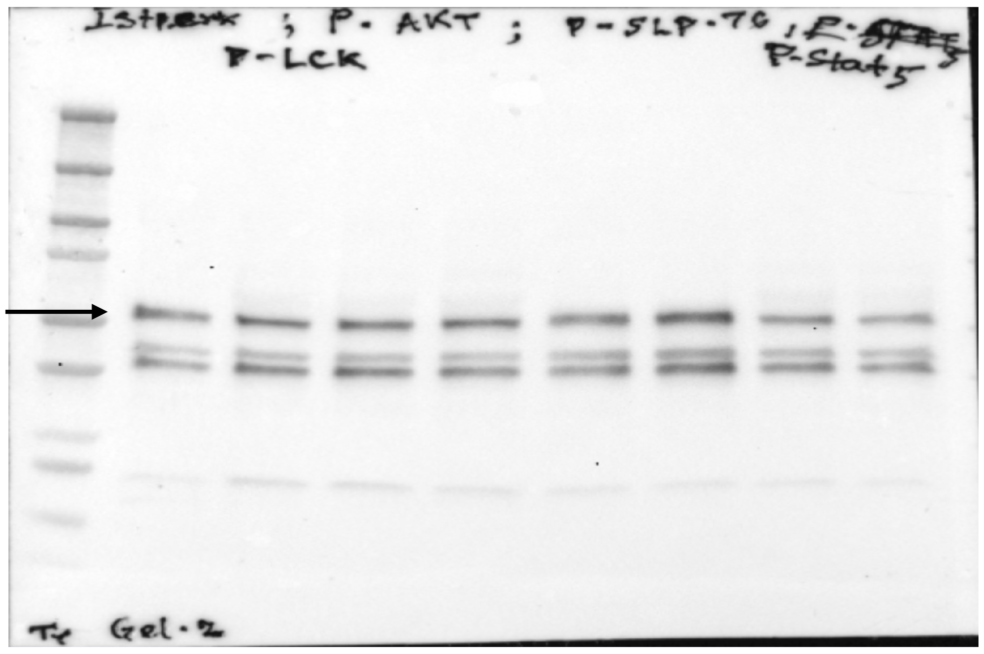

## 6. Full unedited blot/gel for Figure 7f:

CD4-Cre Treg | HDAC8$^{-/-}$ Treg

0’ 10’ 30’ 60’ 0’ 10’ 30’ 60’

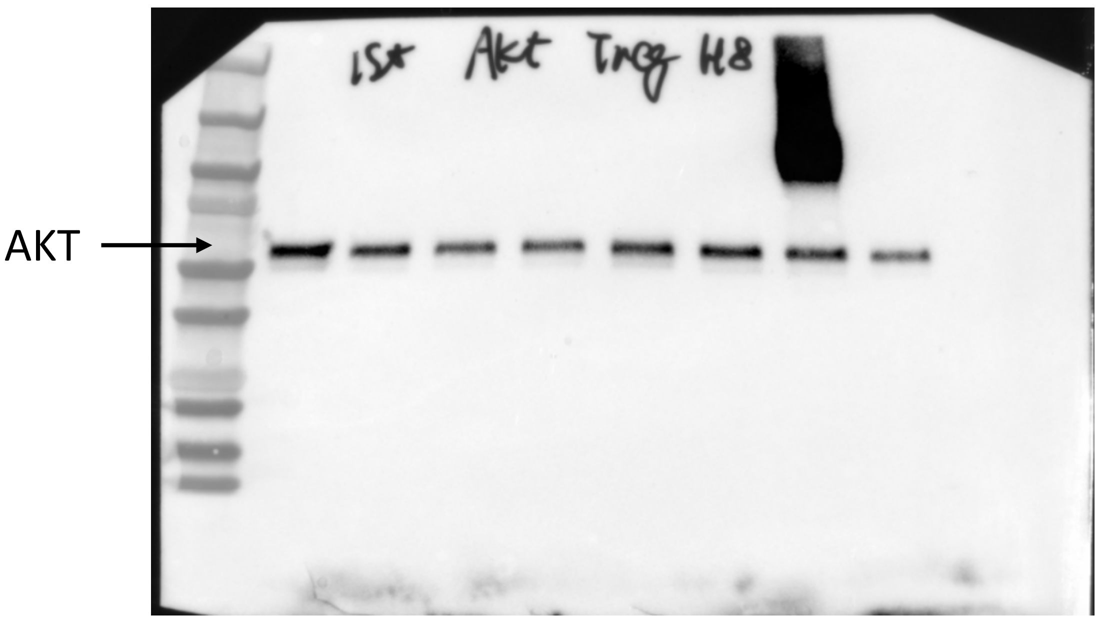


AKT

CD4-Cre Treg | HDAC8$^{-/-}$ Treg

0’ 10’ 30’ 60’ 0’ 10’ 30’ 60’

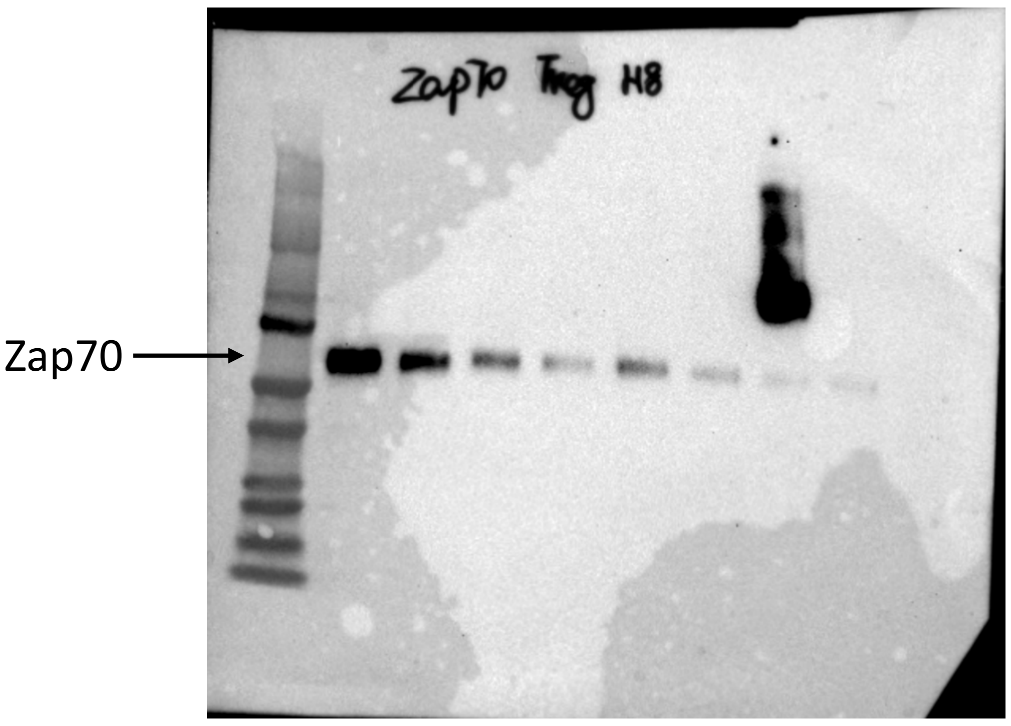


Zap70

CD4-Cre Treg | HDAC8$^{-/-}$ Treg

0’ 10’ 30’ 60’ 0’ 10’ 30’ 60’

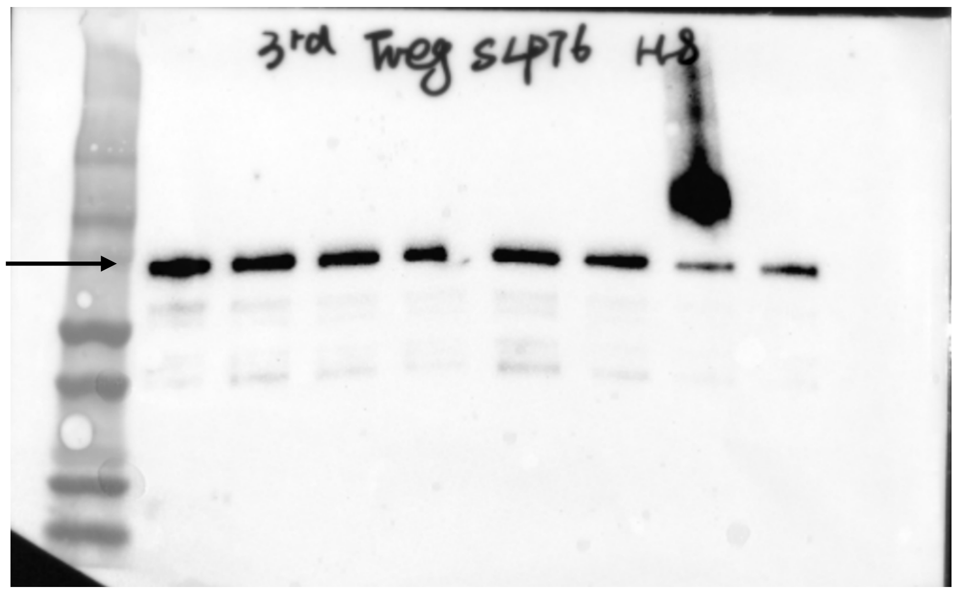


SLP76

CD4-Cre Treg | HDAC8$^{-/-}$ Treg

0’ 10’ 30’ 60’ 0’ 10’ 30’ 60’

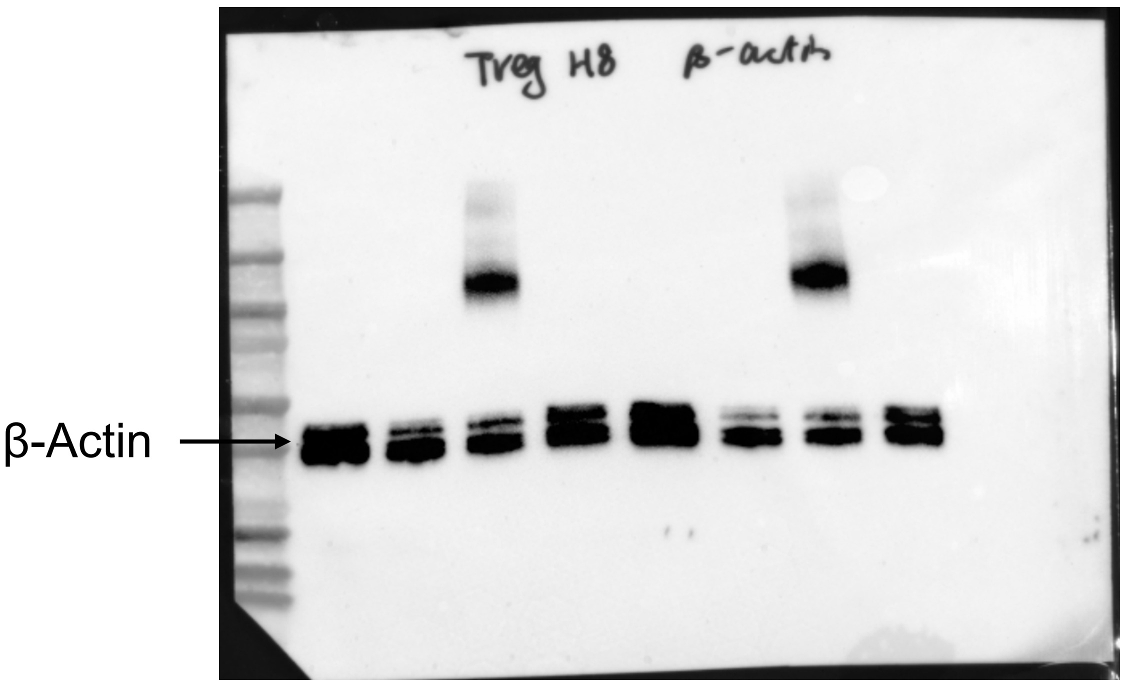


β-Actin

CD4-Cre Treg | HDAC8$^{-/-}$ Treg

0’ 10’ 30’ 60’ 0’ 10’ 30’ 60’

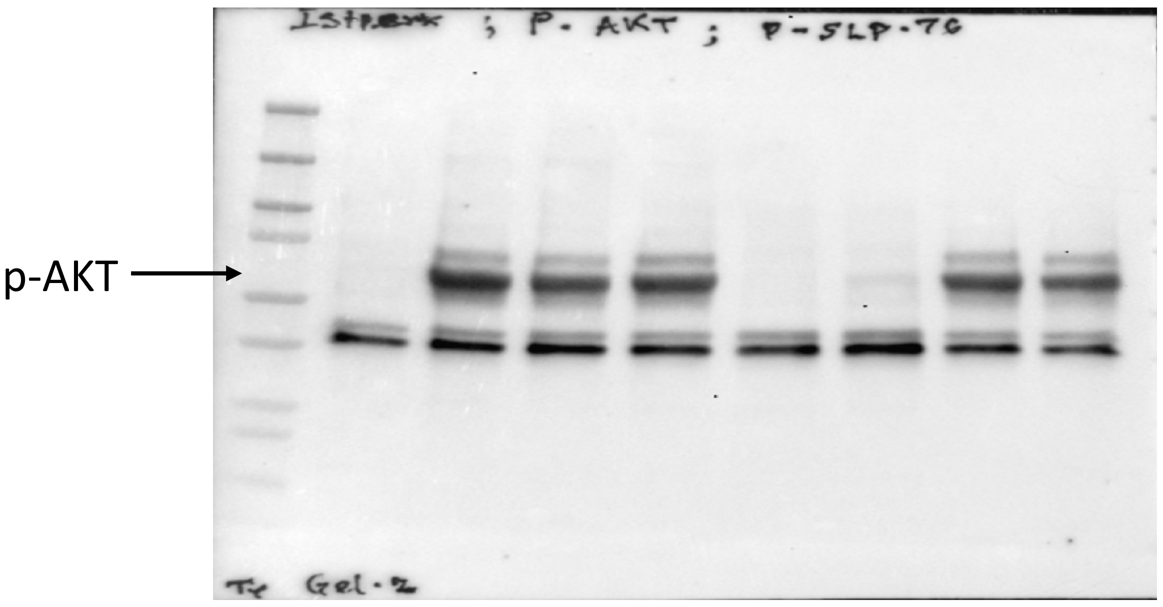


p-AKT

CD4-Cre Treg | HDAC8$^{-/-}$ Treg

0’ 10’ 30’ 60’ 0’ 10’ 30’ 60’

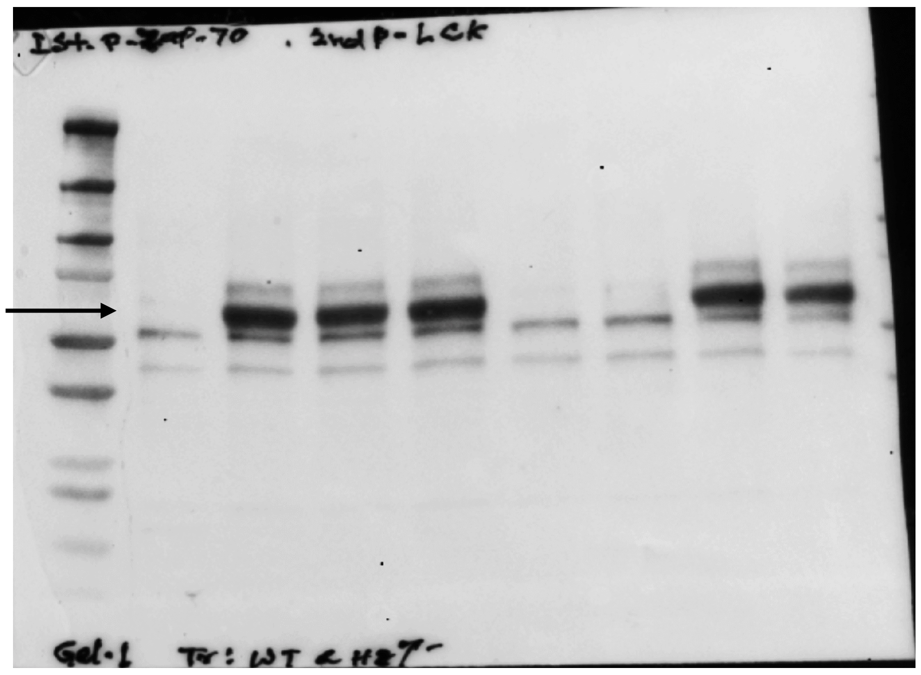


p-Zap70

CD4-Cre Treg | HDAC8$^{-/-}$ Treg

0’ 10’ 30’ 60’ 0’ 10’ 30’ 60’

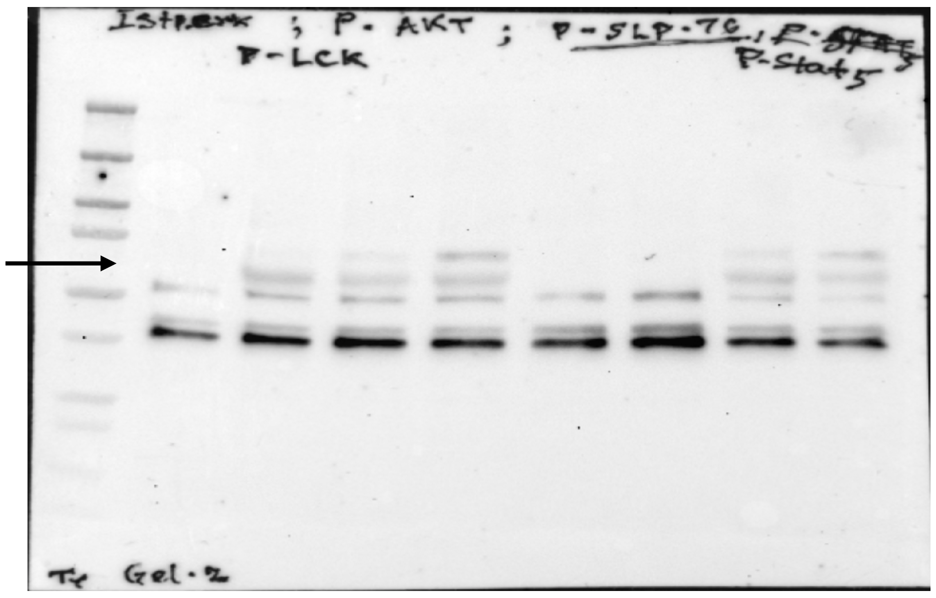


p-SLP76

CD4-Cre Treg | HDAC8$^{-/-}$ Treg

0’ 10’ 30’ 60’ 0’ 10’ 30’ 60’

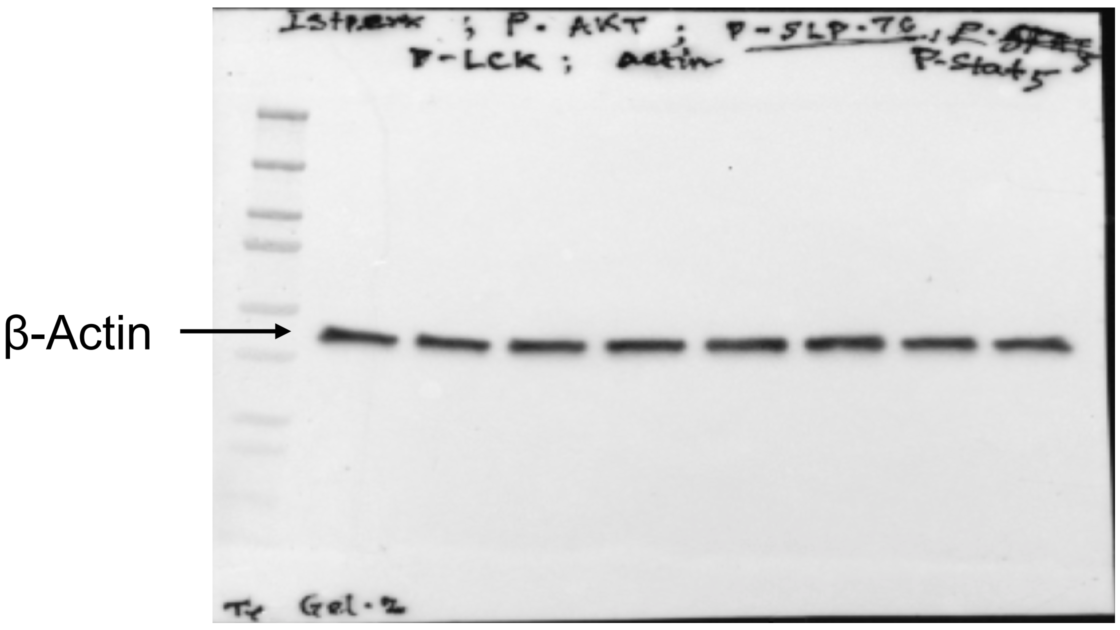


β-Actin

## 7. Full unedited blot/gel for Figure S17:

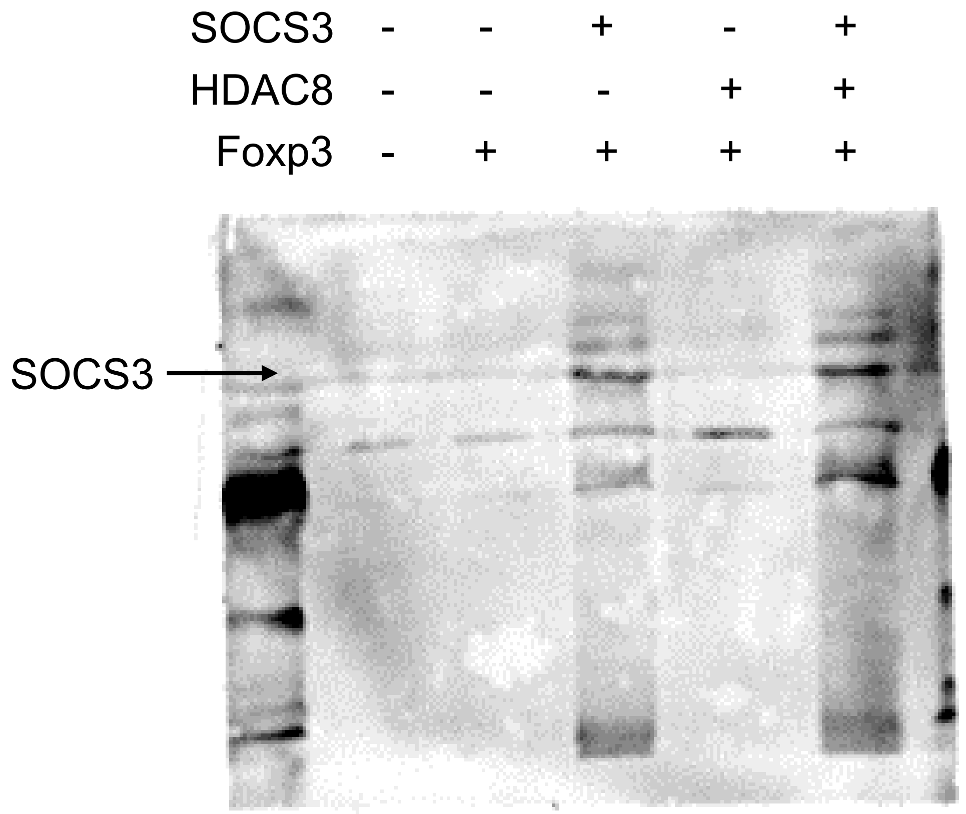


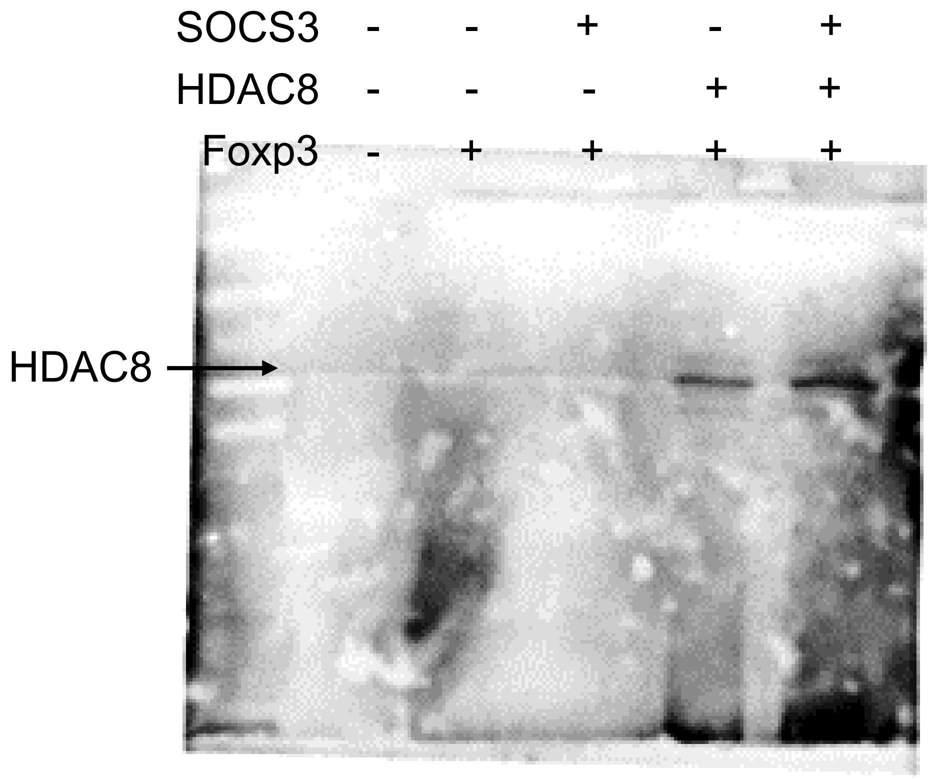


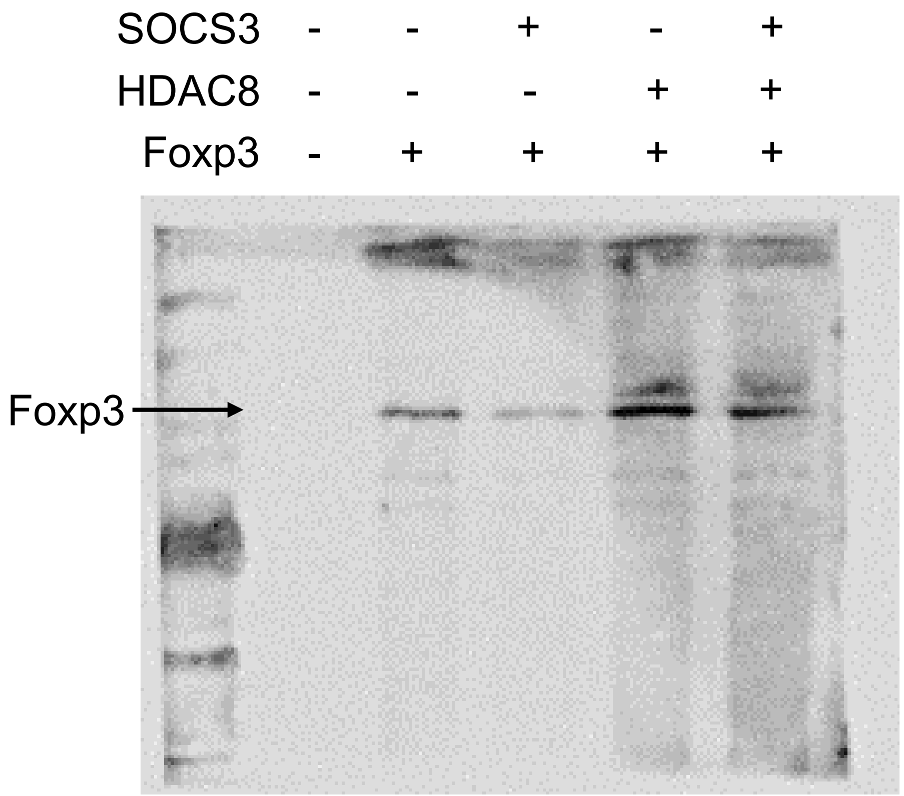


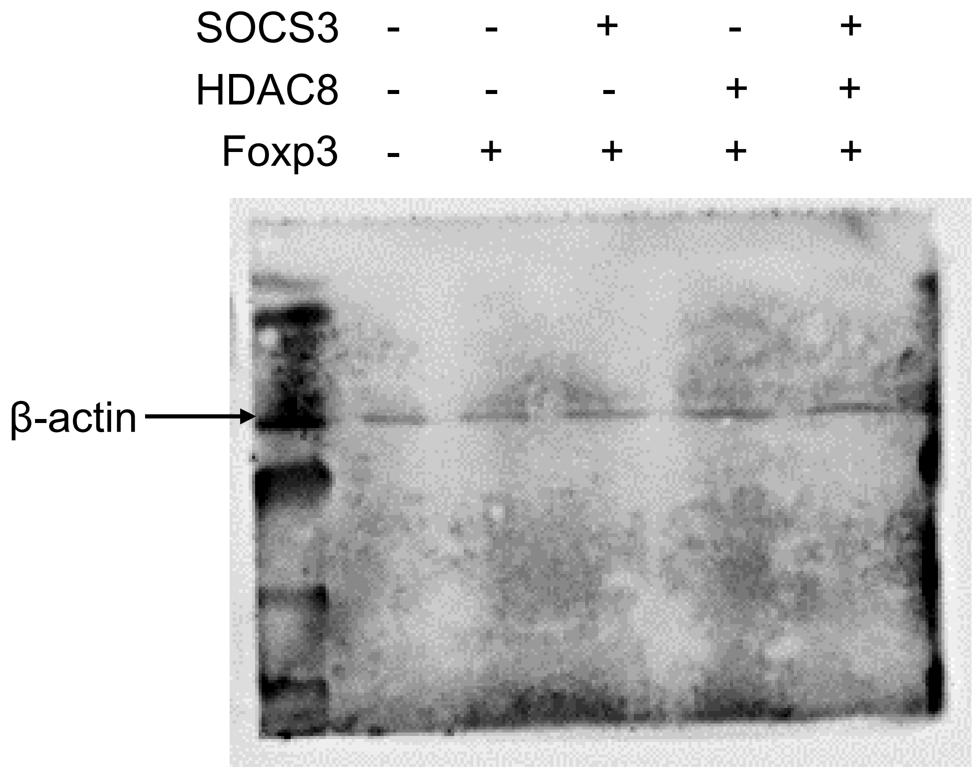

## 8. Full unedited blot/gel for Figure S18:

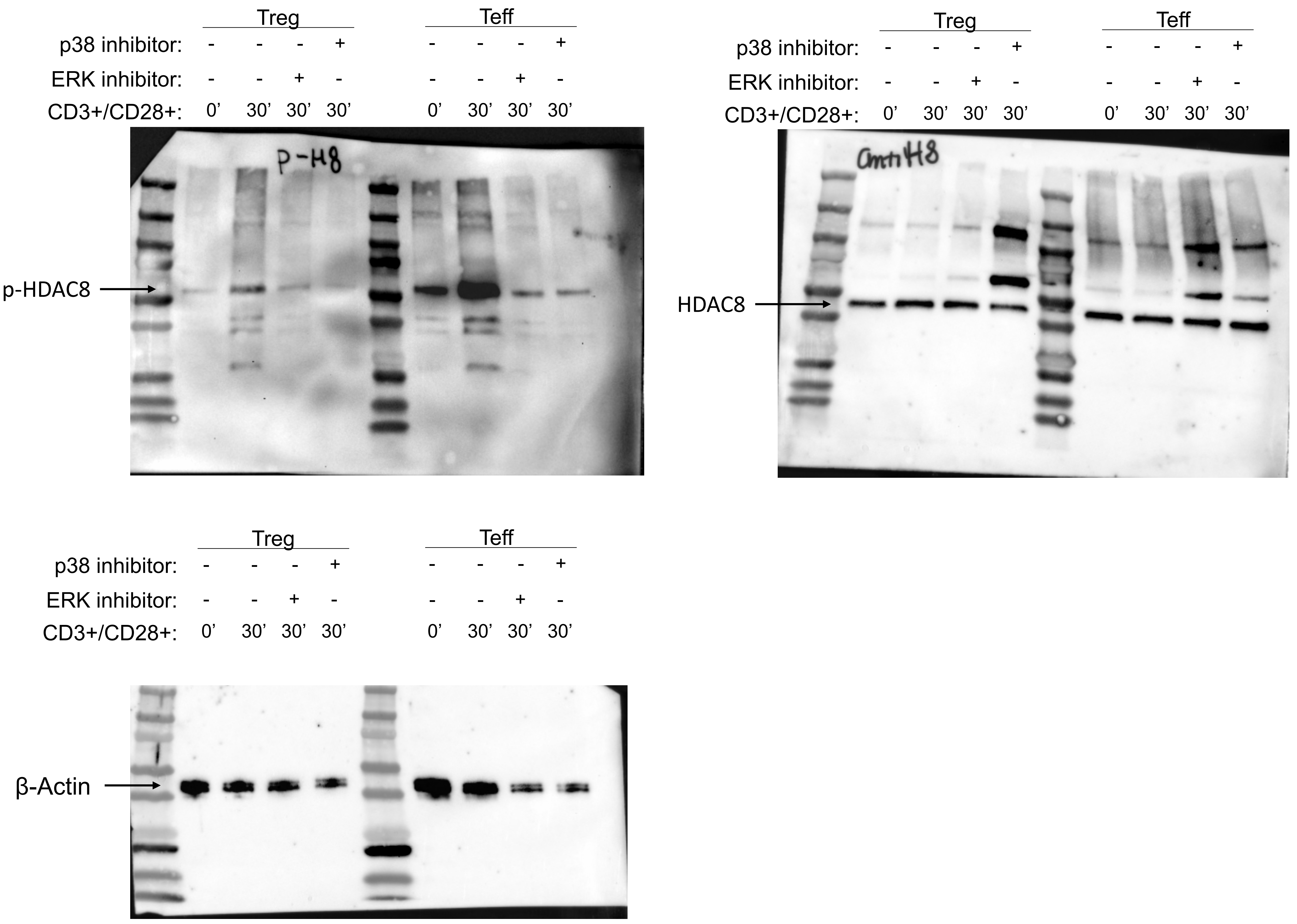


## 9. Full unedited blot/gel for Figure S23:

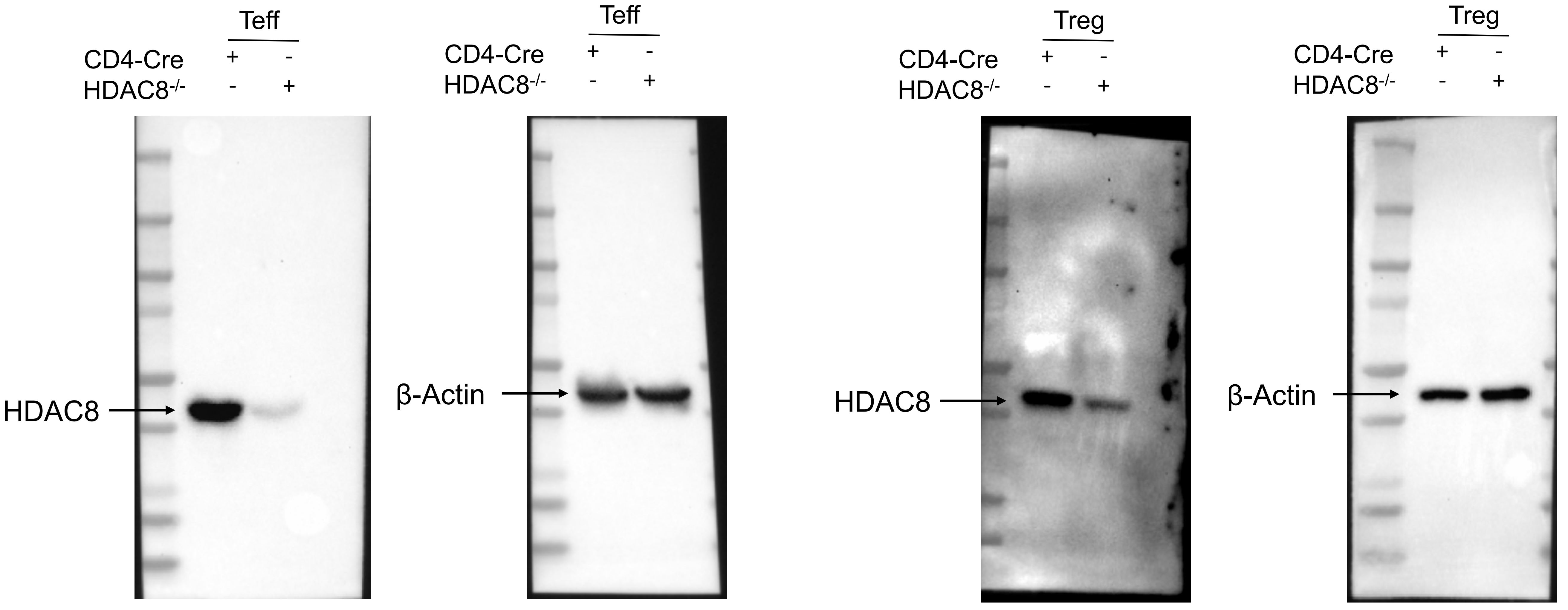

Supplement: Unedited blot and gel images [file jciinsight-11-186461-s049.pdf]
